# Abiraterone acetate and prednisolone with or without enzalutamide for high-risk non-metastatic prostate cancer: a meta-analysis of primary results from two randomised controlled phase 3 trials of the STAMPEDE platform protocol

**DOI:** 10.1016/S0140-6736(21)02437-5

**Published:** 2022-01-29

**Authors:** Gerhardt Attard, Laura Murphy, Noel W Clarke, William Cross, Robert J Jones, Christopher C Parker, Silke Gillessen, Adrian Cook, Chris Brawley, Claire L Amos, Nafisah Atako, Cheryl Pugh, Michelle Buckner, Simon Chowdhury, Zafar Malik, J Martin Russell, Clare Gilson, Hannah Rush, Jo Bowen, Anna Lydon, Ian Pedley, Joe M O'Sullivan, Alison Birtle, Joanna Gale, Narayanan Srihari, Carys Thomas, Jacob Tanguay, John Wagstaff, Prantik Das, Emma Gray, Mymoona Alzoueb, Omi Parikh, Angus Robinson, Isabel Syndikus, James Wylie, Anjali Zarkar, George Thalmann, Johann S de Bono, David P Dearnaley, Malcolm D Mason, Duncan Gilbert, Ruth E Langley, Robin Millman, David Matheson, Matthew R Sydes, Louise C Brown, Mahesh K B Parmar, Nicholas D James, Systemic Therapy in Advancing or Metastatic Prostate cancer: Evaluation of Drug Efficacy (STAMPEDE) investigators, Elin Jones, Katherine Hyde, Hilary Glen, Sarah Needleman, Ursula McGovern, Denise Sheehan, Sangeeta Paisey, Richard Shaffer, Mark Beresford, Zafar Malik, Anjali Zarkar, Emilio Porfiri, David Fackrell, Ling Lee, Thiagarajan Sreenivasan, Sue Brock, Simon Brown, Amit Bahl, Mike Smith-Howell, Cathryn Woodward, Mau-Don Phan, Danish Mazhar, Krishna Narahari, Jacob Tanguay, Fiona Douglas, Anil Kumar, Abdel Hamid, Azman Ibrahim, Dakshinamoorthy Muthukumar, Matthew Simms, Jane Worlding, Anna Tran, Mohammed Kagzi, Prantik Das, Carmel Pezaro, Virgil Sivoglo, Benjamin Masters, Pek Keng-Koh, Caroline Manetta, Duncan McLaren, Nishi Gupta, Denise Sheehan, Stergios Boussios, Henry Taylor, John Graham, Carla Perna, Lucinda Melcher, Warren Grant, Katherine Hyde, Ami Sabharwal, Uschi Hofmann, Robert Dealey, Neil McPhail, Robert Brierly, Simon Brown, Lisa Capaldi, Norma Sidek, Peter Whelan, Thiagarajan Sreenivasan, Peter Robson, Alison Falconer, Sarah Rudman, Sindu Vivekanandan, Vinod Mullessey, Sarah Needleman, Maria Vilarino-Varela, Vincent Khoo, Karen Tipples, Mehran Afshar, Alison Falconer, Patryk Brulinski, Vijay Sangar, Clive Peedell, Ashraf Azzabi, Peter Hoskin, Viwod Mullassery, Santhanam Sundar, Yakhub Khan, Ruth Conroy, Andrew Protheroe, Judith Carser, Paul Rogers, Lisa Capaldi, Kathryn Tarver, Stephanie Gibbs, Mohammad Muneeb Khan, Mohan Hingorani, Ashraf Azzabi, Simon Crabb, Manal Alameddine, Neeraj Bhalla, Caroline Manetta, Robert Hughes, John Logue, Darren Leaning, Salil Vengalil, Ashraf Azzabi, Daniel Ford, Georgina Walker, Ahmed Shaheen, Omar Khan, Andrew Chan, Imtiaz Ahmed, Serena Hilman, Fiona Douglas, Anil Kumar, Anna Tran, Sangeeta Paisey, Ian Sayers, Lisa Capaldi, Ashok Nikapota, David Bloomfield, Tim Porter, Joji Joseph, Cyrill Rentsch, Ricardo Pereira Mestre, Enrico Roggero, Jörg Beyer, Markus Borner, Raeto Strebel, Dominik Berthold, Daniel Engeler, Hubert John, Razvan Popescu, Donat Durr

**Affiliations:** a Cancer Institute, University College London, London, UK; b University College London Hospitals, London, UK; c MRC Clinical Trials Unit at University College London, London, UK; d The Christie and Salford Royal NHS Foundation Trusts, Manchester, UK; e St James's University Hospital, Leeds, UK; f Beatson West of Scotland Cancer Centre, Glasgow, UK; g Royal Marsden NHS Foundation Trust and Institute of Cancer Research, London, UK; h Oncology Institute of Southern Switzerland, Bellinzona, Switzerland; i Universita della Svizzera Italiana, Lugano, Switzerland; j Guy's and St Thomas' NHS Foundation Trust, London, UK; k Clatterbridge Cancer Centre NHS Foundation Trust, Wirral, UK; l University of Glasgow Institute of Cancer Sciences, Glasgow, UK; m Cheltenham General Hospital, Cheltenham, UK; n Torbay and South Devon NHS Foundation Trust, Torbay, UK; o Northern Centre for Cancer Care, Newcastle upon Tyne, UK; p Queen's University Belfast, Belfast, UK; q Royal Preston Hospital, Preston, UK; r Queen Alexandra Hospital, Portsmouth, UK; s Shrewsbury and Telford Hospital NHS Trust, Shrewsbury, UK; t Kent Oncology Centre, Maidstone, UK; u Velindre Hospital, Cardiff, UK; v Singleton Hospital, Swansea, UK; w Royal Derby Hospital, Derby, UK; x Yeovil District Hospital NHS Foundation Trust, Yeovil, UK; y Musgrove Park Hospital, Taunton, UK; z Weston Park Hospital, Sheffield, UK; aa Lancashire Teaching Hospitals NHS Foundation Trust, Preston, UK; ab Royal Sussex County Hospital, Brighton, UK; ac University Hospitals Birmingham NHS Foundation Trust, Birmingham, UK; ad Inselspital Universitatsspital Bern, Bern, Switzerland; ae Cardiff University, Cardiff, UK; af Faculty of Education Health and Wellbeing, University of Wolverhampton, Walsall, UK

## Abstract

**Background:**

Men with high-risk non-metastatic prostate cancer are treated with androgen-deprivation therapy (ADT) for 3 years, often combined with radiotherapy. We analysed new data from two randomised controlled phase 3 trials done in a multiarm, multistage platform protocol to assess the efficacy of adding abiraterone and prednisolone alone or with enzalutamide to ADT in this patient population.

**Methods:**

These open-label, phase 3 trials were done at 113 sites in the UK and Switzerland. Eligible patients (no age restrictions) had high-risk (defined as node positive or, if node negative, having at least two of the following: tumour stage T3 or T4, Gleason sum score of 8–10, and prostate-specific antigen [PSA] concentration ≥40 ng/mL) or relapsing with high-risk features (≤12 months of total ADT with an interval of ≥12 months without treatment and PSA concentration ≥4 ng/mL with a doubling time of <6 months, or a PSA concentration ≥20 ng/mL, or nodal relapse) non-metastatic prostate cancer, and a WHO performance status of 0–2. Local radiotherapy (as per local guidelines, 74 Gy in 37 fractions to the prostate and seminal vesicles or the equivalent using hypofractionated schedules) was mandated for node negative and encouraged for node positive disease. In both trials, patients were randomly assigned (1:1), by use of a computerised algorithm, to ADT alone (control group), which could include surgery and luteinising-hormone-releasing hormone agonists and antagonists, or with oral abiraterone acetate (1000 mg daily) and oral prednisolone (5 mg daily; combination-therapy group). In the second trial with no overlapping controls, the combination-therapy group also received enzalutamide (160 mg daily orally). ADT was given for 3 years and combination therapy for 2 years, except if local radiotherapy was omitted when treatment could be delivered until progression. In this primary analysis, we used meta-analysis methods to pool events from both trials. The primary endpoint of this meta-analysis was metastasis-free survival. Secondary endpoints were overall survival, prostate cancer-specific survival, biochemical failure-free survival, progression-free survival, and toxicity and adverse events. For 90% power and a one-sided type 1 error rate set to 1·25% to detect a target hazard ratio for improvement in metastasis-free survival of 0·75, approximately 315 metastasis-free survival events in the control groups was required. Efficacy was assessed in the intention-to-treat population and safety according to the treatment started within randomised allocation. STAMPEDE is registered with ClinicalTrials.gov, NCT00268476, and with the ISRCTN registry, ISRCTN78818544.

**Findings:**

Between Nov 15, 2011, and March 31, 2016, 1974 patients were randomly assigned to treatment. The first trial allocated 455 to the control group and 459 to combination therapy, and the second trial, which included enzalutamide, allocated 533 to the control group and 527 to combination therapy. Median age across all groups was 68 years (IQR 63–73) and median PSA 34 ng/ml (14·7–47); 774 (39%) of 1974 patients were node positive, and 1684 (85%) were planned to receive radiotherapy. With median follow-up of 72 months (60–84), there were 180 metastasis-free survival events in the combination-therapy groups and 306 in the control groups. Metastasis-free survival was significantly longer in the combination-therapy groups (median not reached, IQR not evaluable [NE]–NE) than in the control groups (not reached, 97–NE; hazard ratio [HR] 0·53, 95% CI 0·44–0·64, p<0·0001). 6-year metastasis-free survival was 82% (95% CI 79–85) in the combination-therapy group and 69% (66–72) in the control group. There was no evidence of a difference in metatasis-free survival when enzalutamide and abiraterone acetate were administered concurrently compared with abiraterone acetate alone (interaction HR 1·02, 0·70–1·50, p=0·91) and no evidence of between-trial heterogeneity (*I*^2^ p=0·90). Overall survival (median not reached [IQR NE–NE] in the combination-therapy groups *vs* not reached [103–NE] in the control groups; HR 0·60, 95% CI 0·48–0·73, p<0·0001), prostate cancer-specific survival (not reached [NE–NE] *vs* not reached [NE–NE]; 0·49, 0·37–0·65, p<0·0001), biochemical failure-free-survival (not reached [NE–NE] *vs* 86 months [83–NE]; 0·39, 0·33–0·47, p<0·0001), and progression-free-survival (not reached [NE–NE] *vs* not reached [103–NE]; 0·44, 0·36–0·54, p<0·0001) were also significantly longer in the combination-therapy groups than in the control groups. Adverse events grade 3 or higher during the first 24 months were, respectively, reported in 169 (37%) of 451 patients and 130 (29%) of 455 patients in the combination-therapy and control groups of the abiraterone trial, respectively, and 298 (58%) of 513 patients and 172 (32%) of 533 patients of the combination-therapy and control groups of the abiraterone and enzalutamide trial, respectively. The two most common events more frequent in the combination-therapy groups were hypertension (abiraterone trial: 23 (5%) in the combination-therapy group and six (1%) in control group; abiraterone and enzalutamide trial: 73 (14%) and eight (2%), respectively) and alanine transaminitis (abiraterone trial: 25 (6%) in the combination-therapy group and one (<1%) in control group; abiraterone and enzalutamide trial: 69 (13%) and four (1%), respectively). Seven grade 5 adverse events were reported: none in the control groups, three in the abiraterone acetate and prednisolone group (one event each of rectal adenocarcinoma, pulmonary haemorrhage, and a respiratory disorder), and four in the abiraterone acetate and prednisolone with enzalutamide group (two events each of septic shock and sudden death).

**Interpretation:**

Among men with high-risk non-metastatic prostate cancer, combination therapy is associated with significantly higher rates of metastasis-free survival compared with ADT alone. Abiraterone acetate with prednisolone should be considered a new standard treatment for this population.

**Funding:**

Cancer Research UK, UK Medical Research Council, Swiss Group for Clinical Cancer Research, Janssen, and Astellas.

## Introduction

The majority of men who die from prostate cancer in Europe and North America are non-metastatic at diagnosis.^1^, ^2^ 3 years of androgen-deprivation therapy (ADT; orchiectomy or gonadotropin-releasing hormone agonists or antagonists) and local radiotherapy are the mainstay of treatment for non-metastatic prostate cancer with high-risk features.^3^, ^4^, ^5^, ^6^ It is known that combining ADT with docetaxel or second-generation hormone treatment (abiraterone, enzalutamide, or apalutamide) improves the outcome of metastatic prostate cancer.^7^, ^8^, ^9^, ^10^, ^11^, ^12^, ^13^ However, to date, none of these drugs has demonstrated a clear and consistent improvement in the survival of patients with non-metastatic prostate cancer starting long-term ADT.^14^, ^15^, ^16^

**Research in context**

**Evidence before this study**

We used a range of terms, including “non-metastatic prostate cancer and docetaxel or abiraterone or apalutamide or enzalutamide or androgen receptor blockade or androgen deprivation therapy”, to search MEDLINE (Jan 1, 1966, to June 30, 2021), Embase (Jan 1, 1982, to June 30, 2021), and major urology and oncology conference proceedings (Jan 1, 1990 to June 30, 2021) for randomised controlled trials, published in English, of abiraterone, enzalutamide, apalutamide, or docetaxel added to 3-year androgen-deprivation therapy (ADT) alone or in combination with local radiotherapy for men with non-metastatic prostate cancer. In three trials, one conducted in the STAMPEDE platform protocol, the NRG Oncology/RTOG 0521 trial, and the GETUG-12 trial, docetaxel added to ADT prolonged time to relapse but neither metastasis-free survival nor overall survival. Another trial in the STAMPEDE platform protocol reported a survival benefit of adding abiraterone to ADT in both metastatic and non-metastatic patients but the latter group had insufficient events for certainty of the treatment benefit.

**Added value of this study**

To our knowledge, this is the first report of a systemic treatment that when added to ADT improves metastasis-free and overall survival of men with non-metastatic, high-risk prostate cancer. Our pooled analysis on 1974 patients randomly assigned in two phase 3 trials conducted in the STAMPEDE protocol shows convincing evidence of treatment benefit with 2-year abiraterone with or without enzalutamide. Additionally, our analysis identifies more toxicity but no evidence of a difference in treatment effect from combination of enzalutamide and abiraterone compared with abiraterone alone.

**Implications of all the available evidence**

Our results suggest a clear improvement in metastasis-free and overall survival from the addition of 2 years of abiraterone to ADT in men with high-risk non-metastatic prostate cancer. The addition of enzalutamide to abiraterone does not appear justified as additional toxicity and cost come with no evidence of a difference in treatment effect. Non-metastatic patients relapsing after previous treatment were under-represented and, although they might also benefit from treatment, further trials are required for certainty of the treatment effect in this group. Abiraterone for 2 years should now be considered a standard treatment option in addition to 3-year ADT for newly diagnosed non-metastatic prostate cancer with high-risk features.

Abiraterone acetate (hereafter referred to as abiraterone) is a selective, irreversible inhibitor of CYP17, which results in more effective androgen depletion than ADT alone.^17^ It is combined with prednisone to reduce side-effects of mineralocorticoid excess.^18^ Enzalutamide is a potent androgen receptor antagonist.^19^ Combining enzalutamide or apalutamide, a related androgen receptor antagonist, with abiraterone has been evaluated and reported for patients before prostatectomy or with castration-resistant prostate cancer, but not in patients starting long-term ADT.^20^, ^21^, ^22^, ^23^, ^24^ We used meta-analysis methods to pool new data from two randomised controlled phase 3 trials conducted in the Systemic Therapy in Advancing or Metastatic Prostate cancer: Evaluation of Drug Efficacy (STAMPEDE, MRC-PR08) platform that, using a multiarm, multistage (MAMS) protocol, have randomised non-metastatic patients between ADT and ADT with abiraterone and prednisolone or ADT and ADT with abiraterone, prednisolone, and enzalutamide. The primary outcome selected for this meta-analysis was metastasis-free survival after the intermediate clinical endpoints for prostate cancer (ICECaP) consortium showed this measure was a surrogate of overall survival in non-metastatic patients.^25^

## Methods

### Study design and participants

The STAMPEDE MAMS platform rationale and design have been previously described.^7^, ^13^ The protocol was sponsored by the UK Medical Research Council (MRC) from April 6, 2004, to Aug 1, 2013, and thereafter by University College London (UCL; London, UK). The study was done at 113 UK and Swiss sites, including hospitals and oncology centres. The protocol recruited patients with advanced prostate cancer starting ADT stratified by the presence or absence of distant metastases on conventional imaging (whole-body bone scintigraphy or equivalent, CT or MRI of the pelvis, abdomen, and chest, and a chest x-ray if the chest was omitted) within 8 weeks of randomisation.

Two separate trials, one comparing ADT with ADT plus abiraterone and prednisolone and another comparing ADT with ADT plus abiraterone, prednisolone, and enzalutamide, with the same eligibility criteria and no overlapping control patients have been undertaken to evaluate the efficacy of adding abiraterone with prednisolone (henceforth called abiraterone trial) or abiraterone with prednisolone and enzalutamide (henceforth referred to as abiraterone and enzalutamide trial). Metastatic and non-metastatic patients were managed differently and the metastatic patients have been reported separately for long-term outcomes.^26^, ^27^ Because of the data that emerged after completion of accrual to both trials, namely the greater than expected efficacy of abiraterone and prednisolone in metastatic patients^7^ and the absence of a survival benefit from combination of enzalutamide with abiraterone and prednisone in metastatic castration-resistant prostate cancer,^20^ we concluded that we would be unable to observe a difference in treatment effect between the two trials. Given the clinical need to determine the efficacy of treatment intensification in non-metastatic patients, a set of decisions was made by the trial management group before inspection of any efficacy outcomes by randomised group in the abiraterone and enzalutamide trial and with no subsequent efficacy analysis of non-metastatic patients in the abiraterone trial. These decisions were to (1) formally separately report non-metastatic and metastatic patients, (2) combine the non-metastatic patients from both trials (both trials assigned control patients to the same standard-of-care treatment, had no overlapping control patients and included the same dose and regimen of abiraterone acetate, given either with prednisolone or with both prednisolone and enzalutamide), (3) change the primary outcome measure for the non-metastatic population from overall survival to metastasis-free survival, and (4) extend follow-up of both trials until sufficient events in the non-metastatic population. This change in reporting plan was approved by the trial steering committee, which functions independently from the trial management group, on Dec 2, 2019, and was subsequently published as a prespecified declaration of our intentions.^28^

Full details on the patient population are provided in the protocol. In summary, eligible patients had prostate adenocarcinoma confirmed histologically, WHO performance status 0–2 (on a scale of 0–4, with higher numbers indicating greater disability), no evidence of distant metastases on conventional imaging, and were either node positive or, if node negative, were either high risk (defined as having at least two of the following: tumour stage T3 or T4, Gleason sum score of 8–10, and prostate-specific antigen [PSA] concentration ≥40 ng/mL) or relapsing with high-risk features (≤12 months of total ADT with an interval of ≥12 months without treatment and a PSA concentration ≥4 ng/mL with a doubling time of <6 months or a PSA concentration ≥20 ng/mL). There were no age restrictions. Patients with confirmed clinically significant cardiovascular disease (eg, severe angina, recent myocardial infarction, or a history of cardiac failure) were excluded.

The STAMPEDE protocol is conducted in accordance with Good Clinical Practice guidelines and the Declaration of Helsinki. Ethics approval was granted by West Midlands Research Ethics Committee (REC), now West Midlands, Edgbaston REC (REC number 04/MRE07/35), and all patients were required to provide written informed consent. Regulatory approval was granted in the UK under clinical trials authorisation 20363/0404 (previously 00316/0026) and in Switzerland under 2009 DR 3235. Full details for the trial can be found in the protocol and statistical analysis plan (appendix) or online.

### Randomisation and masking

Patients were randomly assigned (1:1) to standard of care alone (control group) or with combination therapy. Randomisation was performed centrally by telephone with the use of a computerised algorithm, which was developed and maintained by the MRC Clinical Trials Unit at UCL. Minimisation with a random element of 80% was used, with stratification according to randomising centre, age at randomisation (<70 *vs* ≥70 years), planned use of prostate radiotherapy (yes *vs* no), nodal involvement (negative *vs* indeterminate *vs* positive), WHO performance status (0 *vs* 1–2), type of ADT, and regular, long-term use of aspirin or non-steroidal anti-inflammatory drugs (yes *vs* no). Both trials were open label because masking of the treatment assignment was deemed impracticable. Eligible patients could be assigned to any of the trials that were contemporaneously recruiting patients in STAMPEDE; we focus here on non-metastatic patients assigned to the abiraterone or abiraterone and enzalutamide trials.

### Procedures

The protocol recommended that patients received standard-of-care treatment with 3 years ADT, which could include surgery and luteinising-hormone-releasing hormone agonists and antagonists (details in the protocol in the appendix), that started no longer than 12 weeks before randomisation. Radiotherapy (as per local guidelines, 74 Gy in 37 fractions to the prostate and seminal vesicles or the equivalent using hypofractionated schedules; details are shown in the protocol in the appendix) after randomisation was mandated (unless contraindicated) for patients with node-negative disease and encouraged for node-positive disease. Abiraterone acetate (1000 mg) with prednisolone (prednisone at Swiss sites, 5 mg) alone or with enzalutamide (160 mg), hereafter referred to as combination therapy, were given orally once daily and were to continue for 2 years or until progression, whichever came first. When radiotherapy was omitted, treatment could continue until disease progression. Dose modifications are described in the protocol.

Patients were assessed for the trial 6-weekly during the first 6 months, 12-weekly until year 2, 6-monthly until year 5, then annually. Assessments included PSA testing and ascertainment of adverse events (data on dose modifications or interruptions will not be shown); further tests were conducted at the discretion of the treating physician. The nadir PSA concentration (for the definition of PSA progression see protocol) was defined as the lowest level within 24 weeks after randomisation. After randomisation, follow-up imaging (using the same modality as the one used for that patient at screening) was as per local guidelines. Adverse events were assessed by the National Cancer Institute Common Terminology Criteria for Adverse Events (initially, version 3.0; later, version 4.0). Serious adverse events and reactions were reported accordingly.

### Outcomes

The primary endpoint of this meta-analysis was metastasis-free-survival, defined as time from randomisation to death from any cause or to distant metastases confirmed by imaging. Secondary endpoints were overall survival (defined as time from randomisation to death); prostate cancer-specific survival (defined as time from randomisation to death from prostate cancer); failure-free survival, defined as time from randomisation to biochemical failure, local progression, distant metastases, or death from prostate cancer; progression-free-survival defined as failure-free survival but excluding biochemical failure; and toxicity and adverse events. Ascertainment of death from prostate cancer was determined using a prespecified algorithm or manual review by a panel of clinicians according to an agreed set of rules and without knowledge of randomised group.

### Statistical analysis

The original sample size calculations for each trial are available in the protocol. Both trials recruited to their required overall target sample size; there was no predefined sample size for non-metastatic patients. To determine how many metastasis-free survival events were required to be reported in the control patients, we used the nstage function within Stata (Stata Corp, Texas, TX, USA; version 16.1). Assuming a metastasis-free-survival of 70% at 5·5 years for control patients, we targeted a 25% relative improvement between the combination-therapy and control groups (target hazard ratio [HR] 0·75). For 90% power and a one-sided α level set at 1·25% (to account for previous reporting of overall survival [but not metastasis-free survival] of patients in the abiraterone trial^7^), we required approximately 315 metastasis-free survival events in the control groups. Data were analysed according to a prespecified statistical analysis plan (appendix) using Stata, version 16).

All patients were included in the efficacy analyses under their assigned treatment on an intention-to-treat basis. For toxicity and adverse events, patients were included if any treatment was administered as per randomised allocation.

The median follow-up calculation used a reverse Kaplan-Meier method, censoring on death or withdrawal. Fixed-effects individual patient data meta-analyses were used to pool estimates from both trials. Standard survival analysis methods including Cox proportional hazards regression and Kaplan-Meier curves were used to analyse and present time-to-event outcomes between randomised groups. Estimates were adjusted for stratification factors (except randomising centre and ADT type) and were stratified according to time periods defined by other recruiting trials. Patients without an event of interest were censored when they were last known to be event-free according to a received follow-up case record form. The proportional hazards assumption was tested using scaled Schoenfeld residuals regressed over time for the primary endpoint and all secondary endpoints. Prespecified subgroup (nodal status, age, WHO performance status, non-steroidal anti-inflammatory drugs or aspirin use, and planned radiotherapy) analyses of metastasis-free survival and overall survival explored the consistency of treatment effect between both trials and across randomisation stratification factors. The proportion of heterogeneity between trials was calculated based on Cochran's Q test (quantified by the *I*^2^ value). Prostate cancer-specific survival used a competing risks approach with death from non-prostate cancer causes as the competing risk.

STAMPEDE is registered with ClinicalTrials.gov, NCT00268476, and with the ISRCTN registry, ISRCTN78818544.

### Role of the funding source

Cancer Research UK provided peer review of the study design and Janssen and Astellas Pharma approved the final design. The funders had no role in data collection, data analysis, data interpretation, or writing of the manuscript and the decision to submit; representatives from Janssen and Astellas Pharma were invited to comment on the manuscript. Interpretation and the decision to submit the manuscript for publication were made by the trial management group.

## Results

**Figure 1:**
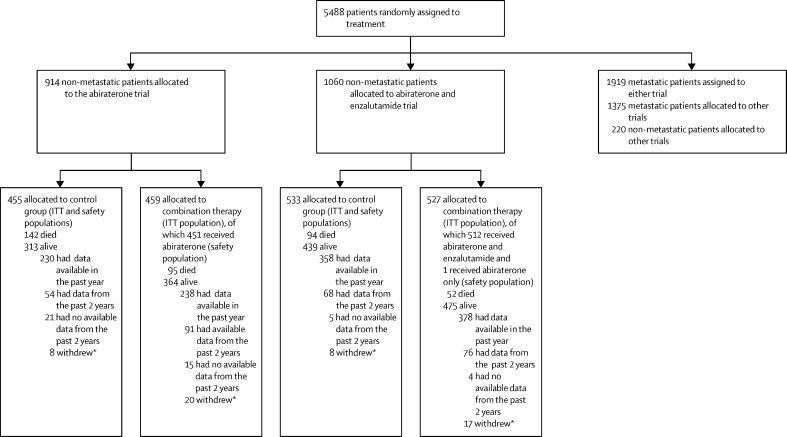
Study profile ITT=intention-to-treat. *Withdrawn patients include both patients with no further data collection allowed and those withdrawn from follow-up but some data collection allowed; the number shown is those who have withdrawn and do not have any data in the past 2 years.

Between Nov 15, 2011, and March 31, 2016, 1974 non-metastatic patients were randomly assigned in both trials (455 to the control group and 459 to the combination-therapy group of the abiraterone trial from Nov 15, 2011, to Jan 17, 2014, and 533 to the control group and 527 to the combination-therapy group of the abiraterone and enzalutamide trial from July 29, 2014, to March 31, 2016). Database lock and extraction occurred on Aug 3, 2021. The flow of patients in these two trials is shown in figure 1 and other contemporaneously recruiting trials are shown in the appendix (p 10). The baseline characteristics of the patients are shown in table 1 and the appendix (pp 3–4), and were well balanced between randomised groups. The median age was 68 years (IQR 63–73), median PSA was 34 ng/ml (IQR 15–74), 774 (39%) of 1974 patients were node-positive, and 1563 (79%) of 1968 had a Gleason score sum of 8–10. The median follow-up was 72 months (IQR 63–73; 85 months [83–96] in the abiraterone trial and 60 months [59–71] in the abiraterone and enzalutamide trial).

**Table 1:** Baseline characteristics

	**Control group in the abiraterone trial (n=455)**	**Control group in the abiraterone and enzalutamide trial (n=533)**	**Combination therapy group in the abiraterone trial (n=459)**	**Combination therapy group in the abiraterone and enzalutamide trial (n=527)**
**Age at randomisation, years**				
Median (IQR)	67 (62–73)	69 (64–73)	68 (63–73)	68 (63–73)
Range	48–83	43–86	44–84	46–86
**PSA at randomisation, ng/ml**				
Median (IQR)	40 (16–83)	34 (15–74)	34 (15–68)	32 (13–74)
Range	1–1000	1–2773	1–2300	0–556
**Nodal status of newly diagnosed patients**				
N0	256 (56%)	327 (61%)	253 (55%)	325 (62%)
N1	187 (41%)	190 (36%)	181 (39%)	187 (35%)
**Nodal status of relapsed patients**				
N0	7 (2%)	8 (2%)	14 (3%)	7 (1%)
N1	5 (1%)	7 (1%)	10 (2%)	7 (1%)
Nx	0	1 (<1%)	1 (<1%)	1 (<1%)
**WHO performance status**				
0	375 (82%)	435 (82%)	370 (71%)	429 (81%)
1–2	80 (17%)	98 (18%)	89 (19%)	98 (19%)
**Time from diagnosis to randomisation**				
Median (IQR)	83 (63–107)	84 (65–104)	83 (62–105)	85 (68–105)
Range	5–2771	4–4807	1–5274	2–5434
**Gleason sum score**				
<8	105 (23%)	95 (18%)	107 (23%)	98 (19%)
8–10	348 (76%)	437 (82%)	351 (77%)	427 (81%)
Missing	2	1	1	2
**T stage at randomisation**				
T0–T2	39 (9%)	30 (6%)	30 (7%)	26 (5%)
T3–T4	41 (90%)	496 (93%)	423 (92%)	493 (94%)
TX	5 (1%)	7 (1%)	6 (1%)	8 (2%)
**Local radiotherapy as standard of care**				
No	83 (18%)	62 (12%)	87 (19%)	58 (11%)
Yes	372 (82%)	471 (88%)	372 (81%)	469 (89%)

Data are n (%) or n, unless stated otherwise. PSA=prostate-specific antigen.

The median time from randomisation to the initiation of combination therapy was 1·4 weeks (IQR 1·0–2·7) and the median time from the initiation of ADT to the initiation of combination therapy was 8·4 weeks (5·1–11·3; most patients started ADT before randomisation). Of 459 patients randomly assigned to abiraterone and prednisolone, 451 started treatment; of 527 patients randomly assigned to abiraterone, prednisolone, and enzalutamide, 512 started both treatments and one patient started only abiraterone acetate and prednisolone. The median time to permanently stopping abiraterone was 23·7 months (IQR: 17·6–24·1) when assigned alone and 20·7 months (4·4–24) when assigned in combination with enzalutamide and to stopping enzalutamide was 23·2 months (6·3–24; appendix p 10); 266 (59%) of 451 patients randomly assigned to abiraterone and prednisolone and 222 (43%) of 513 randomly assigned to abiraterone acetate, prednisolone, and enzalutamide reported stopping for planned treatment completion (appendix p 5). 84 (9%) of 964 patients continued treatment beyond 24 months. The planned rate of use of local radiotherapy was 1684 (85%) of 1974, namely for newly diagnosed patients, 99% for node-negative patients and 71% for node-positive patients, and 7% for previously treated patients.

**Figure 2:**
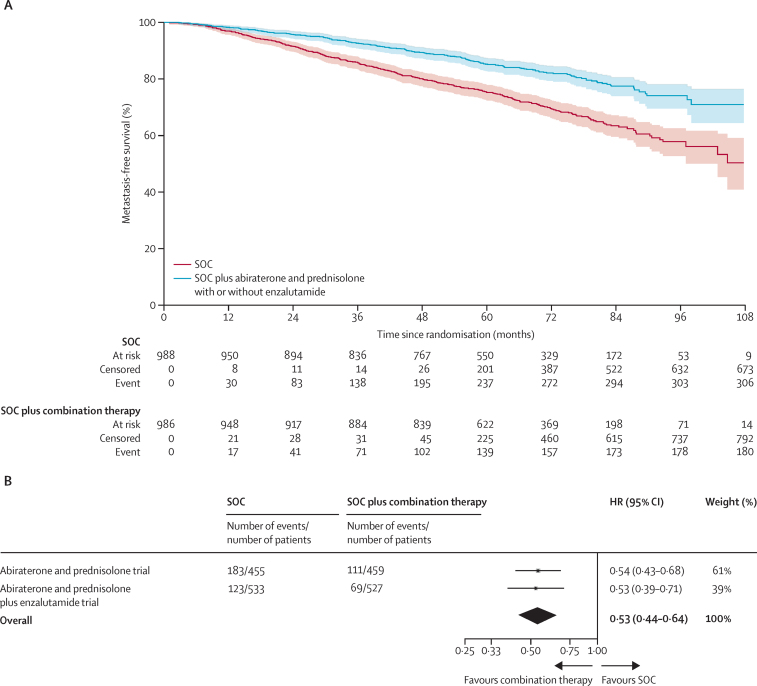
Metastasis-free survival (A) Kaplan-Meier estimates of all patients in individual patient data meta-analysis; shaded regions represent 95% CIs. (B) Prespecified subgroup analysis by trial. HR=hazard ratio. SOC=standard of care.

**Figure 3:**
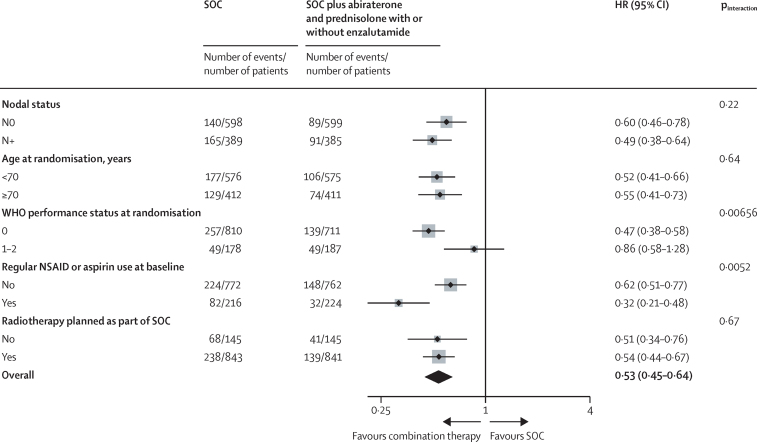
Forest plots of treatment effect on metastasis-free survival for baseline randomisation stratification factors (except recruiting centre and type of androgen-deprivation therapy) Weighting is by sample size. Three patients were excluded from the nodal status subgroup analysis as Nx. HR=hazard ratio. NSAID=non-steroidal anti-inflammatory drug. SOC=standard of care.

There were 180 metastasis-free survival events in the combination-therapy groups and 306 in the control groups. Of the 180 metastasis-free survival events in the combination-therapy groups, 93 (52%) were deaths and 87 (48%) were a report of extra-pelvic metastases. Of the 306 events in the control groups, 117 (38%) were deaths and 189 (62%) were a report of metastases (appendix p 6). Metastasis-free survival was significantly longer in the combination-therapy groups (median not reached, IQR not estimable [NE]–NE) than in the control groups (not reached, 97–NE; HR 0·53, 95% CI 0·44–0·64; p<0·0001), with 6-year metastasis-free survival improved from 69% in the control groups to 82% in the combination-therapy groups (figure 2). There was no evidence of non-proportional hazards in the treatment effect (p=0·46). A preplanned subgroup analysis included 294 metastasis-free survival events in the abiraterone trial and 192 in the abiraterone and enzalutamide trial. This analysis showed a strong effect in each trial separately (abiraterone trial HR 0·54, 95% CI 0·43–0·68, p<0·0001; abiraterone and enzalutamide trial 0·53, 0·39–0·71, p<0·0001) with no evidence of a difference in treatment effect (interaction HR 1·02, 0·70–1·50, p=0·91) and no evidence of between-trial heterogeneity (*I*^2^ p=0·90; figure 2, appendix p 11). In analysis of subgroups defined by randomisation stratification factors, statistically significant heterogeneity of effect was observed for WHO performance status 1–2 or use of non-steroidal anti-inflammatory drugs or aspirin (figure 3).

**Figure 4:**
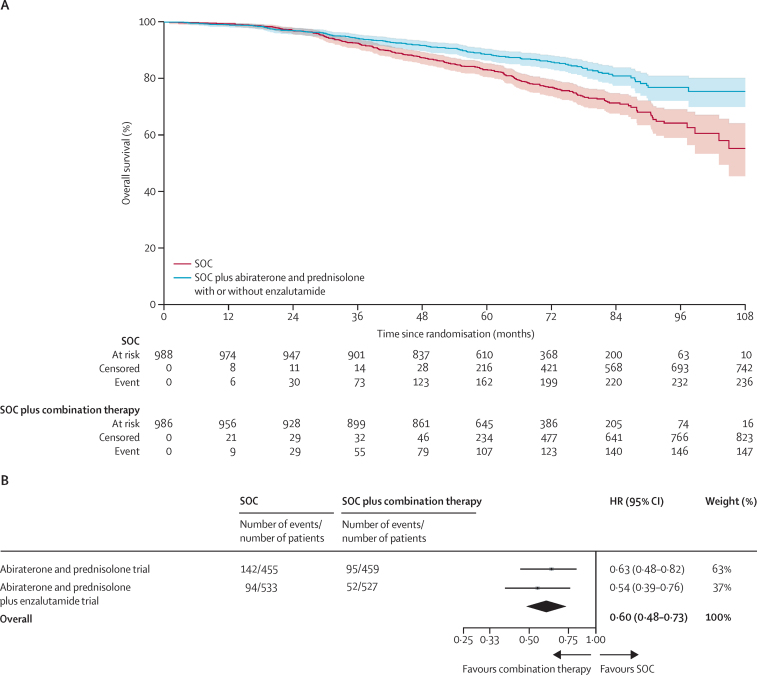
Overall survival (A) Kaplan-Meier estimates of all patients in individual patient data meta-analysis; shaded regions represent 95% CIs. (B) Prespecified subgroup analysis by trial. HR=hazard ratio. SOC=standard of care.

There were 147 deaths in the combination-therapy groups and 236 in the control groups. Overall survival was significantly longer in the combination-therapy groups (median not reached, IQR NE–NE) than in the control groups (not reached, 103–NE; HR 0·60, 95% CI 0·48–0·73, p<0·0001) with 6-year survival improved from 77% in the control groups to 86% in the combination-therapy groups (figure 4, appendix p 6). There was no evidence of non-proportional hazards in the treatment effect (p=0·10). A preplanned subgroup analysis included 237 deaths in the abiraterone trial and 146 deaths in the abiraterone and enzalutamide trial. The effect was strongly observed in both trials (abiraterone trial HR 0·63, 95% CI 0·48–0·82, p=0·0005; abiraterone and enzalutamide trial 0·54, 0·39–0·76, p=0·0004) with no evidence of between-trial heterogeneity (*I*^2^ p=0·51; figure 4 and appendix p 12).

**Figure 5:**
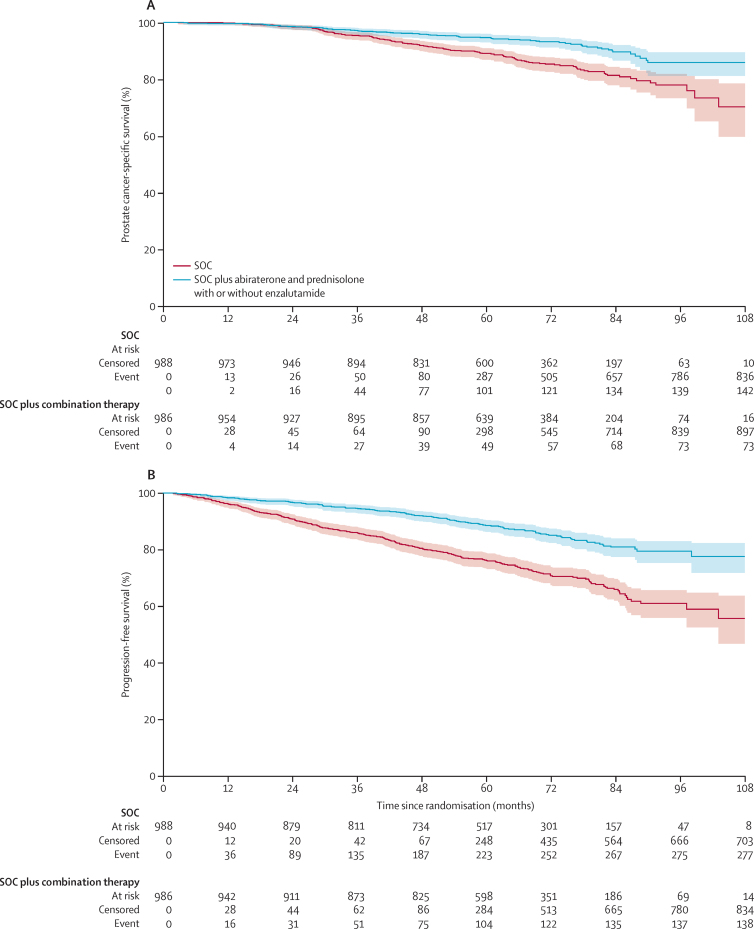
Kaplan-Meier estimates of prostate cancer-specific survival (A) and progression-free survival (B) Kaplan-Meier estimates of all patients in individual patient data meta-analysis; shaded regions represent 95% CIs. SOC=standard of care.

73 (50%) of 147 deaths in the combination-therapy groups and 142 (60%) of 236 deaths in the control groups were attributed to prostate cancer. Prostate-cancer-specific survival was significantly improved in the combination-therapy groups (median not reached, IQR NE–NE) compared with the control groups (not reached, NE–NE; HR 0·49, 95% CI 0·37–0·65, p<0·0001), with 6-year prostate-cancer specific survival of 93% in the combination-therapy groups compared with 85% in the control groups (figure 5). There was no evidence of non-proportional hazards (p=0·44) and the effect was consistent in both trials (appendix p 13). There were 138 events of radiological or clinical progression or death from prostate cancer in the combination-therapy groups and 277 in the control groups, with progression-free survival being significantly longer in the combination-therapy groups (median not reached, IQR NE–NE) than in the control groups (not reached, 103–NE; HR 0·44, 95% CI 0·36–0·54, p<0·0001; figure 5). The effect was consistent in both trials (appendix p 13). There were 204 events of treatment failure (including PSA progression) in the combination-therapy groups and 402 in the control groups, with failure-free survival being significantly longer in the combination-therapy groups (median not reached, IQR NE–NE) than in the control groups (86 months, 83–NE; HR 0·39, 95% CI 0·33–0·47, p<0·0001; appendix p 14). The effect was consistent in both trials (appendix p 14).

In the safety population, 130 (29%) of 455 control patients in the abiraterone trial and 172 (32%) of 533 control patients in the abiraterone and enzalutamide trial had grade 3 or worse adverse events during the first 24 months (table 2, appendix p 7). In the combination-therapy groups, 169 (37%) of 451 patients in the abiraterone trial and 298 (57%) of 513 patients in the abiraterone and enzalutamide trial had grade 3 or worse adverse events during the first 24 months. Seven grade 5 events were reported: none in the control groups, three (1%) in the abiraterone and prednisolone group (one [<1%] event each of rectal adenocarcinoma, pulmonary haemorrhage, and a respiratory disorder), and four (1%) in the abiraterone and prednisolone with enzalutamide group (two events [<1%] each of septic shock and sudden death). The most common adverse events reported in the combination-therapy groups compared with the control groups were hypertension (153 ([5%] of 988 in the control groups *vs* 393 [41%] of 964 in the combination-therapy groups) and aminotransaminases (136 [14%] *vs* 332 [34%]; table 2, appendix p 7). The most common grade 3 or worse adverse events when enzalutamide and abiraterone were combined were hypertension (23 [5%] of 451 in the abiraterone trial *vs* 73 [14%] of 513 in abiraterone and enzalutamide trial), fatigue (ten [2%] *vs* 49 [10%]), and increases in aminotransferases (25 [5%] *vs* 69 [13%]; table 2).

**Table 2:** Adverse events in the safety population

	**Control group in the abiraterone trial (n=455)**			**Control group in the abiraterone and enzalutamide trial (n=533)**			**Combination therapy in the abiraterone trial (n=451)**			**Combination therapy in the abiraterone and enzalutamide trial (n=513)**		
	Grade 1–2	Grade 3	Grade 4	Grade 1–2	Grade 3	Grade 4	Grade 1–2	Grade 3	Grade 4	Grade 1–2	Grade 3	Grade 4
Erectile dysfunction	211 (46%)	48 (11%)	0	237 (44%)	55 (10%)	0	209 (46%)	41 (9%)	0	243 (47%)	71 (14%)	0
Hypertension	65 (14%)	6 (1%)	0	74 (14%)	8 (2%)	0	108 (24%)	23 (5%)	0	189 (37%)	73 (14%)	0
ALT increased	51 (11%)	0	0	72 (14%)	4 (1%)	0	82 (18%)	23 (5%)	2 (<1%)	145 (28%)	59 (12%)	5 (1%)
Fatigue	279 (61%)	4 (1%)	NM	371 (70%)	12 (2%)	NM	299 (66%)	10 (2%)	NM	411 (80%)	49 (10%)	NM
AST increased	14 (3%)	1 (<1%)	0	17 (3%)	0	0	33 (7%)	2 (<1%)	0	61 (12%)	17 (3%)	2 (<1%)
Insomnia	126 (28%)	1 (<1%)	NM	162 (30%)	1 (<1%)	NM	133 (29%)	8 (2%)	NM	200 (39%)	7 (1%)	NM
Hypokalemia	4 (1%)	1 (<1%)	0	9 (2%)	1 (<1%)	0	50 (11%)	4 (1%)	1 (<1%)	56 (11%)	6 (1%)	0
Anaemia	142 (31%)	3 (1%)	2 (<1%)	211 (40%)	2 (<1%)	0	185 (41%)	1 (<1%)	1 (<1%)	225 (44%)	2 (<1%)	0
Dizziness	53 (12%)	0	NM	70 (13%)	1 (<1%)	NM	72 (16%)	1 (<1%)	NM	126 (25%)	4 (1%)	NM
Constipation	104 (23%)	3 (1%)	0	149 (28%)	0	0	128 (28%)	1 (<1%)	0	181 (35%)	1 (<1%)	0
Cough	72 (16%)	0	0	107 (20%)	0	0	103 (23%)	5 (1%)	0	103 (20%)	0	0
Nausea	43 (9%)	1 (<1%)	NM	67 (13%)	0	NM	60 (13%)	0	NM	130 (25%)	3 (1%)	NM
Cognitive disturbance	21 (5%)	0	0	29 (5%)	0	0	26 (6%)	2 (<1%)	0	85 (17%)	2 (<1%)	0
Dyspepsia	47 (10%)	0	0	71 (13%)	0	0	64 (14%)	1 (<1%)	0	103 (20%)	2 (<1%)	0
Anorexia	24 (5%)	0	0	41 (8%)	1 (<1%)	0	29 (6%)	0	0	94 (18%)	1 (<1%)	0
Headache	57 (13%)	0	0	75 (14%)	0	0	74 (16%)	0	0	138 (27%)	2 (<1%)	0
Anxiety	NM	NM	NM	52 (10%)	0	0	NM	NM	NM	88 (17%)	1 (<1%)	0
Depression	NM	NM	NM	48 (9%)	0	0	NM	NM	NM	86 (17%)	1 (<1%)	0

Data are n (%). Anxiety and depression included as options in toxicity forms in the abiraterone and enzalutamide trial, but not abiraterone trial. Grade 5 events reported in the text only. ALT=alanine transminase. AST=aspartate transminase. NM=not measured.

## Discussion

MAMS platform protocols have been used in prostate cancer and other diseases to efficiently test therapeutic efficacy.^29^ In prostate cancer, primary analyses from three randomised trials done in the STAMPEDE protocol have, to date, supported a change in the standard of care for men with metastatic prostate cancer, namely addition of docetaxel or abiraterone and prednisolone to ADT and radiotherapy of the primary tumour in low-burden metastatic disease.^7^, ^13^, ^30^

In this primary analysis of high-risk, non-metastatic patients initiating ADT, the magnitude of the beneficial effect from combining with abiraterone-based treatment is notably larger than was estimated from immature data following a shorter duration of follow-up^7^ and was consistent across the primary endpoint and all secondary efficacy outcome measures, including overall survival. This finding contrasts with those that show that docetaxel improves the survival of metastatic patients but, for high-risk non-metastatic patients, although it delays relapse-free survival, it does not have a benefit in terms of metastasis-free or overall survival.^14^, ^15^, ^16^ The proportion of metastasis-free survival events in the primary outcome measure that were attributed to metastasis and of deaths attributed to prostate cancer were lower in the combination-therapy groups. These results might suggest that, by preventing relapse and death from prostate cancer, patients treated with combination therapy are more likely to live longer and die from another cause. The relationship between the treatment effect on metastasis-free and on overall survival is as anticipated from a previous surrogacy analysis of metastasis-free survival in non-metastatic prostate cancer.^25^ Similarly to these trials, imaging scans were done in both our trials as per local clinical guidelines.

Following completion of accrual of both our trials, studies of the combination of abiraterone with enzalutamide or apalutamide in metastatic castration-resistant prostate cancer reported modest improvements in radiographic progression-free survival with no evidence of an improvement in overall survival.^20^, ^23^ We therefore assumed that combining abiraterone and enzalutamide would have no demonstrably different treatment effect compared with abiraterone alone in men starting ADT, which appears to be the case, and the two trials individually provide independent confirmation of the magnitude of benefit for abiraterone-based therapy in non-metastatic prostate cancer. We conclude that, although the benefit from combination therapy is clear in both trials and our indirect comparison cannot exclude a small benefit from combining enzalutamide and abiraterone, the increased toxicity and cost from adding enzalutamide is not currently justified for this population.

Patients were stratified by baseline use of non-steroidal anti-inflammatory drugs or aspirin, included due to previous evaluation of celecoxib (reported previously)^31^ in the platform protocol. There is currently no biological explanation for the evidence of heterogeneity of treatment effect observed in patients receiving these drugs at randomisation and the direction of treatment effect favours combination therapy in both subgroups (ie, patients who received non-steroidal anti-inflammatory drugs or aspirin and those who did not). In the absence of additional biological or clinical data, this observation should not affect clinical management. Subgroup analysis also identified some evidence of a heterogeneity of effect for patients with a reduced performance status, probably secondary to concomitant illnesses. When implementing these results into clinical practice, clinicians are encouraged to balance the treatment benefit reported here with the risk of death from other causes. Moreover, subgroup analyses need to be interpreted with caution because of the subgroups small sample sizes.

Our data have several strengths to note. By pooling events from two large randomised trials that included non-metastatic patients prospectively, followed up in the same platform protocol, we have achieved very clear and consistently significant results. The conduct of both trials was enabled by an academic–pharma partnership and benefited from engagement at more than 100 hospitals across the UK and Switzerland, achieving very rapid accrual and the delivery of a novel result that has immediate worldwide clinical implications given the global access to abiraterone acetate.

Our data also have some limitations. Data on subsequent therapies instituted for metastatic disease or at development of castration resistance are unreliable given this often occurred several years after completion of trial treatment. Nonetheless, second-generation hormone therapies for metastatic castration-resistant prostate cancer were widely available in the health-care jurisdictions where and when the trials were conducted so the implications of our report are relevant to patients managed according to modern-day clinical guidelines. Also, notably, as our trials were open label, it is possible that patients in the control groups received second-generation hormone treatments in clinical trials for non-metastatic castration-resistant prostate cancer or, since 2019, as standard-of-care treatment. For the same reason of reliability long term after completion of trial medication, we only report toxicity during the first 2 years of treatment. Importantly, we are reassured that any effect on survival from additional toxicity in the combination-therapy groups is offset by the efficacy of hormone intensification and consequent improved outcomes. However, further improvements in patient outcomes could be achieved by better understanding and then mitigating against the adverse effects of treatment.

Our study, to our knowledge, is the first to show a large treatment benefit for combination of fixed-duration second-generation hormone therapy with ADT for non-metastatic prostate cancer. The conclusions of this study should be restricted to patients who meet the protocol's definition for disease at high risk of relapse. A few questions remain that could be addressed by ongoing or future studies. Our trials recruited patients across a wide range of presentations of aggressive prostate cancer, selected based on the plan for at least 3 years of ADT. The protocol did not place a preference or limit on accrual by any of the eligibility criteria. Non-metastatic patients relapsing after previous local therapy are under-represented and more studies are required for certainty on the benefit in this group. 2 years of treatment in our study is a pragmatic duration based on the requirement of ADT with radiotherapy for at least 2 years.^6^ Shorter durations of treatment could be as effective and longer might be even more effective, hence treatment duration could be considered for further testing. We have not included data for adding an androgen receptor antagonist alone to ADT and radiotherapy nor for combination therapy in men undergoing prostatectomy. Future studies confirming efficacy in these situations will give treatment options for non-metastatic prostate cancer additional to abiraterone and radiotherapy.

In summary, men with high-risk non-metastatic prostate cancer who receive ADT with combination therapy have significantly better metastases-free survival and overall survival than those who receive ADT alone. 2 years of abiraterone and prednisolone added to ADT and, if indicated, radiotherapy should be considered a new standard treatment for non-metastatic prostate cancer with high-risk features.

## Data sharing

The individual participant data that underlie the results reported in this Article are available upon request and after deidentification as per the moderated access approach of the Medical Research Council Clinical Trials Unit at UCL. Please contact the corresponding author or mrcctu.stampede@ucl.ac.uk for more information.

## Declaration of interests

GA reports personal fees, grants, and travel support from Janssen and Astellas Pharma during the conduct of the study; personal fees or travel support from Pfizer, Ipsen, Novartis/AAA, Abbott Laboratories, Ferring, ESSA Pharmaceuticals, Bayer Healthcare Pharmaceuticals, Beigene, Takeda, AstraZeneca, and Sanofi-Aventis; and grant support from AstraZeneca, Innocrin Pharma, and Arno Therapeutics, outside the submitted work; in addition, GA's former employer, The Institute of Cancer Research, receives royalty income from abiraterone and GA receives a share of this income through the Institute's Rewards to Discoverers Scheme. NWC reports personal fees from Janssen Pharmaceuticals and Astellas Pharma, during the conduct of the study; and personal fees from Bayer outside the submitted work. WC reports personal fees from Janssen and Bayer, outside the submitted work. RJJ reports grants from Sanofi, and grants and non-financial support from Novartis, during the conduct of the study; grants, personal fees, and non-financial support from Sanofi and Novartis, and grants and personal fees from Janssen, Astellas, and Bayer, outside the submitted work. CCP reports personal fees from AAA and Janssen, and research funding and speaker's honoraria from Bayer, outside the submitted work. SG reports personal fees from Bayer, Janssen Cilag, Dendreon Corporation, Millennium Pharmaceuticals, Orion, Sanofi, and MaxiVax; and speaker's fees and travel support from Bayer, CureVac, Janssen Cilag, Astellas, AAA Advanced Accelerator Applications International, Bristol-Myers Squibb (BMS), Ferring, Roche, Orion, lnnocrin Pharmaceuticals, Sanofi, Novartis, Nektar Therapeutics, and ProteoMedix, outside the submitted work. SC reports grants and personal fees from Sanofi-Aventis and personal fees from Janssen Pharmaceutical, outside the submitted work. ZM reports consultancy and advisory board membership for Janssen Consultancy, advisory board membership for Sanofi and Astellas, and sponsorship to attend medical conferences from Astellas, Bayer, and Janssen. JMR reports personal fees from Janssen (lecture fee), outside the submitted work. JB reports payments from Janssen for webinar presentations. AL reports payment or honoraria for lectures, presentation, or events from BMS, and support for attending meetings or travel from Astellas, Bayer, BMS, and Merck Sharpe and Dohme. IP reports sponsorship from Bayer for attending the European Society for Medical Oncology congress virtually in 2021. JMO reports participating on a data safety monitoring board or advisory board for AA, Astellas, Bayer, Janssen, Novartis, and Sanofi. AB reports speaker's fees and travel support from Astellas, and personal fees, speaker's fees, and travel support from Sanofi and Janssen, during the conduct of the study; speaker's fees and travel support from Bayer and AstraZeneca, outside the submitted work; and speaker fee payment from Janssen. NS reports support for attending meetings or travel from Jansen, Astellas, and Eusa Pharma UK. JT reports support for travel and speaker fees from Jansen, Roche, and Bayer, and participating on a data safety monitoring board or advisory board for AstraZeneca, Astellas, and Bayer. JWa reports a paid consultancy for BMS, Eisai, Novartis, and MSD, oustide the submitted work. OP reports participating on a data safety monitoring board and advisory board for Janssen in October, 2021. IS reports support for attending meetings or travel for the 2020 American Society of Clinical Oncology Genitourinary Cancers symposium from Bayer. AZ reports payment or honoraria for lectures, presentations, speaking, bureaus, manuscript writing, or educational events from Pfizer, Janssen, Astellas, and Sanofi, and received support for attending meetings or travel from Bayer and Merck Sharpe and Dohme. JSdB reports employment by the Institute of Cancer Research that has a commercial interest in abiraterone acetate and personal fees from Janssen, during the conduct of the study; and personal fees, speaker's fees, and travel support from AstraZeneca, Pfizer, GlaxoSmithKline, Taiho, Daiichi, Novartis, Genmab, Merck Serono, Merck, and Genentech/Roche, outside the submitted work. DPD reports personal fees and speaker's fees and travel support from Takeda, Amgen, Astellas, Sandoz, and Cadence Research; personal fees and non-financial support from Janssen; travel support from Clovis; and personal fees and non-financial support from International Society for the Study and Exchange of evidence from Clinical research And Medical experience, outside the submitted work; in addition, DPD has a patent (GB9305269–17-substituted steroids useful in cancer treatment) with royalties paid to Janssen Pharmaceutical. MDM reports personal fees from Sanofi, Bayer, Dendreon, BMS, and Janssen, outside the submitted work. REL reports support for the present manuscript with funding from the UK Medical Research Council, part of the UK Research and Innovation. DM reports grants or contracts from Health Education England made to their institution, University of Wolverhampton, payments from Routledge for royalties on a book, payments from Authors' licensing and collecting society for royalties on photocopies of the book and chapters, and unpaid role as chair of a patient advocacy group, Prostate Cancer Research. MRS reports grants and non-financial support from Sanofi-Aventis, Novartis, Pfizer, Janssen, and Astellas, during the conduct of the study; and personal fees from Eli-Lilly, outside the submitted work. MKBP reports grants and non-financial support from Janssen, during the conduct of the study; and grants and non-financial support from Astellas, Clovis Oncology, Novartis, Pfizer, and Sanofi, outside the submitted work. NDJ reports grants and personal fees from Sanofi and Novartis, during the conduct of the study; and grants and personal fees from Janssen, Astellas, and Bayer, outside the submitted work. All other authors declare no competing interests.
