# Supplementary material for: Abiraterone acetate and prednisolone with or without enzalutamide for high-risk non-metastatic prostate cancer: a meta-analysis of primary results from two randomised controlled phase 3 trials of the STAMPEDE platform protocol
Source: Lancet. 2022 Jan 29;399(10323):447–60. doi: 10.1016/S0140-6736(21)02437-5 (PMC8811484; doi:10.1016/S0140-6736(21)02437-5)
Supplement: Supplementary appendix [file mmc1.pdf]

THE LANCET

Supplementary appendix

This appendix formed part of the original submission and has been peer reviewed. We post it as supplied by the authors.

Supplement to: Attard G, Murphy L, Clarke N W, et al. Abiraterone acetate and prednisolone with or without enzalutamide for high-risk non-metastatic prostate cancer: a meta-analysis of primary results from two randomised controlled phase 3 trials of the STAMPEDE platform protocol. *Lancet* 2021; published online Dec 23. https://doi.org/10.1016/S0140-6736(21)02437-5.

Supplementary appendix

Supplement to: Attard G, Murphy L, Clark, NWC, et al. Abiraterone acetate and prednisolone with or without enzalutamide for high-risk nonmetastatic prostate cancer: a meta-analysis of primary results from two randomised controlled Phase 3 trials of the STAMPEDE platform protocol.

CONTENTS

Supplementary Tables
S1. Extended baseline characteristics not included in **Table 1** 3
S2. Reasons for permanently stopping combination therapy in the abiraterone trial 5
S3. Reasons for permanently stopping combination therapy in the abiraterone and enzalutamide trial 5
S4. Breakdown of metastasis-free survival events 6
S5. Sub-group analysis of treatment effect on overall survival for baseline randomisation stratification factors 6
S6. Reported toxicities 7

Supplementary Figures
S1. Contemporaneous and previously recruiting trials in the STAMPEDE platform protocol 10
S2. Time from starting to permanently stopping combination therapy 10
S3. Metastasis-free survival in each trial 11
S4. Overall survival in each trial 12
S5. Effect of treatment on prostate cancer-specific survival, by trial 13
S6. Effect of treatment on progression-free survival, by trial 13
S7. Failure-free survival 14
S8. Effect of treatment on failure-free survival, by trial 14

Author contributions 15

Statistical Analysis Plan 16

Protocol 58

List of STAMPEDE oversight committees, staff and collaborators 230

SUPPLEMENTARY TABLES

Table S1. Extended baseline characteristics (not included in **Table 1**).

Characteristic	Control group (abiraterone trial) N = 455		Control group (abiraterone + enzalutamide trial) N = 533		Combination therapy group (abiraterone trial) N = 459		Combination therapy group (abiraterone and enzalutamide trial) N = 527	
	n	%	n	%	n	%	n	%
**Pain from prostate cancer**								
Absent	441	97%	505	95%	428	95%	493	94%
Present	12	3%	26	5%	24	5%	32	6%
Missing	*2*	*n/a*	*2*	*n/a*	*7*	*n/a*	*2*	*n/a*
**Planned/current ADT**								
Orchidectomy	2	0%	0	0%	0	0%	0	0%
LHRH agonist/antagonist	448	98%	532	>99%	455	99%	524	99%
Bicalutamide	4	1%	0	0%	4	1%	3	1%
Dual androgen blockade	1	0%	1	0%	0	0%	0	0%
**Planned anti-androgen use**								
None	24	5%	13	2%	31	7%	8	2%
Short-term anti-androgen	430	95%	519	97%	428	93%	519	98%
Long-term anti-androgen	1	0%	1	0%	0	0%	0	0%
**Docetaxel planned as SOC***								
No			110	20%			108	20%
Yes			11	2%			12	2%
N/A (Pre-SOC change)	455	100%	412	77%	459	100%	407	77%
**Aspirin use**								
No	371	82%	442	83%	372	81%	448	85%
Yes	84	18%	91	17%	87	19%	79	15%
**NSAID use**								
No	426	94%	505	95%	429	93%	487	92%
Yes	29	6%	28	5%	30	7%	40	8%
**Short-term bisphosphonate use**								
No	455	100%	531	>99%	458	>99%	525	>99%
Yes	0	0%	2	<1%	1	<1%	2	<1%
**HT started before rand'n**								
No	30	7%	15	3%	29	6%	19	4%
Yes	425	93%	518	97%	430	94%	508	96%
**Smoker**								
No	400	89%	472	89%	393	87%	471	90%
Yes	50	11%	61	11%	61	13%	55	10%
Missing	5	n/a	0	n/a	5	n/a	1	n/a
**Diabetes**								
No	401	88%	458	86%	408	89%	469	89%

Characteristic	Control group (abiraterone trial) N = 455		Control group (abiraterone + enzalutamide trial) N = 533		Combination therapy group (abiraterone trial) N = 459		Combination therapy group (abiraterone and enzalutamide trial) N = 527	
	**n**	**%**	**n**	**%**	**n**	**%**	**n**	**%**
Yes, type 1	12	3%	20	4%	17	4%	12	2%
Yes, type 2	42	9%	55	10%	34	7%	46	9%
Missing	0	n/a	0	n/a	0	n/a	0	n/a
**Myocardial infarction**								
No	433	95%	500	94%	435	95%	505	96%
Yes, but still fit for trial	22	5%	32	6%	24	5%	22	4%
Missing	0	n/a	1	n/a	0	n/a	0	n/a
**Cerebrovascular disease**								
No	446	98%	514	97%	448	98%	501	95%
Yes, but still fit for trial	9	2%	18	3%	11	2%	26	5%
Missing	0	n/a	1	n/a	0	n/a	0	n/a
**Congestive heart failure**								
No	454	100%	529	99%	456	99%	524	99%
Yes, but still fit for trial	1	0%	3	1%	3	1%	3	1%
Missing	0	n/a	1	n/a	0	n/a	0	n/a
**Angina**								
No	438	96%	504	95%	442	96%	501	95%
Yes, but still fit for trial	17	4%	28	5%	17	4%	26	5%
Missing	0	n/a	1	n/a	0	n/a	0	n/a
**Hypertension**								
No	256	56%	295	56%	266	58%	302	57%
Yes, but still fit for trial	199	44%	237	44%	193	42%	225	43%
Missing	0	n/a	1	n/a	0	n/a	0	n/a

SOC, Standard of care; NSAID, nonsteroidal anti-inflammatory drug. *Docetaxel allowed as SOC after protocol amendment December 22, 2015.

Table S2. Reasons for permanently stopping abiraterone acetate and prednisolone in the abiraterone trial

**Reason**	**N**	**%**
**Treatment complete**	266	59%
**Excessive toxicity**	60	13%
**Treatment refusal**	14	3%
**Disease progression**	18	4%
**Death**	3	1%
**Other**	63	14%
**Patient choice**	5	1%
**Clinician decision**	3	1%
**Intercurrent Illness**	1	<1%
**Not stopped**	18	4%
**Total started treatment**	**451**	**100%**

Table S3. Reasons for permanently stopping abiraterone acetate and prednisolone or enzalutamide in the abiraterone and enzalutamide trial

**Reason for stopping abiraterone acetate and prednisolone** / **Reason for stopping enzalutamide**	**Treatment complete**	**Excessive toxicity**	**Treatment refusal**	**Disease progression**	**Death**	**Other, please specify***	**Patient choice**	**Clinician decision**	**Intercurrent Illness**	***Not reported as stopped***	**Never started**	**Total**	**%**
**Treatment complete**	222	10	0	0	0	1	0	1	0	0	1	**235**	45%
**Excessive toxicity**	33	116	2	0	1	3	0	0	0	6	0	**161**	31%
**Treatment refusal**	1	1	25	0	0	0	0	0	0	0	0	**27**	5%
**Disease progression**	0	0	0	8	0	0	0	0	0	0	0	**8**	2%
**Death**	0	1	0	0	0	0	0	0	0	0	0	**1**	0%
**Other**	2	4	0	1	0	38	0	0	0	0	0	**45**	9%
**Patient choice**	0	1	0	0	0	1	9	0	0	0	0	**11**	2%
**Clinician decision**	0	0	0	0	0	0	0	3	0	0	0	**3**	1%
**Intercurrent Illness**	0	0	0	1	0	0	0	0	1	0	0	**2**	0%
**Not stopped**	0	1	0	0	0	0	0	0	0	19	0	**20**	4%
**Never started**	0	0	0	0	0	0	0	0	0	0	14	**14**	3%
**Total**	**258**	**134**	**27**	**10**	**1**	**43**	**9**	**4**	**1**	**25**	**15**	**527**	
**%**	49%	25%	5%	2%	0%	8%	2%	1%	0%	5%	3%		

Table S4. Breakdown of metastasis-free survival events

	Control group (abiraterone trial)	Control group (abiraterone and enzalutamide trial)	Combination therapy (abiraterone trial)	Combination therapy (abiraterone + enzalutamide trial)
**MFS events**	**183**	**123**	**111**	**69**
event = death (% of events)	73 (40%)	44 (36%)	60 (54%)	33 (48%)
event = metastasis	110 (60%)	79 (64%)	51 (46%)	36 (52%)

MFS, metastasis-free survival

Table S5. Sub-group analysis of treatment effect on overall survival for baseline randomisation stratification factors

	**Control groups**<br><br>Number of Events/Number of patients	**Combination Therapy groups**<br><br>Number of Events/Number of patients	**HR**	**95% CI**	**Interaction p-value**
**N-stage at randomisation**					**0.094**
N0	105/598	76/599	0.70	0.52-0.97	
N+	130/389	71/385	0.53	0.39-0.70	
NX	*1/1*	*0/2*			
**Age**					**0.302**
< 70	128/576	81/575	0.58	0.44-0.77	
70+	108/412	66/411	0.60	0.44-0.82	
**WHO performance status**					**0.048**
0	194/810	106/799	0.53	0.42-0.67	
1-2	42/178	41/187	0.87	0.57-1.34	
**NSAID/aspirin use at baseline**					**0.008**
Neither	164/772	117/762	0.71	0.56-0.90	
Receiving NSAID or aspirin	72/216	30/224	0.36	0.23-0.55	
**Local radiotherapy planned**					**0.306**
No	62/145	36/145	0.50	0.33-0.76	
Yes	174/843	111/841	0.62	0.49-0.79	

SOC, standard of care; HR, hazard ration; NSAID, non-steroidal anti-inflammatory drug

Table S6. Reported toxicities not included in Table 2. Ordered by frequency of grade 3 event in the combination therapy group in the abiraterone and enzalutamide trial.

	**Control group in the abiraterone trial (n=455)**			**Control group in the abiraterone and enzalutamide trial (n=533)**			**Combination therapy in the abiraterone trial (n=451)**			**Combination therapy in the abiraterone and enzalutamide trial (n=513)**		
	**Grade 1–2**	**Grade 3**	**Grade 4**	**Grade 1–2**	**Grade 3**	**Grade 4**	**Grade 1–2**	**Grade 3**	**Grade 4**	**Grade 1–2**	**Grade 3**	**Grade 4**
Hot flashes	388 (85%)	16 (4%)	n/a	448 (84%)	32 (6%)	n/a	386 (86%)	18 (4%)	n/a	443 (86%)	39 (8%)	n/a
Musculoskeletal and connective tissue - other	126 (28%)	5 (1%)	0 (0%)	116 (22%)	9 (2%)	0 (0%)	126 (28%)	9 (2%)	0 (0%)	127 (25%)	14 (3%)	1 (<1%)
Renal and urinary - other	97 (21%)	4 (1%)	2 (<1%)	125 (23%)	12 (2%)	1 (<1%)	85 (19%)	5 (1%)	0 (0%)	109 (21%)	13 (3%)	0 (0%)
Respiratory, thoracic and mediastinal - other	31 (7%)	5 (1%)	1 (<1%)	25 (5%)	3 (1%)	1 (<1%)	49 (11%)	14 (3%)	0 (0%)	30 (6%)	7 (1%)	2 (<1%)
Investigations - other	107 (24%)	2 (<1%)	0 (0%)	118 (22%)	7 (1%)	0 (0%)	137 (30%)	7 (2%)	0 (0%)	151 (29%)	8 (2%)	2 (<1%)
Nervous system - other	32 (7%)	4 (1%)	0 (0%)	33 (6%)	10 (2%)	0 (0%)	40 (9%)	5 (1%)	1 (<1%)	59 (12%)	5 (1%)	1 (<1%)
General and admin - other	23 (5%)	4 (1%)	3 (1%)	22 (4%)	6 (1%)	0 (0%)	37 (8%)	5 (1%)	4 (1%)	34 (7%)	7 (1%)	2 (<1%)
GI - other	75 (16%)	4 (1%)	1 (<1%)	78 (15%)	5 (1%)	4 (1%)	93 (21%)	8 (2%)	1 (<1%)	106 (21%)	5 (1%)	1 (<1%)
Breathlessness	86 (19%)	2 (<1%)	0 (0%)	121 (23%)	5 (1%)	0 (0%)	106 (24%)	8 (2%)	0 (0%)	143 (28%)	5 (1%)	0 (0%)
Diarrhoea	163 (36%)	5 (1%)	0 (0%)	230 (43%)	3 (1%)	0 (0%)	162 (36%)	5 (1%)	0 (0%)	231 (45%)	8 (2%)	0 (0%)
Urinary tract infection	35 (8%)	4 (1%)	0 (0%)	36 (7%)	6 (1%)	0 (0%)	38 (8%)	5 (1%)	2 (<1%)	44 (9%)	6 (1%)	2 (<1%)
Abnormal hepatic function	20 (4%)	0 (0%)	1 (<1%)	26 (5%)	1 (<1%)	0 (0%)	44 (10%)	5 (1%)	0 (0%)	28 (5%)	4 (1%)	0 (0%)
Eye disorders - other	19 (4%)	3 (1%)	0 (0%)	16 (3%)	3 (1%)	0 (0%)	34 (8%)	5 (1%)	0 (0%)	35 (7%)	3 (1%)	0 (0%)
Generalised pain	113 (25%)	3 (1%)	0 (0%)	93 (17%)	5 (1%)	0 (0%)	115 (25%)	4 (1%)	0 (0%)	97 (19%)	2 (<1%)	0 (0%)
Psychiatric - other	100 (22%)	2 (<1%)	0 (0%)	84 (16%)	3 (1%)	0 (0%)	103 (23%)	4 (1%)	0 (0%)	109 (21%)	5 (1%)	0 (0%)
Skin - other	81 (18%)	3 (1%)	0 (0%)	66 (12%)	0 (0%)	0 (0%)	92 (20%)	3 (1%)	0 (0%)	98 (19%)	8 (2%)	0 (0%)
Haematuria	26 (6%)	2 (<1%)	0 (0%)	34 (6%)	5 (1%)	0 (0%)	33 (7%)	1 (<1%)	2 (<1%)	38 (7%)	3 (1%)	0 (0%)
Abdominal pain	64 (14%)	2 (<1%)	0 (0%)	88 (17%)	3 (1%)	0 (0%)	80 (18%)	2 (<1%)	0 (0%)	106 (21%)	3 (1%)	0 (0%)

Fracture	n/a	n/a	n/a	4 (1%)	4 (1%)	0 (0%)	n/a	n/a	n/a	12 (2%)	4 (1%)	0 (0%)
Back pain	n/a	n/a	n/a	75 (14%)	3 (1%)	0 (0%)	n/a	n/a	n/a	90 (18%)	5 (1%)	0 (0%)
Vomiting	13 (3%)	1 (<1%)	0 (0%)	29 (5%)	2 (<1%)	0 (0%)	33 (7%)	1 (<1%)	0 (0%)	47 (9%)	3 (1%)	0 (0%)
Arthralgia	101 (22%)	0 (0%)	n/a	151 (28%)	3 (1%)	n/a	90 (20%)	5 (1%)	n/a	171 (33%)	2 (<1%)	n/a
Myalgia	68 (15%)	0 (0%)	n/a	102 (19%)	2 (<1%)	n/a	50 (11%)	3 (1%)	n/a	125 (24%)	2 (<1%)	n/a
Acute coronary syndrome	0 (0%)	1 (<1%)	2 (<1%)	2 (<1%)	3 (1%)	4 (1%)	1 (<1%)	1 (<1%)	4 (1%)	1 (<1%)	2 (<1%)	2 (<1%)
Cardiac dysrhythmia	14 (3%)	2 (<1%)	0 (0%)	15 (3%)	0 (0%)	0 (0%)	15 (3%)	3 (1%)	0 (0%)	21 (4%)	2 (<1%)	0 (0%)
Cataract	n/a	n/a	n/a	4 (1%)	5 (1%)	0 (0%)	n/a	n/a	n/a	8 (2%)	2 (<1%)	0 (0%)
Upper respiratory infection	40 (9%)	1 (<1%)	0 (0%)	49 (9%)	1 (<1%)	0 (0%)	55 (12%)	3 (1%)	0 (0%)	61 (12%)	2 (<1%)	0 (0%)
Bone pain	87 (19%)	1 (<1%)	n/a	82 (15%)	0 (0%)	n/a	65 (14%)	3 (1%)	n/a	100 (19%)	3 (1%)	n/a
Acute kidney injury	1 (<1%)	2 (<1%)	0 (0%)	15 (3%)	1 (<1%)	0 (0%)	2 (<1%)	1 (<1%)	1 (<1%)	13 (3%)	3 (1%)	1 (<1%)
Urinary tract obstruction	n/a	n/a	n/a	14 (3%)	5 (1%)	1 (<1%)	n/a	n/a	n/a	13 (3%)	1 (<1%)	0 (0%)
Fever	12 (3%)	2 (<1%)	0 (0%)	16 (3%)	0 (0%)	0 (0%)	13 (3%)	1 (<1%)	0 (0%)	24 (5%)	2 (<1%)	0 (0%)
Lower respiratory infection	n/a	n/a	n/a	9 (2%)	1 (<1%)	0 (0%)	n/a	n/a	n/a	7 (1%)	4 (1%)	2 (<1%)
Neutrophil count decreased	15 (3%)	0 (0%)	0 (0%)	37 (7%)	1 (<1%)	0 (0%)	21 (5%)	1 (<1%)	0 (0%)	49 (10%)	3 (1%)	1 (<1%)
Blurred vision	18 (4%)	0 (0%)	0 (0%)	26 (5%)	1 (<1%)	0 (0%)	20 (4%)	2 (<1%)	0 (0%)	45 (9%)	1 (<1%)	0 (0%)
Lower GI haemorrhage	n/a	n/a	n/a	30 (6%)	2 (<1%)	0 (0%)	n/a	n/a	n/a	34 (7%)	2 (<1%)	0 (0%)
GI haemorrhage	42 (9%)	3 (1%)	1 (<1%)	24 (5%)	0 (0%)	0 (0%)	50 (11%)	0 (0%)	0 (0%)	25 (5%)	1 (<1%)	0 (0%)
Fall	n/a	n/a	n/a	19 (4%)	1 (<1%)	0 (0%)	n/a	n/a	n/a	35 (7%)	3 (1%)	0 (0%)
Blood bilirubin increased	0 (0%)	0 (0%)	0 (0%)	5 (1%)	1 (<1%)	1 (<1%)	1 (<1%)	1 (<1%)	0 (0%)	18 (4%)	2 (<1%)	0 (0%)
Glucose intolerance	22 (5%)	0 (0%)	0 (0%)	38 (7%)	1 (<1%)	1 (<1%)	31 (7%)	2 (<1%)	0 (0%)	45 (9%)	1 (<1%)	1 (<1%)
Gynecomastia	68 (15%)	2 (<1%)	0 (0%)	141 (26%)	1 (<1%)	0 (0%)	59 (13%)	0 (0%)	0 (0%)	139 (27%)	1 (<1%)	0 (0%)
Rash maculo-papular	44 (10%)	1 (<1%)	0 (0%)	59 (11%)	0 (0%)	0 (0%)	59 (13%)	0 (0%)	0 (0%)	64 (12%)	3 (1%)	0 (0%)
Hypotension	11 (2%)	0 (0%)	0 (0%)	14 (3%)	1 (<1%)	0 (0%)	18 (4%)	1 (<1%)	0 (0%)	24 (5%)	2 (<1%)	0 (0%)
Febrile neutropenia	0 (0%)	0 (0%)	0 (0%)	0 (0%)	2 (<1%)	1 (<1%)	0 (0%)	0 (0%)	0 (0%)	0 (0%)	1 (<1%)	2 (<1%)

Chronic kidney disease	38 (8%)	2 (<1%)	0 (0%)	16 (3%)	4 (1%)	0 (0%)	42 (9%)	1 (<1%)	0 (0%)	36 (7%)	0 (0%)	1 (<1%)
Flatulence	77 (17%)	0 (0%)	0 (0%)	130 (24%)	0 (0%)	0 (0%)	70 (16%)	0 (0%)	0 (0%)	152 (30%)	0 (0%)	0 (0%)
Urinary urgency	n/a	n/a	n/a	144 (27%)	0 (0%)	0 (0%)	n/a	n/a	n/a	166 (32%)	0 (0%)	0 (0%)
Hepatobiliary - other	8 (2%)	0 (0%)	0 (0%)	4 (1%)	0 (0%)	0 (0%)	22 (5%)	3 (1%)	0 (0%)	9 (2%)	0 (0%)	0 (0%)
Localised (o)dema	70 (15%)	0 (0%)	0 (0%)	118 (22%)	1 (<1%)	0 (0%)	85 (19%)	1 (<1%)	1 (<1%)	98 (19%)	0 (0%)	0 (0%)
Flu-like symptoms	21 (5%)	0 (0%)	0 (0%)	23 (4%)	1 (<1%)	0 (0%)	38 (8%)	0 (0%)	0 (0%)	43 (8%)	1 (<1%)	0 (0%)
Allergic reaction	12 (3%)	0 (0%)	0 (0%)	15 (3%)	0 (0%)	0 (0%)	17 (4%)	0 (0%)	0 (0%)	23 (4%)	2 (<1%)	0 (0%)
Alkaline phosphatase increased	n/a	n/a	n/a	29 (5%)	1 (<1%)	0 (0%)	n/a	n/a	n/a	23 (4%)	1 (<1%)	0 (0%)
Weight gain	n/a	n/a	n/a	80 (15%)	2 (<1%)	0 (0%)	n/a	n/a	n/a	70 (14%)	0 (0%)	0 (0%)
Metabolism and nutrition - other	53 (12%)	0 (0%)	0 (0%)	28 (5%)	1 (<1%)	0 (0%)	42 (9%)	0 (0%)	0 (0%)	33 (6%)	1 (<1%)	0 (0%)
Neoplasms	n/a	n/a	n/a	0 (0%)	1 (<1%)	0 (0%)	n/a	n/a	n/a	0 (0%)	1 (<1%)	0 (0%)
Heart failure	n/a	n/a	n/a	0 (0%)	0 (0%)	0 (0%)	n/a	n/a	n/a	5 (1%)	1 (<1%)	0 (0%)
Upper GI haemorrhage	n/a	n/a	n/a	2 (<1%)	1 (<1%)	0 (0%)	n/a	n/a	n/a	5 (1%)	0 (0%)	0 (0%)
Infections - other	0 (0%)	0 (0%)	0 (0%)	0 (0%)	0 (0%)	0 (0%)	0 (0%)	0 (0%)	0 (0%)	0 (0%)	1 (<1%)	0 (0%)
Platelet count decreased	17 (4%)	0 (0%)	0 (0%)	22 (4%)	0 (0%)	0 (0%)	23 (5%)	1 (<1%)	0 (0%)	34 (7%)	0 (0%)	0 (0%)
Hypoalbuminaemia	n/a	n/a	n/a	6 (1%)	1 (<1%)	0 (0%)	n/a	n/a	n/a	19 (4%)	0 (0%)	0 (0%)
Osteoporosis	n/a	n/a	n/a	5 (1%)	0 (0%)	0 (0%)	n/a	n/a	n/a	16 (3%)	1 (<1%)	0 (0%)
Seizure	1 (<1%)	0 (0%)	0 (0%)	1 (<1%)	0 (0%)	0 (0%)	1 (<1%)	0 (0%)	1 (<1%)	0 (0%)	1 (<1%)	0 (0%)
Stroke	n/a	n/a	n/a	0 (0%)	1 (<1%)	0 (0%)	n/a	n/a	n/a	3 (1%)	0 (0%)	0 (0%)
Thromboembolic event	n/a	n/a	n/a	1 (<1%)	0 (0%)	0 (0%)	n/a	n/a	n/a	2 (<1%)	1 (<1%)	1 (<1%)
Urinary frequency	321 (71%)	n/a	n/a	417 (78%)	n/a	n/a	315 (70%)	n/a	n/a	407 (79%)	n/a	n/a

ALT, alanine aminotransferase; AST, aspartate aminotransferase; GI, gastro-intestinal; N/a, not applicable/not measured

SUPPLEMENTARY FIGURES

Fig S1. Contemporaneous and previously recruiting trials in STAMPEDE platform protocol.

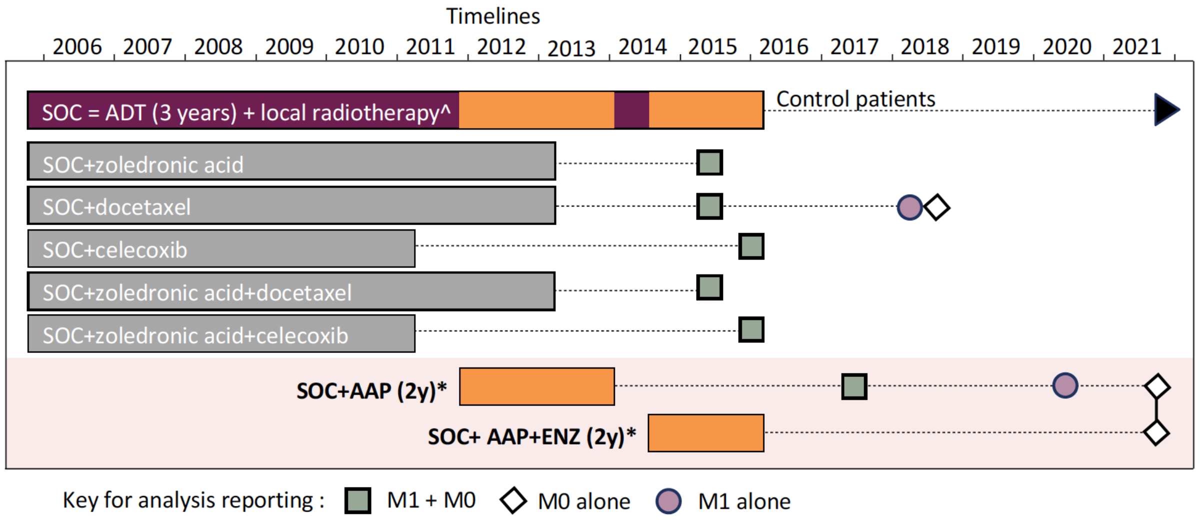

Period of accrual denoted by solid bars. Analysis timelines denoted by dotted lines and analysis reporting by symbols in key. SOC, standard of care; ADT, androgen deprivation therapy; AAP, abiraterone acetate and prednisolone; ENZ, enzalutamide; 2y, 2 years; M0, nonmetastatic; M1 metastatic; ^when indicated; *meta-analysed trials

Fig S2. Time from starting to permanently stopping combination therapy.

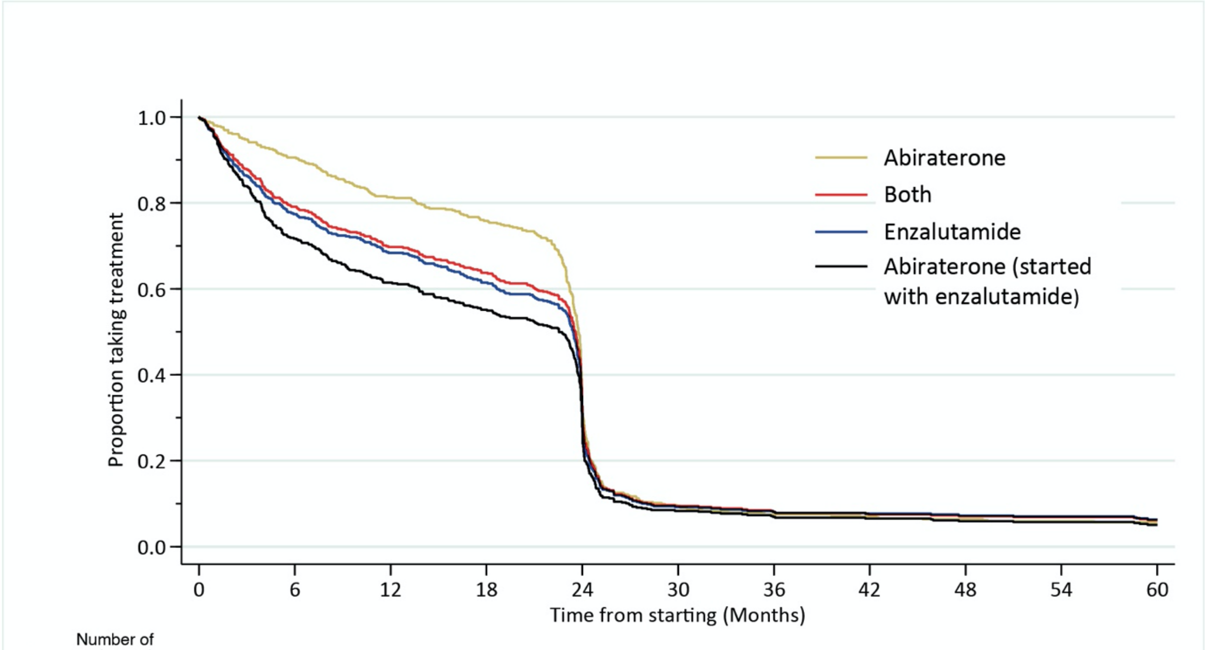

Number of patients (events)																					
Abiraterone	459	(43)	413	(42)	369	(25)	344	(199)	145	(102)	43	(9)	34	(2)	32	(3)	28	(2)	25	(1)	24
Both	527	(109)	412	(49)	363	(31)	332	(161)	169	(120)	48	(7)	41	(3)	38	(2)	35	(1)	31	(3)	18
Enzalutamide	527	(118)	403	(47)	356	(36)	320	(158)	161	(113)	47	(6)	41	(2)	38	(2)	35	(1)	31	(3)	18
Abiraterone (started with enzalutamide)	527	(148)	373	(53)	320	(33)	287	(141)	144	(101)	42	(7)	35	(2)	33	(3)	30	(1)	27	(3)	16

All patients are reported. Those not reporting stopping are censored at time of last contact.

Fig S3. Effect on metastasis-free survival in each trial individually.

A. Metastasis-free survival, SOC vs SOC with abiraterone

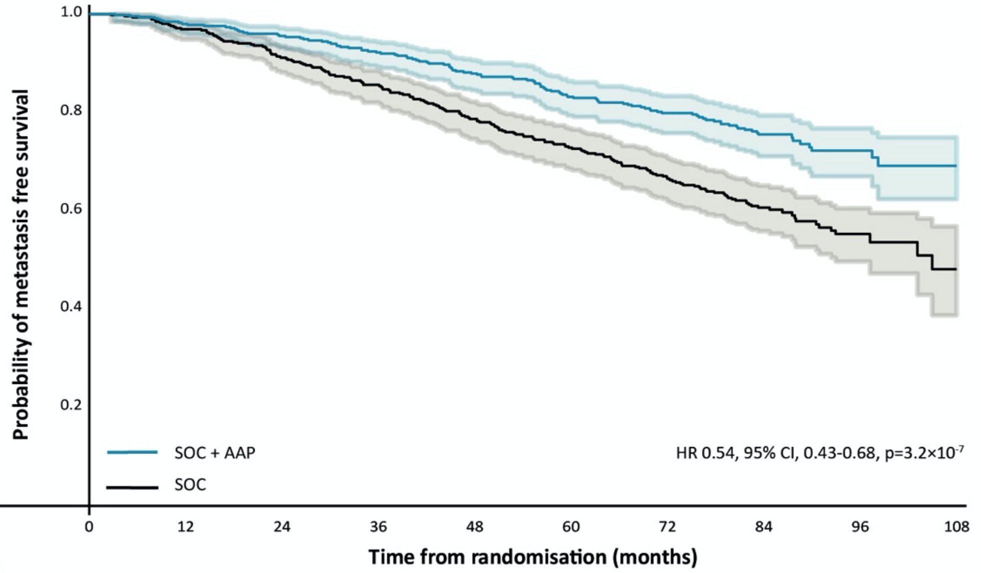

	0	12	24	36	48	60	72	84	96	108
**SOC**										
At-risk	455	438	411	385	351	318	266	172	53	9
Censored	0	3	4	5	8	15	40	112	222	263
Event	0	14	40	65	96	122	149	171	180	183
**SOC+AAP**										
At-risk	459	441	426	411	391	362	312	198	71	14
Censored	0	9	13	13	14	22	58	157	279	334
Event	0	9	20	35	54	75	89	104	109	111

B. Metastasis-free survival, SOC vs SOC with abiraterone and enzalutamide

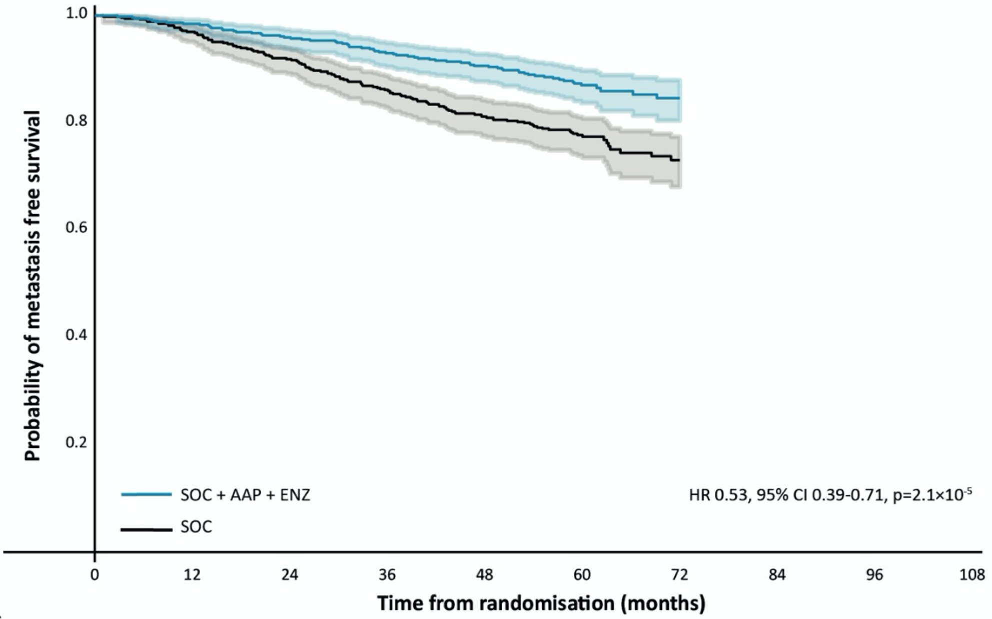

	0	12	24	36	48	60	72
**SOC**							
At-risk	533	512	483	451	416	232	63
Censored	0	5	7	9	18	186	347
Event	0	16	43	73	99	115	123
**SOC+AAP+ENZ**							
At-risk	527	507	491	473	448	260	57
Censored	0	12	15	18	31	203	402
Event	0	8	21	36	48	64	68

Kaplan-Meier estimates; 95% confidence interval represented by lighter shade. Sensitivity analysis excluding 22 patients (1%) who were classified as protocol deviations due to excursions from eligibility criteria was also performed: hazard ratios (95% confidence intervals) for abiraterone and abiraterone with enzalutamide trials respectively, 0.54 (0.43, 0.68) and 0.53 (0.39, 0.71). SOC, standard of care; AAP, abiraterone; ENZ, enzalutamide.

Fig S4. Effect on overall survival in each trial individually.

A. Overall survival, SOC vs SOC with abiraterone

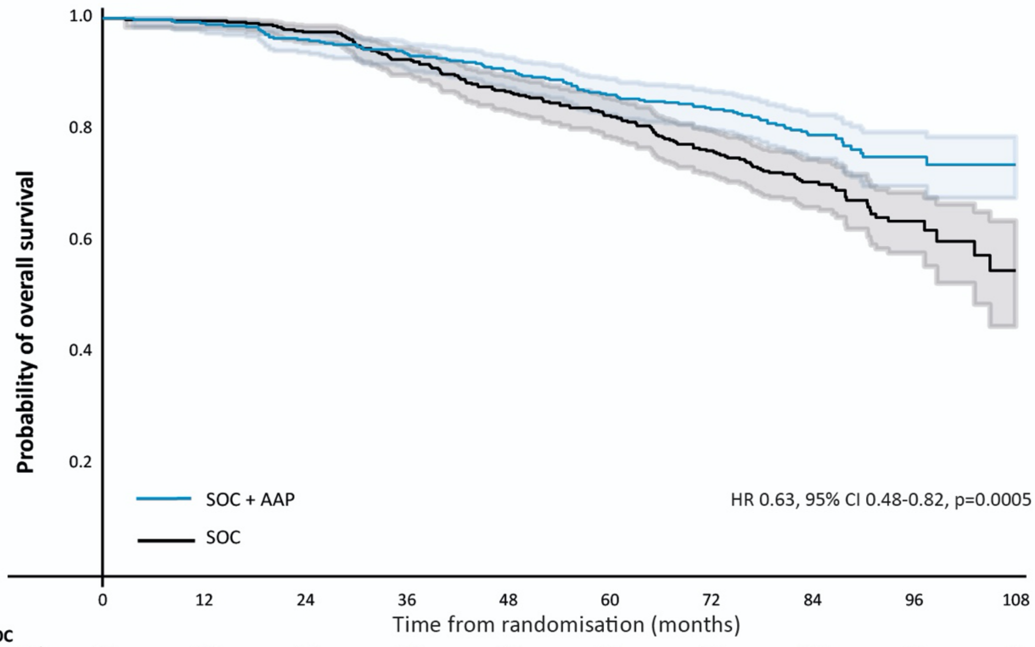

	0	12	24	36	48	60	72	84	96	108
**SOC**										
At-risk	455	450	440	417	388	361	302	200	63	10
Censored	0	3	4	5	8	16	48	129	254	303
Event	0	2	11	33	59	78	105	126	138	142
**SOC+AAP**										
At-risk	459	446	429	418	403	376	328	205	74	16
Censored	0	9	13	13	14	22	59	166	291	348
Event	0	4	17	28	42	61	72	88	94	95

B. Overall survival, SOC vs SOC with abiraterone and enzalutamide

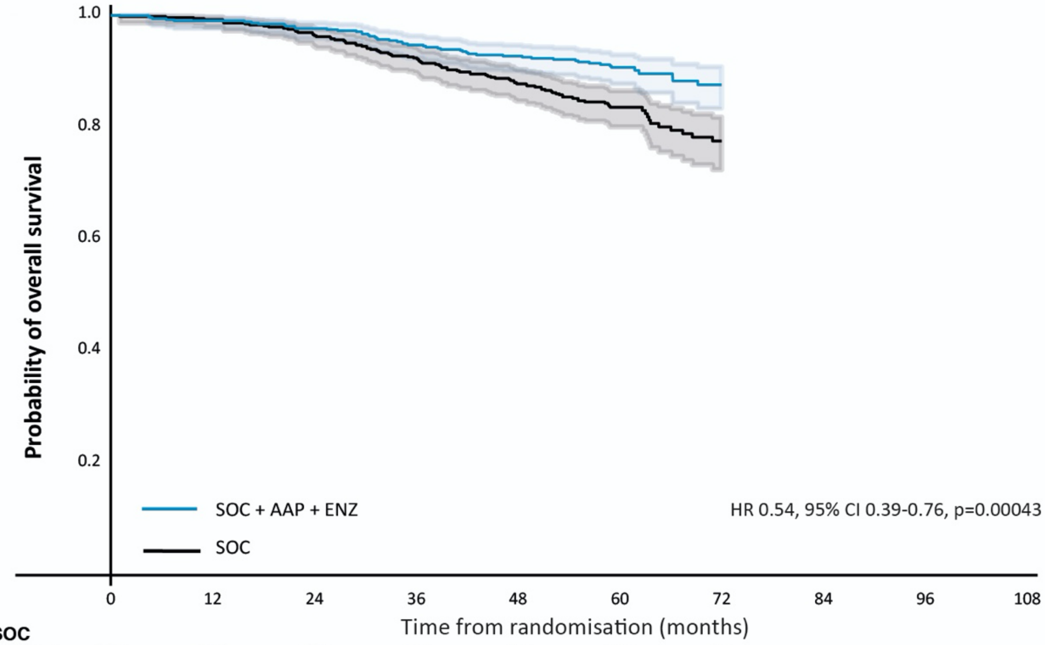

	0	12	24	36	48	60	72
**SOC**							
At-risk	533	524	507	484	449	249	66
Censored	0	5	7	9	20	200	373
Event	0	4	19	40	64	84	94
**SOC+AAP+ENZ**							
At-risk	527	510	499	481	458	269	58
Censored	0	12	16	19	32	212	418
Event	0	5	12	27	37	46	51

Kaplan-Meier estimates; 95% confidence interval represented by lighter shade. SOC, standard of care; AAP, abiraterone; ENZ, enzalutamide.

Fig S5. Effect of treatment on prostate cancer-specific survival by trial

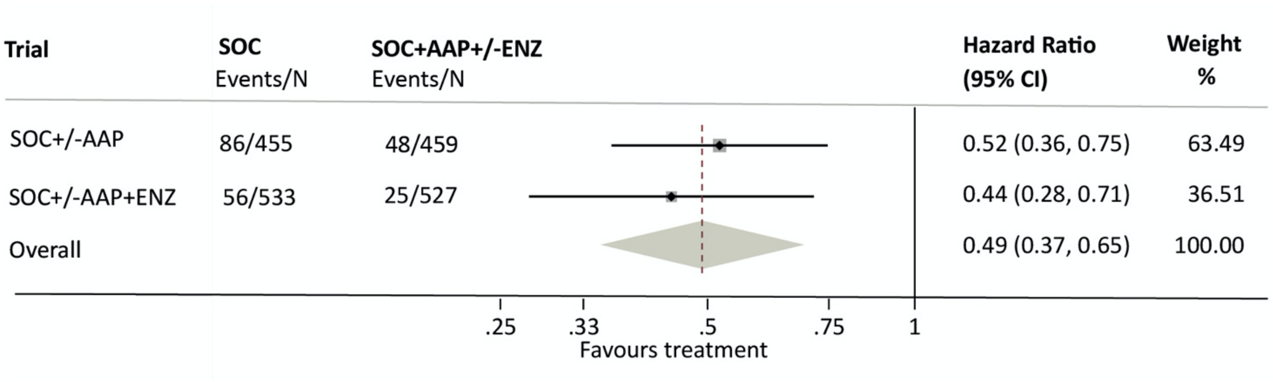

The dashed line is the point estimate for the IPD meta-analysis. The p value for the interaction between comparisons was 0.610 and the p value for I^2 was 0.598. SOC, standard of care; AAP, abiraterone; ENZ, enzalutamide.

Fig S6. Effect of treatment on progression-free survival by trial

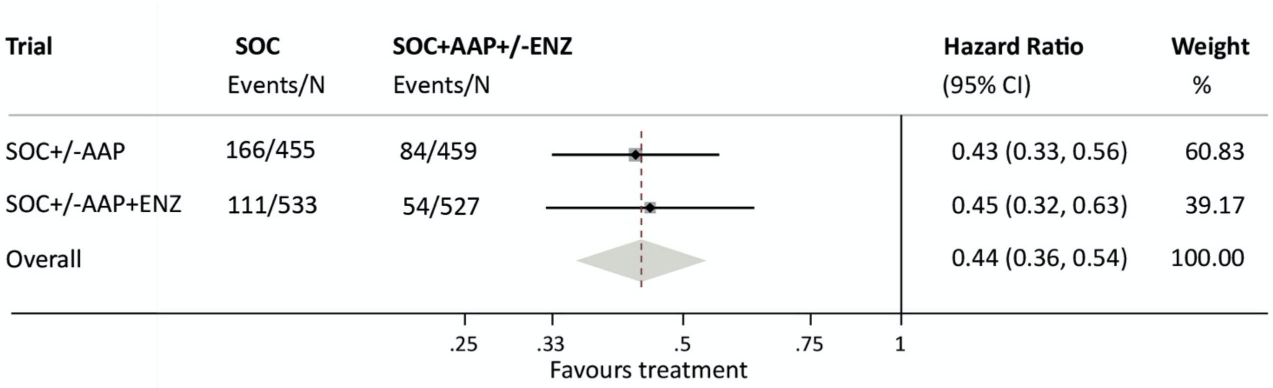

The dashed line is the point estimate for the IPD meta-analysis. The p value for the interaction between comparisons was 0.842 and the p value for I^2 was 0.831. SOC, standard of care; AAP, abiraterone; ENZ, enzalutamide.

Fig S7. Failure-free survival. Kaplan-Meier estimates of all patients in individual patient data (IPD) meta-analysis; 95% confidence interval represented by lighter shade. SOC, standard of care; AAP, abiraterone acetate and prednisolone; ENZ, enzalutamide.

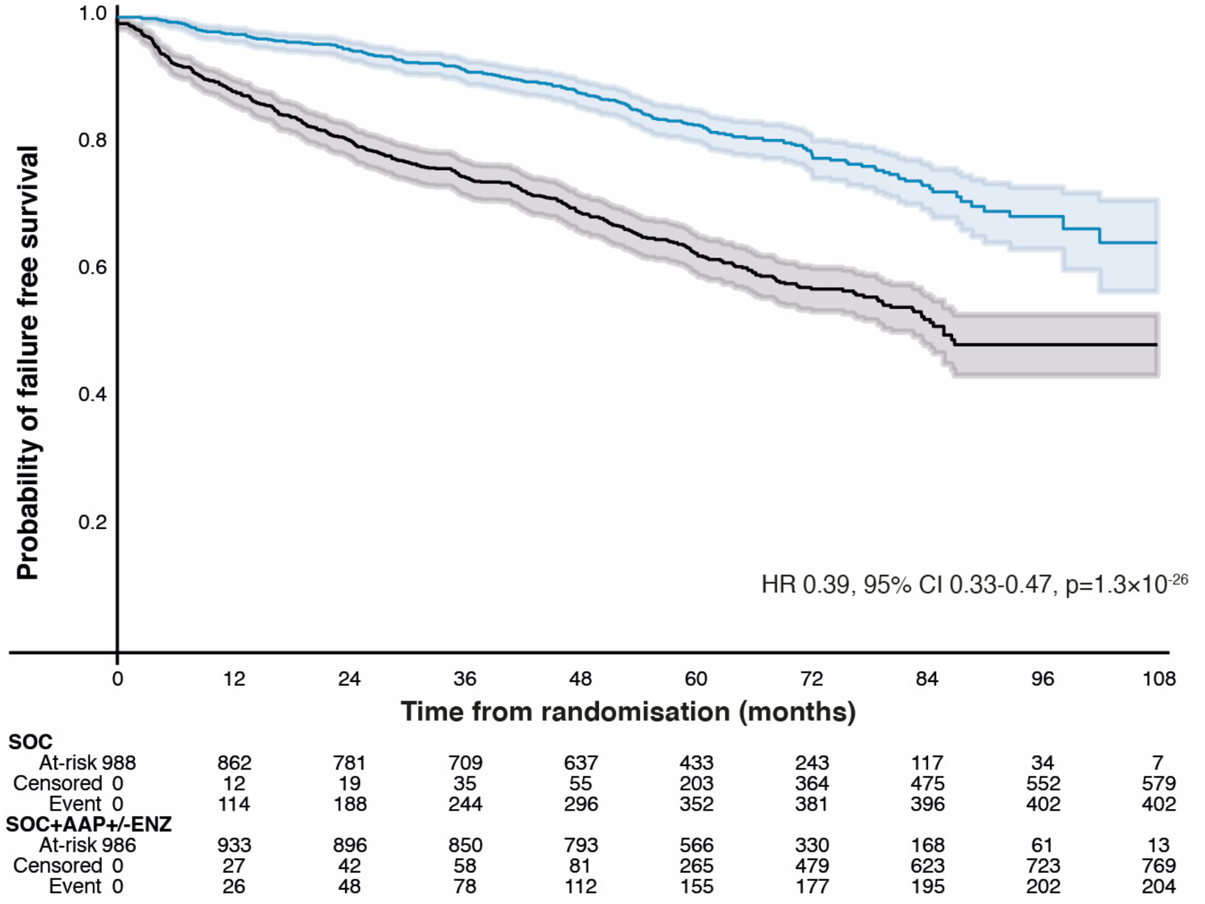

Fig S8. Effect of treatment on failure-free survival by trial

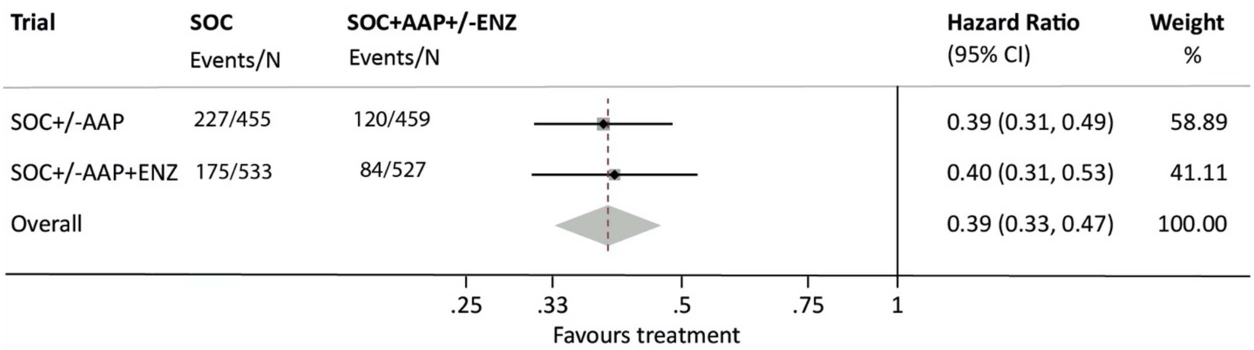

The dashed line is the point estimate for the IPD meta-analysis. The p value for the interaction between comparisons was 0.849 and the p value for I^2 was 0.839. SOC, standard of care; AAP, abiraterone; ENZ, enzalutamide.

Author contributions

Name	Conceived the original MAMS idea	Designed comparisons (both/either)	Was a grant holder	Enrolled study subjects	Collected or collated data	Performed or oversaw statistical analyses	Interpreted data	Wrote critical sections of the report	Reviewed and agreed the report
Attard, Gerhardt		x	x	x	x		x	x	x
Murphy, Laura R					x	x	x	x	x
Clarke, Noel W		x	x	x	x		x	x	x
Cross, William				x	x		x		x
Jones, Rob J				x	x		x		x
Parker, Chris C				x	x		x		x
Gillessen, Silke				x	x		x		x
Cook, Adrian					x	x	x	x	x
Brawley, Chris					x	x	x		x
Amos, Claire		x			x		x		x
Atako, Nafisah					x		x		x
Pugh, Cheryl					x		x		x
Buckner, Michelle					x		x		x
Chowdhury, Simon				x	x		x		x
Malik, Zafar				x	x		x		x
Russell, J Martin				x	x		x		x
Gilson, Claire					x		x		x
Rush, Hannah					x		x		x
Bowen, Jo				x	x				x
Lydon, Anna				x	x				x
Pedley, Ian				x	x				x
Sullivan, Joe M				x	x				x
Birtle, Alison				x	x				x
Gale, Joanna				x	x				x
Srihari, Narayanan				x	x				x
Thomas, Carys				x	x				x
Tanguay, Jacob				x	x				x
Wagstaff, John				x	x				x
Das, Prantik				x	x				x
Gray, Emma				x	x				x
Alzoueb, Mymoona				x	x				x
Parikh, Omi				x	x				x
Robinson, Angus				x	x				x
Syndikus, Isabel				x	x				
Wylie, James				x					
Zarkar, Anjali				x					
Thalmann, George				x					x
de Bono, Johann			x		x				x
Dearnaley, David				x	x		x		x
Mason, Malcolm				x	x				x
Gilbert, Duncan				x	x		x		x
Langley, Ruth				x	x		x		x
Millman, Robin							x		x
Matheson, David							x		x
Sydes, Matthew R		x	x		x	x	x	x	x
Brown, Louise					x	x	x	x	x
Parmar, Mahesh K B	x	x	x		x	x	x	x	x
James, Nicholas David		x	x	x	x		x	x	x

STAMPEDE

Systemic Therapy in Advancing or Metastatic Prostate Cancer: Evaluation of Drug Efficacy
A multi-arm multi-stage randomised controlled trial

MRC PR08

ISRCTN number:	ISRCTN78818544
EUDRACT number:	2004-000193-31
CTA number:	00316/0026/001-0001
NCT number:	NCT00268476

STATISTICAL ANALYSIS PLAN
"Abiraterone and enzalutamide" comparison and combined primary analysis of M0 patients in the A-G and A-J comparisons

MRC Clinical Trials Unit at UCL

Tel: +44 (0)20 7670 4798
Fax: +44 (0)20 7670 4818
Email: mrcctu.stampede@ucl.ac.uk

Version date: 30-July-2021
Will replace version 2.0 (31-Jan-2017)

Contacts
Laura Murphy
Adrian Cook
Louise Brown

This template and all preceding versions will be stored in the Statistical Analysis Master File for this trial held in S:\MRCCTU_Stampede_Stats\SAP

CONTENTS

1 ABBREVIATIONS 4

2 BACKGROUND AND DESIGN 6
2.1 TRIAL SUMMARY 6
2.2 COMPARISONS 7
2.3 ELIGIBILITY CRITERIA 9
2.4 METHOD OF RANDOMISATION 10
2.5 ALLOCATION RATIO 10

3 DATA COLLECTION AND PROCESSING 11
3.1 CASE REPORT FORMS AND VARIABLES 11
3.2 DATA COMPLETION SCHEDULE FOR CONSENT, BASELINE, TREATMENT & FOLLOW-UP 11
3.3 DATA COMPLETION SCHEDULE FOR OTHER 'NON-SCHEDULED' CRFS 12
3.4 MANAGEMENT OF DATASETS 12
3.4.1 Data Cleaning 12
3.4.2 Data verification 13
3.4.3 Database Lock 14
3.5 DATA CODING 14
3.5.1 Free-text variables 14
3.6 PRE-PLANNED DATA CHECKS 14
3.7 DATA RETURNS 15
3.8 DATA MATURITY 15
3.9 MISSING DATA 15

4 OUTCOME MEASURES 17

5 SAMPLE SIZE CALCULATIONS 21
5.1 SAMPLE SIZE 21
5.2 SAMPLE SIZE FOR INTERMEDIATE ANALYSES 21
5.3 FURTHER DETAILS IN SAMPLE SIZE CALCULATIONS AND DESIGN 22

6 STATISTICAL ANALYSIS 23
6.1 TIME PERIODS 23
6.2 POPULATIONS FOR ANALYSIS 24
6.3 RECRUITMENT AND PATIENT FLOW 25
6.3.1 Recruitment 25
6.3.2 patient flow through the trial 25
6.4 BASELINE CHARACTERISTICS 25
6.5 STANDARD OF CARE TREATMENT 26
6.6 TRIAL TREATMENT 27
6.7 SURVIVAL OUTCOME MEASURES 28
6.7.1 primary outcome measure 28
6.7.2 secondary outcome measures 28
6.7.3 statistical analysis methods 28
6.7.4 Proportional and non-proportional hazards 29
6.7.5 Competing Risks 30
6.7.7 patients with no data post randomisation 30
6.7.8 withdrawn patients with outcome data 30
6.7.9 sensitivity analysis to assess impact of covid-19 pandemic 30

6.8 PRE-SPECIFIED SUBGROUP ANALYSES 30
6.8.1 Stratification Factors used for minimisation 31
6.8.2 OTHER PRE-SPECIFIED SUBGROUP ANALYSIS 31
6.8.2.1 Metastatic volume in M1 patients 31
6.9 PSA RESPONSE 31
6.10 TOXICITY AND SAFETY ANALYSES 31
6.11 QUALITY-OF-LIFE ANALYSIS 34
6.12 HEALTH ECONOMICS 34

7 APPENDIX 1 – STATISTICAL ANALYSIS PLAN FOR COMBINED ANALYSIS OF M0 PATIENTS IN THE A-G AND A-J COMPARISONS 35
7.1 RECRUITMENT AND PROGRESS FOR A-G AND A-J COMPARISONS 35
7.2 NEW KNOWLEDGE WITH IMPLICATIONS FOR THE FINAL ANALYSIS OF THE A-J COMPARISON 35
7.3 PROPOSED OUTCOME MEASURES FOR M0 PATIENTS 36
7.4 TIMELINES FOR REPORTING M0 RESULTS 37
7.5 REFERENCES 38
7.6 ADDENDUM 1 - NUMBERS OF EVENTS IN CONTROL ARMS BY JULY 2019 39
7.7 ADDENDUM 2 - NSTAGE CALCULATION FOR TARGET CONTROL ARM EVENT NUMBER, BASED ON OBSERVED MFS IN COMBINED ANALYSIS OF A-G AND A-J FOR M0 PATIENTS 40
7.8 ADDENDUM 3 - ESTIMATION OF CONTROL ARM EVENT ACCRUAL FOR M0 PATIENTS, ASSUMING LINEAR INCREASE BASED ON OBSERVED QUARTERLY ACCRUAL IN 2018 41

8 SIGNATURES OF APPROVAL 42

1 ABBREVIATIONS

Abbreviation	Expansion
**Abi**	Abiraterone
**ADT**	Androgen-deprivation therapy
**AMP**	Adenosine monophosphate
**AS**	Activity Stage
**BMD**	Bone mineral density
**CCI**	Comparison Chief Investigator
**Cel**	Celecoxib
**CHF**	Congestive heart failure
**CI**	Chief Investigator
**CI**	Confidence interval
**CRF**	Case Report Form
**CTCAE**	Common Toxicity Criteria for Adverse Events
**CTU**	Clinical Trials Unit
**CV**	Cerebrovascular
**DAB**	Dual Androgen Blockade
**DMP**	Data Management Plan
**Doc**	Docetaxel
**DAB**	Dual Androgen Blockade (previously Maximum Androgen Blockade [MAB])
**Enza**	Enzalutamide
**ES**	Efficacy Stage
**FFS**	Failure-free survival
**FPM**	Flexible parametric models
**HE**	Health Economics
**HEAP**	Health Economics Analysis Plan
**HR**	Hazard ratio
**HT**	Hormone therapy
**IDMC**	Independent Data Monitoring Committee
**ITT**	Intention-to-treat
**KM**	Kaplan-Meier
**LHRH**	Luteinising hormone-releasing hormone
**LOB**	Lack-of-benefit
**M0**	Non-metastatic
**M1**	Metastatic
**MACE**	Major adverse cardiac event
**MAMS**	Multi-arm multi-stage
**MCAR**	Missing completely at random
**MI**	Myocardial infarction
**MPFS**	Metastatic progression-free survival
**MRC**	Medical Research Council
**N+**	Lymph node-positive
**N0**	Lymph node-negative
**NX**	Lymph node stage unknown
**NSAID**	Non-steroidal anti-inflammatory drug
**ONS**	Office of National Statistics
**OS**	Overall survival
**PCa**	Prostate cancer
**PH**	Proportional hazards
**PHE**	Public Health England
**PSA**	Prostate specific antigen
**q6wk**	Every 6 weeks
**q12wk**	Every 12 weeks
**q6m**	Every 6 months

Abbreviation	Expansion
**q12m**	Every 12 months
**QL**	Quality of Life
**RMST**	Restricted mean survival time
**rPFS**	Radiological progression-free survival
**RT**	Radiotherapy
**RTOG**	Radiation Therapy Oncology Group
**SAE**	Serious adverse event
**SAP**	Statistical Analysis Plan
**SOC**	Standard-of-care
**SOP**	Standard Operating Procedures
**TBD**	To be determined
**TMG**	Trial Management Group
**TSC**	Trial Steering Committee
**WHO PS**	WHO Performance Status
**ZA**	Zoledronic acid

2 BACKGROUND AND DESIGN

This section gives a brief summary of the trial, including the trial aims/objectives; treatment/randomisation arms; main outcomes; and the patient eligibility criteria.

Full details of the background to the trial and its design are presented within the current, activated version of the protocol (Version 21.0; Oct-2020.

2.1 TRIAL SUMMARY

STAMPEDE is a multi-centre, platform protocol, including a number of randomised controlled trials. It recruits patients with locally advanced (M0) or metastatic (M1) prostate cancer who are starting long-term androgen-deprivation therapy (ADT) for the first time. Patients can have either newly-diagnosed disease or have been previously treated with radical radiotherapy or surgery but now have a rising prostate specific antigen (PSA). Further details on eligibility can be found in section 2.3.

The trial uses multi-arm, multi-stage (MAMS) methods to simultaneously assess a number of different research treatments. The trial aims to assess the effects of adding one or two approaches to the standard-of-care (SOC).

The investigational agents to date are:

(i) A bisphosphonate, zoledronic acid

(ii) A cytotoxic chemotherapeutic agent, docetaxel

(iii) A cyclooxygenase (Cox-2) inhibitor, celecoxib

(iv) A CYP-17 inhibitor, abiraterone

(v) Radiotherapy to the prostate amongst newly-diagnosed M1 patients only

(vi) An androgen receptor signalling inhibitor, enzalutamide

(vii) Metformin

(viii) Transdermal oestradiol (tE2)

Patients on the control arm receive the standard-of-care; the research arms have this standard supplemented with other potential treatments, except for patients allocated to the tE2 arm who receive transdermal oestradiol in place of standard hormone treatment.

The standard-of-care is based on ADT, achieved through the use of luteinising hormone-releasing hormone (LHRH) analogues or antagonists, Dual Androgen Blockade (DAB: long-term anti-androgens in combination with LHRH agonist) or bilateral orchidectomy according to local practice (bicalutamide for non-metastatic (M0) patients was allowed in some early versions of the Protocol). This standard-of-care is also the backbone of therapy for the research arms.

Standard-of-care radiotherapy (RT) was mandated for all N0M0 patients (unless contraindicated) and encouraged for N+M0 patients. RT to the prostate was permitted for M1 patients, too, although whilst the M1|RT research Arm H was recruiting, radiotherapy was not permitted as SOC for newly-diagnosed M1 patients and should only be received through allocation to Arm H for patients corresponding to this sub-set.

Standard-of-care docetaxel was permitted from Protocol version 14.0 (Dec-2015) onwards, following the results of STAMPEDE's "original comparisons"[1], along with other external trial results combined within a meta-analysis[2].

Planned use of docetaxel, as reported at randomisation, is included as a stratification factor from this time.

2.2 COMPARISONS

A research comparison is defined by those patients allocated to the research arm, along with the corresponding contemporaneously randomised, eligible control arm patients. See Table 1 for the definition of each research comparison within STAMPEDE to date.

Table 1: STAMPEDE Research Comparisons

Comparison name	Included Arms	Eligible patients	Accrual		Time Period(s)	NB
			Start Date	End Date		
"Zoledronic acid comparison"	A, B	All patients	05-Oct-2005	31-Mar-2013	1-4	Note
"Docetaxel comparison"	A, C	All patients	05-Oct-2005	31-Mar-2013	1-4	Note
"Celecoxib comparison"	A, D	All patients	05-Oct-2005	06-Apr-2011	1	Note
"Zoledronic acid + docetaxel comparison"	A, E	All patients	05-Oct-2005	31-Mar-2013	1-4	Note
"Zoledronic acid + celecoxib comparison"	A, F	All patients	05-Oct-2005	06-Apr-2011	1	Note
"Abiraterone comparison"	A, G	All patients	15-Nov-2011	17-Jan-2014*	3-5	Note
"M1|RT comparison"	A, H	Newly-diagnosed M1 pts<br>No contraindication to RT	22-Jan-2013	02-Sep-2016	4-9	Note
"Enzalutamide + abiraterone comparison"	A, J	All patients	29-Jul-2014	31-Mar-2016	7-9	Note
"Metformin comparison"	A, K	Non-diabetic pts<br>No contraindication to metformin	05-Sep-2016	TBD	10-TBD	---
"tE2 comparison"	A, L	<8wk anti-androgen use<br>Maximum 4wk LHRH t'py<br>No bilateral orchidectomy	20-Jun-2017	TBD	11-TBD	---

***Note:** One patient was manually randomised to Arm G after the cut-off of 17-Jan-2014

D, F: The celecoxib-containing arms closed accrual early due to lack of sufficient activity following their Activity Stage 2 analysis.

B, C, E: The remaining original research arms closed to recruitment having reached an acceptable sample size (based on time to analysis projections).

G: The abiraterone arm closed to recruitment having reached its revised sample size target (1800 pts) ahead of schedule.

H: The M1|RT arm closed to recruitment having reached its revised target sample size ahead of schedule.

J: The enzalutamide + abiraterone arm closed to recruitment having reached its target sample size ahead of schedule.

[1] http://www.thelancet.com/pdfs/journals/lancet/PIIS0140-6736(15)01037-5.pdf
[2] http://www.thelancet.com/pdfs/journals/lanonc/PIIS1470-2045(15)00489-1.pdf

The trial is summarised at relevant times for this comparison in Figures 1 and 2. The trial initially started with Arms A to F. Additional research arms have been included in the trial over time.

At the time a new research arm is activated, the strata totals in the randomisation system are reset; the team checks for imbalances before this occurs. Consideration is given to applying a small weight to the reset total to help correct this only if there is a major imbalance, defined as a difference of 12 patients or more in any given strata (NB to date this has not been required).

Figure 1: Recruiting arms from Jul-2014 to Mar-2016 (Protocol version 12.0)

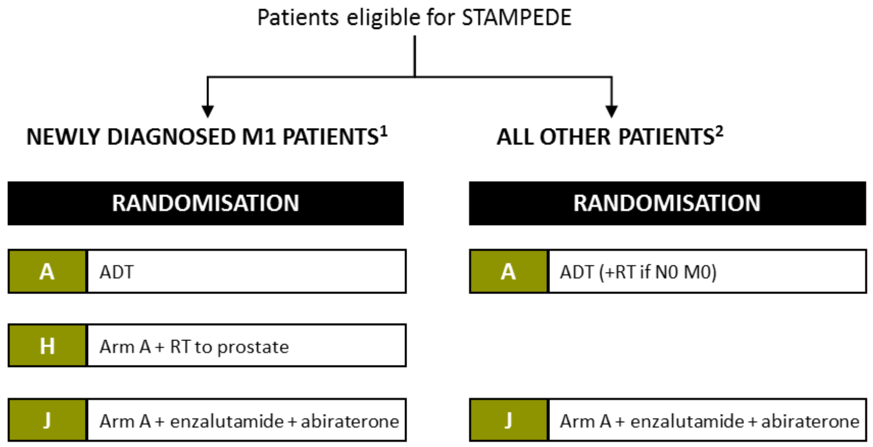

[1] except pts with a contra-indication to RT
[2] all suitable pts with newly diagnosed locally advanced disease should also have RT[1]

Figure 2: Activity-by-time graph showing patients contributing to this comparison

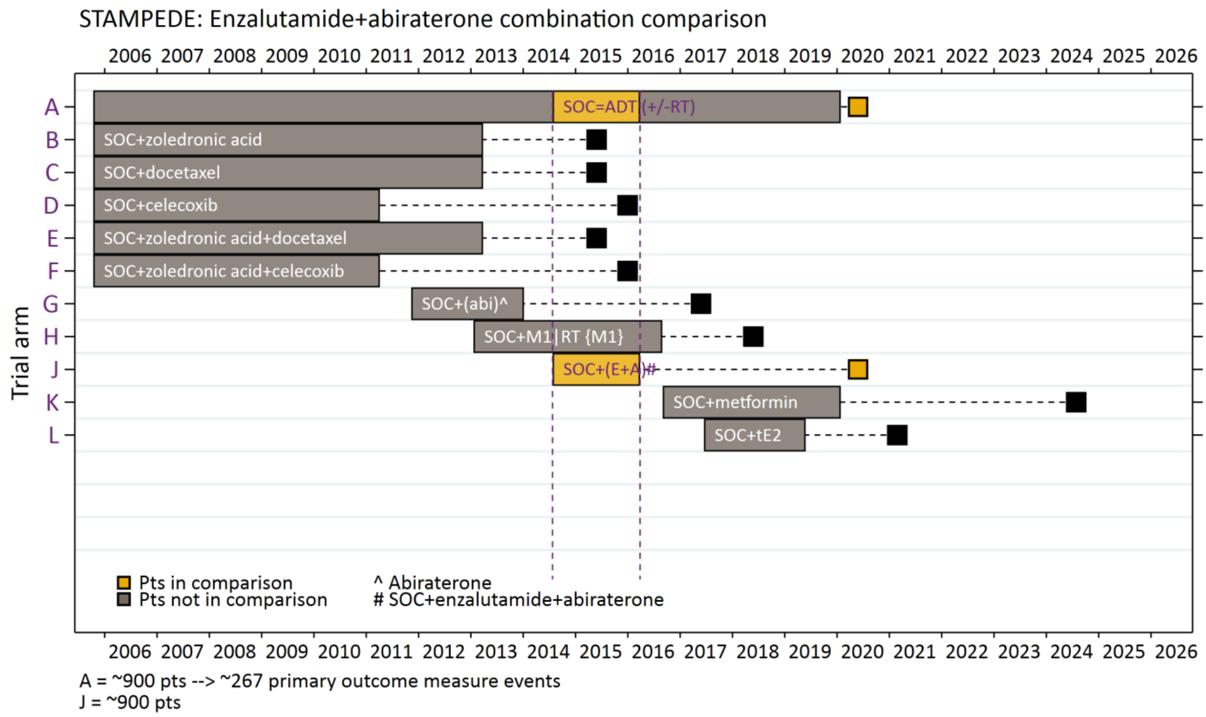

2.3 ELIGIBILITY CRITERIA

The eligibility criteria describe a broad population of patients unified by the need to start long-term ADT for the first time.

In the broadest terms, this includes patients who have had previous local therapy and now have high-risk relapse, along with patients with newly-diagnosed disease. For this comparison, this included patients with:

- High-risk locally advanced disease (at least two of: Stage T3/4 N0 M0 histologically confirmed prostate adenocarcinoma, PSA≥40ng/ml or Gleason sum score 8-10)
- Nodal involvement
- Metastatic disease

All patients need to give written consent, be sufficiently fit for any protocol treatment and follow-up, and meet other detailed eligibility criteria.

The details of the eligibility criteria have changed a little over time; this is shown by protocol version in a specific document [3] which will continue to be updated throughout the trial.

[3] S:\MRCCTU_Stampede\10 Audit & Quality Control\10.1_Monitoring & QC\10.1.1_Monitoring Tools

2.4 METHOD OF RANDOMISATION

Patients are randomised centrally using a computerised algorithm developed and maintained by the CTU. Randomisation is performed using the method of minimisation over a number of clinically important stratification factors with an additional random element. These factors are:

• Randomising centre	each centre
• Metastases	M0 vs M1
• Nodal involvement	N0 vs NX vs N+
• Age at randomisation	up to 69yrs vs 70yrs and over
• WHO performance status	PS=0 vs PS=1-2
• Method of ADT[4]	Orchidectomy vs LHRH agonist vs LHRH antagonist vs Dual Androgen Blockade (DAB)
• Regular aspirin or NSAID use at baseline	yes vs no
• Radiotherapy planned[5]	yes vs no
• Docetaxel planned[6]	yes vs no

When implementing the additional random element of the randomisation, an 80% probability of allocation will be split between the (one or more) arms with the lowest strata totals (i.e. 80% probability of being allocated to one of the minimising arms); and the remaining 20% probability of allocation will be split between the remaining (one or more) arms. This method should provide simplicity of reporting and implementation.

2.5 ALLOCATION RATIO

As of Oct-2020 (Protocol version 21.0), the trial had one control arm and ten research arms. Overall, the allocation ratio has been A:B:C:D:E:F:G:H:J:K:L = 2:1:1:1:1:1:2:2:2:2:2.

Therefore, this comparison had equal weighting for the research and control patients in comparison-eligible patients i.e. allocation ratio 1:1.

[4] Method of ADT options have changed over time, from LHRH vs orchidectomy, to then include bicalutamide, then specify LHRH agonist or antagonist and more recently exclude bicalutamide but include DAB; see S:\\MRCCTU_Stampede_Stats\\SAP\\Stratification_Factors_OverTime

[5] "Radiotherapy planned" was added as a stratification factor at the start of recruitment to Efficacy Stage I for the "original comparisons" (Mar-2008)

[6] Docetaxel planned was added as a stratification factor from 17-Dec-2015 following publication of the "original comparisons" results indicating docetaxel improved overall survival

3 DATA COLLECTION AND PROCESSING

3.1 CASE REPORT FORMS AND VARIABLES

Full details of data collection and timings are described in the protocol[7].

A copy of the Case Report Forms (CRFs) and Quality of Life (QL) questionnaires are presented in the Statistical Master File and the Trial Master File. Details of the variables, and any corresponding validations, are presented in the metadata which forms part of the Trial Master File.

3.2 DATA COMPLETION SCHEDULE FOR CONSENT, BASELINE, TREATMENT & FOLLOW-UP

A record of consent to trial participation in the J comparison is held by the MRC CTU for 1708/1976 patients randomised; for the remaining 268 patients, consent was collected by the local site prior to randomisation but a copy of the completed consent form was not available to the MRC CTU at the time the data were frozen. All patients are included in analyses to maintain the ITT integrity of the trial results.

Table 2 gives detail on the expected timing of scheduled CRFs. Please note that this only applies to arm J; please see earlier versions of the SAP for detail of timings for other comparisons.

Table 2: Timings for completion of scheduled CRFs on arm J

Timing of Assessment		Baseline		Treatment		Outcomes		Freq
		Rand^{n}	Pre-Trt	RT	SOC Doc	Follow-up	QL HE	
Yr 0	Wk 0	✓	✓				✓	
	Wk 6					✓	✓	6 weekly
	Wk 12					✓	✓	
	Wk 18					✓	✓	
	Wk 24				J only	✓	✓	
	Wk 36					✓	✓	12 weekly
	Wk 48			M0 only		✓	✓	
	Wk 60					✓	✓	
	Wk 72					✓	✓	
	Wk 84					✓	✓	
	Wk 96					✓	✓	
Yr 2	Month 24					✓	✓	6 monthly
	Month 30					✓	✓	
Yr 3	Month 36					✓	✓	
	Month 42					✓	✓	
Yr 4	Month 48					✓	✓	
	Month 54					✓	✓	
Yr 5	Month 60					✓	✓	
Yr 6+						✓	✓	annual

Note:

[7] S:\MRCCTU_Stampede\01 Study Protocol_Key Documents\1.1 Signed Protocol

Pre-Trt assessment includes the following CRFs: baseline, cardiovascular, bone density risk factor questionnaire.

QL & HE data were collected for the first 700 patients randomised and then for all patients randomised from Protocol v8.0 onwards (Nov-2011) who opt-in.

QL & HE data is collected until disease progression.

SOC Doc forms are required for all patients randomised from 17-Dec-2015, when the change in SOC to permit upfront docetaxel use was implemented, and for any patients randomised before this date and receiving upfront docetaxel.

3.3 DATA COMPLETION SCHEDULE FOR OTHER 'NON-SCHEDULED' CRFS

The following CRFs should be completed as required:

- Hormone therapy log (All Arms)
- Abiraterone & enzalutamide treatment log (Arms G & J)
- Toxicity form (All Arms)
- RT acute toxicity form (All Arms, pts who received RT only)
- Progression log (All Arms)
- Additional treatment log (All Arms)
- Saliva pathology form (All Arms)
- Blood form (All Arms)
- SAE form (All Arms)
- End of research treatment form (Research Arms only)
- Co-enrolment form (All Arms)
- Death form (All Arms)

For all CRFs, details of associated timings and requirements are given within their guidance notes.

3.4 MANAGEMENT OF DATASETS

The datasets will be managed in accordance with CTU Standard Operating Procedures (SOPs) and in accordance with the Data Management Plan[8]. Full details of data cleaning, verification and lock procedures are contained in this document.

3.4.1 DATA CLEANING

Routine data extracts are run on an approximate fortnightly basis. In preparation for an IDMC review and interim or final efficacy analysis, several of these extracts will be used, at least one to check the data and a final one for analysis. The statistician will work with the data managers to resolves as many queries and discrepancies as possible (entered onto MACRO prior to the final freeze) and this process will be formally documented with the exact details agreed prior to the analysis in the in the Database Lock Quality Checklist.

If any outstanding data queries are resolved during the analysis that relate to data in the final copied dataset (eg. problems that are found during analysis or amended CRFs that are returned to CTU), and it is desired to

[8] S:\MRCCTU_Stampede\08 Data Management\8.6 Data Management Plan

include these changes in the report or analysis being run, corrections should be made in MACRO as usual but the data should not be extracted again. Rather, the corrections should be made in the Statistician's analysis files and appropriately highlighted and annotated (notably any confirmed changes should only be made within cr_fieldupdates.do). For the primary analysis the Senior Statistician will decide on a case-by-case basis whether a correction warrants exporting the data again.

Datasets for all analyses will be constructed and analyses performed using Stata programs (*.do).

3.4.2 DATA VERIFICATION

Data verification, consistency and range checks are performed at the data entry stage by the CTU, as well as checks for missing data, as documented in the trial working practices (copies of these checks can be found in the Trial Master File). Additional range, consistency and missing data checks will be performed automatically within the MACRO databases, as programmed in the database. Copies of the data chase and query report forms are stored electronically, with completed and returned forms stored in patient files. Some of the checks may be duplicated and others will be performed when the datasets for analysis are constructed by the statistician. All outcome variables (primary and secondary) including treatment details will be examined for unusual, outlying, unlabelled or inconsistent values by the Statistician.

In general, given the thorough nature of our follow-up procedure we expect the issue of missing data to be relatively minimal. We anticipate high compliance with initial data collection as this is close to the time of patient registration. If any data is missing, imputation will not routinely be done for the main analyses, but this may be reconsidered if recommended or requested. Sensitivity analyses may be undertaken which include additional variables in statistical models, e.g. alternative adjusted Cox models including other variables considered to be predictive of the outcome. For these analyses, missing values of adjustment variables may be multiply imputed under the Missing At Random (MAR) assumption to avoid reliance on complete case analysis.

Data from sites on overall survival, the definitive primary outcome for patients with baseline M1 disease and a component of the metastasis-free survival primary outcome for patients with baseline M0 disease, and other outcome measures may be supplemented by linked data from the Office of National Statistics (ONS) or a similar system. This would be used to ascertain the final survival status of those not reported as having died on the most recent follow-up, and additionally to help in the ascertainment of causes of death.

Any problems with trial data will be queried with the Trial Managers, Data Managers, or Statistician(s), as appropriate. Where possible, data queries will be resolved, although it is accepted that due to administrative reasons and data availability, the dataset cannot be perfect. Evidence of preferential data collection on one arm would be unacceptable and further data cleaning would be required.

Prior to formal final primary analyses an additional set of checks will be completed. These include a Trial Manager checking primary outcome data in MACRO against the original CRFs (including date of death) for a sample of patients to ensure concordance. These checks are detailed in the Trial Management File, kept by the STAMPEDE Trial Managers.

3.4.3 DATABASE LOCK

A copy of the trial databases will be locked before the primary activity analysis for each research comparison once the Database Lock Quality Checklist has been agreed and the conditions met. However, the live databases will remain open, as long-term follow-up data for patients may be collected in the database after this final activity stage analysis. Additionally some patients contribute to multiple comparisons that may mature at differing times.

3.5 DATA CODING

The coding of the raw variables can be found in the metadata[9]. The latest version has the largest version number "X" at the end of the document name.

Coding of variables for analysis can be found in the Analysis File Specification documentation[10].

3.5.1 FREE-TEXT VARIABLES

If required, review and (re-)coding of free-text variables is carried out by a clinical member of the TMG.

Such free-text variables to be reviewed and (re-)coded include:

- Those items reported as "other, specify", including reason for treatment action; toxicity; additional treatment; and place of death.
- Concomitant medications (collected as free-text at baseline; up to Protocol v15.0, Sep-2016)

3.6 PRE-PLANNED DATA CHECKS

- Current ADT on randomisation form *vs* baseline form (patients randomised before 05-Sep-2016; check for concordance)
- Broad disease category *vs* [pre-HT PSA value, metastatic status, nodal status, gleason score, previous treatment reported]; check for concordance
- Disease progression on FU form *vs* progression form; check for concordance
- Death on progression form *vs* death form [deaths reported before 05-Sep-2016]; check for concordance
- Death reported as an SAE on Death CRF *vs* SAE form with death reported
- Metastatic status (M0 versus M1) concordance between randomisation server and subsequent randomisation form. In a small number of patients, the assessment of metastatic status at randomisation can change on the basis of subsequent testing and imaging such that the randomisation form becomes a more correct representation of the true metastatic status of the patient. For the purposes of separating

[9] S:\MRCCTU_Stampede\08 Data Management\8.4 Metadata and Data Handling\Metadata\STAMPEDE Metadata vX

[10] S:\MRCCTU_Stampede_Stats\Data\Auto_Macro\Analysis File Spec

M0 and M1 analyses, the data from the randomisation form (rather than the rando server) will be used to select patients. The number of patients in whom this status changed will be described in any data report. It is acknowledged that the minimisation factors will differ slightly from the true status.

3.7 DATA RETURNS

- Data Forms returned compared to expectations (routine forms only; randomisation, baseline (incl. cardiovascular & BD Risk), follow-up, radiotherapy detail/acute toxicity)
- Death, progression and end of trial participation forms received for the control arm
- FU forms expected vs number of FU forms received, for each patient
- Scatter plot of timing of most recent received FU for each patient *vs* time from randomisation, by treatment arm (including detail on number of patients randomised, number of deaths, number of withdrawals, number okay and number late for follow-up)
- Number of FU forms dated within each week since patient's randomisation
- Time since last FU form (KM graph; subgroup: randomised <24wks vs 24+wks)
- Duration of follow-up after randomisation (time from randomisation to last contact), by arm, with median using reverse KM plot
- For surviving patients the time since last follow-up form received, by treatment arm
- Details of patients Lost-to-FU or for whom early stopping of follow-up and data collection has been reported

3.8 DATA MATURITY

Median follow-up time will be calculated using a "reversed" Kaplan-Meier approach, taking censor date (if alive) to be an event and death as the time of censorship. The median follow-up time will be detailed by arm and within any pre-defined sub-groups of interest. In patients who were last known to be alive, time to last follow-up will be presented using standard summary statistics. Date last seen (if alive) is as defined within the censor date in Table 4.

3.9 MISSING DATA

Missing data will be assumed to be missing completely at random (MCAR) in the main intention-to-treat analyses. Information on covariates included in the main pre-specified analysis models is collected at randomisation so these key variables should be complete in most cases.

Where appropriate, for sensitivity analyses and additional exploratory analyses, missing data on explanatory variables to be included in analysis models may be multiply imputed using the Missing At Random (MAR) assumption to avoid reliance on complete-case analysis. Evidence in favour of the MAR assumption will be

presented at the same time and sensitivity analyses conducted to assess the impact of data being Missing Not At Random (MNAR) on estimates of treatment effect.

4 OUTCOME MEASURES

For all outcome measures the comparison is between patients on the relevant research arm and those contemporaneously randomised control arm patients eligible for the research arm of interest. Outcomes are listed in Table 3 and definitions are provided in Table 4.

Note: All arms are un-blinded so primary outcome measures for all comparisons are objectively measured, with caution to be taken around interpretation of more subjective secondary outcome measures such as symptomatic skeletal events.

As for other comparisons, the "enzalutamide+abiraterone comparison" was originally designed to use Failure-Free-Survival (FFS) as the intermediate outcome and Overall Survival (OS) as the final primary outcome. However, the ICECaP meta-analysis of patients with M0 disease has demonstrated that Metastasis-Free-Survival (MFS), defined as time from randomisation to distant metastases confirmed by imaging or histological evidence or death from any cause, is a robust surrogate outcome measure for OS in this M0 patient group (9). Therefore, MFS was added to the list of outcome measures in 2021 and will become the primary outcome measure for M0 patients in the combined G and J analysis. Details for this analysis are provided in **Appendix 1**.

Table 3: Trial outcome measures by comparison stage for the J comparison

COMPARISON STAGE	PRIMARY OUTCOME MEASURE	SECONDARY OUTCOME MEASURES[T]
**Pilot phase(s)**	Safety[*]	Feasibility
**Activity Stages**	Failure-free survival (FFS)[†]	Overall survival (OS)[x]<br>Biochemical Failure<br>Progression-free-survival (PFS)<br>Disease-specific survival<br>Non-PCa death<br>Lymph node progression<br>Distant metastases (Metastatic Progression-Free Survival)<br>Toxicity<br>Symptomatic skeletal events<br>Therapy for progression
**Efficacy Stage**	Overall survival[x]	All OMs as for Activity Stages plus:<br>Metastasis-Free-Survival^<br>Quality of life<br>Cost effectiveness

[T] Presented at activity stages where data is mature

[*] Based on toxicity

[†] Including biochemical failure (see Table 4 for definition of FFS)

[x] Cause of death with a view to cause-specific survival from PCa (with death from other causes as a competing risk) is considered under this.

^ As defined in ICECaP 2017 *JCO* paper: DOI: 10.1200/JCO.2017.73.9987

Table 4: Definition of outcome measures and censoring dates

TERM	DEFINITION
Overall survival (OS)	Time from randomisation until death from any cause. For surviving patients, censor date 1 is used; if ONS data is available use censor date 2.
Failure-free Survival (FFS)	Time from randomisation until the first of the following events:<br>• Biochemical failure<br>• Local progression<br>• Lymph node progression<br>• Distant metastases<br>• Skeletal Related Event (where confirmed disease progression)<br>• Death from prostate cancer<br>For patients who have not had an event, censor date 3 is used (see below).<br>If a suspected event is reported for any of: local progression, lymph node progression, distant metastases progression this will be counted as a FFS event.
Progression-free Survival (PFS)	Time from randomisation until first of:<br>• Local progression<br>• Lymph node progression<br>• Distant metastases<br>• Skeletal Related Event (where confirmed disease progression)<br>• Death from prostate cancer<br>For patients who have not had an event, censor date 3 is used (see below).<br>If a suspected event is reported for any of: local progression, lymph node progression, distant metastases progression this will be counted as a PFS event.
Metastatic Progression-Free Survival (mPFS)	Time from randomisation until first of:<br>• Distant metastases<br>• Skeletal Related Event (where confirmed disease progression)<br>• Death from prostate cancer<br>For patients who have not had an event, censor date 3 is used (see below).<br>If a suspected event is reported for distant metastases progression this will be counted as an MPFS event
Metastasis-Free Survival (MFS)	Time from randomisation until first of:<br>• Radiologically-confirmed distant metastases<br>• Death from any cause<br>For patients who have not had an event, censor date 3 is used (see below).
Skeletal Related Event (SRE)	• Bone pain requiring radiotherapy and/or surgery<br>• Pathological fracture with or without disease progression at that cancer site<br>• Metastatic spinal cord compression
Disease-specific survival	Time from randomisation until death from prostate cancer (see below).<br>For patients who have not had an event, censor date 1 is used; if ONS data is available use censor date 2 (see below).

<table>
<tr><th>TERM</th><th>DEFINITION</th></tr>
<tr><td>Death from prostate cancer</td><td>All deaths are reviewed following the death review process[11]. A Statistician runs and maintains the Stata program to automatically assign either PCa or non-PCa as a cause of death with the following rules:
<table>
<tr><th colspan="2">Rule</th><th>Cause death</th></tr>
<tr><td>1</td><td>Primary cause of death is PCa and no secondary causes are reported; progression event prior to death; no evidence of another cancer as an SAE</td><td>PCa</td></tr>
<tr><td>2</td><td>Primary cause of death is pneumonia and secondary cause of death is PCa; progression event prior to death</td><td>PCa</td></tr>
<tr><td>3</td><td>Primary cause of death is neutropenic sepsis and secondary cause of death is PCa; progression event prior to death</td><td>PCa</td></tr>
<tr><td>4</td><td>Primary cause of death is carcinomatosis and secondary cause of death is PCa; progression event prior to death</td><td>PCa</td></tr>
<tr><td>5</td><td>Death is reported as caused by PCa treatment; progression event prior to death</td><td>PCa</td></tr>
<tr><td>6</td><td>Primary cause of death is other primary cancer, and is confirmed by SAE report</td><td>Non-PCa</td></tr>
<tr><td>7</td><td>Primary cause of death is cardiovascular disease; PCa not listed as secondary cause of death</td><td>Non-PCa</td></tr>
</table>
Any patients that cannot by classified by these rules will be reviewed by a clinician. Further information will be sought where there is insufficient information for the reviewer(s) to make a judgement. Where the review is not completed for any reason, the local investigator's opinion will be taken.</td></tr>
<tr><td>Censor date 1 for OS</td><td>Date taken from the latest of the relevant variables defined below:

Date of randomisation (Form 1)
BMD assessment date (scan, blood sample, urine sample)
Date of treatment cycle (as taken from the bisphosphonate, docetaxel; Forms 4, 5, 6)
Date bloods taken (as taken from the bisphosphonate Forms 4, 5)
Date of last SOC docetaxel cycle (Form 21)
Date of any treatment action (Forms 7, 7B, 7C, 7D)
Date of any tE2 treatment action for Arm L patients (Form 25)
Date of tests recorded on hormone results log for Arm L patients (Form 24)
Dates reported on the Follow-up CRF (including date of PSA tests, date of any surgical interventions, date of any SRE, date of any metabolic or cardiovascular event; Forms 7, 7A)
Date of any reported progression event (Form 8)
Date additional treatment started or stopped (Forms 8, 8A)
Date of first/last RT fraction (Form 9A)
Date of late RT toxicity assessment (Form 10)
Date HT/research treatment ended (Form 11)
SAE date (onset, resolved, recent HT or trial treatment administration, start/end date of other treatment, test date) (Form 14)
Date of palliative RT fraction (Form 19)
Date blood or saliva sample obtained as reported on the pathology form (Form 18)
Date of co-enrolment to another trial (Form 15)
Date trial participation ended (Form 20)
Date last known alive (Form 7 from Version 13.0)
Date of death (Censoring date only for outcomes other than overall survival and disease-specific survival; Form 12)

Notes:

Dates from the QoL forms are no longer used as a censor date as these are completed by the patient and cannot be queried for errors.
Dates of form completion are no longer used as the CRF may have been completed retrospectively.
Any date pre-randomisation is ignored within the calculation.
</td></tr>
</table>

[11] : S:\MRCCTU_Stampede_Stats\Data\Death Review\

TERM	DEFINITION
	Unusual dates which have not yet been resolved or dates after the date of the corresponding data freeze will be ignored for the purposes of calculating this censor date.
Censor date 2 for OS	For patients with successful flagging with ONS (or equivalent) a censoring date will be set as 4 or 8 weeks before the ONS data transfer
Censor date 3 for non-OS outcome measures	Date taken from the latest of the relevant variables defined below:<br>• Date of randomisation (Form 1)<br>• Date of assessment on the Follow-up CRF (unless recorded as a missed visit; Form 7)<br>• Date of any reported progression event (If type of progression not included as event; Form 8)

5 SAMPLE SIZE CALCULATIONS

5.1 SAMPLE SIZE

This comparison includes patients allocated to research Arm J and all patients contemporaneously allocated to the control Arm A who meet the eligibility criteria for the research treatment. The sample size calculations were performed using the latest update of –nstage- available at the time, with an allocation ratio of 1:1.

This comparison was added in Jul-2014 and was designed under similar assumptions as the original research comparisons; however a smaller HR for the intermediate outcome measure of 0.70 was targeted with only 2 pre-planned activity analyses.

Consideration was given to ceasing further randomisations to Arm J if it was not showing sufficient evidence of activity on the intermediate primary outcome measure (FFS), just as for the other research arms.

The patient mix for this comparison is likely to represent a more favourable prognosis on average than in earlier comparisons, due to concurrent recruitment of M1 but not M0 patients to Arm H (resulting in a lower proportion of M1 patients in the "enzalutamide + abiraterone comparison").

We anticipated that around 1,800 patients needed to be recruited within 3.5 years to observe ~269 control arm deaths within 6 years. For our target HR of 0.75 for SOC+enzalutamide+abiraterone vs SOC alone on OS this would give 90% power to detect a treatment difference of this magnitude with a one-sided significance level of 2.5%. The timing of the definitive analysis will be partly dependent on the observed overall survival. The default scenario assumed that (i) recruitment is constantly 70pts/m to the trial overall, (ii) the M1|RT Arm H accrues throughout and (iii) a further new research arm with an equal allocation ratio is introduced 18 months after activation of Arm J.

Note that these sample size calculations have been superseded by the new analysis plan set out for M0 patients in the appendix but are presented for completeness and transparency.

5.2 SAMPLE SIZE FOR INTERMEDIATE ANALYSES

Intermediate analyses will be collated and reported to the IDMC. Data will be released to the TSC if required and on to the TMG if deemed appropriate. Not all comparisons will be featured in every intermediate reporting, depending on the stage of the trial, the need for the analysis and time pressures.

Table 5 gives details of the intermediate analyses for all research comparisons.

Table 5: Intermediate and Final Activity Stage Analyses – Targeted vs Observed events

Research Comparison		AS#	Control Arm Events		IDMC / Main Report	IDMC Recommendation
			Target	Observed		
Original	(Arms B-F)	1	113 FFS	129 FFS	30-Mar-2010	Continue as planned
	(Arms B-F)	2	216 FFS	209 FFS	31-Mar-2011	Stop accrual to arms D&F
	(Arms B,C,E)	3	334 FFS	341 FFS	30-May-2012	Continue as planned
	(Arms B,C,E)	4	404 OS	415 OS	13-May-2015	Main report
Abiraterone (Arm G)		1	75 FFS	88 FFS	30-Aug-2013	Continue as planned
		2	142 FFS	162 FFS	07-Feb-2014	Continue as planned
		3	221 FFS	241 FFS	06-Jun-2014	Continue as planned
		4	267 OS	262 OS	10-Feb-2017	Main report
M1|RT (Arm H)		1	75 FFS	69 FFS	06-Jun-2014	Continue as planned
		2	142 FFS	144 FFS	21-Nov-2014	Continue as planned
		3	221 FFS	255 FFS	13-May-2015	Continue as planned
		4	267 OS	TBD OS	Q2/3-2018	Main report
Enzalutamide+abiraterone (Arm J)		1	66 FFS	85 FFS	05-Nov-2015	Continue as planned
		2	139 FFS	136 FFS	22-Mar-2016	Continue as planned
		3	269 OS	345 OS	21-Jun-2021	Main report
Metformin (Arm K)		1	121 OS*	TBD OS*	TBD	TBD
		2	473 OS*	TBD OS*	TBD	Main report

* Events in M1 patients only

5.3 FURTHER DETAILS IN SAMPLE SIZE CALCULATIONS AND DESIGN

For further details relating to the sample size calculations and design for each research comparison, see the Statistical Design Document. All do-files, Stata logs, and related presentations can be found within the relevant subfolder in S:\\MRCCTU_Stampede_Stats\\STAMPEDE_sample_size.

6 STATISTICAL ANALYSIS

The results of the analyses will be reported following the principle of the ICH E3 guidelines on the Structure and Content of Clinical Study Reports.[12]

6.1 TIME PERIODS

The main analysis for all comparisons using Cox PH models will be stratified by each time period when the choice of treatment allocations at randomisation within STAMPEDE was changed, or another fundamental aspect which may affect the patient population being randomised. As of Jun-2017, there are eleven time-defined strata (see Table 6 and Figure 3 below). The main J analysis includes periods 7 to 11.

Table 6: Time periods within STAMPEDE

Time Period	Definition	Accrual		Co-recruiting research arms
		Start date	End date	
1	From the start of the trial up to the stopping of the celecoxib-containing research Arms D & F	05-Oct-2005	06-Apr-2011	B C D E F
2	Post-closure of Arms D & F up to the opening of the abiraterone research Arm G	06-Apr-2011	14-Nov-2011	B C E
3	Post-opening of Arm G up to the opening of the M1 radiotherapy research Arm H	15-Nov-2011	21-Jan-2013	B C E G
4	Post-opening of Arm H up to the closure of the remaining original research Arms B, C & E	22-Jan-2013	31-Mar-2013	B C E G H
5	Post-closure of Arms B, C & E up to the closure of abiraterone research Arm G	01-Apr-2013	17-Jan-2014*	G H
6	Post-closure of Arm G up to the opening of the enzalutamide+abiraterone research Arm J	18-Jan-2014	28-Jul-2014	H
7	Post-opening of Arm J up to the update in SOC to permit planned use of docetaxel as first line treatment	29-Jul-2014	16-Dec-2015	H J
8	Post-update of SOC up to the closure of enzalutamide+abiraterone research Arm J	17-Dec-2015	31-Mar-2016	H J
9	Post closure of Arm J up to the close of M1|RT research Arm H and opening of metformin research Arm K	01-Apr-2016	02-Sep-2016	H
10	Post-opening of Arm K to the opening of transdermal oestradiol research arm L	03-Sep-2016	19-Jun-2017	K
11	Post-opening of Arm L onwards	20-Jun-2017	TBD	K L

***Note:** One patient was manually randomised to Arm G after the cut-off of 17-Jan-2014

[12] http://www.ich.org/fileadmin/Public_Web_Site/ICH_Products/Guidelines/Efficacy/E3/E3_Guideline.pdf

Figure 3: Arms active over time (time strata)

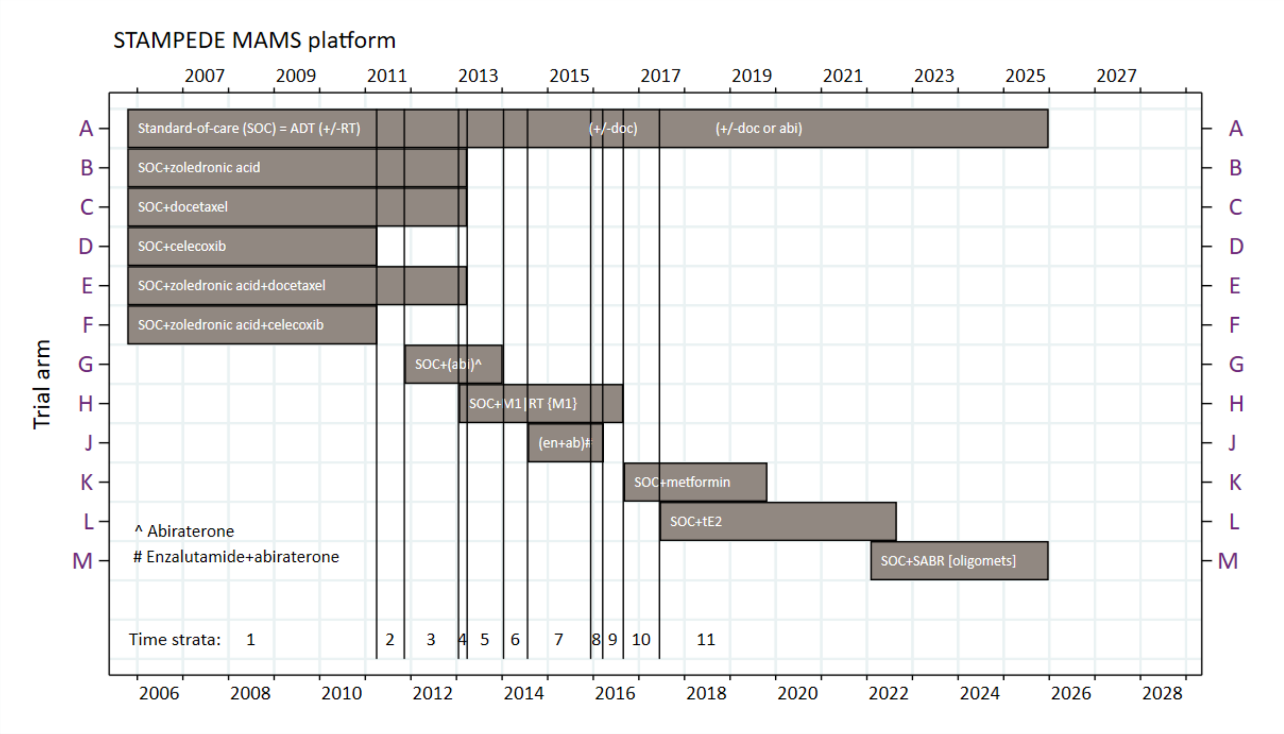

6.2 POPULATIONS FOR ANALYSIS

We define two populations for analysis; the intention-to-treat population and the safety population. The ITT population will be used for all analyses unless specified. The safety population will be included in analyses of adverse events, toxicity and other safety data (safety analyses).

Intention-to-treat (ITT) population

- Comprised of all randomised patients, whether or not they actually received the allocated trial treatment.
- In ITT analyses by treatment arm, patients will be included in the treatment arm to which they were randomised.

Safety population

- Comprised of patients who have been administered at least one dose of their allocated trial treatment.
- Patients will be classed as having started trial treatment if they report a treatment start date, report an SAE which is treatment related, or report death as being within 4 weeks of trial treatment (& related to trial treatment)
- In safety population analyses, patients will be included in the treatment arm corresponding to the treatment they actually started; for example an Arm G or J patient not starting abiraterone or enzalutamide will be excluded from the analysis.

For visual illustration, a CONSORT flow diagram will clearly identify any patients found to be ineligible post-randomisation or stopping trial follow-up early; these patients will be included in relevant analyses where possible. For reference, a template flow diagram can be found in S:\\MRCCTU_Stampede_Stats\\SAP.

6.3 RECRUITMENT AND PATIENT FLOW

6.3.1 RECRUITMENT

- Graph of observed recruitment to the relevant comparison
- Table of recruitment by year & centre
- Table of recruitment across relevant time strata, by treatment arm

6.3.2 PATIENT FLOW THROUGH THE TRIAL

The flow of patients through the trial during the time the relevant comparison was recruited will be presented in a CONSORT diagram that also presents the ITT and safety populations (see section 6.2).

6.4 BASELINE CHARACTERISTICS

The following baseline characteristics will be presented, broken down by treatment arm unless otherwise stated, either as n (%) or median (IQR; min-max) as applicable. Any imbalances between the arms will be assessed using χ^2 or Fishers Exact tests for categorical data and t-tests or Mann-Whitney U tests for continuous data.

- All stratification factors (see section 2.4)
- Randomisation CRF data:
  - Age at randomisation (years)
  - PSA at randomisation (ng/ml; defined as PSA pre-HT)
  - Ln (PSA) at randomisation (defined as Ln (PSA) pre-HT)
  - Time from diagnosis to randomisation (days)
  - Pain from prostate cancer at randomisation: Absent; Present
  - Broad disease category: N0M0 new; N+M0 new; M1 new; Local treatment now relapsing
  - T-stage at randomisation
  - N-stage at randomisation
  - Any metastases at randomisation
  - Bone metastases at randomisation
  - Liver metastases at randomisation
  - Lung metastases at randomisation
  - Nodal metastases at randomisation
  - Other metastases at randomisation
  - Metastatic volume (when available)
  - Use of aspirin
  - Use of NSAIDsUse of short-term bisphosphonates (subgroup: broad disease category)

  - Planned type of HT (randomisation CRF; subgroup: broad disease category)
  - Planned use of long-term anti-androgens
  - Planned use of radiotherapy (subgroup: nodal & metastatic status)
  - Cumulative randomisations (subgroup: nodal, metastatic & planned RT status)
  - Participation in QL study
  - Time from randomisation to starting current HT (times negative if pt starts HT pre-randomisation)
  - Previous HT type: None; LHRH (agonist or antagonist if known); AAs alone; DAB
  - Previous local therapy type (if known): None; radical prostatectomy; radical radiotherapy; radical prostatectomy with post-operative radical radiotherapy; other
  - Duration of previous HT (days)
  - Months between end of previous HT and randomisation (subgroup: broad disease category)
- Baseline CRF data:
  - Gleason sum score at presentation
  - T-stage at presentation
  - N-stage at presentation
  - M-stage at presentation
  - PSA at first presentation (ng/ml)
  - Ln(PSA) at first presentation
  - Concomitant medications (to be clinically recoded)
- Cardiovascular assessment data
  - Smoking status (subgroup: broad disease category)
  - Diabetes and type (subgroup: broad disease category)
  - History of MI, CV disease, CHF, angina or hypertension (subgroup: broad disease category)

6.5 STANDARD OF CARE TREATMENT

For all standard-of-care treatments the following data will be presented, broken down by treatment arm:

- Hormone therapy details (from the FU CRF, HT CRF or the HT Log)
  - Numbers reporting treatment action of changing or stopping (N, %)
  - Time to treatment action
  - Reason for treatment action
- Docetaxel treatment details (from the SOC Docetaxel Treatment CRF; planned details from Randomisation CRF)
  - Reported vs planned docetaxel
  - Time from randomisation to first cycle of docetaxel (days)
  - Time from starting ADT to first cycle of docetaxel (days)
  - Number of cycles administered
  - Reason for less than 6 cycles
  - Daily steroid formulation

    - Daily steroid dose
- Radiotherapy treatment details (from the RT detail CRF; planned details from the Randomisation CRF)
    - Reported vs planned use of RT
    - Timing from randomisation to first fraction of RT in:
        - All patients
        - Patients planned for RT
        - N0M0 patients
        - N+M0 patients

6.6 TRIAL TREATMENT

For **abiraterone** and **enzalutamide** research treatment data the following analyses are planned:

- Number of patients starting allocated trial treatment (N, %)
- Details of patients confirmed as not starting allocated trial treatment (as reported on the End of Research Treatment CRF)
- Time from randomisation to start of treatment (in all pts; censor those who don't report starting)
- Starting dose
- Number of treatments administered (where multiple allocated; i.e. Arm J enzalutamide + abiraterone)
- Treatment compliance/tolerance
    - Numbers reporting treatment action of changing or pausing or restarting (N, %)
    - Time to treatment action (only in pts who report starting)
    - Reason for treatment action (including associated toxicity details if relevant)
    - Length of each treatment break
    - Proportion of missed days
- Frequency and reason for dose modification and delays, treatment termination
    - Include detail of any protocol deviations in relation to overdoses.
- Permanent cessation of trial treatment (as reported on the End of Research Treatment CRF)
    - Numbers (N, %)
    - Time from randomisation to stopping (Overall & subgroup: metastatic status)
        - Censor at randomisation if explicitly reported as not starting
        - Censor at last contact if reported as starting but no report of permanent stopping
    - Time from starting treatment to stopping in patients who report starting (Overall & subgroup: metastatic status)
        - Censor at last contact if reported as starting but no report of permanent stopping
    - Reason (including associated toxicity details if relevant)

Plus

- Additional treatments given (as reported on the Additional Treatment CRF)
    - Include detail of any patients receiving non-protocol disease-directed interventions prior to study outcomes

6.7 SURVIVAL OUTCOME MEASURES

6.7.1 PRIMARY OUTCOME MEASURE

The primary outcome measure up to March 2021 was overall survival (see Table 4 for definition). From March 2021, for analyses where the M0 patients are analysed separately, the primary outcome measure for M0 patients will be metastasis-free-survival (MFS), see Table 4 for definition. This is based upon the ICECAP consortium recommendation that MFS is a suitable surrogate for overall survival in these patients. Overall survival remains the primary outcome measure for M1 patients.

The following data will be presented overall and split by metastatic status, if required:

- Incidence of death, by treatment arm
- Estimate of survival by year from randomisation, by treatment arm (%, 95% CI)
- Cause of death, by treatment arm (death form and death review process)
- Reported cause of death *vs* review cause of death
- Place of death, by treatment arm
- Death within 4 weeks of trial drug, by treatment arm
- Death related to trial treatment, by treatment arm
- Time from randomisation to death from any cause, by treatment arm
    - KM survival plot and median from FPM
    - Censor individuals at last contact if not died
- Comparisons of research vs control from Cox model
    - Adjusted for all stratification factors except centre and method of hormones; stratified by time period

6.7.2 SECONDARY OUTCOME MEASURES

For all secondary outcome measures the following analyses will be performed for the research comparison, overall and split by metastatic status, unless specified:

- Incidence of the outcome, by treatment arm
- Estimate of (*freedom from*) outcome by year from randomisation, by treatment arm (%, 95% CI)
- First reported progression event, by treatment arm [FFS only]
- Time from randomisation to outcome, by treatment arm
    - KM survival plot and median from FPM
    - Censor individuals at last contact if outcome not reported
- Comparisons of research vs control from Cox model adjusted for stratification factors (excluding centre and method of hormones); stratified by time period.

6.7.3 STATISTICAL ANALYSIS METHODS

Time-to-event data will be presented using Kaplan-Meier curves. Censoring dates will be used in all time-to-event analyses for patients who have not experienced the event in question (e.g. progression, death), as defined in

Table 4. For KM plots, all patients randomised to the comparison being analysed will be included. Those patients who have no reported event and contribute no information, such that they are censored at the date of randomisation, will be censored with a time of 0.1 days.

The HR will be analysed using a Cox proportional hazards model, adjusted for those stratification factors used at randomisation (see section 2.4) except for centre and method of hormones, and stratified by the relevant time period/strata for when the comparison was recruiting (see Table 6). This adjusted estimate will be the primary analysis estimate of treatment effect.

Differences in time-to-event outcomes will be assessed using the log-rank χ^2 test and expressed using a hazard ratio (HR) with both a one-sided 97.5% confidence interval and also a 2-sided 95% confidence interval; where the design alpha differs from 0.025 the upper limit of the corresponding one-sided confidence interval will be presented (for example, for an alpha of 0.25 this would be the upper limit of a one-sided 75% confidence interval). The number of events observed and the log-rank expected number of events will be presented.

Alternative estimation of the treatment effect will use exploratory methods e.g. log-rank hazard ratio. Flexible parametric models, modelling the difference between treatment groups over time, will be fitted to the time-to-event data with and without including time-dependent treatment effects.

6.7.4 PROPORTIONAL AND NON-PROPORTIONAL HAZARDS

The assumption of proportional-hazards (PH) will be tested on the basis of Schoenfeld residuals after fitting the Cox model. Application in Stata will be using the command `-estat phtest-` with evidence of non-PH between the two treatment groups being the main focus. Appropriate methods will be applied to the data in the case of any violation and presented alongside the primary analysis to aid interpretation; see details below.

If there is no evidence of non-PH in the treatment effect, the HR for the adjusted Cox model takes primacy.

If there is evidence of non-PH in the treatment effect, the HRs can be difficult to interpret and the restricted mean survival time (RMST) (or "restricted mean time-to-event time") difference constructed from a flexible parametric model with time-varying treatment effect will take primacy. This analysis will use a flexible parametric model, adjusted for the stratification factors used at randomisation (except for centre and method of hormones) and relevant time strata, to determine the time-dependent treatment effect and then predict values needed for subsequent RMST analysis. The application of this methodology will be particularly useful in the case where there is adequate evidence that the proportional hazard assumptions are violated at the 5% level (i.e. $P<0.05$). Application in Stata will be using the command `-strmst-`.

RMST will be calculated and reported for the main treatment effects regardless of whether the PH assumption is violated, as this is recognised as a useful measure. The time within which RMST will be determined by the timing of events in the control arm (using the command `-maturity_rmst-` in Stata). For this purpose, t* (the time up to when we want to compute the outcome measure of RMST) will be defined as the maximum available follow-

up time (if clinically meaningful) where there is most power for the analysis, or a suitable salient time point otherwise, for the research comparison of interest at the time of the analysis.

6.7.5 Competing Risks

Analysis of those outcomes where there are considered to be competing risks will be performed by fitting an adjusted Cox model as well as a competing risks regression model, with a competing risk defined as any event the patient would likely have experienced had they not experienced the outcome of interest first. To date, outcomes with competing risks are FFS, PFS, mPFS, SRE and disease-specific survival where the competing risk is death from non-PCa cause.

6.7.7 PATIENTS WITH NO DATA POST RANDOMISATION

All models used to estimate treatment effect will include those individuals with no reported outcome event and contributing no censoring information such that they are censored at their date of randomisation (t=0.001 days after randomisation).

6.7.8 WITHDRAWN PATIENTS WITH OUTCOME DATA

In May-2019 it was highlighted that a small number of patients who had withdrawn consent for further data collection had outcome data (e.g. death date) recorded in the database; as a result, they were included as events rather than censorings in analyses. To avoid this issue in future analyses, the code used to produce time-to-event and event indicator variables was updated. Any patients who withdraw consent for future data collection and for whom an outcome event has not been reported up to the date of withdrawal are now included as censored on the date of withdrawal in the relevant analysis.

6.7.9 SENSITIVITY ANALYSIS TO ASSESS IMPACT OF COVID-19 PANDEMIC

From March 2020, there was significant disruption to UK cancer services as a result of the COVID-19 pandemic. It is unclear whether this disruption had an impact on the ways in which patients were followed up or assessed for progressive disease. Therefore, for all analyses where it is considered possible that results could be affected by the pandemic, a sensitivity analysis will be undertaken where the administrative censoring date of 1st March 2020 is used as a truncation date. A visual (not statistical) comparison will be made between the point estimates from primary analyses that include and exclude data after this date and the numbers of events and censoring occurrences excluded will be summarised by randomised group.

6.8 PRE-SPECIFIED SUBGROUP ANALYSES

STAMPEDE recruits patients at high risk of dying from prostate cancer and who are starting long-term hormone therapy for the first time (hormone-naïve disease). This is a broad spectrum of patients including both men with metastases at randomisation and men without metastases, and men with de novo disease or disease relapsing after previous local therapy.

6.8.1 STRATIFICATION FACTORS USED FOR MINIMISATION

The stratification factors described in section 2.4 (apart from recruiting centre or hormone use) used for minimisation at the point of randomisation will form subgroups in which treatment effect will be assessed, with an interaction p-value of less than 0.1 used to suggest evidence of a difference in treatment effect across the relevant subgroups. As with all subgroups, we accept that there is limited power to detect an interaction and for analyses restricted to patients in a particular subgroup. The raised probability of a type 1 error from multiple testing will be acknowledged. As a result these subgroup analyses will be regarded as exploratory.

6.8.2 OTHER PRE-SPECIFIED SUBGROUP ANALYSIS

6.8.2.1 METASTATIC VOLUME IN M1 PATIENTS

During the trial, interest has grown in estimating the "volume" of metastases, following analyses of docetaxel in CHAARTED which divided its metastatic patients into "low volume" and "high volume" metastatic disease, and of abiraterone in LATITUDE which divided metastatic patients into "low risk" and "high risk", and only recruited the latter. Volume is being measured by retrospective collection of bone and CT scans for STAMPEDE patients. When available, subgroup analysis of treatment effect by volume will be conducted.

6.9 PSA RESPONSE

PSA response data within the first 24 weeks on trial are used to determine a patient's biochemical progression category (see Protocol for details). Of interest is whether this differs by allocated treatment arm, therefore the following data should be presented by treatment arm on an intention-to-treat basis:

- Boxplot of Log of PSA nadir (to 24 weeks after randomisation)
  - Give data underpinning this including N, min, Q, Max, mean, SD
- Biochemical progression category, N (%)

6.10 TOXICITY AND SAFETY ANALYSES

Toxicity data will be reported using the NCI Common Toxicity Criteria for Adverse Events and presented in the **ITT populations for interim analysis reports and annual review reports** to the IDMC; however breakdown of safety/toxicity data should be presented in the **safety population for final analysis publication and reports**, with only headline figures shown for the ITT population to demonstrate comparability of the populations.

Data presented by treatment arm should be:

- KM plot of time to first G3-5 toxicity reported on the FU, Toxicity or SAE CRF; include maximum SAE grade
- Worst toxicity grade in any category (Overall & subgroup: metastatic status); in each category; proportion with grade 3-5, for the following time points/periods:
  - Within 6m from randomisation (up to and including week 30)

  - At 1 year (+/- 12 weeks)
  - At 2 years (+/- 12 weeks)
  - Ever on trial
- Time to first grade 3-5 SAE
- Time to any grade SAE
- Time to first grade 3-5 SAR
- Time to any grade SAR
- Time to first grade 3-5 SUSAR
- Time to any grade SUSAR
- Table of all reported SAEs reviewed as being related to treatment (classified as a SAR or SUSAR); table to include the following details ordered by trial arm and Patient ID:
  - Patient ID
  - Date of randomisation
  - Date of onset
  - Main diagnosis and associated symptom(s)
  - Grade (as determined by clinical reviewer; site-attributed grade used if not yet reviewed)
  - Why the event is serious
  - SAE status
  - Causal relationship to HT/trial drug; related? expected?
  - Reviewed relatedness; SAR or SUSAR

Safety analyses will be performed and presented on the safety population. All safety analyses will focus on adverse events experienced during treatment and up to 30 days after the end of research treatment (see Protocol).

Safety will be evaluated by tabulation of adverse events at or up to pre-defined follow-up time points. Adverse events will be classified using the NCI Common Toxicity Criteria for Adverse Events (CTCAE v4.0 for all assessments dated from Protocol v15.0 (05-Sep-2016) onwards; assessments made before this using CTCAE v3.0 will be re-coded to fit with CTCAE v4.0) and summarised for each treatment arm. Reported grading is "0 = toxicity not experienced" up to "5 = fatal".

Adverse events (AEs) may be detected through several sources reported by sites on CRFs:

1. **Follow-up CRF–** routinely reported symptoms and "toxicities" (severity not seriousness reported)
   - AEs reported here up to Sep-2016.
2. **Toxicity CRF –** prompted reporting of symptoms and "toxicities" (severity not seriousness reported)
   - AEs reported here from Sep-2016 onwards.
   - Linked to routine follow-up visits, where sites are asked to report any toxicities experienced in the period covered by the follow-up assessment; and treatment actions and permanent stopping of treatment where toxicity is given as the reason for the action.

3. **SAE CRF –** spontaneously reported serious adverse events (severity and seriousness reported)

Not all serious events are severe nor are all severe events serious.

"Seriousness" is a term specific to the reporting of events to regulatory bodies. We have prioritised the consideration of "severity" for balancing evidence of treatment side-effects against activity data. SAE forms and follow-up forms both request the severity of events. Therefore, these sources can be merged to form one dataset for reporting the **severity** of toxicities experienced across different body systems specific disease categories. The focus of severity-reporting will be on toxicities with grade 3, 4 or 5 (fatal), however all toxicity grades will be reported for completeness.

For toxicity data reported **at** pre-specified set time points from randomisation (e.g. at one year since randomisation), this will be reported only for patients who have not progressed before the set time point; this is because for patients on either the control arm or research arms where trial treatment is to be stopped at progression, toxicity data is only expected up to the time of first reported progression. For patients on research arms where trial treatment can (and should) continue beyond first progression (i.e. beyond a first FFS event), namely current Arms G, J and K, an additional report of toxicity data at specific time points from randomisation will be presented only for research arm patients still on treatment at that time; this is because toxicity data for their contemporaneous control patients would not have toxicity collected after the FFS events. Reporting windows will be defined around these set time points which will be as close to the time of interest while accepting that clinical practice means that most patients will not be reviewed on a specific day. These windows are as follows:

- Toxicity at one year on trial: based on information provided for follow-up assessment or SAE report closest to a patient's 1-year anniversary of randomisation to the trial, within 12 weeks of this anniversary. Patients are included in the relevant cross-sectional analysis if progression / permanent treatment stopping has not been reported by 48 weeks since randomisation.

- Toxicity at two years on trial: based on information provided for follow-up assessment or SAE report closest to a patient's 2-year anniversary of randomisation, within 12 weeks of this anniversary. Patients are included in the relevant cross-sectional analysis if progression / permanent treatment stopping has not been reported by 96 weeks since randomisation.

For toxicity data reported **up to** pre-specified time points from randomisation this will include all patients with follow-up/toxicity/SAE data available within that time frame.

All patients receive ADT as standard-of-care and so interest will be in the additional toxicity reported for patients on research arm relative to control arm, compared informally. Interest will also be in any proportion of known treatment toxicity above that which is expected in this population.

"Relatedness" is only collected for SAEs and cannot be reported for all adverse events.

6.11 QUALITY-OF-LIFE ANALYSIS

Quality of life data will not be analysed as part of the primary analysis, but may be analysed separately at a later date. A separate SAP will be developed for such analyses.

6.12 HEALTH ECONOMICS

Health economic data will not be analysed as part of the primary analysis, but may be analysed separately at a later date. A separate SAP will be developed for such analyses.

7 APPENDIX 1

Statistical analysis plan for combined primary analysis of M0 patients in the A-G and A-J comparisons.

This appendix relates to a combined analysis of the M0 patients recruited into either the Abiraterone (G) or Abiraterone + Enzalutamide (J) comparisons. The appendix should be used in combination with the main Arm J statistical analysis plan that describes in more detail the methods for analysis of the whole comparison.

Please note, in a small number of patients, the assessment of metastatic status at randomisation has changed on the basis of subsequent testing and imaging such that the randomisation form becomes a more correct representation of the true metastatic status of the patient. For the purposes of separating M0 and M1 analyses, the data from the randomisation form (rather than the rando server) will be used to select patients. The number of patients in whom this status changed will be described in any data report. It is acknowledged that the minimisation factors will differ slightly from the true status.

7.1 RECRUITMENT AND PROGRESS FOR A-G AND A-J COMPARISONS

Recruitment into the A-G (abiraterone) comparison commenced in Nov 2011 and closed in Jan 2014 with a total of 1917 patients randomised using a 1:1 ratio. The primary results were published in 2017 with results reported by M0 (N=915) and M1 (N=1002) subgroups. Long-term results for the M1 patients are close to submission for publication. Follow-up will continue in all patients until summer 2021 with close out of the comparison at sites by June 2022. Patients still receiving abiraterone will be given access to continued drug supply but no further outcome data will be collected on them apart from SAE reporting.

Recruitment into the A-J (abiraterone + enzalutamide) comparison commenced in July 2014 and closed in March 2016 with a total of 1976 patients randomised using a 1:1 ratio. The primary analysis was planned to occur when 267 deaths had occurred in the control arm for all patients. We have already reached this event total but rather than undertake the final analysis in all patients, we aim to modify the final statistical analysis and the rationale for this is described below. We plan to continue follow-up of the A-J comparison until summer 2021 with close out of the comparison at sites by June 2022. Patients still receiving abiraterone and/or enzalutamide will be given access to continued drug supply but no further outcome data will be collected on them apart from SAE reporting.

7.2 NEW KNOWLEDGE WITH IMPLICATIONS FOR THE FINAL ANALYSIS OF THE A-J COMPARISON

Abiraterone is more effective than was expected when Arm A-J was designed. The target HR for the Arm A-G comparison was 0.75 and the observed HR was 0.63, 95% confidence interval [CI], 0.52 to 0.76; P<0.001 (1). Failure free survival benefit was even greater: 0.29; 95% CI, 0.25 to 0.34; P<0.001. These results were confirmed in the independent, industry-sponsored LATITUDE trial (2). Similar benefit has been observed with enzalutamide in the EnzaMet trial, although the comparison in the latter trial was ADT with a non-steroidal anti-androgen (3), and with the anti-androgen apalutamide in the industry-sponsored Titan trial (4). Given the high efficacy of abiraterone or enzalutamide as single agents, it is considered unlikely that we will detect superiority

for the combination over the single agents in an unselected population. This is especially notable in the M0 cohort where only 34/460 patients in the active arm died at primary analysis of Arm A-G (1).

Two of three trials in mCRPC evaluating combination CYP17A1 and AR inhibition have been reported. Both found no increase in efficacy for the combination but a slight increase in toxicity (5). Most notably, the Alliance study randomized 1311 1st line mCRPC patients 1:1 to abiraterone and enzalutamide versus enzalutamide. This was an open label trial and no OS benefit was found (NCT01949337, presented at ASCO 2019 by Morris et al.). The third trial is the industry-sponsored ACIS trial that randomised 983 1st line mCRPC patients to abiraterone with apalutamide versus abiraterone with placebo (NCT02257736) and is due to be reported in 2021.

Given the major differences in event rates for M0 and M1 patients we propose to split these two populations for the A-J primary analysis and combine them with the patients from the A-G comparison testing abiraterone alone. Thus, we will be testing the efficacy of androgen receptor (AR) therapy (using abiraterone alone or in combination with enzalutamide) in M1 and M0 patients separately. The analysis of M1 patients is likely to form part of a number of biomarker-stratified analyses aiming to identify both prognostic and predictive biomarkers to inform de-escalation or escalation of AR therapy use in metastatic disease. Separate SAPs will be developed for these analyses.

7.3 PROPOSED OUTCOME MEASURES FOR M0 PATIENTS

As for other STAMPEDE comparisons, A-G and A-J have been designed to use Failure-Free-Survival (FFS) as the intermediate outcome and Overall Survival (OS) as the final primary outcome. However, the ICECAP meta-analysis of patients with M0 disease has demonstrated that Metastasis-Free-Survival (MFS), defined as time from randomisation to distant metastases confirmed by imaging or histological evidence or death from any cause, is a robust surrogate outcome measure for OS (6). A similar meta-analysis of patients with M1 disease is currently under way (STOPCAP) but has not yet reported. Therefore, we propose to use MFS as our primary outcome measure for the M0 patients but will keep OS as our primary outcome measure for the M1 patients. A full description of these outcome measures is provided in the main Arm J SAP.

See Addendum 1 for a summary of the number of events in the control arm for different outcome measures in the A-G and A-J comparisons based upon data extraction in July 2019.

Proposed combined analysis of A-G and A-J comparisons for M0 patients

By combining M0 patients from A-G and A-J into one meta-analysis we will have 920 M0 patients randomised between Abi and control in G and 1062 M0 patients randomised between Abi/Enza vs control in J => total meta-analysis size of 1982 patients equally randomised between AR therapy vs control over a 4.5 year recruitment period.

There was ~6 month gap in recruitment between Jan-June 2014 between close of G and activation of J. Thus, there are no shared control group issues for the M0 patients as each trial has their own set of unshared concurrent controls.

Addendum 1 summarises the number of MFS events in the control arms of the M0 patients in A-G and A-J, with a total of 135 (A-G) and 87 (A-J) = 222 MFS events having occurred by July 2019.

Addendum 2 provides the nstage software output from Stata indicating the number of events we need in the control arms of A-G and A-J to power a robust comparison in M0 patients. The following assumptions were used:
Using 70% survival at 66 months (taken from the M0 patients in the A-G control arm curves)
Assume a recruitment rate of 37 patients per month for 4.5 years (54 months) => 1998 patients
Target power for primary analysis: 90%, significance level of 1.25% (one-sided) to account for one previous primary analysis of M0 patients in the A-G comparison.
Target HR for treatment: 0.75
For a simple 2-arm trial as there are no shared M0 controls, we require 315 MFS events in the control arm. Further consideration of the number of shared events with the 2017 G analysis indicates that only one quarter of the events in the control arm have been used in that previous 2017 analysis so our one-sided significance level of 1.25% is conservative. Accounting for the actual number of shared events indicates that this can be relaxed to 2.18% and this would lead to the need for at least 277 MFS events to have occurred in M0 patients in the combined control arms of G and J. There will also be a small loss in power associated with combining the datasets using a meta-analysis approach so boosting the power to 92% will require 299 events in the M0 control arms. Thus, we will aim for at least 300 MFS events to have occurred in the control arm before we undertake the analysis.

7.4 TIMELINES FOR REPORTING M0 RESULTS

We have used ARTPEP and other forecasting approaches to predict when ~300 control arm events will be reached and this suggests that it will occur in Q2 2021 (see Addendum 3). Following data cleaning and analysis, we anticipate that this analysis of M0 patients will be able to report by Q4 2021 at the latest. It is possible we may be able to analyse earlier to coincide with presentation of results at ESMO 2021 but this will depend on the extent of data cleaning and quality by early August 2021.
Meta-analysis methods for the combined analysis of M0 patients in G and J
An IPD meta-analysis will be undertaken to combine the G and J comparisons for M0 patients. Definition of outcomes, handling of data and analysis methods for survival outcomes will be as described in the main SAP for the primary analysis of the Abiraterone + Enzalutamide comparison (version 3.0). As for the main analysis the adjusted treatment effect estimate is regarded as the primary analysis and this pooled meta-analysis will combine the estimates adjusted for the minimisation strata.
After consultation with the MRC CTU meta-analysis group, it was agreed that a fixed effects meta-analysis would be most appropriate for the pooled estimation of treatment effects in this meta-analysis. Given the longer follow-up in the A-G comparison we anticipate a more precise estimate than that for the A-J comparison and it is possible that the A-J comparison treatment effect estimate will be larger than for A-G as it is testing a combination of two AR therapies. Both of these aspects are likely to generate some trial heterogeneity but a fixed rather than random effects meta-analysis was preferred as a) we are exploring the pooled effect of any AR therapy in these patients and b) all aspects of trial delivery have been almost identical for the two comparisons in terms of sites, protocols and eras of SoC. Forest plots will be used to present the adjusted estimates for each comparison separately alongside the pooled estimate.

7.5 REFERENCES

1. James ND, de Bono JS, Spears MR, Clarke NW, Mason MD, Dearnaley DP, et al. Abiraterone for Prostate Cancer Not Previously Treated with Hormone Therapy. N Engl J Med. 2017;377(4):338-51.

2. Fizazi K, Tran N, Fein L, Matsubara N, Rodriguez-Antolin A, Alekseev BY, et al. Abiraterone plus Prednisone in Metastatic, Castration-Sensitive Prostate Cancer. N Engl J Med. 2017;377(4):352-60.

3. Davis ID, Martin AJ, Stockler MR, Begbie S, Chi KN, Chowdhury S, et al. Enzalutamide with Standard First-Line Therapy in Metastatic Prostate Cancer. N Engl J Med. 2019;381(2):121-31.

4. Chi KN, Agarwal N, Bjartell A, Chung BH, Pereira de Santana Gomes AJ, Given R, et al. Apalutamide for Metastatic, Castration-Sensitive Prostate Cancer. N Engl J Med. 2019;381(1):13-24.

5. Attard G, Borre M, Gurney H, Loriot Y, Andresen-Daniil C, Kalleda R, et al. Abiraterone Alone or in Combination With Enzalutamide in Metastatic Castration-Resistant Prostate Cancer With Rising Prostate-Specific Antigen During Enzalutamide Treatment. J Clin Oncol. 2018;36(25):2639-46.

6. Xie W, Regan MM, Buyse M, Halabi S, Kantoff PW, Sartor O et al. Metastasis-Free Survival Is a Strong Surrogate of Overall Survival in Localized Prostate Cancer. J Clin Oncol. 2017 Sep 20;35(27):3097-3104. doi: 10.1200/JCO.2017.73.9987.

7.6 ADDENDUM 1 - NUMBERS OF EVENTS IN CONTROL ARMS BY JULY 2019

Abiraterone comparison (A vs G) - control event numbers

```
   Has FFS |
     event |      Mets status
 occured? |        M0          M1 |     Total
-----------+----------------------+----------
  No event |       263          69 |       332
 Yes event |       192         433 |       625
-----------+----------------------+----------
     Total |       455         502 |       957

   Has MFS |
     event |
   (ICECAP |
definition |      Mets status
) occured? |        M0          M1 |     Total
-----------+----------------------+----------
  No event |       320         143 |       463
 Yes event |       135         359 |       494
-----------+----------------------+----------
     Total |       455         502 |       957

       Has |
   patient |      Mets status
     died? |        M0          M1 |     Total
-----------+----------------------+----------
  Not died |       362         195 |       557
  Yes died |        93         307 |       400
-----------+----------------------+----------
     Total |       455         502 |       957
```

Enz+Abi comparison (A vs J) - control event numbers

```
   Has FFS |
     event |      Mets status
  occured? |        M0          M1 |     Total
-----------+----------------------+----------
  No event |       413         103 |       516
 Yes event |       120         351 |       471
-----------+----------------------+----------
     Total |       533         454 |       987

   Has MFS |
     event |
   (ICECAP |
definition |      Mets status
) occured? |        M0          M1 |     Total
-----------+----------------------+----------
  No event |       446         188 |       634
 Yes event |        87         266 |       353
-----------+----------------------+----------
     Total |       533         454 |       987

       Has |
   patient |      Mets status
     died? |        M0          M1 |     Total
-----------+----------------------+----------
  Not died |       479         258 |       737
  Yes died |        54         196 |       250
-----------+----------------------+----------
     Total |       533         454 |       987
```

7.7 ADDENDUM 2 - NSTAGE CALCULATION FOR TARGET CONTROL ARM EVENT NUMBER, BASED ON OBSERVED MFS IN COMBINED ANALYSIS OF A-G AND A-J FOR M0 PATIENTS

```
nstage, s(0.7) t(66) tunit(4) accrue(37) alpha(0.0125) omega(0.9) hr0(1)
hr1(0.75) tstop(54) nstage(1) aratio(1) arms(2)

n-stage trial design                  version 3.0.1, 10 Sept 2014
----------------------------------------------------------------
Sample size for a 2-arm 1-stage trial with time-to-event outcome
based on Royston et al. (2011) Trials 12:81
----------------------------------------------------------------

Median survival time: 128.3 time units

Operating characteristics
------------------------------------------------------
Alpha(1S)     Power     HR|H0     HR|H1 Crit. HR Duration
------------------------------------------------------
    0.013     0.901     1.000     0.750    0.836   97.750
------------------------------------------------------
Patient accrual stopped at time 54.000

Duration is expressed in one month periods and assumes
survival times are exponentially distributed

Sample size and number of events
------------------------------------
           Overall  Control   Exper.
------------------------------------
Arms             2        1        1
Acc. rate       37       19       19
Patients      1998      999      999
Events         563      315      248
------------------------------------
```

7.8 ADDENDUM 3 - ESTIMATION OF CONTROL ARM EVENT ACCRUAL FOR M0 PATIENTS, ASSUMING LINEAR INCREASE BASED ON OBSERVED QUARTERLY ACCRUAL IN 2018

As of the July 2019 data extraction, there were 135+87=222 MFS events in the combined G+J control arms. To account for the lag in reporting that is typically seen between a data extraction and return of the progression CRFs from sites, we add 16 additional events to the total of 222 observed leading to a total of 238 by Q3 2019.

Event forecast assuming rate of MFS event accrual continues as per 2018 for both comparisons (5 per quarter in A-G comparison; 6 per quarter in A-J comparison; total = 11 events per quarter). We anticipate reaching 315 events in the control arm of the combined comparisons at the end of Q2 2021. Therefore, we propose to close follow-up of both A-G and A-J comparisons at the end of June 2021. We would require 3 months for data cleaning and analysis with reporting in Q4 2021. Further close out funding would be required to complete the FSR and EuRACT upload by June 2022.

	quarter	quarte~y	tot_ev~s	
1.	1	Q3 2019	238	
2.	2	Q4 2019	249	
3.	3	Q1 2020	260	
4.	4	Q2 2020	271	
5.	5	Q3 2020	282	
6.	6	Q4 2020	293	
7.	7	Q1 2021	304	
8.	8	Q2 2021	315	A-G and A-J comparisons closes follow-up
9.	9	Q3 2021	321	
10	10	Q4 2021	327	
11	11	Q1 2022	333	
12	12	Q2 2022	339	

DocuSign Envelope ID: E1B1DB5B-0530-47B0-A2CB-3A7081E06B73

7 SIGNATURES OF APPROVAL

Date: 30-July-2021

Version: 3.0

Signatures

Name	Trial Role	Signature	Date
Nick James	Chief Investigator* (DocuSigned by: Nicholas James)		
Gert Attard	CCI "Enzalutamide + abiraterone comparison" (DocuSigned by: Gert Attard)		
Louise Brown	Project Lead (DocuSigned by: Louise Brown)		
Adrian Cook	Senior statistician (DocuSigned by: Adrian Cook)		
Laura Murphy	Trial statistician (DocuSigned by: Laura Murphy)		
Max Parmar	CTU Director and Programme Lead		

*On behalf of the STAMPEDE Trial Management Group

Credits: Other statisticians involved in development of this SAP have been Chris Brawley, Rachael Jinks, Gordana Jovic and Melissa Spears

Reviewers: Ian White (Apr-2018), Babak Oskooei-Choodari (Apr-2018)

STAMPEDE

Systemic Therapy in Advancing or Metastatic Prostate Cancer: Evaluation of Drug Efficacy

A multi-arm multi-stage randomised controlled trial

Version: 21.0
Date: 20 October 2020

**MRC CTU AT UCL ID:**	**PR08**
**ISRCTN #:**	**ISRCTN78818544**
**NCT #:**	**NCT00268476**
**EUDRACT #:**	**2004-000193-31**
**CTA #:**	**20363/0404/001**
**MREC #:**	**04/MRE07/35**

Authorised by:

**Name:**	**Professor Nicholas D James**
**Role:**	**Chief Investigator & Comparison CI for "Abiraterone comparison"**
**Signature:**	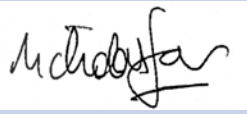

**Name:**	**Professor Louise Brown**
**Role:**	**Trial Statistician**
**Signature:**	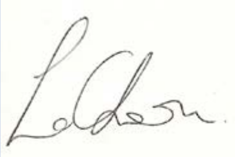

Authorised by:

Name: **PROFESSOR CHRIS PARKER**
Role: **CCI “M1|RT COMPARISON”**
Signature:

Name: **PROFESSOR GERTHARDT ATTARD**
Role: **CCI “ENZALUTAMIDE & ABIRATERONE COMPARISON”**
Signature:

Name: **PROFESSOR NOEL CLARKE**
Role: **CO-CCI "METFORMIN COMPARISON"**
Signature:

Name: **PROFESSOR SILKE GILLESSEN**
Role: **CO-CCI "METFORMIN COMPARISON"**
Signature:

Name: **PROFESSOR RUTH LANGLEY**
Role: **CCI "TRANSDERMAL OESTRADIOL COMPARISON"**
Signature:

GENERAL INFORMATION

This document was constructed using the MRC CTU at UCL Protocol Template Version 4.0. It describes the STAMPEDE trial, coordinated by the Medical Research Council (MRC) Clinical Trials Unit (CTU) at University College London (UCL), and provides information about procedures for entering patients into it. The protocol should not be used as an aide-memoire or guide for the treatment of other patients. Every care has been taken in drafting this protocol, but corrections or amendments may be necessary. These will be circulated to the registered investigators in the trial, but sites entering patients for the first time are advised to contact the Cancer And Other Non-Infectious Diseases Group, MRC CTU at UCL, London, UK, to confirm they have the most up-to-date version.

COMPLIANCE

The trial will be conducted in compliance with the approved protocol, the Declaration of Helsinki 1996, the principles of Good Clinical Practice (GCP), Commission Directive 2005/28/EC with the implementation in national legislation in the UK by Statutory Instrument 2004/1031 and subsequent amendments, the UK Data Protection Act (DPA number: Z6364106), and the National Health Service (NHS) Research Governance Framework for Health and Social Care (RGF).

*Until the Clinical Trials Regulation EU No 536/2014 becomes applicable, the trial will be conducted in accordance with the Clinical Trials Directive as implemented in the UK statutory instrument. When the directive is repealed on the day of entry into application of the Clinical Trial Regulation the trial will work towards implementation of the Regulation (536/2014) following any transition period.

International sites will comply with the principles of GCP as laid down by the ICH topic E6 (Note for Guidance on GCP), Commission Directive 2005/28/EC (the European Directive 2001/20/EC [where applicable]) and applicable national regulations.

SPONSOR

UCL is the sponsor of STAMPEDE and MRC CTU has been delegated responsibility for the overall management of STAMPEDE. Queries relating to UCL sponsorship should be addressed to the Director, Professor Max Parmar, Institute of Clinical Trials & Methodology, MRC CTU at UCL, 2nd Floor, 90 High Holborn, London, WC1V 6LJ UK, or via the STAMPEDE Trial Team.

FUNDING

Cancer Research UK's Clinical Research Committee (formerly the Clinical Trials Advisory Awards Committee), Medical Research Council, and educational grants from Novartis, Sanofi-Aventis, Pfizer, Janssen Pharma NV, Astellas, Clovis Oncology.

AUTHORISATIONS AND APPROVALS

The following persons are authorised to sign the final protocol and protocol amendments for the sponsor: Chief Investigator and Trial Statistician and the Co-Chief-investigators for each comparison subsequently added.

TRIAL REGISTRATION

This trial has been registered with the ClinicalTrials.gov Clinical Trials Register, where it is identified as NCT00268476.

RANDOMISATION
Call MRC CTU at UCL, Monday to Friday 0900-1700
Excluding public holidays or dates when notice has been given by the Unit.
Tel: +44 (0) 20 7670 4777

SAE REPORTING
Fax to 020 7670 4818 within 24 hours of becoming aware of the event
Or send via **encrypted** email to mrcctu.stampede@ucl.ac.uk

COORDINATING SITE

MRC Clinical Trials Unit at UCL
90 High Holborn
2nd Floor
London WC1V 6LJ
UK

Switchboard:	020 7670 4700
Fax:	020 7670 4818
Email:	mrcctu.stampede@ucl.ac.uk
Website:	www.stampedetrial.org

MRC CTU AT UCL STAFF

Trial Manager:	Michelle Buckner	Tel:	0207 670 4840
Trial Manager:	Sarah Jackson	Tel:	0207 670 4674
Trial Manager	Dymphna Lee	Tel:	0207 670 4916
Trial Manager	Malissa Richmond	Tel:	0207 670 4910
Trial Manager:	Christopher Wanstall	Tel:	0207 670 4882
Trial Manager	Melanie Weiss	Tel:	0207 670 4843
Clinical Project Manager	Claire Amos	Tel:	0207 670 4771
Clinical Project Manager	Nafisah B Atako	Tel:	0207 670 4896
Clinical Project Manager	Cheryl Pugh	Tel:	0207 670 4935

FOR ALL OTHER CONTACTS: http://www.stampedetrial.org/contact-us/trial-team/

SAAK COORDINATING CENTRE

Zuzanna Maniecka   Bern, Switzerland

CHIEF INVESTIGATOR

Prof Nicholas James

Prostate and Bladder Cancer Research Team Leader
The Institute of Cancer Research,
237 Fulham Road London, SW3 6JB
UK

Tel: 020 7153 5130 (PA)

Email: nick.james@icr.ac.uk

CO-INVESTIGATORS AND TMG MEMBERS

Prof Gerhardt Attard<br>Oncologist	London, UK	CCI "Enzalutamide+Abiraterone comparison"
Dr Simon Chowdhury<br>Oncologist	London, UK	
Prof Noel Clarke<br>Urologist	Manchester, UK	TMG Vice-Chair<br>Co-CCI "Metformin comparison"
Mr William Cross<br>Urologist	Leeds, UK	
Prof David Dearnaley<br>Oncologist	London, UK	
Dr Duncan Gilbert<br>Oncologist	London & Brighton, UK	
Prof Silke Gillessen<br>Oncologist	St Gallen, Switzerland	Co-CCI "Metformin comparison"
Prof Rob Jones<br>Oncologist	Glasgow, UK	
Prof Ruth Langley<br>Oncologist	London & Brighton, UK	CCI "Transdermal oestradiol comparison"
Dr Zafar Malik<br>Oncologist	Liverpool, UK	
Prof Chris Parker<br>Oncologist	London, UK	CCI "M1|RT comparison"
Dr Martin Russell<br>Oncologist	Glasgow, UK	
Robin Millman<br>Patient representative	UK	
David Matheson,<br>Patient representative	UK	
Prof Mark Sculpher<br>Health Economics Advisor	York, UK	
Rachel Hunter<br>PRIMENT UCL	London, UK	
Dr Amanda Adler<br>Metformin adviser	Cambridge UK	
Dr Hannah Rush<br>Clinical Research Fellow	London, UK	

Dr Archie Macnair<br>Clinical Research Fellow	London, UK

TRIAL ADVISORY GROUPS	CHAIRS
Biological Research Group<br>STRATOSPHere consortium	Prof Gerhardt Attard
Bone & Imaging Group	Prof Noel Clarke
Clinical Safety Committee	Prof Noel Clarke
Comparison Management Groups	Each led by Comparison Chief Investigator(s)
Metabolic Translational Group	Prof Silke Gillessen
Outcome Review Group	Prof Robert Jones
Site Advisory Team	Mr William Cross
Quality of Life Group	Prof Nicholas James

CONTENTS

GENERAL INFORMATION 3

CONTENTS 7

SUMMARY OF TRIAL 11

ABBREVIATIONS & GLOSSARY 19

2.1 INTRODUCTION AND SETTING 28
2.1.1 Long-term Androgen Deprivation Therapy 28
2.1.2 Role Of SOC Radiotherapy 28
2.1.3 Role Of SOC systemic therapy: docetaxel, abiraterone, enzalutamide or apalutamide in addition to ADT 29
2.2 DESIGN 30
2.3 PREVIOUSLY-REPORTED RESEARCH TREATMENTS 30
2.4 COMPARISONS IN FOLLOW-UP 31
2.5 RATIONALE FOR RECRUITING COMPARISONS 31
2.5.1 Metformin 31
2.5.2 Transdermal Oestradiol 32
3.1 SITE/INVESTIGATOR CRITERIA 34
3.1.1 Principle Investigator's Qualifications & Agreements 34
3.1.2 Adequate Resources 35
3.2 COMPARISON-SPECIFIC SITE ACCREDITATION 35
3.2.1 Transdermal Oestradiol Comparison: arms A & L 35
3.3 REQUIRED TRIAL DOCUMENTATION 36
4.1 IDENTIFYING POTENTIAL TRIAL PARTICIPANTS 37
4.2 APPROACH TO INFORMED CONSENT 37
4.2.1 Screening Investigations Prior To Randomisation 37
4.2.2 Baseline Investigations required for participants allocated to arms A, K, L 38
4.3 PRIOR PERMITTED SOC TREATMENTS 38
4.3.1 Hormone Treatment Prior To Randomisation 38
4.3.2 Standard-Of-Care (SOC) Radiotherapy 39
4.3.3 Standard-Of-Care (SOC) Systemic Therapy; docetaxel, abiraterone, enzalutamide or apalutamide 39
4.4 GENERAL INCLUSION CRITERIA – DISEASE CATEGORIES 40
4.4.1 High-Risk Newly-Diagnosed Non-Metastatic Node-Negative (N0/Nx) Disease 40
4.4.2 Newly-Diagnosed Metastatic Or Node-Positive Disease 40
4.4.3 Previously Radically Treated, Now Relapsing (Prior Radical Surgery And/or Radiotherapy) .. 40
4.5 GENERAL INCLUSION CRITERIA REQUIRED FOR ALL PARTICIPANTS 40
4.6 GENERAL EXCLUSION CRITERIA 41
4.7 COMPARISON-SPECIFIC ELIGIBILITY CRITERIA 42
4.7.1 Metformin Comparison (randomisation between arm A and arm K) 42
4.7.2 Transdermal Oestradiol Comparison (randomisation between arm A and arm L) 42
4.8 SUB-STUDY ELIGIBILITY CRITERIA 43
4.8.1 Eligibility for germline DNA sub-study (Saliva samples) 43
4.8.2 Eligibility for the circulating tumour DNA sub-study (sequential blood samples) 43
4.8.3 Eligibility for tumour sample analysis (FFPE blocks) 43
4.8.4 Eligibility for metformin metabolic sub-study 43
5.1 RANDOMISATION 44
5.2 CO-ENROLMENT GUIDELINES 44

6.1 STANDARD-OF-CARE (SOC) 46
6.1.1 Androgen Deprivation Therapy 46
6.1.2 SOC Radiotherapy to prostate 47
6.1.3 Administration of SOC RT 47
6.1.4 SOC upfront systemic therapy: docetaxel, abiraterone, enzalutamide or apalutamide 48
6.2 RESEARCH TREATMENTS 49
6.2.1 Treatment Duration 49
6.2.2 Research Abiraterone + Prednisolone (relevant to Arms G & J) 51
6.2.3 Research Enzalutamide (Arm J) 60
6.2.4 Research Metformin (Arm K) 63
6.2.5 Research Transdermal Oestradiol (Arm L) 68
6.3 CONCOMITANT TREATMENTS 71
6.4 TRIAL PRODUCTS 71
6.5 TREATMENT DATA COLLECTION 71
6.6 MEASURES OF COMPLIANCE/ADHERENCE 71
7.1 SCHEDULE FOR ASSESSMENTS 73
7.1.1 Follow-up Schedules 73
7.1.2 PSA, Testosterone And Oestradiol Measurements 73
7.1.3 Assessment Of Treatment Failure (Definition Of Progression) 73
7.1.4 Additional Metabolic And Cardiovascular Outcomes 75
7.1.5 Additional Safety Assessments 76
7.2 DATA COLLECTION PROCEDURES 77
7.2.1 Data Collection For SOC Hormone Therapy 77
7.2.2 Data Collection For SOC Systemic therapy: docetaxel, abiraterone, enzalutamide, or apalutamide 77
7.2.3 Data Collection And Non-Administration Of Standard Radiotherapy 78
7.2.4 Data Collection For Palliative Radiotherapy 78
7.2.5 Data Collection for Research (M1) Radiotherapy 78
7.2.6 Data Collection for Additional Treatments Given for Disease Progression 78
7.3 FOLLOW-UP PROCEDURE 79
7.3.1 Follow-up Telephone Consultations 79
7.3.2 Follow-up Using Electronic Healthcare Records 79
7.4 TRIAL & COMPARISON CLOSURE 80
7.4.1 Comparisons for which Follow-up has Ceased 80
8.1 STOPPING RESEARCH INTERVENTIONS 84
8.1.1 Stopping Research Treatment: Abiraterone, Enzalutamide + Abiraterone 84
8.1.2 Stopping Research Treatment: Metformin 84
8.1.3 Stopping Research Treatment: Transdermal Oestradiol 85
8.2 BREAKS IN SOC ADT 85
8.3 PARTICIPANT TRANSFERS 86
8.4 EARLY CESSATION OF TRIAL PARTICIPATION 86
9.1 METHOD OF RANDOMISATION 88
9.2 OUTCOME MEASURES 88
9.3 SAMPLE SIZE: PRINCIPLES 89
9.4 SAMPLE SIZE ISSUES & TRIAL STAGES: ADDITIONAL RESEARCH ARM H 92
9.4.1 Pilot Phase: Additional Research Arm H 92
9.4.2 Activity Stages I-III: Additional Research Arm H 92
9.4.3 Efficacy Stage IV: Additional Research Arm H 92
9.4.4 Sample Size For Additional Research Arm H 92
9.5 SAMPLE SIZE ISSUES & TRIAL STAGES: ADDITIONAL RESEARCH ARM J 94
9.5.1 Pilot Phase: Additional Research Arm J 94
9.5.2 Activity Stages I-II: Additional Research Arm J 94

9.5.3 Efficacy Stage III: Additional Research Arm J 94
9.5.4 Sample Size For Additional Research Arm J 95
9.5.5 Update to Efficacy Stage Analysis For Additional Research Arm J 95
9.6 SAMPLE SIZE ISSUES & TRIAL STAGES: ADDITIONAL RESEARCH ARM K 96
9.6.1 Implementation: Additional Research Arm K 96
9.6.2 Outcome Measures: Additional Research Arm K 97
9.6.3 Pilot Phase: Additional Research Arm K 98
9.6.4 Activity Stage I: Additional Research Arm K 98
9.6.5 Efficacy Stage II: Additional Research Arm K 98
9.6.6 Sample Size For Additional Research Arm K 99
9.6.7 Further Sample Size Issues For Additional Research Arm K 99
9.7 SAMPLE SIZE ISSUES & TRIAL STAGES: ADDITIONAL RESEARCH ARM L 100
9.7.1 Implementation And Outcome Measures: Additional Research Arm L 100
9.7.2 Additional Use of Outcome Data from the “transdermal oestradiol comparison” 101
9.7.3 Definition of PFS and Use As Co-primary Outcome Measure: Additional Research Arm L 101
9.7.4 Abiraterone/Enzalutamide/Apalutamide enhanced safety monitoring 102
9.8 FURTHER NOTES ON TRIAL DESIGN 102
9.8.1 Overall Sample Size 102
9.8.2 Factorial Design 102
9.9 INTERIM MONITORING AND ANALYSES 103
9.10 OUTLINE ANALYSIS PLAN 103
9.10.1 Pilot / Safety Phases 104
9.10.2 Activity And Efficacy Stages 104
10.1 DATA MONITORING 105
10.1.1 Central Monitoring Of Consent 105
10.1.2 Central Monitoring Of Data 105
10.1.3 Direct Access to Patient Data 105
10.1.4 Monitoring Visits to Investigator Sites 105
10.2 CONFIDENTIALITY 106
11.1 SAFETY REPORTING DEFINITIONS 107
11.1.1 Adverse event definitions 108
11.1.2 Defining “treatment” for the purposes of safety reporting 108
11.2 SITE INVESTIGATOR RESPONSIBILITIES 109
11.2.1 Notification period 109
11.2.2 Trial-Specific “Expedited Reporting” Exemptions 110
11.2.3 Investigator Assessment 111
11.2.4 Event Follow-up 113
11.3 CTU RESPONSIBILITIES 114
11.3.1 Sponsor (CTU) Assessment 114
12.1 ETHICAL CONSIDERATIONS 116
12.2 ETHICAL APPROVAL 117
13.1 DATA COLLECTION & RETENTION 119
16.1 TRIAL MANAGEMENT GROUP (TMG) 122
16.2 TRIAL STEERING COMMITTEE (TSC) 122
16.3 INDEPENDENT DATA MONITORING COMMITTEE (IDMC) 122
16.4 TMG SUB-GROUPS AND EXPERT PANELS 123
16.5 MRC CTU AT UCL INTERNAL GROUPS 123
17.1 PATIENT REPORTED OUTCOMES 125
17.1.1 Quality of life (QL) 125
17.1.2 Health Economics 125
17.2 TRANSLATIONAL SUB-STUDIES 127
17.2.1 Germline DNA Analysis 127

17.2.2 Circulating Tumour-DNA Analysis (Sequential Blood Samples) 127
17.2.3 Tissue Sample Analysis (FFPE Blocks) 127
17.2.4 Biomarker-Screening Pilot 128
17.2.5 Informed consent to receive results arising from genetic sub-studies 128
17.3 DISEASE VOLUMETRIC ANALYSIS SUB-STUDY 128
17.4 USING ROUTINE DATA TO IDENTIFY CLINICAL TRIAL OUTCOMES 128
17.5 METFORMIN METABOLIC SUBSTUDY 129
18.1 POTENTIAL IMPACT OF PPI 130
18.2 PATIENT REPRESENTATIVES 130
21.1 PROTOCOL 133
21.1.1 Amendments Made To Protocol Version 1.0 (May-2004) 133
21.1.2 Amendments Made To Protocol Version 1.1 (May-2005) 133
21.1.3 Amendments Made To Protocol Version 2.0 (Jun-2005) 133
21.1.4 Amendments Made To Protocol Version 3.0 (Jul-2006) 134
21.1.5 Amendments Made To Protocol Version 4.0 (Dec-2007) 135
21.1.6 Amendments Made To Protocol Version 5.0 (Aug-2008) 135
21.1.7 Amendments Made To Protocol Version 6.0 (Jul-2009) 136
21.1.8 Amendments Made To Protocol Version 7.0 (Jul--2011) 136
21.1.9 Amendments Made To Protocol Version 7.1 (Jul-2011) 137
21.1.10 Amendments Made To Protocol Version 8.0 (Sep-2011) 138
21.1.11 Amendments Made To Protocol Version 9.0 (Oct-2012) 139
21.1.12 Amendments Made To Protocol Version 10.0 (Apr-2013) 139
21.1.13 Amendments Made To Protocol Version 11.0 (Sep-2013) 140
21.1.14 Amendments Made To Protocol Version 12.0 (Jan-2014) 140
21.1.15 Amendments Made To Protocol Version 13.0 (Feb-2015) 140
21.1.16 Amendments Made To Protocol Version 14.0 (Oct-2015) 141
21.1.17 Amendments Made To Protocol Version 15.0 (Mar-2017) 142
21.1.18 Amendments Made To Protocol Version 16.0 (Oct-2017) 142
21.1.19 Amendments Made To Protocol Version 17.0 (Feb-2017) 143
21.1.20 Amendments made to Protocol Version 18.0 (Jun-2018) 144
21.1.21 Amendments made to Protocol Version 19.0 (Aug-2020) 145
21.1.22 Amendments made to Protocol Version 20.0 (Oct-2020) 147

SUMMARY OF TRIAL

SUMMARY INFORMATION TYPE	SUMMARY DETAILS
Acronym	STAMPEDE
Long Title of Trial	Systemic Therapy in Advancing or Metastatic Prostate Cancer: Evaluation of Drug Efficacy: A multi-arm multi-stage randomised controlled trial
Version	21.0
Date	20-October-2020
MRC CTU at UCL ID	PR08
NCT #	NCT00268476
EudraCT #	2004-000193-31
Study Design	Multi-arm multi-stage platform randomised controlled trial
Type of Participants to be Studied	People starting long-term hormone therapy for metastatic or high-risk non-metastatic prostate cancer
Setting	Tertiary care
Interventions to be Compared	Various - see comparison-specific tables
Study Hypothesis	Various - see comparison-specific tables
Definitive Primary Outcome Measure	Overall survival (unless stated)
Intermediate Primary Outcome Measure	Failure-free survival (unless stated)
Secondary Outcome Measure(s)	Toxicity<br>Symptomatic skeletal events<br>Quality-of-life<br>Cost-effectiveness
Randomisation	Minimisation using a random element across a number of stratification factors
Number of Participants	See comparison-specific tables
Duration	See comparison-specific tables
Sponsor	University College London
Funders	Cancer Research UK<br>Medical Research Council<br>Astellas<br>Clovis Oncology<br>Janssen<br>Novartis<br>Pfizer<br>Sanofi-Aventis

SUMMARY INFORMATION TYPE	SUMMARY DETAILS
"Original comparisons"	
Type of Participants to be Studied	People starting long-term hormone therapy for metastatic or high-risk non-metastatic prostate cancer
Control Arm	• Arm A: Standard-of-care (SOC)
Interventions to be Compared	• Arm B: SOC + zoledronic acid<br>• Arm C: SOC + docetaxel<br>• Arm D: SOC + celecoxib<br>• Arm E: SOC + zoledronic acid + docetaxel<br>• Arm F: SOC + zoledronic acid + celecoxib
Allocation ratio	• 2 control arm : 1 research arm [2A:1B:1C:1D:1E:1F]
Study Hypothesis	Research interventions will improve survival over SOC
Definitive Primary Outcome Measure	Overall survival
Intermediate Primary Outcome Measure	Failure-free survival
Status	Primary results published and **active follow-up discontinued** Summer 2018 (1, 2)
"Abiraterone comparison"	
Type of Participants to be Studied	People starting long-term hormone therapy for metastatic or high-risk non-metastatic prostate cancer
Control arm	• Arm A: Standard-of-care (SOC)
Intervention to be Compared	• Arm G: SOC + abiraterone
Allocation ratio	• 1 control arm : 1 research arm [1A:1G]
Study Hypothesis	Addition of abiraterone to SOC will improve survival over SOC alone
Definitive Primary Outcome Measure	Overall survival
Intermediate Primary Outcome Measure	Failure-free survival
Number of Participants	Around 1,800 patients for 267 control arm definitive primary outcome measure events
Duration	6 to 8 years
Status	Primary results published (3), remains on active follow-up to permit a further longer-term analysis
"M1|RT comparison"	
Type of Participants to be Studied	People starting long-term hormone therapy for newly-diagnosed metastatic prostate cancer with no contraindication to prostate radiotherapy
Control arm	• Arm A: Standard-of-care (SOC)
Intervention to be Compared	• Arm H: SOC + radiotherapy to the prostate (RT)
Allocation ratio	• 1 control arm : 1 research arm [1A:1H]
Study Hypothesis	Addition of RT to SOC will improve survival over SOC alone

SUMMARY INFORMATION TYPE	SUMMARY DETAILS
Definitive Primary Outcome Measure	Overall survival
Intermediate Primary Outcome Measure	Failure-free survival
Number of Participants	Around 1,800 patients for 267 control arm definitive primary outcome measure events
Duration	6 to 8 years
Status	Primary results published (4) remains on active follow-up to permit a further longer-term analysis
"Enzalutamide + abiraterone comparison"	
Type of Participants	People starting long-term hormone therapy for metastatic or high-risk non-metastatic prostate cancer
Control Arm	• Arm A: Standard-of-care (SOC)
Interventions to be Compared	• Arm J: SOC + enzalutamide + abiraterone
Allocation ratio	• 1 control arm : 1 research arm [1A:1J]
Study Hypothesis	Addition of enzalutamide, in combination with abiraterone, to SOC will improve survival over SOC alone
Definitive Primary Outcome Measure	Overall survival
Intermediate Primary Outcome Measure	Failure-free survival
Number of Participants	Around 1,800 patients for 267 control arm definitive primary outcome measure events
Duration	6 to 8 years
Status	In follow-up
"Metformin comparison"	
Type of Participants to be Studied	Non-diabetic people, with no contraindication to metformin, starting long-term hormone therapy for metastatic or high-risk non-metastatic prostate cancer
Control arm	• Arm A: Standard-of-care (SOC)
Intervention to be Compared	• Arm K: SOC + metformin
Allocation ratio	• 1 control arm : 1 research arm [1A:1K]
Study Hypothesis	Addition of metformin to SOC will improve survival over SOC alone
Definitive Primary Outcome Measure	Overall survival
Intermediate Primary Outcome Measure	Overall survival
Number of Participants	Around 2800 patients, including around 1,700 M1 (metastatic) patients, for 473 control arm definitive primary outcome measure events among M1 patients
Duration	7 years

SUMMARY INFORMATION TYPE	SUMMARY DETAILS
Status	Recruiting
"Transdermal oestradiol comparison"	
Type of Participants to be Studied	People starting long-term hormone therapy for metastatic or high-risk non-metastatic prostate cancer, having had no more than one 4-week (or one-month) LHRH (Luteinizing hormone releasing hormone) injection & 8 weeks of anti-androgens
Control arm	• Arm A: Standard-of-care (SOC)
Intervention to be Compared	• Arm L: Transdermal oestradiol ± RT ± docetaxel/abiraterone/enzalutamide/apalutamide
Allocation ratio	• 1 control arm : 1 research arm [1A:1L]
Study Hypothesis	Transdermal oestradiol will be non-inferior to standard hormone therapy, while having fewer side-effects and improved quality-of-life
Definitive Primary Outcome Measures	Co-primary endpoints of progression-free survival and overall survival
Intermediate Primary Outcome Measure	Progression-free survival
Number of Participants	Around 700 to include within a meta-analysis with the PATCH trial (EudraCT 2005-001030-33), which will include around 2,500 patients overall
Duration	4 to 6 years
Status	Recruiting

Figure 1: Randomisation schema from protocol v21.0 onwards

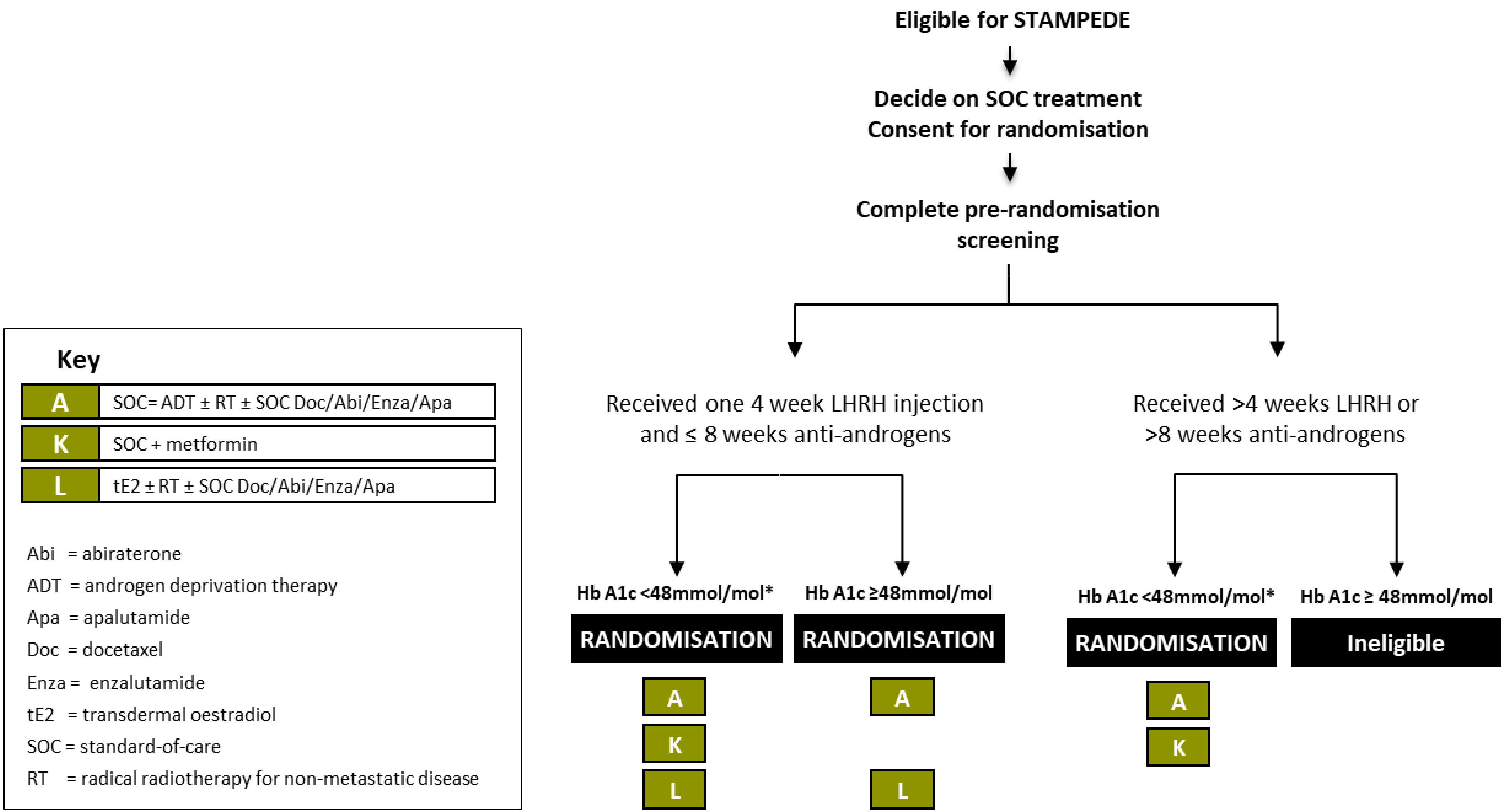

*Participants must not have received any treatment with any anti-diabetes medication but diet controlled diabetes is allowed if HbA1c now in limits.

Figure 2: Arms of the STAMPEDE trial open to recruitment over time

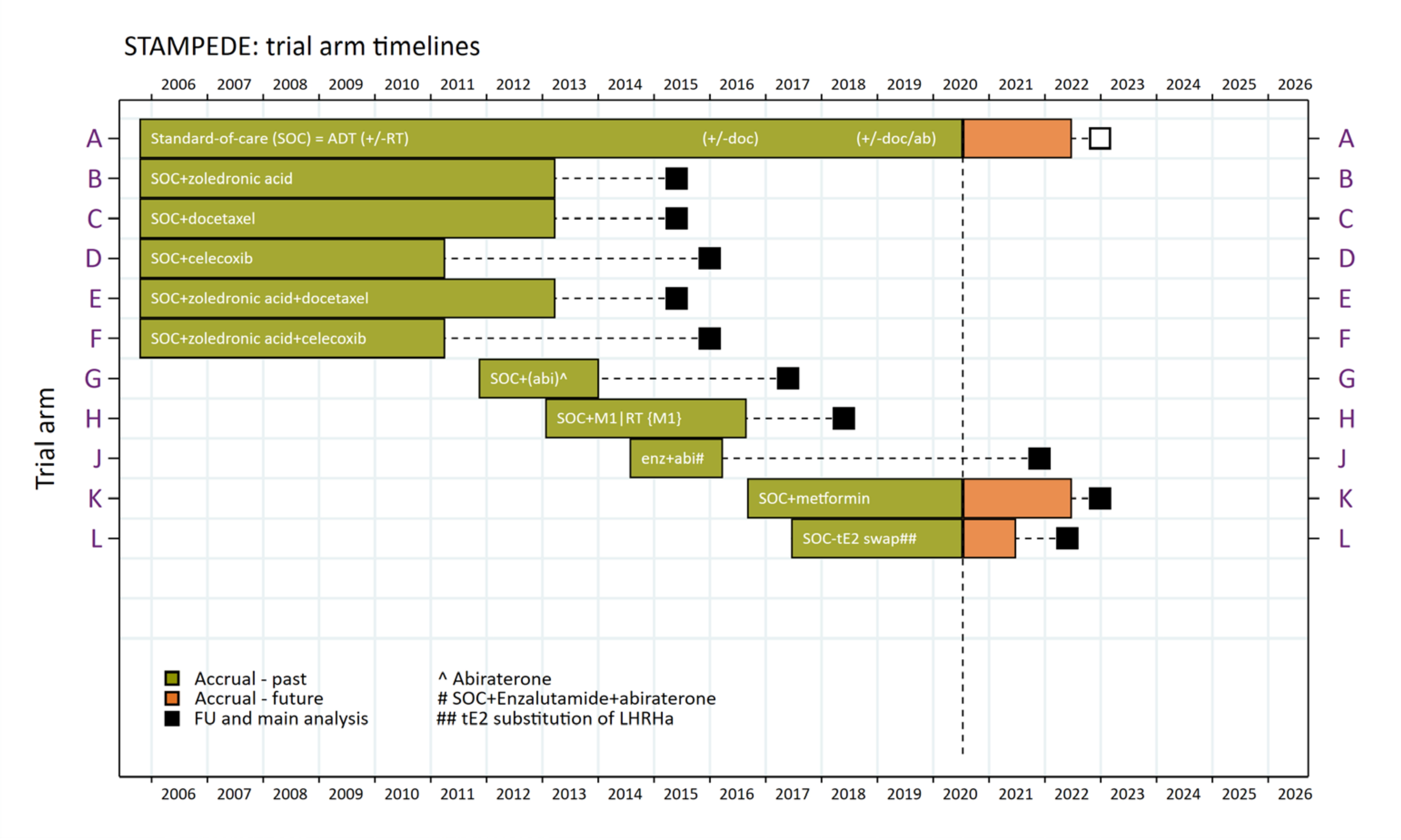

Table 1: Schedule of Assessments for Participants Randomised before 05-Sep-2016

	ASSESSMENT WEEK											ALL FURTHER VISITS [1]	AT EACH DISEASE EVENT [2]	END OF TRT	PRIOR TO 2ND LINE TRT
	4-6	12	18	24	36	48	60	72	84	96	104				
**Arm A/G/J**															
Blood collection cell-free DNA Streck™ tubes [3]						X [4]		X [4]	X [4]				X	X	X
Saliva sample [3]	Any time point														
FFPE block [3]	Once, at the point of request														
PSA	X	X	X	X	X	X	X	X	X	X	X	X			
Waist circumference + Weight	X	X	X	X	X	X	X	X	X	X	X	X			
Concomitant medication	X	X	X	X	X	X	X	X	X	X	X	X			
QL + HE [5,3]	X	X	X	X	X	X	X	X	X	X	X	X			
**Arm G&J only**															
Blood pressure[6]	X	X	X	X	X	X	X	X	X	X	X	X			
Safety bloods (LFTs and potassium) [6]	X	X	X	X	X	X	X	X	X	X	X	X			

[1] Follow-up visits after year 2 need to be carried out every 6 months for the first 5 years. At year 6 and onwards visits should be every 12 months whilst active follow-up continues.

[2] Disease events are defined as each type of disease progression: PSA (biochemical), clinical (symptomatic) and radiological (objective).

[3] Only if participating in relevant sub-study, for information regarding samples see the **Sample Collection & Handling Manual** available via the STAMPEDE website for details

[4] Sample only required for participants with metastatic disease at trial entry (M1)

[5] Review **Table 38** for a breakdown of participants that are still required to complete Quality-of-life (QL) + health economic (HE) questionnaires

[6] For participants receiving research abiraterone, BP, liver function tests (LFTs) and serum potassium monitoring is required 2-weekly in the first 12 weeks, then monthly until 12 months on treatment. For participants who have not experienced Toxicity following 12 months of treatment, this may be reduced to every 2 months whilst research abiraterone continues. Arm J participants continuing on enzalutamide alone may reduce to 3 monthly BP monitoring, no requirement for ongoing safety blood tests. Increased monitoring is required in participants experiencing toxicity; see **Table 13**, **Table 14** and **Table 15** for details.

Table 2: Schedule of Assessments for Participants Randomised on or after 05-Sep-2016

	Pre-Rand^{n}	ASSESSMENT WEEK											ALL FURTHER VISITS[1]	AT EACH DISEASE EVENT[2]	END OF TRT	PRIOR TO 2ND LINE TRT
		4-6	12	18	24	36	48	60	72	84	96	104				
**Arms A/K/L**																
Cardiac (BP)	X															
Screening bloods[3]	X															
Full radiological screening[4]	X															
WHO PS	X															
Blood collection cell-free DNA Streck™ tubes[5]	X						X[3]		X[3]	X[3]				X	X	X
Saliva sample[5]	Any time point															
FFPE block[5]	At the point of request															
Waist circumference + Weight	X	X	X	X	X	X	X	X	X	X	X	X	X			
Height	X															
QL & HE[5, 6]	X	X	X	X	X	X	X	X	X	X	X	X	X			
HbA1c & Lipid profile[7]	X				X[8]		X[8]					X[8]	X[8]			
Glucose & Triglycerides	X				X[8]		X[8]					X[8]	X[8]			
PSA	X[9]	X	X	X	X	X	X	X	X	X	X	X	X			
Concomitant medication	X	X	X	X	X	X	X	X	X	X	X	X	X			
**Arm K only**																
Safety bloods (eGFR)[10]					X		X		X		X		X			
**Arm L only**																
Testosterone & Oestradiol[11]		X[12]	X		X		X		X		X		X			

[1] Follow-up visits after year 2 need to be carried out every 6 months for the first 5 years. At year 6 and onwards visits should be every 12 months whilst active follow-up continues.
[2] Disease events are defined as each type of disease progression: PSA (biochemical), clinical (symptomatic) and radiological (objective).
[3] U&Es, LFTs, Serum creatinine and FBCs to be completed before randomisation. Cholesterol, albumin, serum corrected calcium, phosphate, magnesium within 4 weeks before or after randomisation.
[4] Pre-randomisation imaging must be representative of current disease status, see **section 4.2.1**
[5] Only if participating in relevant sub-study, for information regarding samples see the **Sample Collection & Handling Manual** available via the STAMPEDE website for details
[6] Review **Table 38** for a breakdown of participants that are still required to complete Quality-of-life (QL) + health economic (HE) questionnaires
[7] HBA1c required prior to randomisation for participants being considered for "metformin comparison".
[8] If missed, samples can be obtained +/-12 weeks of the scheduled FU visit, maintaining 10-12 weeks in between the tests due at week 24 and 48 weeks.
[9] Pre-ADT PSA must have been obtained within 6 months prior to randomisation and another PSA analysis should be completed within 2 weeks of randomisation.
[10] Increased monitoring of renal function required if renal function declines see **Table 23**. To continue until metformin permanently stopped.
[11] Hormone tests are required whilst the participant is receiving research transdermal oestradiol. Note that additional tests may be necessary as detailed in **Section 6.2.5.B**.
[12] First hormone tests for patients receiving research transdermal oestradiol should be at 4 weeks.

ABBREVIATIONS & GLOSSARY

ABBREVIATION	EXPANSION
AA	Anti-androgen
ACE	Angiotensin-Converting Enzyme
ACTH	Adrenocorticotropic hormone
ADT	Androgen deprivation therapy
AE	Adverse Event
AR	Androgen receptor
AS	Activity Stage
AUC	Area under the plasma concentration–time curve
BID	Twice a day (bis in die)
BP	Blood pressure
BRCA2	BReast CAncer gene 2
BRG	Biological Research Group
BSA	Body surface area
CCI	Comparison Chief Investigator
CF	Consent Form
CI	Confidence interval
Co-CCI	Comparison Co-Chief Investigator
Cox-2	Cyclooxygenase 2
CRF	Case Report Form
CRN	Clinical Research Network
CRUK	Cancer Research UK
CRPC	Castrate-Resistant Prostate Cancer
CT	Computerised tomography
CTA	Clinical Trials Authorisation
CTAAC	Clinical Trials Advisory and Awards Committee
ctDNA	Circulating tumour DNA
CTC	Common Toxicity Criteria
CTU	Clinical Trials Unit
CTV	Clinical Tumour Volume
CVS	Cardiovascular
CXR	Chest X-ray
DAB	Dual Androgen Blockade

ABBREVIATION	EXPANSION
DHT	Dihydrotestosterone
DNA	Deoxyribonucleic Acid
DPA	Data Protection Act
ES	Efficacy Stage
IB	Investigator Brochure
ICH	International Conference on Harmonization
ECG	Electrocardiogram
FBC	Full Blood Count
FFS	Failure-Free Survival
FFPE	Formalin Fixed Paraffin Embedded
GCP	Good Clinical Practice
GFR	Glomerular Filtration Rate
GP	General Practitioner
HbA1c	Glycosylated haemoglobin
Hb	Haemoglobin
HE	Health Economics
HES	Hospital Episode Statistics
Hr	Hour
HR	Hazard Ratio
HRA	Health Research Authority
HSCIC	Health & Social Care Information Centre
HSPC	Hormone Sensitive Prostate Cancer
HT	Hormone Therapy
IDMC	Independent Data Monitoring Committee
IM	Intramuscular
IMRT	Intensity Modulated Radiation Therapy
INR	International Normalized Ratio
IR	Immediate-Release
ISRCTN	International Standard Randomised Controlled Trial Number
IU	International Units
IV	Intravenous
LFTs	Liver Function Tests
LHRH	Luteinising Hormone Releasing Hormone
LHRHa	Luteinising Hormone Releasing Hormone antagonist/agonist

ABBREVIATION	EXPANSION
LREC	Local Research Ethics Committee
m	Month
mcg	Microgram
MHRA	Medicine and Healthcare Products Regulatory Agency
min	Minutes
MRC	Medical Research Council
MREC	Main Research Ethics Committee
MRI	Magnetic resonance imaging
mTOR	Mammalian Target of Rapamycin
M0	Non-metastatic
M1	Metastatic
NCI	National Cancer Institute (USA)
NCRAS	National Cancer Registration and Analysis Service
NHS	National Health Service
N0	Node-negative
N+	Node-positive
NSAID	Non-Steroidal Anti-inflammatory Drugs
NYHA	New York Heart Association
OD	Once per day (omne in die)
ONS	Office for National Statistics
OS	Overall Survival
PATCH	Prostate Adenocarcinoma: TransCutaneous Hormones
PFS	Progression-free survival
PHE	Public Health England
PI	Principal Investigator
PIS	Patient Information Sheet
po	Orally (per orum)
PSA	Prostate Specific Antigen
pts	Patients
PTV	Planned Tumour Volume
QALY	Quality-adjusted Life Years
qds	Four times each day (quater die sumendus)
QL	Quality-of-life
RSI	Reference Safety Information
RTDS	National Radiotherapy Dataset

ABBREVIATION	EXPANSION
R&D	Research and Development
SACT	Systemic Anti-Cancer Therapy Dataset
SAE	Serious Adverse Event
SAR	Serious Adverse Reaction
sc	Under skin (sub-cutaneous)
SmPC	Summary of Product Characteristics
SOC	Standard-of-Care
SR	Sustained-Release
SSA	Site Specific Assessment
SUSAR	Suspected Unexpected Serious Adverse Reactions
SWOG	South West Oncology Group
tE2	Transdermal Oestradiol
TMG	Trial Management Group
TMT	Trial Management Team
TEAE	Treatment-emergent adverse event
TURP	Trans-Urethral Resection of Prostate
TSC	Trial Steering Committee
T2DM	Type 2 Diabetes Mellitus
UCL	University College London
ULN	Upper Limit of Normal
U+E	Urea and Electrolytes
WHO	World Health Organisation

TERM	DEFINITION
ADT	Androgen deprivation therapy given in the form of LHRH agonists/antagonists (abbreviated to LHRH) or alternatively, transdermal oestradiol.
Anti-androgens	Refers to 1st generation oral androgen receptor blockers including bicalutamide, flutamide and cyproterone. Please note that the use of cyproterone will impact on comparison-specific eligibility.
Comparison	In STAMPEDE many research treatments are evaluated and compared with participants receiving the current protocol standard-of-care. The term comparison describes the participants who have been randomised to receive research treatment and their comparable controls, each comparison is named by the research treatment e.g. the "metformin comparison" refers to all participants in arm K and the comparable non-diabetic patients contemporaneously randomised to arm A.
Hormone Therapy	Refers to all forms of hormone therapy given in the first line setting and includes LHRH, anti-androgens, transdermal oestradiol, GnRH agonists and antagonists. This term does not include novel AR-targeted agents such as abiraterone or enzalutamide.
PSA nadir	For trial purposes, this refers to the lowest PSA value detected between randomisation and week 24 on trial. This is used to derive the PSA progression value.
Protocol research treatment	Investigational Medicinal Products (IMPs) that are additional treatments participants allocated to research arms receive as part of the STAMPEDE protocol e.g. metformin for participants allocated to arm K, or alternative in the case of transdermal oestradiol for participants allocated to arm L.
Protocol standard-of-care (SOC) treatment	Standard forms of background treatment which are IMPs, permitted as part of the STAMPEDE protocol which include licenced ADT (e.g. LHRH analogues) given in the setting of hormone-naïve prostate cancer and first-line use of docetaxel, abiraterone, enzalutamide or apalutamide.
Non-protocol treatments	All prostate cancer treatments given following disease progression in the management of CRPC.
Prednisolone	In Swiss sites this may be referred to as prednisone.

1 LAY SUMMARY

STAMPEDE is a large clinical trial that aims to assess new treatment approaches for people affected by high-risk prostate cancer. The trial has been open since 2005 and has tested many different ways of treating prostate cancer and some results are now already known. Each new or alternative treatment is compared with the current standard approach, referred to as a “comparison”. More than 11,000 people have joined STAMPEDE so far with answers becoming available throughout the trial as information on life expectancy and disease control rates are gathered and compared.

New participants joining the trial from protocol v21.0 onwards may be eligible to join one of two treatment comparisons:

- The “metformin comparison” made between the control arm (arm A) who receive standard treatment only and the metformin treatment group (arm K) who receive standard treatment and metformin. **Note: randomisation is open to only a select number of sites participating in the metabolic substudy.**
- The “transdermal oestradiol comparison” made between the control arm (arm A) and the transdermal oestradiol treatment group (arm L) who receive transdermal oestradiol as an alternative form of standard hormone treatment.

Eligibility for each treatment group is dependent on several factors including the stage of prostate cancer, whether it has spread to involve other parts of the body (metastatic), and how long a patient has received hormone therapy prior to joining STAMPEDE. A computer program will be used to randomly allocate participants between all treatment groups for which they are eligible. **Table 3** summarises which treatment arms are currently open to recruitment.

Trial participants are asked whether they would like to join certain sub-studies being run alongside the trial. These aim to address several additional research questions such as what effect each treatment has on quality-of-life (QL), and which provides the greater value for money for the health service. Some sub-studies are focused on improving our understanding of the biology of prostate cancer. For example, can genetic changes be identified in prostate cancer cells that could predict which treatments might work best and may explain why some treatments stop working?

Table 3: Summary of treatment groups currently open to recruitment (Protocol version 21.0)

TREATMENT BEING TESTED	TREATMENT GROUP	SUMMARY	FROM PROTOCOL VERSION
Metformin	Arm K	This anti-diabetic medication is proposed to have anti-cancer effects and may help prevent the side-effects of long-term ADT. STAMPEDE will investigate whether adding metformin to the current standard-of-care for non-diabetic people can improve life expectancy.<br>Please note from protocol v21 onwards only sites participating in the metabolic sub-study can recruit to Arm K.	15.0
Transdermal oestradiol	Arm L	This is a form of hormone treatment which can suppress testosterone as effectively as standard forms of androgen-deprivation therapy (ADT) and has been shown to avoid some of the side-effects. For example, treatment with transdermal oestradiol does not appear to cause the bone to thin, a common problem with standard forms of ADT which might lead to the bones becoming fragile (osteoporosis) and more likely to break. It may also help to avoid some of the side-effects and therefore improve overall quality of life compared with standard forms of ADT. STAMPEDE will investigate whether transdermal oestradiol can treat prostate cancer as well as current standard forms of ADT. Transdermal oestradiol is currently being tested in another large clinical trial called PATCH which already has over 1,400 men participating.	16.0

Further results are expected in the next few years from other treatments tested in STAMPEDE, which have completed recruitment, summarised in **Table 4.**

Table 4: Summary of treatment groups closed to recruitment; results awaited but follow-up ongoing

TREATMENT BEING TESTED	TREATMENT GROUP	SUMMARY	FROM PROTOCOL VERSION
Abiraterone and Enzalutamide combination	Arm J	Enzalutamide is another novel hormone treatment, similar to abiraterone, which is also used in advanced prostate cancer, when standard hormone therapy has stopped working. Enzalutamide works by blocking androgen receptors and this may complement abiraterone. STAMPEDE is testing whether this treatment combination is a more effective way of controlling prostate cancer growth for longer and improving life expectancy.	12.0

Abiraterone was tested alone in arm G and the primary results of this comparison have been presented. Follow-up is ongoing as a further longer term analysis is planned.

Table 5: Summary of treatment group for which primary results reported but follow-up ongoing

TREATMENT TESTED	TREATMENT GROUP	SUMMARY OF RATIONALE AND RESULTS	PROTOCOL VERSION ADDED
Abiraterone	Arm G	This is a novel hormone treatment which works by inhibiting steroid hormone synthesis so blocks prostate cancer cells from generating their own male hormones. This is thought to be a major way in which prostate cancer cells resume growth following anti-hormonal therapies. The results of STAMPEDE have shown that the addition of abiraterone with prednisone improves life expectancy and disease control or relapse rates when used earlier, for people with locally-advanced or metastatic disease.	8.0
Prostate radiotherapy	Arm H	This is treatment with high-energy x-rays targeted to the prostate gland. This treatment is now mandatory within STAMPEDE for participants with cancer that is confined to the prostate gland as large trials have shown it improves life expectancy. The results from the primary analysis of the Arm H comparison demonstrate RT to the prostate prolonged overall survival in patients with oligometastatic (low burden) prostate cancer. Prostate RT did not provide any survival benefit to patients with high burden metastatic disease.	9.0

In the past STAMPEDE also tested whether adding docetaxel chemotherapy, zoledronic acid, or celecoxib, alone or in combination, and radiotherapy to prostate in M1 patients was beneficial in controlling prostate cancer growth and improving life expectancy. Recruitment has been completed to all of these original treatment groups, the results have been presented and it is no longer necessary to provide follow-up information relating to participants allocated to these comparisons, see **Table 6**.

Table 6: Summary of treatment groups reported and no longer on active follow-up

TREATMENT TESTED	TREATMENT GROUP	SUMMARY OF RATIONALE AND RESULTS	PROTOCOL VERSION ADDED
Zoledronic acid	Arm B	Prostate cancer cells can spread to bones and weaken them. Zoledronic acid is a drug that reduces bone destruction and hardens bones.<br>The results of STAMPEDE show that the addition of zoledronic acid alone does not prolong life expectancy. These results were comparable with data from other similar trials.	1.0
Docetaxel	Arm C	Docetaxel is a type of chemotherapy which can stop cells replicating. It has been used to treat advanced prostate cancer for some time, and is also used in e.g. the treatment of lung, breast and ovarian cancer.<br>The results of STAMPEDE show that the addition of docetaxel to hormone treatment does improve life expectancy, most markedly in people with metastatic disease, and delays time to progression or relapse for people with locally-advanced and metastatic disease.<br>The results of STAMPEDE were combined with other similar trials testing docetaxel and together, the results support this effect.<br>Docetaxel may now be given as part of standard treatment to all suitable people entering STAMPEDE (from protocol v14.0).	1.0
Celecoxib	Arm D	Celecoxib is an aspirin-like drug that is used to treat arthritis. It slows down the growth of cancer cells in the laboratory. STAMPEDE tested whether the addition of celecoxib could delay the growth of prostate cancer cells. Recruitment stopped early as an earlier analysis failed to demonstrate sufficient benefit. The final results were presented at GU ASCO 2016, a major international congress, and show that alone, celecoxib does not improve life expectancy.	1.0
Docetaxel and zoledronic acid combination	Arm E	The combination of these two medications did not offer any benefit to overall survival compared to the docetaxel alone.<br>Currently we do not recommend this combination as treatment for HSPC in STAMPEDE	1.0
Zoledronic and celecoxib	Arm F	The combination of these two medications did not improve overall survival in all patients randomised to this comparison. However, there was a small effect seen in patients with metastatic disease.<br>Currently we do not recommend this combination as treatment for HSPC in STAMPEDE	1.0

For further information relevant to these treatment groups, refer to the STAMPEDE website where you can see earlier versions of the protocol and find summaries of the results and links to the scientific publications, www.stampedetrial.org.

2 BACKGROUND

2.1 INTRODUCTION AND SETTING

Prostate cancer is a major health problem world-wide and accounts for nearly one fifth of all newly-diagnosed male cancers. In the UK, approximately 47,150 people were diagnosed with prostate cancer in 2015 and over 11,000 people died from the disease (5).

2.1.1 Long-term Androgen Deprivation Therapy

The initial (first-line) treatment for locally-advanced or metastatic prostate cancer is based on androgen deprivation therapy (ADT) achieved either surgically with bilateral orchidectomy, or medically with LHRH agonists or antagonists (6). Long-term use of oral anti-androgens is permitted only when given with LHRH agonists, to achieve dual androgen blockade (previously termed maximum androgen blockade - MAB).

When used alone ADT produces initial responses in up to 95% of patients but is rarely curative. STAMPEDE aims to improve outcomes for people affected by high-risk prostate cancer by testing if additional treatments added to ADT can improve disease control and life-expectancy. Data from the control arm in STAMPEDE has shown that for people with newly-diagnosed metastatic disease treated with ADT alone, the time to progression is just 11 months (6). Such progressive disease is referred to as castrate-resistant prostate cancer (CRPC).

Another important issue with ADT is the numerous associated side-effects, particularly with prolonged use. Since patients continue on LHRH after disease progression (with additional agents added), many people remain on treatment for a decade or longer. STAMPEDE is evaluating alternative forms of ADT and additional treatment with metformin aiming to mitigate some of the adverse effects of ADT which include osteoporosis (leading to an increased risk of fracture), adverse metabolic disturbance, cognitive decline, sexual dysfunction, hot flushes, physical deterioration and fatigue.

2.1.2 Role Of SOC Radiotherapy

Two randomised trials, SPCG7 (7) and NCIC PR.3 / MRC PR07 (8-10) have tested the question of whether ADT alone combined with radiotherapy is the best treatment for patients with high-risk localised prostate cancer (N0M0). Both trials demonstrated an improvement in overall and disease specific survival from the addition of radiotherapy to ADT. The size of this overall survival benefit is substantial (hazard ratio 0.68 in SPCG7 and 0.77 in PR07). As these two mature, large, well-conducted randomised trials have demonstrated benefit, we now mandate that radiotherapy be standard for patients with N0M0 disease (i.e. no nodal or metastatic spread). Patients with node-negative M0 prostate cancer will only be allowed to enter the trial if standard radiotherapy is planned. For patients with node-positive, M0 disease there are no randomised data on whether radiotherapy is indicated or not. However the NCIC PR.3 / MRC PR07 trial included patients with unknown nodal status who received whole pelvic radiotherapy (11) and demonstrated a large overall benefit. Additionally, non-randomised data from the STAMPEDE control arm suggests that the benefit observed in patients with N0M0 disease can be extended to those with pelvic nodal involvement. Therefore the STAMPEDE TMG recommends that pelvic nodal radiotherapy be considered for patients with node-positive, M0 disease at the discretion of the treating clinician (12).

More recently, data from the “M1:RT” arm showed that in patients with oligo-metastatic disease, RT to the prostate improved overall survival (4). Therefore, the STAMPEDE TMG recommends that

prostate RT is considered for patients with oligometastatic (low burden) disease at the discretion of the treating physician.

2.1.3 Role Of SOC systemic therapy: docetaxel, abiraterone, enzalutamide or apalutamide in addition to ADT

A variety of trials have demonstrated that addition of systemic therapy at the start of long term ADT prolongs survival, particularly in participants with metastatic disease. On the basis of the studies described below, the STAMPEDE TMG stongly recommends the clinician to consider either docetaxel, abiraterone, enzalutamide or apalutamide in all participants with metastatic disease at presentation who are commencing ADT for the first time and are fit enough to additional treatment. Choice of which systemic therapy to use is at the discretion of the clinician, but will need to be guided by availability of each treatment at site. We also suggest site investigators keep abreast of the latest published literature to inform choices between these treatments when options are available.

The primary analysis of the "original comparisons" has shown docetaxel significantly prolongs survival (HR 0.78; 95% CI 0.66-0.93)(1). This is in support of the results of the CHAARTED trial which showed docetaxel improved survival in people with metastatic disease (13, 14). There was no evidence of heterogeneity in STAMPEDE in the treatment effect across any patient subgroups and median survival was improved by 10 months, from 71 to 81 months. In a well powered and pre-planned sub-group analysis of people with metastatic disease at randomisation the treatment effect was most apparent with a median survival benefit of 15 months.

Data from the long term follow up of the docetaxel arm, specifically in men with non-metastatic disease, demonstrate that men who had RT to the prostate without chemotherapy had a superior FFS, PFS and a trend towards better OS, compared to men treated with both (15) . Therefore, although docetaxel is now permitted as part of the standard-of-care for all people entering STAMPEDE, we recommend patients with non-metastatic disease should be considered primarily for prostate RT, and chemotherapy considered only for those in whom RT is contra-indicated. Ultimately the decision is at the discretion of the treating clinician and patient.

The primary analysis of the “abiraterone comparison” has shown abiraterone improves survival in the HSPC setting (HR 0.63; 0.52 to 0.76; p<0.001)(3). The results are consistent with the co-published LATITUDE trial which recruited an overlapping subset (newly-diagnosed high-risk metastatic patients (16)) of the population eligible for STAMPEDE. A post-hoc subgroup analysis of the metastatic HSPC participants recruited to the STAMPEDE abiraterone comparison, suggest that benefit from abiraterone was irrespective of risk stratification via “risk” or “volume” measures (17).

The ENZAMET trial (18) demonstrated that enzalutamide used alongside ADT in the metastatic HSPC setting improved overall survival (HR 0.67; 0.52 to 0.86; P=0.002). Where available it is acceptable to use the addition of enzalutamide to ADT on the basis of evidence of benefit. Of note, there was no additional survival benefit seen in those patients treated with both docetaxel and enzalutamide in the upfront setting, whilst this combination resulted in higher rates of peripheral sensory neuropathy.

The TITAN trial (19) demonstrated that apalutamide used alongside ADT in the metastatic HSPC improved overall survival (HR 0.67; 0.51 to 0.89; p=0.005).

In the absence of data supporting a combination of treatment in the upfront setting, investigators are required to specify which upfront treatment will be used. It is **not** appropriate to use a **combination** of these treatments. Therefore, from protocol v21.0 onwards, SOC use of docetaxel**, or**

abiraterone, **or** enzalutamide, **or** apalutamide is permitted at the discretion of the treating clinician and patient. The choice of SOC treatment must be selected **prior** to randomisation.

Transdermal oestradiol has not previously been used alongside abiraterone, enzalutamide or apalutamide. Therefore the first cohort of participants recruited to STAMPEDE and started on this combination will have enhanced safety monitoring, with close monitoring of hormone levels. Any concerns that an interaction between these treatments is impacting efficacy will result in a pause to recruitment whilst this is investigated. A pre-planned review of early efficacy to achieve castration will be carried out once sufficient participants have been treated with these combinations. See **Section 9.7.4** for further details.

2.2 DESIGN

STAMPEDE (also known as MRC PR08) is an innovative, multi-arm multi-stage (MAMS) randomised controlled trial open in multiple sites in the UK and Switzerland. The multi-arm design allows many treatment approaches to be tested simultaneously, and multi-stage refers to the pre-specified interim analyses that can be used to stop recruitment early to arms showing insufficient evidence of activity. The trial recruits people with high-risk locally advanced or metastatic prostate cancer, commencing long-term ADT for the first time.

The trial opened to recruitment in 2005 and initially assessed the effects of a bisphosphonate (zoledronic acid), a cytotoxic chemotherapeutic agent (docetaxel) and a cyclooxygenase (Cox-2) inhibitor (celecoxib), as single agents or combinations (arms B-F), referred to as the “original comparisons”.

Since the start of the trial, a number of new research arms have been added to STAMPEDE to evaluate:

- Abiraterone, a steroid synthesis inhibitor (arm G)
- Prostate radiotherapy for patients with newly-diagnosed metastatic disease (arm H)
- Enzalutamide, an inhibitor of androgen receptor signalling, given with abiraterone (arm J)
- Metformin, a repurposed anti-diabetic medication (arm K)
- Transdermal oestradiol, a repurposed alternative form of ADT (arm L)

2.3 PREVIOUSLY-REPORTED RESEARCH TREATMENTS

Data have been reported on the “original comparisons” evaluating zoledronic acid, docetaxel, celecoxib and the combination of zoledronic acid with docetaxel or with celecoxib (1, 2, 20). As such, the rationale for these treatments, along with their design and details of treatment administration, are no longer covered within this version of the protocol.

The primary survival analysis of the “abiraterone comparison” has also now been reported although these participants remain on active follow-up as subsequent analyses of long-term follow-up are planned (3). The rationale can be found in previous protocol versions, however treatment information remains as it is relevant to participants who remain on abiraterone given alone (arm G) and in combination with enzalutamide (arm J).

The primary survival analysis of the “M1:RT” comparison testing RT to the primary tumour for men with newly diagnosed metastatic prostate cancer (Arm H) has now also been reported. These patients remain on active follow-up as subsequent analyses of long term follow-up planned. The treatment information is no longer covered within this version of the protocol as all treatment has now completed, however this can be accessed via previous versions of the protocol as below.

All previous versions of the protocol are available via www.stampedetrial.org, please refer to:

- Protocol version 11.0 and before for information relevant to "original comparisons" (Zoledronic acid, docetaxel, celecoxib)
- Protocol version 8.0 to 13.0 for information relevant to the "abiraterone comparison"
- Protocol version version 9.0 to 15.0 for information relevant to the "M1:RT comparison"

2.4 COMPARISONS IN FOLLOW-UP

The rationale for comparisons that have completed recruitment and remain in follow-up can be found in previous versions of the protocol. Recruitment was completed to the "enzalutamide and abiraterone comparison" in March 2016, as the recruitment target was reached. Participants remain on treatment therefore this information remains in this protocol version.

All previous versions of the protocol are available via www.stampedetrial.org, please refer to:

- Protocol version 16.0 or older for details relevant to "enzalutamide & abiraterone comparison"

2.5 RATIONALE FOR RECRUITING COMPARISONS

2.5.1 Metformin

All people joining STAMPEDE are planned for long-term ADT, a treatment associated with an increased risk of insulin resistance, hyperglycaemia, dyslipidaemia and obesity. Over 50% of people receiving long-term ADT will develop metabolic syndrome resulting in increased cardiovascular morbidity and mortality (21). Obesity and high bind insulin C-peptide levels, indicating insulin resistance are independent predictors of increased prostate cancer-specific mortality and the presence of metabolic syndrome and diabetes in people treated with ADT is associated with shorter survival.

Metformin, which in non-diabetic individuals has been shown to lower the incidence of diabetes, counteracts some of these side-effects of ADT, including insulin insensitivity, hyperinsulinaemia and diabetes. It also reduces the levels of cholesterol, LDLs and triglycerides by inhibiting the fatty acid synthesis via activation of Adenosine Monophosphate Activated Kinase (AMPK) and decreases the platelet aggregation factor 1, platelet aggregation, vascular adhesion molecules, CRP and leptin (22-25). Through mitigation of the cardiovascular and metabolic consequences of ADT, metformin is proposed to reduce treatment-associated morbidity and improve all-cause mortality.

In addition, recent data has emerged consolidating the knowledge that cancer progression is linked integrally with metabolic modulators and that modification of this process by metformin has an important effect on cancer progression and survival. Pre-clinical data has shown that metformin is an important stimulator of AMPK which acts as the cellular "master switch" for energy regulation. AMPK acts to inhibit the effects of elevated insulin levels which promote metastasis, tumour growth and treatment resistance. Insulin increases mRNA and protein expression of steroidogenic enzymes leading to the up-regulation of intracellular testosterone levels, secreted androgens, thereby activating the AR (26). Metformin also influences the PI3K-AKT pathway and has an anti-proliferative effect via inhibitor of mTOR as well as targeting cancer stem cells. In vitro, metformin has been shown to inhibit androgen-induced IGF-IR up-regulation through disruption of androgen signalling (27).

Evidence in support of this includes a systematic review and meta-analysis of 13,008 people with type 2 diabetes mellitus (T2DM) and concurrent cancer which has shown improved survival in people treated with metformin compared with other anti-diabetic agents. In a systematic review of

observational data from over 1 million people, there was a significant association seen between metformin and decreased risk of death from any cancer. Another systematic review found that the use of metformin in diabetic patients was associated with a significantly lower risk of cancer incidence and cancer mortality (28). In a large retrospective cohort study of 3837 diabetic people with prostate cancer, metformin was associated with a decreased risk of prostate cancer specific mortality (HR=0.76 [0.64-0.89]) and death (HR=0.76 [0.70-0.82]). In a prospective non-randomised phase II study in non-diabetic CRPC patients, 36% of patients receiving metformin were progression-free at 3 months and >50% had a prolongation of their PSA doubling time (29).

In summary, meformin is proposed to mitigate many of the adverse side-effects of long-term ADT as well as having multiple potential anti-cancer effects and therefore STAMPEDE will evaluate re-purposing this treatment as a novel therapeutic approach in the management of high risk locally-advanced or metastatic prostate cancer.

2.5.2 Transdermal Oestradiol

2.5.2.A Background & Rationale

ADT with LHRH analogue injections suppresses testosterone to castrate levels, but also depletes oestradiol, since around 80% of oestradiol in men is derived by aromatisation from testosterone. Thus men who are treated with LHRHa will have toxicities caused by low levels of both testosterone and oestrogen. The LHRH-associated toxicities which are due to low testosterone include loss of libido, erectile dysfunction and decrease in muscle mass. Other toxicities associated with LHRHa such as osteoporosis, increased fracture risk, hot flushes, memory loss, dyslipidemia and increased body fat deposition are thought to be due to oestradiol deficiency. In particular, the adverse effect of LHRHa on bone health has been well documented. Oestradiol deficiency prolongs the life-span of bone-resorptive osteoclasts, with the resulting imbalance between osteoclasts and bone-forming osteoblasts increasing the rate of bone thinning. This may lead to osteoporosis and increased risk of fracture, with the rate of fracture increasing with duration of LHRHa (30).

Transdermal oestradiol is a potential alternative to LHRHa that may avoid some treatment-related side-effects, therefore improving quality-of-life, which would be advantageous if shown to be equally effective at prolonging survival. Exogenous administration of oestradiol suppresses androgen production through a negative feedback loop involving the hypothalamic-pituitary axis, whilst avoiding the fall in oestradiol associated with castrate levels of testosterone (31). This, in turn, mitigates the toxicities of LHRH associated with oestradiol deficiency. Oral oestrogen was previously used for ADT before the development of LHRHa, but discontinued as first-line treatment due to increased thromboembolic toxicity, attributable to first-pass hepatic metabolism (32).

Parenteral administration (e.g. intravenous, intramuscular or transdermal oestradiol) avoids first-pass hepatic metabolism, mitigating the cardiovascular risk, as supported by results so far from the ongoing PATCH (Prostate Adenocarcinoma TransCutaneous Hormones [MRC PR09; ISRCTN70406718]) trial and previous studies evaluating parenteral oestradiol in the form of intramuscular polyestradiol phosphate (31, 33).

To date, there are a number of encouraging results from the PATCH trial demonstrating the safety and early activity of transdermal oestradiol compared to LHRH agonists in people with advanced hormone-naïve prostate cancer (see **Appendix I** for further details). In particular, similar rates of cardiovascular events have been observed in the transdermal oestradiol and LHRHa arms, as well as equivalent rates of testosterone suppression (based on around 900 patients enrolled up to Oct-2015) (31). Transdermal oestradiol has been shown to avoid the loss in bone mineral density associated with LHRHa, and results in improved metabolic profiles and quality-of-life compared to LHRHa (34). Furthermore, a pre-planned, confidential, interim analysis of the PATCH trial in Jun-2013 based on progression-free survival (PFS) led to the trial being extended to phase III; that analysis

included n=638 participants with 206 PFS events, reviewed against a pre-specified non-inferiority margin hazard ratio of 1.25 and 1-sided alpha 0.25. The phase III evaluation of clinical efficacy for transdermal oestradiol will be based on progression-free and overall survival as co-primary outcome measures.

Demonstrating that transdermal oestradiol is an equally effective approach to ADT would provide a globally important alternative (to LHRHa), with the potential to reduce treatment-associated morbidity and improve quality-of-life. In addition, there is a possibility that transdermal oestradiol may improve overall survival compared to standard hormone therapy. First, transdermal oestradiol may reduce treatment-associated morbidity and could potentially benefit overall survival. Second, up to 30% of people with castrate-resistant prostate cancer respond to oral oestrogen as post-relapse therapy, suggesting oestradiol may potentially have additional direct anti-tumour effects (35).

2.5.2.B Meta-Analysis With PATCH Trial

To further assess the clinical efficacy of transdermal oestradiol, the relevant data from the “transdermal oestradiol comparison” within STAMPEDE will be combined with data from patients recruited into PATCH, i.e. the “transdermal oestradiol comparison” within STAMPEDE is not sufficiently powered to form a stand-alone analysis. The evaluation of transdermal oestradiol will be based on a non-inferiority approach (in contrast to the other comparisons within STAMPEDE which are superiority questions), to test the hypothesis that transdermal oestradiol is at least as effective as standard hormone therapy, but with fewer side-effects.

Recruitment of patients to the “transdermal oestradiol comparison” through STAMPEDE enables the transdermal oestradiol research question to be answered more quickly than via PATCH alone. It also reduces the number of participants allocated standard treatment alone in both trials, thereby increasing the proportion of participants receiving a novel treatment approach and improving trial efficiency.

As of Feb-2017, nearly 1,200 participants had been recruited directly to the PATCH trial (also coordinated by MRC CTU at UCL) for the phase III evaluation of clinical efficacy of transdermal oestradiol. The overall recruitment target for the transdermal oestradiol evaluation is approximately 2,500 participants (including around 700 to be recruited through STAMPEDE).

3 SELECTION OF INSTITUTIONS AND INVESTIGATORS

Sites who wish to participate in STAMPEDE should be registered with the MRC CTU at UCL for this purpose. Before any participants are randomised, the CTU must receive a completed and signed Investigator Statement. The STAMPEDE Investigator Statement is signed by the Principal Investigator for that institution (download from http://www.stampedetrial.org/). The return of the Investigator Statement will be taken as confirmation of agreement to adhere to the trial protocol. In addition, a fully-signed model agreement is also required before recruitment can begin.

In compliance with the principles of GCP, all institutions participating in the trial will complete a delegation log and forward this to the CTU. Each person working on the STAMPEDE trial must sign off a section of this log indicating their responsibilities. The CTU must be notified of any changes to trial personnel and/or their responsibilities and an updated delegation log needs to be sent in to the CTU. An up-to-date copy of this log must be stored in the Investigator Site file at the institution and also at the CTU.

The Clinical Trial Authorisation (CTA) for the STAMPEDE trial requires that the Medicines and Healthcare Products Regulatory Agency (MHRA) be supplied with the names and addresses of all participating investigators/institutions. Trial staff at the CTU will perform this task; hence, it is vital to receive full contact details for all investigators prior to their entering participants.

Following substantial amendments and new comparisons opening, sites will be notified of relevant documents and training required and if and when they are able to participate. Further accreditation packs may be circulated as a result to update trial documentation.

3.1 SITE/INVESTIGATOR CRITERIA

3.1.1 Principle Investigator's Qualifications & Agreements

1. The investigator(s) should be qualified by education, training, and experience to assume responsibility for the proper conduct of the trial at their site and should provide evidence of such qualifications through an up-to-date curriculum vitae and/or other relevant documentation requested by the Sponsor, the REC, the IRB, and/or the regulatory authorities.

2. The investigator must hold a long term contract with their site. Locum members of staff cannot fill the role of Principle Investigator (PI).

3. The investigator should be thoroughly familiar with the appropriate use of the investigational products, as described in the protocol, current Investigator Brochure or Summary of Product Characteristics and in other information sources provided by the Sponsor.

4. The investigator should be aware of, and should comply with, the principles of GCP and the applicable regulatory requirements. A record of GCP training should be accessible for all investigators.

5. The investigator/site should permit monitoring and auditing by the Sponsor, and inspection by the appropriate regulatory authorities.

6. The investigator should maintain a delegation log of appropriately-qualified persons to whom the investigator has delegated significant trial-related duties.

7. The investigator should sign an investigator statement, which verifies that the site is willing and able to comply with the requirements of the trial.

3.1.2 Adequate Resources

1. The investigator should be able to demonstrate a potential for recruiting the required number of suitable subjects within the agreed recruitment period (that is, the investigator regularly treats the target population).

2. The investigator should have sufficient time to properly conduct and complete the trial within the agreed trial period.

3. The investigator should have available an adequate number of qualified staff and adequate facilities for the foreseen duration of the trial to conduct the trial properly and safely. In addition, the investigator should arrange for suitably qualified investigator cover for safety reporting in the event of their absence.

4. The investigator should ensure that all persons assisting with the trial are adequately informed about the protocol, the investigational products, and their trial-related duties and functions.

5. The site should have sufficient data management resources to allow prompt data return to the CTU.

3.2 COMPARISON-SPECIFIC SITE ACCREDITATION

3.2.1 Transdermal Oestradiol Comparison: arms A & L

Only UK sites participating in STAMPEDE will be accredited for the “transdermal oestradiol comparison”.

3.3 REQUIRED TRIAL DOCUMENTATION

Table 7 presents a summary of the required trial documentation for participating sites. Templates are provided on the STAMPEDE website, www.stampedetrial.org.

Table 7: Trial documentation required for participating sites

TRIAL DOCUMENTATION	TIMING
Confirmation of capacity and capability (including IRMER approval)	Before site participation
Signed Investigator Statement	Before site participation
Signature list & delegation of responsibilities	Before site participation
Trial personnel contact details	Before site participation
Participant information sheets (PIS), GP Letter & Informed consent form (ICF) on local paper	Before site participation
Signed Clinical Trial Agreement between Trust and Sponsor (or Variation if applicable)	Before site participation
Site initiation training	Before site participation
Signed Pharmacy Pack acknowledgment	Before site participation

4 SELECTION OF PARTICIPANTS

4.1 IDENTIFYING POTENTIAL TRIAL PARTICIPANTS

STAMPEDE recruits participants with high-risk prostate cancer who are commencing long-term androgen-deprivation therapy (defined as at least 2 years) for the first time. All participants must fulfil one of the following **broad disease categories:**

- High-risk newly-diagnosed non-metastatic, node-negative disease
- Newly-diagnosed metastatic or node-positive disease
- Previously radically treated, now relapsing with high risk features

See **Section 4.4** for detailed category inclusion criteria

4.2 APPROACH TO INFORMED CONSENT

Potential participants should be provided with information about STAMPEDE at the earliest opportunity to allow sufficient time to consider their participation and complete the required screening procedures to determine comparison-specific eligibility.

Informed consent is an ongoing process and participants must be made aware that refusal to take part in all or any aspect of the trial at any time for any reason is permitted, without incurring any consequence or impact on their standard treatment. All aspects of the trial e.g. sub-studies should be presented and optional participation discussed, and investigators are encouraged to adopt a staged approach where possible to avoid information overload.

Original signed consent forms must be stored in the site investigator file, a copy stored in the patient's medical notes (electronic/paper), and a copy provided to the participant. For central monitoring purposes an anonymised copy must also be sent to the CTU, refer to **Section 10.1.1**.

4.2.1 Screening Investigations Prior To Randomisation

All participants must have the following examinations performed to confirm eligibility prior to randomisation. Please note, all screening investigations should be recent such that they reflect the participant's current disease status.

The following imaging is always required within 6 months (184 days) prior to randomisation:

- Cross-sectional imaging (CT, MRI, PSMA-PET-CT or Choline-PET-CT) of pelvis and abdomen, SPECT-CT is not sufficient
- AND Bone Scan (or equivalent e.g. whole body MRI, or SPECT-CT)
- AND Chest X-ray (only if chest was not included in cross sectional imaging i.e. CT, Choline-PET-CT or PSMA-CT-PET which would be preferable; MRI imaging of chest is not sufficient on its own)

Please note, for trial purposes M1 disease will be defined using internationally agreed criteria, therefore M1 staging cannot be based solely on PET avid lesions. To be considered M1, the metastatic lesion must also be visible on standard imaging i.e. CT (can be CT component of PET-CT) or bone scan.

The following blood tests are required within **6 months (184 days)** prior to randomisation:

- Pre-hormone treatment PSA

- Pre-hormone treatment Testosterone (if available)

The following bloods and additional measurements are required within **4 weeks (28 days)** prior to randomisation:

- Haematology: Full blood count*
- Biochemistry: Liver function tests, serum creatinine
- Systolic and diastolic blood pressure
- Waist circumference measurement
- Weight and height

The following bloods and additional measurements are required within **2 weeks (14 days)** prior to randomisation:

- Baseline PSA

* If a participant has started SOC docetaxel please use a full blood count measured shortly prior to chemotherapy. This means in the setting of SOC docetaxel these results provided should be from within the last 16 weeks (112 days) prior to randomisation. This will ensure an appropriate baseline is reviewed to confirm fitness for treatment and eligibility for participants. For all other participants the blood count used should be taken within 4 weeks prior to randomisation.

Participants who initially fail to meet the trial eligibility criteria can be re-screened at a later date if timelines permit.

4.2.2 Baseline Investigations required for participants allocated to arms A, K, L

The following blood tests and additional measurements are required at baseline **within 4 weeks (28 days)** prior to randomisation:

- HbA1c (for participants being considered for metformin comparison)
- Glucose and triglycerides (preferably fasting for metabolic analysis, but if only able to obtain a non-fasting result please record on randomisation CRF)
- Lipid profile (fasting or non-fasting; total cholesterol, LDL and HDL)

See **Table 2** for a detailed schedule of assessments for all participants randomised to arms A, K or L.

We encourage site investigators to carry out any additional investigations they feel are necessary in particular cases to ensure that participants are appropriately fit to be randomised in the STAMPEDE trial.

4.3 PRIOR PERMITTED SOC TREATMENTS

4.3.1 Hormone Treatment Prior To Randomisation

From protocol v16.0, participants can potentially be randomised to the “transdermal oestradiol comparison” and it would be preferable for these participants to have had as little exposure to ADT as possible.

Within the separate PATCH trial, participants are randomised within 8 weeks after starting anti-androgens and cannot have received an LHRHa injection. This approach is also favoured in STAMPEDE, but participants who have received a single 4-week (or 1-month) LHRHa injection remain eligible, as shown in **Table 8.**

Anti-androgen monotherapy is not permitted as a form of long-term hormone therapy but initial use is encouraged to meet the eligibility criteria for the “transdermal oestradiol comparison”. Anti-

androgens may include flutamide or bicalutamide, however use of cyproterone will mean the participant is ineligible for arm L (36).

Table 8: Maximum prior hormone therapy

TIME CONSIDERATIONS	PRIOR ANTI-ANDROGENS	PRIOR LHRH	PRIOR SOC ABIRATERONE, ENZALUTAMIDE OR APALUTAMIDE	ELIGIBLE FOR INCLUSION TO
Maximum duration – all arms except Arm L	14 weeks	12 weeks	12 weeks	A:K
Arm L	≤8 weeks	≤4 weeks	Nil	A:L

Permitted prior hormone therapy for now-relapsing disease: Any patients now presenting with relapsed disease, previously treated with adjuvant or neo-adjuvant hormone therapy alongside their radical surgery or radiotherapy, must have completed that period of hormone therapy **at least 12 months** before joining STAMPEDE and it must have been **no longer than 12 months in duration**.

4.3.2 Standard-Of-Care (SOC) Radiotherapy

In participants with N0M0, N+M0 and oligometastatic disease (as per M1RT definition (4), see Section 6.1.3), the treating clinician and participant must have decided, **prior** to randomisation, whether prostate radiotherapy will be given as part of SOC.

4.3.3 Standard-Of-Care (SOC) Systemic Therapy; docetaxel, abiraterone, enzalutamide or apalutamide

The treating clinician and participant must have decided, **prior** to randomisation, whether docetaxel, abiraterone, enzalutamide or apalutamide is to be given as part of SOC.

Please note that only one SOC treatment can be selected at randomisation. See Section 6.1.4 for treatment details.

Investigators should aim to start SOC docetaxel treatment within 12 weeks after starting ADT, consistent with the timelines achieved for research arm C. Participants may start docetaxel treatment prior to randomisation. See Section 6.1.3 for treatment details.

If SOC docetaxel treatment was not commenced prior to randomisation and participants are subsequently allocated to receive transdermal oestradiol (Arm L), it is recommended that docetaxel treatment commences **after** participants have been established on transdermal oestradiol for around 4 weeks, when most participants are likely to have completed the induction period (see Section 6.2.5.A).

Participants may start abiraterone, enzalutamide or apalutamide prior to randomisation. However the use of these treatments prior to randomisation will impact comparison-specific eligibility. At present participants who have **already started** these treatments will only be eligible for the "metformin comparison". Patients **planned** for these treatments can be considered for both metformin and transdermal oestradiol comparisons.

At present, there are no safety data available on the use of abiraterone, enzalutamide or apalutamide in combination with transdermal oestradiol. The initial cohort of participants randomised to receive transdermal oestradiol and planned for SOC abiraterone, enzalutamide or apalutamide will be subject to additional CTU review to monitor these combinations, and an additional early pre-planned analysis of safety.

When complete, the findings will be reviewed by the relevant committees and sites will be advised as to whether these combinations can continue. If found to be safe in the upfront setting then we will also there-after permit the use of the medications alongside transdermal oestradiol patches in the CRPC setting.

4.4 GENERAL INCLUSION CRITERIA – DISEASE CATEGORIES

Participants must fulfil all the criteria in one of the following three categories:

4.4.1 High-Risk Newly-Diagnosed Non-Metastatic Node-Negative (N0/Nx) Disease

Both:

- At least two of: T category T3/4, PSA≥40ng/ml or Gleason sum score 8-10
- Intention to treat with radical radiotherapy (unless there is a contra-indication)

OR

4.4.2 Newly-Diagnosed Metastatic Or Node-Positive Disease

At least one of:

- Stage T_{any} N+ M0
- Stage T_{any} N_{any} M+

OR

4.4.3 Previously Radically Treated, Now Relapsing (Prior Radical Surgery And/or Radiotherapy)

At least one of:

- PSA ≥4ng/ml and rising with doubling time less than 6 months
- PSA ≥20ng/ml
- N+
- M+

AND

4.5 GENERAL INCLUSION CRITERIA REQUIRED FOR ALL PARTICIPANTS

I. Histologically confirmed prostate adenocarcinoma
II. Intention to treat with long-term androgen deprivation therapy
III. Fit for all protocol treatment[1] and follow-up, WHO performance status 0-2[2]
IV. Have completed the appropriate investigations prior to randomisation
V. Adequate haematological function: neutrophil count ≥$1.5x10^9$/l and platelets ≥$100x10^9$/l
VI. Adequate renal function, defined as GFR ≥30ml/min/$1.73m^2$
VII. Written informed consent
VIII. Willing and expected to comply with follow-up schedule
IX. Using effective contraceptive method if applicable

[1] Medical contraindications to the trial medications are given in **Section 6**
[2] For WHO performance status definitions see **Appendix A**

4.6 GENERAL EXCLUSION CRITERIA

I. Prior systemic therapy for locally-advanced or metastatic prostate cancer (except as listed in **Section 4.3**[1])

II. Prior exposure to hormone therapy for a duration of > 12 months, or prior exposure completing < 12 months before randomisation (see **Section 4.3.1** for permitted prior exposure details)

III. Metastatic brain disease or leptomeningeal disease

IV. Abnormal liver functions consisting of any of the following:
   - Serum bilirubin ≥1.5 x ULN (except for participants with Gilbert's disease, for whom the upper limit of serum bilirubin is 51.3µmol/l or 3mg/dl)
   - Aspartate aminotransferase (AST) or alanine aminotransferase (ALT) ≥2.5 x ULN - site must indicate at randomisation whether one or both tests are performed at site. Where both results are available, both must confirm eligibility.

V. Any other previous or current malignant disease which, in the judgement of the responsible clinician, is likely to interfere with STAMPEDE treatment or assessment

VI. Any surgical wound (e.g. TURP) which in the judgement of the responsible clinician may interfere with or be exacerbated by protocol treatment

VII. Participants with significant cardiovascular disease, including:

- Severe/unstable angina
- Myocardial infarction less than 6 months prior to randomisation
- Arterial thrombotic events less than 6 months prior to randomisation
- Clinically significant cardiac failure requiring treatment, defined as New York Heart Association (NYHA) class II or above[2]
- Cerebrovascular disease (e.g. stroke or transient ischaemic episode) less than 6 months prior to randomisation
- Any other significant cardiovascular disease that in the investigator's opinion means the participant is unfit for any of the study treatments.

[1] Details timelines for recently initiated SOC docetaxel, abiraterone, enzalutamide, apalutamide
[2] NYHA classifications can be found in Appendix A

4.7 COMPARISON-SPECIFIC ELIGIBILITY CRITERIA

In addition to the general inclusion and exclusion criteria, the following comparison-specific eligibility criteria apply.

4.7.1 Metformin Comparison (randomisation between arm A and arm K)

Please note from protocol v21 only patients willing to participate in the metabolic sub study should be randomised to the metformin comparison. The sub study will be conducted in a limited number of sites, see section 4.7.4 for further information.

In addition to the general inclusion and general exclusion criteria the following comparison-specific inclusion criteria must be met to be eligible for randomisation to the "metformin comparison":

- Hb A1c <48mmol/mol (equivalent to <6.5%)
- Adequate renal function, defined as GFR ≥45ml/min/1.73m^2 (except for Switzerland[1])
- No history of lactic acidosis or predisposing conditions
- No current or previous treatment with metformin
- No current or previous medication for treatment of diabetes
- No contraindications to metformin
- Willingness to join the metabolic sub study

The method used to determine glomerular filtration rate may vary according to local practice. Equations that either estimate glomerular filtration rate (eGFR) or creatinine clearance (CrCl) may be used and the same threshold value applies. Where possible, HbA1c should be performed prior to commencing SOC docetaxel to reduce the likelihood of corticosteroid-related hyperglycaemia impacting on eligibility. All participants with abnormal baseline HbA1c (i.e. 6.5% or higher) should be informed and referred to their GP for further management.

4.7.2 Transdermal Oestradiol Comparison (randomisation between arm A and arm L)

In addition to the general inclusion and exclusion criteria, participants fulfilling all of the following are eligible for the “transdermal oestradiol comparison”:

- ≤8 weeks of anti-androgen (AR-antagonists) use
- Maximum of 1 dose of monthly or 4-weekly LHRH agonist/antagonist
- No prior LHRH agonist injection with a stated duration of effect greater than 1 month
- ≤12 weeks since first dose of any hormone therapy
- Not had a bilateral orchidectomy
- No use of cyproterone acetate prior to randomisation
- No known porphyria
- No history of radiologically confirmed deep vein thrombosis or pulmonary embolism
- No known thrombophilic disorder (e.g. Protein C, Protein S, antithrombin deficiency)
- Not yet started SOC abiraterone, enzalutamide or apalutamide (see Section 4.3.4 for information)

[1] Switzerland sites - please refer to SAKK appendix for local guidance

4.8 SUB-STUDY ELIGIBILITY CRITERIA

There are currently four sub-studies that aim to further the understanding of the biology of prostate cancer through additional genetic analyses and correlation with clinical data. For details on each sub-study, see **Section 17.2.**

4.8.1 Eligibility for germline DNA sub-study (Saliva samples)

All newly randomised trial participants who join arms A, K or L are asked to provide a saliva sample from which germline (inherited) DNA can be extracted.

Participants randomised from **15-Nov-2011** onwards who consented to provide a blood spot (Consent Form version 4.0 part K) can also be retrospectively approached to provide a saliva sample providing they have received the REC-approved letter explaining the need for additional saliva sample collection as the DNA extraction using the blood spot method did not work as well as anticipated.

For further information please refer to the **Sample collection and handling manual**.

4.8.2 Eligibility for the circulating tumour DNA sub-study (sequential blood samples)

This substudy is not recruiting currently. For details relating to blood sample collection for patients already participating in the substudy, including eligibility criteria and shipping refer to the **Sample collection and handling manual.**

4.8.3 Eligibility for tumour sample analysis (FFPE blocks)

All newly randomised trial participants should be provided with the STAMPEDE Additional Research Participant Information Sheet in order to consider optional donation of remaining diagnostic prostate cancer tissue stored as formalin fixed paraffin embedded (FFPE) blocks.

The criteria for enrolment into the FFPE block collection:

- Newly randomised STAMPEDE participants
- Informed consent to gift remaining tissue to be used for additional research analyses

Tumour blocks will only need to be sent for a select subset of STAMPEDE patients. For more details, please refer to the **Sample collection and handling manual.**

4.8.4 Eligibility for metformin metabolic sub-study

All newly randomised trial participants who meet the eligibility criteria to join the A/K comparison are eligible to join the metformin metabolic sub-study, if the site where they are being treated is participating in this sub-study. A limited number of sites will be recruiting for this sub-study. Selected sites involved have volunteered and demonstrated they have sufficient resources to undertake the metabolic sub-study.

Participants who are eligible for randomisation to the metformin comparison must be willing to take part in the metabolic sub-study and be able to adhere to the blood sample schedule. Appropriate consent to the additional blood samples must be provided.

5 RANDOMISATION AND ENROLMENT

5.1 RANDOMISATION

Participant eligibility will be confirmed during the randomisation process and participants will be allocated to any of the open research comparisons for which they are eligible (see **Section 4.6**). To randomise a participant please carefully complete the Randomisation CRF and then contact the CTU.

**RANDOMISATION**<br>Call MRC CTU at UCL, Monday to Friday 0900-1700<br>Excluding public holidays or dates when notice has been given by the CTU.<br>Tel: +44 (0) 20 7670 4777

A trial ID and treatment will be allocated and given over the phone and by email confirmation. In addition, a letter confirming these details will be sent. The trial ID will be the primary way in which the participant will be identified and should be used in all correspondence. Sites should send a letter to the participant's GP to inform them of their trial participation and treatment allocation. The GP letter is supplied as a template and can be downloaded from the trial website www.stampedetrial.org.

The randomisation CRF, eligibility checklist and anonymised consent form* must be submitted to the CTU following randomisation.

**Anonymised consent forms are not required to be submitted for Swiss participants.*

5.2 CO-ENROLMENT GUIDELINES

Interventional clinical trials
STAMPEDE participants should not join any other interventional clinical trials of prostate cancer treatment until the following criteria have been met:

- The participant has experienced at least one failure-free survival (FFS) event
- The participant is no longer on any STAMPEDE research treatment that is permitted to continue post first progression e.g. metformin, abiraterone or enzalutamide

Once both criteria are satisfied the participant may be entered into further treatment studies evaluating treatments for CRPC.

Site investigators should check with the CTU prior to participants commencing any IMP within an **interventional clinical trial** for any other medical condition, such as a new malignancy, to ensure there are no concerns about interactions with STAMPEDE treatments. Note that STAMPEDE treatment can be continued alongside **non-trial treatments** for a new malignancy providing local pharmacy review to ensure there are no interactions.

The primary outcome measure of STAMPEDE is overall survival, therefore follow-up must continue after co-enrolment (unless the participant withdraws consent). Participation in interventional studies must be reported to CTU on the Co-enrolment CRF. Details of any interventional treatments received post-progression in such studies must be reported on the Additional Treatment Log.

Non-interventional clinical trials
Co-enrolment in non-interventional studies for any indication is permitted at any time providing that it does not interfere with treatment or assessment in STAMPEDE. This does **not** require reporting using the Co-enrolment CRF which captures details of interventional prostate cancer clinical trials only.

Data sharing agreements with “downstream” trials are encouraged to improve data quality in both trials and to reduce costs to both organisations.

6 TREATMENT OF PARTICIPANTS

6.1 STANDARD-OF-CARE (SOC)

The SOC for this patient group is **androgen deprivation therapy** (ADT) as per local practice (see Section 6.1.1). For some participant groups, this should now be supplemented with SOC radiotherapy (see Section 6.1.2). From protocol v14.0 onwards, SOC docetaxel is permitted for all suitable participants. From protocol v19.0 onwards, SOC abiraterone is also be permited as an alternative to docetaxel, where this is available. From protocol v21.0 onwards SOC enzalutamide and apalutamide are also permitted as an alternative to docetaxel or abiraterone, where these are available, (see Section 6.1.4).

**SOC combinations**	**Metformin comparison**	**Transdermal oestradiol comparison**
ADT alone	Yes	Yes
ADT + prostate RT +/- nodal RT	Yes	Yes
ADT + docetaxel	Yes	Yes
ADT + docetaxel + RT	Yes	Yes
ADT + abiraterone/enzalutamide/apalutamide	Yes	Yes*
ADT + abiraterone/enzalutamide/apalutamide + RT	Yes	Yes*

*See section 7.1.5.C for guidelines for submitting hormone treatment logs – please send as quickly as possible to facilitate safety monitoring of combination.

6.1.1 Androgen Deprivation Therapy

The planned duration of ADT should be **at least 2 years** and lifelong in those with metastatic disease. With the exception of those allocated to transdermal oestradiol (Arm L), all participants will receive ADT as per local practice to achieve castrate levels of testosterone. The method of planned or current long-term standard-of-care ADT must be specified prior to randomisation. See below for the permitted methods of ADT and see Section 4.3.1 for more information on ADT timing before randomisation. Participants allocated to Arm L will go on to receive transdermal oestradiol in place of standard ADT methods.

6.1.1.A Bilateral Orchidectomy

Operations should be performed by appropriately trained surgeons. A total or sub-capsular orchidectomy may be performed. Participants having a bilateral orchidectomy are required to adhere to the same timelines for prior LHRH and/or anti-adrogen exposure as specified in Section 4.3.1. Note, bilateral orchidectomy is an exclusion criteria for the “transdermal oestradiol” comparison, see section 4.6.2.

6.1.1.B LHRH Agonists e.g. goserelin, leuprorelin

LHRH agonists used according to local practice. The prophylactic use of anti-androgens to prevent tumour “flare” is recommended.

6.1.1.C LHRH Antagonists e.g. degarelix

LHRH antagonists used according to local practice. The use of prophylactic use of anti-androgens to prevent tumour “flare” is not necessary.

6.1.1.D Dual Androgen Blockade

Long-term use of anti-androgens alongside LHRH agonists, according to local practice. Note this was previously referred to as maximum androgen blockade. Anti-androgen monotherapy is not deemed an androgen deprivation regimen.

6.1.2 SOC Radiotherapy to prostate

6.1.2.A N0M0 Participants

Investigators should give standard RT to participants with node-negative, non-metastatic disease (N0M0), in accordance with data from the PR07 and SPCG trials (7, 10). If RT is contra-indicated this must be recorded on the Randomisation CRF. See Section 6.1.3 for further details of RT administration.

6.1.2.B N+M0 Participants

For participants with node-positive, M0 disease there are no randomised data on whether radiotherapy is indicated or not. However the NCIC PR.3 / MRC PR07 trial included participants with unknown nodal status who received whole pelvic RT (11) and demonstrated a large overall benefit. Additionally, non-randomised data from the STAMPEDE control arm (Arm A) suggests that the benefit observed in participants with N0M0 disease can be extended to those with pelvic nodal involvement. Therefore, the STAMPEDE TMG recommends that pelvic nodal RT be considered for participants with node-positive, M0 disease at the discretion of the treating clinician (12).

6.1.2.C Oligometastatic Participants

For participants with oligometastatic disease, data from the M1:RT arm supports the use of RT to the prostate (4). Currently, data strongly supports the use of prostate RT in men with up to 3 bone metastases and/or lymph node only disease, however we are aware that ongoing analyses may redefine which patients benefit from this treatment. Therefore, the STAMPEDE TMG recommends that prostate +/- pelvic nodal RT be considered for participants with oligometastatic disease, with the treating clinician to determine whether the participant has oligometastatic disease that they deem likely to benefit from this treatment.

6.1.2.D Planned Use Of SOC RT

Suitability for RT is assessed by the treating clinicians. Investigators will be asked to state their intention with regards to planned RT in this group at randomisation. Intention to give RT (or not) for **all** participants must be stated at randomisation to ensure that there is no bias towards particular combinations of systemic therapy with RT.

SOC RT administration is not being investigated as part of the trial, therefore only minimal data about SOC RT will be collected. It is accepted that some participants will develop progressive disease before RT can be administered and if this occurs the reasons for non-delivery of treatment must be recorded on the Radiotherapy Detail CRF.

6.1.3 Administration of SOC RT

Standard radiotherapy will be given to appropriate participants in each of the trial arms, following a period of neo-adjuvant ADT therapy, as is generally standard in UK practice. For participants with negative nodes on axial imaging, clinicians may choose between irradiating prostate and seminal vesicles alone or including the pelvic nodes in addition. Additional staging tests such as pelvic node sampling may be considered in making this decision. Conformal or intensity modulated radiotherapy should be used in all participants. Where participants have good clinical evidence that nodes are free of tumour or participants for whom nodal radiotherapy is contra-indicated (e.g. significant bowel disease), treatment may be given to the prostate gland and seminal vesicles only. The recommended dose is 74Gy in 37 fractions to the prostate and seminal vesicles or the equivalent using hypo-

fractionated schedule, 60Gy in 20 fractions. Alternative dosing schedules are permitted but must be agreed with the STAMPEDE Trial Management Group (TMG).

6.1.3.A Standard-Of-Care RT Timing

If receiving docetaxel as part of the standard-of-care (permitted from protocol v14.0), the participant must have sufficiently recovered from any docetaxel toxicity before RT can begin. In all other participants not receiving SOC docetaxel, SOC RT may be started sooner (2-6 months post-randomisation) consistent with the data from the MRC PR07 trial (11).

6.1.4 SOC upfront systemic therapy: docetaxel, abiraterone, enzalutamide or apalutamide

Docetaxel, abiraterone, enzalutamide or apalutamide may be considered for use as SOC treatments, provided the treatment is available locally. Choice of which systemic therapy to use is at the discretion of the clinician. However, we suggest site investigators keep abreast of the emerging literature to inform choices between these treatments, when options are available.

From protocol v14.0 investigators may consider giving docetaxel as part of the SOC for participants with newly-diagnosed metastatic disease, based on the survival benefit demonstrated by both STAMPEDE in the primary analysis of the "original comparisons" and CHAARTED (13) (14, 20). Investigators may also consider giving docetaxel to participants with high-risk locally-advanced disease.

From protocol v19.0 onwards, the treating clinician and participant may consider the use of abiraterone in the newly diagnosed setting, where this is available.

From protocol v21.0 onwards the treating clinician and participant can also consider the alternate options to use enzalutamide or apalutamide in the newly diagnosed setting, if available.

The treating clinician and participant must have decided **prior** to randomisation if SOC docetaxel, abiraterone, enzalutamide or apalutamide is to be given to ensure use is balanced between control and treatment arms. Treatment with SOC systemic therapy may start prior to randomisation, except in Arm L when abiraterone, enzalutamide or apalutamide cannot have started prior to starting trial treatment. In addition, for participants allocated to Arm L who have not already started SOC docetaxel prior to randomisation, it is recommended that docetaxel commences around 4 weeks after starting research treatment (see **Section 6.2.5**).

In the absence of data supporting the routine use of concurrent or sequential use in the absence of disease progression, investigators are required to specify which SOC treatment will be used (i.e.: **one** of docetaxel, abiraterone, enzalutamide or apalutamide) and may **not** plan to use a combination of these. In the case that SOC treatment is changed from one agent to another in order to manage toxicity/intolerance (as per current national guidelines) please update the SOC Systemic Treatment CRF.

We recommend starting SOC systemic therapy within 12 weeks of initiation of ADT. This timing is consistent with the time-scale for starting treatment within the aforementioned clinical trials.

Docetaxel is given according to local protocols as a standard non-trial treatment. The regime used previously within STAMPEDE (Arm C and Arm E) was 75mg/m^2 Day 1 as 1hr IV infusion, plus prednisolone 5mg BID for 21 days repeated every 3 weeks for a maximum of 6 cycles. GCSF use is at the investigator's discretion; prednisolone may be omitted.

Abiraterone, enzalutamide or apalutamide should be given according to local protocols as a standard non-trial treatment. Currently abiraterone, enzalutamide and apalutamide are funded differently by the NHS dependent on burden of disease and which country the participant is being treated in; follow national guidelines for duration and management of therapy once available.

The dosing, safety monitoring and toxicity management contained within the STAMPEDE protocol refers to research abiraterone and enzalutamide given to participants previously allocated to Arms G or J, but may be used as a guide if required. The protocol guidelines for abiraterone were based on the recommendations from the manufacturers, which are included in the summary of product characteristics and can be found online. Similarly, for dosing, safety monitoring and toxicity management of enzalutamide and apalutamide consider referring to the summary of product characteristics produced by the manufacturers, and any local or national guidelines available.

A SOC Systemic Treatment CRF should be completed for all participants randomised to STAMPEDE regardless of whether any SOC systemic therapy was planned. See Section 7.2.3 for details of data collection for SOC Systemic Treatment.

6.2 RESEARCH TREATMENTS

Research treatment durations are outlined below with a separate section for each individual Investigational Medicinal Product (IMP) detailing the cautions and contraindications, interactions, safety monitoring and toxicity clinical management.

6.2.1 Treatment Duration

Table 9: Intended treatment duration – All arms

RANDOMISED ARM AND TREATMENT	TREATMENT DURATION IS DEPENDENT ON DISEASE STATE		
	M0 WITH PLANNED RADICAL TREATMENT[1]	M0 WITH NO PLANNED RADICAL TREATMENT[1]	M1
Arm A:SOC androgen deprivation therapy	Minimum 2 years	Continue lifelong	Continue lifelong
Arm G: Abiraterone	2 years - *unless progression occurs before (see Table 10)*	Continue until all categories of disease progression have occurred (see Table 10)	Continue until all categories of disease progression have occurred (see Table 10)
Arm J: Abiraterone and enzalutamide	2 years - *unless progression occurs before (see Table 10)*	Continue until all categories of disease progression have occurred (see Table 10)	Continue until all categories of disease progression have occurred (see Table 10)
Arm K: Metformin	Minimum 3 years (continue for 12 months after the last injection of LHRHa to allow for the delay in testosterone levels returning to normal)	Continue lifelong as long as the investigator feels it is in the best interests of the participant.	Continue lifelong as long as the investigator feels it is in the best interests of the participant.
Arm L: Transdermal oestradiol (TE2)	Minimum 2 years – *unless progression occurs before (see Table 10)*	Continue until disease progression at which point it is the site investigators prerogative to choose whether to continue TE2 or change to LHRHa. (see Table 10 for additional details on treatment post-progression)	Continue until disease progression at which point it is the site investigators prerogative to choose whether to continue TE2 or change to LHRHa. (see Table 10 for additional details on treatment post-progression)

[1] For trial purposes: Report the planned duration of trial treatment based on the intention at the time of randomisation, i.e.: based on plans for radical RT. **However** if the treatment received is different from that planned, please ensure treatment the participant receives reflects the most appropriate duration:

E.g.: N0M0 patient does not receive planned RT > treat with lifelong ADT as per "M0 with no radical treatment"
N0M0 patient receives RT that was not planned > treat with minimum 2 years ADT as per "M0 with radical treatment"

Table 10: Management of trial treatment post progression

Continues on next page.

**Randomised treatment**	**Post-progression: How to manage Trial Treatment**			
	**M0 with radical treatment - progresses whilst still on ADT**	**M0 with radical treatment - progresses after completing 2 years ADT**	**M0 with <u>no</u> radical treatment**	**M1**
Arm A:<br>SOC androgen deprivation therapy	Participants who progress before completing 2 years of ADT should continue with ADT.<br>*ADT after progression is not considered a trial protocol treatment.*	M0 participants who progress after stopping ADT at 2 years, should restart ADT.<br>*ADT after progression is not considered a trial protocol treatment.*	*Continue ADT post-progression <u>but</u> ADT is no longer considered a trial protocol treatment.*	
Arm G:<br>Abiraterone	Continue until <u>all</u> types of progression reported:<br>• PSA progression (as defined in section **7.1.3.A**)<br>• Radiological progression<br>• Clinical progression<br>However, if the participant starts second-line treatment before meeting all progression types, abiraterone must be stopped[1].	If the site clinician wishes to retreat with abiraterone this must be done following national guidelines for use in the CRPC setting.<br>*Re-treating with abiraterone in the CRPC setting is not a trial protocol treatment.*	Continue until <u>all</u> types of progression reported:<br>• PSA progression (as defined in section **7.1.3.A**)<br>• Radiological progression<br>• Clinical progression<br>However, if the participant starts second-line treatment before meeting all progression types, abiraterone must be stopped[1].	
Arm: J<br>Abiraterone and enzalutamide	Continue until <u>all</u> types of progression reported:<br>• PSA progression (as defined in section **7.1.3.A**)<br>• Radiological progression<br>• Clinical progression<br>However, if the participant starts second-line treatment before meeting all progression types, abiraterone and enzalutamide must be stopped[1].	If the site clinician wishes to retreat with abiraterone or enzalutamide this must be done following national guidelines for use in the CRPC setting.<br>Re-treating with abiraterone or enzalutamide in the CRPC setting is not a trial protocol treatment.	Continue until <u>all</u> types of progression reported:<br>• PSA progression (as defined in section **7.1.3.A**)<br>• Radiological progression<br>• Clinical progression<br>However, if the participant starts second-line treatment before meeting all progression types, abiraterone and enzalutamide must be stopped[1].	

[1] Participants may continue on abiraterone or abiraterone and enzalutamide if they receive radiotherapy on a single occasion for a skeletal-related event

<table>
<tr><th></th><th colspan="4">POST-PROGRESSION: HOW TO MANAGE TRIAL TREATMENT</th></tr>
<tr><th>RANDOMISED TREATMENT</th><th>M0 WITH RADICAL TREATMENT - PROGRESSES WHILST STILL ON ADT</th><th>M0 WITH RADICAL TREATMENT - PROGRESSES AFTER COMPLETING 2 YEARS ADT</th><th>M0 WITH <u>NO</u> RADICAL TREATMENT</th><th>M1</th></tr>
<tr><td>Arm K: Metformin</td><td>Continue metformin post-progression.
Post-progression metformin should be continued for as long as the investigator feels it is in the best interests of the participant.
Metformin can be given alongside any second-line treatment for prostate cancer.
However, if another trial with any IMP is started in the second-line setting, metformin must be stopped.</td><td>If progression occurs after stopping ADT but while metformin continues, (i.e. progression within the first 12 months of stopping ADT), continue metformin post-progression.
If progression occurs after metformin has stopped (i.e. progression >12 months after the last administration of LHRHa), metformin should not be restarted.</td><td colspan="2">Continue metformin post-progression.
Post-progression metformin should be continued for as long as the investigator feels it is in the best interests of the participant.
Metformin can be given alongside any second-line treatment for prostate cancer.
However, if another trial with any IMP is started in the second-line setting, metformin must be stopped.</td></tr>
<tr><td rowspan="2">Arm L: Transdermal oestradiol (TE2)</td><td>Continuing treatment with TE2 or change to LHRHa is at the discretion of the treating clinician.</td><td>Restart treatment with TE2 or LHRHa, the choice is at the discretion of the treating clinician.</td><td colspan="2">Continuing treatment with TE2 or changing to LHRHa is at the discretion of the treating clinician.</td></tr>
<tr><td colspan="4">TE2 can be used in combination with docetaxel, cabazitaxel and radium in the CRPC setting.
We are currently evaluating the combination of abiraterone, enzalutamide and apalutamide alongside TE2 in the upfront setting. If this is proven to be safe and effective then sites will be informed and it will thereafter also be allowed in the CRPC setting. In the meantime if you wish to use abiraterone, enzalutamide or apalutamide in the CRPC setting the participant must change to LHRHa.
For participants who are on the 3 patch maintenance dose and have castrate levels of testosterone, there is currently no evidence that increasing the number of patches further once the participant has progressed would be beneficial and is therefore not recommended.</td></tr>
</table>

6.2.2 Research Abiraterone + Prednisolone (relevant to Arms G & J)

Note: recruitment has closed to all research comparisons containing abiraterone; that is Arm G (SOC + abiraterone) and Arm J (SOC + enzalutamide + abiraterone).

Participants allocated to Arm G or Arm J will receive abiraterone:

- Arm G: abiraterone alone (taken with prednisolone), in addition to SOC ADT
- Arm J: abiraterone (taken with prednisolone) in combination with enzalutamide, in addition to SOC ADT

6.2.2.A Abiraterone: Clinical particulars – posology and administration

Abiraterone is administered as a single 1000mg daily oral dose (4 tablets to be taken together once a day).

Abiraterone should be taken with prednisolone 5mg (or prednisone 5mg in Switzerland) daily to prevent secondary mineralocorticoid excess. See **Section 6.2.2.H** for further details on prednisolone (and prednisone).

Abiraterone absorption is increased by food therefore should be taken on an empty stomach without food. The tablets should be taken at least 2 hours after food, swallowed whole with water. No food should be eaten for 1 hour afterwards.

6.2.2.B Abiraterone: Clinical particulars - treatment duration

See **Table 9** for details. See **Section 7.1.3** for further information on the trial definition of progression.

6.2.2.C Abiraterone: Safety monitoring

: Hypokalaemia, hepatic impairment and hypertension

Abiraterone may cause:

- Hypokalaemia, due to secondary mineralocorticoid excess; this can be counteracted by co-prescription of prednisolone
- Increased liver enzymes and hepatotoxicity
- Hypertension

Regular monitoring of blood serum potassium, LFTs and blood pressure are therefore required whilst on treatment. Requirements for STAMPEDE are provided in **Table 11**. Safety monitoring requirements are consistent with the approach adopted in the LATITUDE trial in which abiraterone was evaluated in high-risk metastatic hormone-naïve prostate cancer (16) and the abiraterone Investigator Brochure (38).

In summary:

- **Two weekly** monitoring of potassium, LFTs and BP for the **first 12 weeks**
- **Monthly** monitoring of potassium, LFTs and BP from **12 weeks until 1 year**
- **After 1 year** safety monitoring of potassium, LFTs and BP can reduce to **two monthly** if the site investigator thinks it is safe and appropriate to do so.

Table 11: Safety monitoring for participants receiving research abiraterone

Note: In acute toxicity monitoring requirements may increase – see toxicity tables for additional advice.

Adverse event of interest	Monitoring Required	Frequency of monitoring whilst on abiraterone alone or in combination with enzalutamide treatment in the trial setting		
		Weeks 0 – 12	Week 12 – 12 months	12 months – end of treatment
Hypokalaemia	Blood serum potassium*	Every 2 weeks	Monthly	Participants may have the frequency of monitoring reduced to every 2 months*** if judged appropriate by the investigator.<br><br>Continued monthly monitoring is required for participants if there are concerns related to research abiraterone causing hypokalaemia.
Hepatic impairment	LFTs (ALT or AST, and bilirubin)*	Every 2 weeks	Monthly	Participants may have the frequency of monitoring reduced to every 2 months*** if judged appropriate by the investigator.<br><br>Continued monthly monitoring is required for participants if there are concerns related to research abiraterone causing hepatic impairment.
Hypertension	Blood pressure**	Every 2 weeks	Monthly	Participants may have the frequency of monitoring reduced to every 2 months*** if judged appropriate by the investigator.<br><br>Continued monthly monitoring is required for participants if there are concerns related to research abiraterone causing hypertension.

* Blood tests may be taken in the community or by a GP surgery, however the results must be reviewed contemporaneously by the trial team. It is not acceptable to wait until the participant's next oncology appointment before these are reviewed.
**Blood pressure may be monitored using documented self-monitoring or via the GP providing this is reviewed at each follow up.
***Based on advice from Janssen, product IB/SmPC and LATITUDE protocol (16)

:: Hypokalaemia - additional notes
Abiraterone may cause hypokalaemia due to secondary mineralocorticoid excess, this can be counteracted by co-prescription of prednisolone (see management of hypokalaemia Table 14).

After the first 12 months, provided the site investigator feels it is appropriate and safe to do so, it is permissible for patients to be prescribed 3 months of abiraterone. As above, safety monitoring needs to be completed at 2 monthly intervals and these results need to be monitored at the time they are available to ensure it is safe for the patient to continue taking their trial medications.

:: Hepatic impairment – additional notes
Abiraterone treatment can be associated with increased liver enzymes and hepatotoxicity, therefore regular monitoring of LFTs is required whilst on treatment. LFTs should include ALT or AST, and

bilirubin. If clinical symptoms or signs suggestive of hepatotoxicity develop, serum transaminases, in particular ALT, should be measured immediately (see management of abnormal LFTs **Table 15**).

After the first 12 months, provided the site investigator feels it is appropriate and safe to do so it is permissible for patients to be prescribed 3 months of abiraterone. As above, safety monitoring needs to be completed at 2 monthly intervals and these results need to be monitored at the time they are available to ensure it is safe for the patient to continue taking their trial medications.

:: Blood pressure management – additional notes
Abiraterone may cause hypertension. Investigators are required to ensure blood pressure is performed and reviewed. , it is acceptable for this to be documented self-monitoring or via the GP providing this is reviewed at each follow-up. For the management of abiraterone-induced hypertension see **Table 13**.

6.2.2.D Abiraterone: Cautions and contra-indications

- Unusual or allergic reaction to past abiraterone treatment
- Uncontrolled hypertension
- Uncontrolled heart failure
- Active or chronic liver disease

:: Cardiovascular history
Abiraterone should be used with caution in participants with a history of cardiovascular disease. The safety of abiraterone in participants with left ventricular ejection fraction <50% or New York Heart Association (NYHA) Class III or IV heart failure has not been established. Before treatment with abiraterone, hypertension must be controlled and hypokalaemia must be corrected.

Caution is required in treating participants whose underlying medical conditions might be compromised by increases in blood pressure, hypokalaemia, or fluid retention, e.g. those with heart failure, recent myocardial infarction, or ventricular arrhythmia.

:: Renal impairment
No dose adjustments are required in renal impairment; however, caution is advised if participants develop severe renal impairment as there is limited clinical data in this population. Systemic exposure to abiraterone after a single oral 1000mg dose did not increase in participants with end-stage renal disease on dialysis.

6.2.2.E Abiraterone: Special warnings

:: Overdose
Human experience of overdose with abiraterone is limited. There is no specific antidote to abiraterone. In the event of an overdose, administration of abiraterone should be with-held and general supportive measures undertaken, including monitoring for cardiac arrhythmias, liver function and electrolytes.

6.2.2.F Abiraterone: Interactions (medications)

Details on drug interactions are described in **Table 12** provides a summary on the main interactions.

:: Anti-androgens
Abiraterone is steroid synthesis inhibitor and should not be given together with any other anti-androgens given the risk of toxicity. Cyproterone acetate should be discontinued 10 days and finasteride stopped 48 hours before commencing abiraterone. Concomitant use of any anti-androgen including dutasteride, bicalutamide, flutamide and tamoxifen is not recommended.

Participants receiving or planned for dual androgen blockade (DAB) at randomisation should not continue anti-androgens if randomised to receiving abiraterone.

:: Spironalactone

Spironolactone binds to the androgen receptor, may increase PSA levels and is associated with abiraterone resistance therefore concomitant use is **contraindicated.**

:: Statins and medicinal products associated with myopathy/rhabdomyolysis

Myopathy has occurred in patients treated with abiraterone, typically this occurs when first initiating treatment and resolves when abiraterone is stopped. Caution is recommended in participants receiving concomitant treatments with medicinal products known to be associated with myopathy/rhabdomyolysis e.g. statins.

Table 12: Drugs that may interact with abiraterone

DRUGS WHICH MAY REDUCE ABIRATERONE LEVELS			
**Substrate**	**Clinical Use**	**Drug**	**Recommendation**
CYP3A4 inducers	Anti-epileptics*	Phenytoin<br>Carbamazepine<br>Phenobarbital<br>Primadone (39)	Avoid unless no therapeutic alternative, due to risk of decreased exposure to abiraterone.
	Anti-depressants	St Johns Wart	
	Anti-TB	Rifampicin<br>Rifabutin<br>Rifapentine	
**DRUGS WHICH MAY INCREASE ABIRATERONE LEVELS**			
**Substrate**	**Clinical Use**	**Drug**	**Recommendation**
CYP3A4 inhibitors	Anti-retroviral	Atazanavir<br>Saquinavir<br>Ritonavir<br>Indinavir<br>Nelfanavir	Whilst PK studies of other CYP3A4 inhibitors did not indicate a clinically meaningful interaction, there are no specific studies of assessing an abiraterone - anti-retroviral interaction. If it is not possible to avoid the use of anti-retrovirals it would be prudent to have an awareness of a potential for an interaction and monitor for adverse effects from abiraterone.(40)
	Anti-fungal	Ketoconzole	PK studies suggest no clinically meaningful impact of interaction
**DRUGS WHICH MAY ACCUMULATE WHEN GIVEN WITH ABIRATERONE**			
**Substrate**	**Clinical Use**	**Drug**	**Recommendation**
CYP2D6	Cardiac	Metoprolol<br>Propranolol<br>Propafenone<br>Flecainide	Clinical vigilance required as drug levels may increase with abiraterone use, consider a dose reduction of medicinal products metabolised by CYP2D6.
	Anti-depressants	Desipramine<br>Venlafaxine<br>Citalopram	
	Anti-psychotics	Haloperidol<br>Risperidone	
	Analgesia	Tramadol<br>Codeine<br>Oxycodone	
	Alpha blockers	Tamsulosin (41)	
	Anti-diabetic	Repaglinide (42)<br>Pioglitazone	
	Cough suppressant	Dextromethorpam	

*narrow therapeutic index

6.2.2.G Abiraterone Undesirable Effects

The most common adverse drug reactions observed in the integrated safety data for those participants who received 1000mg abiraterone plus prednisone or prednisolone in clinical studies (n=1,070) were fatigue, arthralgia, peripheral oedema, back pain, bone pain, nausea, constipation, hypokalemia and anaemia.

The adverse events graded as Grade 3 or Grade 4 and which occurred in more than 5% of participants were fatigue, peripheral oedema, anaemia and back pain(38).

In the event of a toxicity not listed below clinicians should use their clinical judgement and take appropriate measures to treat the participant, including interruption of research treatment and/or implementing dose modifications if required. Please update treatment logs with any changes. Contact the MRC CTU for further advice if required.

Table 13: Management of abiraterone-associated hypertension (given alone or with enzalutamide)

TOXICITY EVENT	ACTION
BP repeatedly in range of 120-139/80-89 mmHg	**Continue abiraterone (and enzalutamide).**<br>Management as per investigator.
BP repeatedly in range of 140-159/90-99 mmHg	**Continue abiraterone (and enzalutamide).**<br>Management as per investigator with anti-hypertensive treatment. Follow local guidance for selection of anti-hypertensives but avoid thiazide diuretics to minimise risk of serum potassium derangement. Calcium channel antagonists or beta blockers are often preferred.<br>As with other symptoms of mineralocorticoid excess, consider increasing prednisolone dose to 5mg BID.
BP repeatedly ≥ 160/100 mmHg or life-threatening consequences of hypertension)	**Withhold abiraterone and enzalutamide**.<br>Adjust or add anti-hypertensive medications to mitigate the toxicity. When blood pressure resolves to being predominantly <140/90 or baseline, resume both abiraterone and enzalutamide at full dose with prednisolone 5mg bid.

Record hypertension grade as per CTCAE on the follow-up form if required. If a patient experiences ongoing hypertension that the treating clinician deems clinically concerning then consider referral to cardiologist or hypertension clinic.

Table 14: Management of abiraterone associated hypokalaemia (given alone or with enzalutamide)

TOXICITY EVENT	ACTION
Grade 1<br>(<LLN – 3.0mmol/L)	**Continue abiraterone (and enzalutamide).**<br>Supplement with oral potassium and monitor closely and increase prednisolone dose to 5mg BID.<br>Exclude and manage other causes of hypokalemia.
Grade 2<br>(<LLN – 3.0mmol/L and symptomatic)	**Withhold abiraterone (continue enzalutamide).**<br>Supplement with oral potassium and monitor closely and increase prednisolone dose to 5mg BID.<br>Exclude and manage other causes of hypokalemia.<br>Re-start abiraterone with close monitoring, discontinue if recurs.
Grade 3<br>(<3.0 – 2.5mmol/L)<br>or Grade 4<br>(<2.5mmol/L and life-threatening)	**Permanent discontinuation of abiraterone** and hospitalisation for intravenous potassium replacement and cardiac monitoring.<br>After the return of serum potassium to normal, prednisolone (prednisone in Switzerland) should also be discontinued.<br>The participant can continue on enzalutamide alone. If hypokalaemia persists, consider a dose reduction of enzalutamide to 120mg once a day.

Table 15: Management of abnormal LFTs associated with abiraterone (given alone or with enzalutamide)

TOXICITY EVENT	ACTION
**Grade 1** increases in AST, ALT or bilirubin (e.g. increase in AST or ALT from ULN to 3.0X ULN; increase in total bilirubin from ULN to 1.5X ULN)	**Continue abiraterone (and enzalutamide).**<br>Increase frequency of LFT monitoring to at least weekly, if the investigator judges that the laboratory abnormalities are potentially related to study medication.<br>Providing LFTs are stable for 4 weeks, resume normal LFT monitoring.
**Grade 2** increases in AST, ALT or bilirubin (e.g. increase in AST or ALT to >3.0-5.0X ULN; increase in total bilirubin from >1.5-3.0X ULN)	**Withhold abiraterone, enzalutamide** and all other concomitant medications that are potentially hepatotoxic.<br>Increase frequency of LFT monitoring to at least weekly until the LFTs return to baseline value or Grade 1, when both abiraterone and enzalutamide can be re-started.<br>Enzalutamide can be re-started with no dose reduction.<br>Abiraterone can be re-started with no dose reduction after one episode, providing this resolved within 4 weeks.<br>Dose reduction should be considered if Grade 2 derangements persist or recur; see below.
**Grade 3** increases in AST, ALT or bilirubin (e.g. increase in AST or ALT to >5.0X ULN; increase in total bilirubin to >3.0X ULN),	**Withhold abiraterone and enzalutamide** and all other concomitant medications that are potentially hepatotoxic.<br>Immediately increase the frequency of LFT monitoring to at least weekly until the LFTs return to baseline value or Grade 1.<br>Enzalutamide can be re-started with no dose reduction.<br>Abiraterone can be re-started with dose reduction to 250mg once toxicities resolved to Grade 1 or baseline. This dose can be titrated as per liver function blood tests.
**Grade 4** increases in AST, ALT or bilirubin (e.g. increase in AST or ALT to >20.0X ULN; increase in total bilirubin to >10.0X ULN)	**Immediate discontinuation of abiraterone and enzalutamide.**<br>Increase the frequency of LFT monitoring to at least weekly until the LFTs return to baseline value or Grade 1.<br>Prednisone can then be discontinued and the investigator can consider restarting enzalutamide.<br>Abiraterone should not be re-introduced.
**SCENARIO**	**ACTION**
Recurrent or persistent Grade 2 AST, ALT, or bilirubin derangement	**Withhold abiraterone and enzalutamide** and all other concomitant medications that are potentially hepatotoxic.<br>Once LFTs return to Grade 1 restart abiraterone at 250mg and titrate upwards, guided by weekly blood tests.
Second episode of Grade 3 AST, ALT or bilirubin derangement	**Withhold abiraterone and enzalutamide** and all other concomitant medications that are potentially hepatotoxic.<br>Immediately increase LFT monitoring at least weekly is required, continue until returned to baseline values or Grade 1.<br>Recommence enzalutamide initially. If abiraterone resumption is then considered, resume study treatment with abiraterone dose starting at 250mg and titrate upwards guided by LFTs
Third episode of Grade 3 AST, ALT or bilirubin derangement	Permanently discontinue abiraterone.<br>Prednisone can then be discontinued and the investigator can consider restarting enzalutamide.

An opinion from a hepatologist should be considered if there are any concerns or liver function derangement shows no improvement within 2 weeks of discontinuation of abiraterone.

Table 16: Management of fluid retention/oedema associated with abiraterone (given alone or with enzalutamide)

TOXICITY EVENT	ACTION
Grade 1-2	**Continue abiraterone (and enzalutamide).**<br>Increase prednisolone dose to 5mg bid.
Grade 3-4	**Withhold abiraterone. Enzalutamide can be continued.**<br>Consider addition of mineralocorticoid receptor antagonist eplerenone until resolution of symptoms. When fluid retention/oedema returns to baseline or resolves to ≤Grade 1, resume abiraterone at full dose with prednisone 5mg bid.<br>If symptoms do not resolve abiraterone should not be re-started and enzalutamide should be dose reduced to 120mg per day.

Table 17: Management of diarrhoea (associated with abiraterone or enzalutamide)

TOXICITY EVENT	ACTION
Grade 1-2	**Continue abiraterone and enzalutamide.**<br>Symptomatic management.
Grade 3-4	**Withhold abiraterone**. Enzalutamide can be continued in the first instance.<br>If no improvement from witholding abiraterone alone, reduce dose of enzalutamide to 120mg per day.<br>If still no improvement reduce dose of enzaluatmide to 80mg. If diarrhoea persists despite this (and it is believed symptoms are caused by abiraterone or enzalutamide) we recommend the patient stops trial treatment<br>Once resolved to Grade 1, recommence abiraterone at 750mg per day.

6.2.2.H Abiraterone: Prednisolone (prednisone in Switzerland)

The co-administration of prednisolone (prednisone in Switzerland) 5mg once daily is required whilst receiving abiraterone to prevent secondary mineralocorticoid excess.

Prednisolone should be taken as a single dose with food in the morning. If mineralocorticoid-related toxicities occur (e.g., hypokalaemia, hypertension, peripheral oedema) the prednisolone dose should be reviewed. See Table 13, Table 14 and Table 16 for advice on when an increase to 5mg BID is recommended.

If a participant experiences serious symptoms of Cushing's syndrome (e.g., weight gain, muscle loss) investigators may reduce the steroid dose but participants should be closely monitored for symptoms of secondary mineralocorticoid excess. It should be noted that weight gain and muscle loss are also associated with ADT.

If a participant allocated to receive abiraterone develops only biochemical failure, the responsible clinician may switch from abiraterone + prednisolone 5mg OD to abiraterone + dexamethasone 0.5mg OD.

6.2.3 Research Enzalutamide (Arm J)

Note: recruitment has closed to Arm J (SOC + enzalutamide + abiraterone).

Participants allocated to Arm J will receive enzalutamide in combination with abiraterone, in addition to SOC ADT. For information relating to treatment with abiraterone (and prednisolone), refer to Section 6.2.2.

6.2.3.A Enzalutamide: Clinical particulars – posology and administration

Enzalutamide will be administered as a 160mg oral dose (four capsules), taken together at the same time every day, with or without food.

6.2.3.B Enzalutamide: Clinical particulars - treatment duration

Enzalutamide will be taken for the same duration as the co-administered abiraterone, unless either abiraterone or enzalutamide is stopped for toxicity, in which case the other drug may continue.

Enzalutamide treatment duration is included in Table 9. See Section 7.1.3 for further information on the definition of progression.

6.2.3.C Enzalutamide: Safety monitoring

Safety monitoring for participants receiving research enzalutamide alone.

Please see Table 11 for safety monitoring if enzalutamide given alongside abiraterone. In acute toxicity monitoring requirements may increase – see toxicity tables for more advice in this setting.

Adverse event of interest	Monitoring required	Frequency of monitoring whilst on research enzalutamide ALONE (if abiraterone previously stopped for toxicity) in the trial setting		
		Weeks 0 – 12	Week 12 – 12 months	12 months – end of treatment
Hypertension	Blood pressure*	Every 2 weeks	Monthly	Participants may have the frequency of monitoring reduced to every 3 months if judged appropriate by the investigator.<br><br>Continued monthly monitoring is required for participants if there are concerns related to research enzalutamide causing hypertension.

* Blood pressure may be monitored using documented self-monitoring or via the GP providing this is reviewed at each follow up.

6.2.3.D Enzalutamide: Cautions and contra-indications

:: History of seizures

Caution should be used in administering enzalutamide to participants with a history of seizures or other predisposing factors including, but not limited to, underlying brain injury, stroke, primary brain tumours or brain metastases or alcoholism. In addition, the risk of seizure may be increased in participants receiving concomitant medications that may lower the seizure threshold. Enzalutamide should be permanently discontinued in participants who have a seizure while on treatment.

:: Hepatic impairment

A hepatic impairment study showed that the composite AUC of enzalutamide plus N-desmethyl enzalutamide after administration of a single dose of enzalutamide was similar in patients with

baseline mild, moderate or severe hepatic impairment (Child-Pugh Class A, B or C respectively) relative to patients with normal hepatic function, and no starting dose adjustment is needed (43).

:: Renal impairment

No dose adjustments are required in renal impairment; however, caution is advised if participants develop severe renal impairment as there is limited clinical data in this population.

6.2.3.E Enzalutamide: Special warnings

:: Overdose

There is no antidote for enzalutamide. In the event of an overdose, stop treatment with enzalutamide and initiate general supportive measures taking into consideration the half life of 5.8 days. Participants may be at increased risk of seizures following an overdose.

6.2.3.F Enzalutamide: Interactions (medications)

The full induction potential of enzalutamide may not occur until approximately 1 month after the start of treatment, when steady-state plasma concentrations of enzalutamide are reached, although some induction effects may be apparent earlier. Monitoring for drug interactions should continue for at least the first month of treatment and dose adjustments considered. Given the long half-life of enzalutamide (5.8 days), effects on enzymes may persist for one month or longer after stopping enzalutamide. A gradual dose reduction of the concomitant medicinal product may be necessary when stopping enzalutamide treatment. See for further details on specific drug interactions with enzalutamide.

Details on drug interactions are described in **Table 18** provides a summary on the main interactions.

:: Anti-androgens

Enzalutamide is potent androgen receptor antagonist and should **not** be given together with any other anti-androgens given the risk of toxicity. Cyproterone acetate should be discontinued 10 days and finasteride stopped 48 hours before commencing enzalutamide (+ abiraterone). Concomitant use anti-androgens including dutasteride, bicalutamide, flutamide and tamoxifen are **not recommended**.

Participants receiving or planned for dual androgen blockade (DAB) at randomisation should not continue anti-androgens if randomised to receiving enzalutamide (+abiraterone).

Table 18: Drugs which may interact with enzalutamide

DRUGS WHICH MAY INCREASE ENZALUTAMIDE LEVELS			
**Substrate**	**Clinical Use**	**Drug**	**Recommendation**
CYP2C8 inhibitors	Lipid-lowering	Gemfibrozil	Avoid, if no alternatives, reduce enzalutamide dose to 80mg
**DRUGS WHICH MAY DECREASE ENZALUTAMIDE LEVELS**			
**Substrate**	**Clinical Use**	**Drug**	**Recommendation**
CYP2C8 inducers	Anti-tuberculosis	Rifampicin<br>Rifabutin	Avoid if possible. If the therapeutic effect of these medications is of large importance to the patient, and dose adjustments are not easily performed based on monitoring of efficacy or plasma concentrations of these medicinal products, use with caution, due to risk of decreased exposure to enzalutamide.
CYP3A4 inducers	Anti-epileptics	Phenytoin<br>Carbamazepine<br>Phenobarbital	
	Anti-depressant	St John's wort	
	Anti-retrovirals	Atazanavir<br>Saquinavir<br>Ritonavir<br>Indinavir<br>Nelfanavir	Avoid if possible. If the therapeutic effect of these medications is of large importance to the patient, and dose adjustments are not easily performed based on monitoring of efficacy or plasma concentrations of these medicinal products, use with caution. This is due to risk of decreased exposure to enzalutamide and increased risk of toxicity from the anti-retroviral medication.
**ENZALUTAMIDE MAY REDUCE DRUG LEVELS**			
**Substrate**	**Clinical Use**	**Drug**	**Recommendation**
CYP2C19	Gastric protection	Omeprazole	Omeprazole AUC reduced by 70% Consider increasing dose of omeprazole for same therapeutic effect
CYP3A4	Analgesia	Fentanyl*<br>Alfentanil*<br>Tramadol	Consider alternatives as clinical effect may be reduced, if no therapeutic alternative monitor closely
	Immunosuppressants	Sirolimus*<br>Tacrolimus*<br>Cyclosporine*	
	Anti-migraine	Ergotamine	
	Cardiac	Nifedipine<br>Ivabradine	
CYP2C9	Anti-epileptics	Phenytoin*	Contra-indicated
	Anti-coagulants	Warfarin*	Warfarin AUC reduced by 56% Consider switching to low molecular heparin, increase INR monitoring if this is not possible
**DRUGS WHICH MAY ACCUMULATE WHEN GIVEN WITH ENZALUTAMIDE**			
**Substrate**	**Clinical Use**	**Drug**	**Recommendation**
p-gp		Colchicine*<br>Dabigatran*<br>Digoxin*	Consider alternatives, if no therapeutic alternative monitor closely

*narrow therapeutic index

6.2.3.G Enzalutamide: Undesirable effects

Please refer to section 6.2.2.G for management of hypokalaemia, deranged LFTs, hypertension, fluid retention and diarrhoea that occurs whilst on enzalutamide in combination with abiraterone.

The safety monitoring and toxicity management plan described below takes into account AEs based on the reported clinical safety data of abiraterone and enzalutamide given separately. There are limited reported data on the safety and toxicity of the combination of enzalutamide and abiraterone; however, the recommendations summarised here have been updated in light of the experience gained in STAMPEDE as recommended by the STAMPEDE TMG.

Additional toxicity to be aware of relevant to enzalutamide alone include the following:

Table 19: Management of seizure whilst on enzalutamide treatment

TOXICITY EVENT	ACTION
Seizure (any Grade)	If any participant suffers a seizure whilst on treatment, enzalutamide should be permanently discontinued immediately. Abiraterone and prednisolone can be continued providing there are no abiraterone-specific toxicities.

Table 20: Management of arthralgia & muscle pain (associated with enzalutamide)

TOXICITY EVENT	ACTION
Grade 1-2	Symptomatic management
Grade 3-4	Reduce dose of enzalutamide to 120 mg /day

Table 21: Management of fatigue (associated with enzalutamide)

TOXICITY EVENT	ACTION
Grade 1-2	Consider a dose reduction to 120 mg/day
Grade 3	**Pause enzalutamide** for 1 week or until the toxicity grade improves to Grade 2 or lower severity.<br>Re-start at a reduced dose (120mg/day or 80mg/day), dose chosen to restart is at the treating clinicians discretion.

6.2.4 Research Metformin (Arm K)

Note: General recruitment has closed to Arm K. From protocol 21 only participants to be involved in the metabolic sub-study can be randomised to Arm K.

Participants allocated to Arm K will receive metformin, in addition to SOC treatments. All potential SOC systemic treatment options are suitable for combination with metformin.

6.2.4.A Metformin Clinical particulars – posology and administration

For all participants allocated to Arm K, metformin should start as soon as possible after randomisation and ideally within a maximum of 12 weeks.

Metformin will be given as a daily dose in addition to SOC treatment. The target dose is **850mg Std BID.**

The starting dose for metformin is 850mg Std OD. If tolerated, this should be increased to the target dose after 4-6 weeks i.e. at the first follow-up visit.

Metformin should be taken around the same time each day and treatment tolerance is best if taken with or after food. For twice daily dosing, the minimum time between doses should be 8 hours; doses should not be taken closer together if forgotten or missed.

Providing participants have a sufficient supply of STAMPEDE-labelled IMP metformin tablets, a telephone consultation may be sufficient to assess tolerance and give advice regarding dose modification in order to limit hospital visits. This interaction **must be documented** in the medical records.

:: Metformin dose modifications

If metformin target dose of 850mg Std BID is not well tolerated, the dose reductions listed in Table 22 could be implemented.

Note: Sustained release formulations (SR) can sometimes be better tolerated with less GI side-effects than the standard or immediate release formulations (Std). Both drugs provide similar exposure at a given daily dose.

Table 22: Management of metformin related GI-toxicity

<table>
<tr><td colspan="5">GRADE 1 OR 2 TOXICITY:
ASSUMES STARTING ON 850MG STD BD (1,700MG TOTAL DAILY DOSE) DOSE.
IF TOXICITY OCCURS ON LOWER DOSE – DROP TO THE NEXT DOSE LEVEL DOWN</td></tr>
<tr><td colspan="5">IF ONGOING TOXICITY</td></tr>
<tr><td>ACTION 1:</td><td>ACTION 1A:
DOSE REDUCTION LEVEL 1
(OPTIONS)</td><td>ACTION 1B:
DOSE ON RESTART AFTER PAUSE
(OPTIONS)</td><td>ACTION 2:
DOSE REDUCTION LEVEL 2
(OPTIONS)</td><td>ACTION 3:
STOP TREATMENT</td></tr>
<tr><td rowspan="2">Ensure metformin is taken with or after food and consider dose reduction
OR
Ensure metformin is taken with or after food and consider 1-2 week treatment pause.</td><td>a) 750mg SR BID (1500mg total daily dose)
OR
b) 500mg SR BID (1000mg total daily dose)
OR
c) 500mg Std BID (1000mg total daily dose)</td><td>a) 850mg Std OD (850mg total daily dose)
OR
b) 500mg SR OD (500mg total daily dose)</td><td rowspan="2">a) 850mg Std OD (850mg total daily dose)
OR
b) 750mg SR OD (750mg total daily dose)
OR
c) 500mg SR OD (500mg total daily dose)
OR
d) 500mg Std OD (500 mg total daily dose)</td><td rowspan="2">If toxicity recurrs after two dose reductions, we recommend stopping treatment</td></tr>
<tr><td colspan="2">Re-attempt a dose escalation after 1-2 month aiming to continue at the maximum tolerated dose</td></tr>
</table>

:: Treatment breaks
It is anticipated that metformin treatment will be paused for approximately 72 hours around the time of contrast-enhanced CT scans (see **Table 24**) and may need to be paused during episodes of inter-current illness.

- If metformin is paused for 6 days or less this information does not need to be recorded and no additional action is needed.
- Treatment pauses of ≥7 days must be recorded by updating the Metformin Treatment Log CRF.
- If metformin treatment is paused for more than 2 weeks, investigators may consider re-starting at 850mg Std once daily for the first 4 weeks before escalating to full dose providing tolerance is acceptable.
- If treatment is paused for >3 months or >50% of doses are missed for any reason, it is at the discretion of site investigators as to whether it is appropriate to restart trial treatment with metformin. Please ensure treatment pauses are recorded and feel free to contact the CTU for any advice if required.

6.2.4.B Metformin: Clinical particulars – treatment duration
Metformin treatment duration is included in **Table 9**.

6.2.4.C Metformin: Safety monitoring
Routine safety monitoring frequency is described in **Table 23**.

Table 23: Renal function monitoring required whilst on treatment with metformin

RENAL FUNCTION:	FREQUENCY OF MONITORING:
• Stable renal function **AND**<br>• eGFR ≥45ml/min/1.73m^2	6 monthly
• Risk of deteriorating renal function **AND/OR**<br>• eGFR falls to >30 and <45 ml/min/1.73m^2	At least 3 monthly (44)<br>*NB: Max dose is 1000mg per 24 hours in this setting*
• eGFR falls to ≤30ml /min/1.73m^2	Metformin should be paused*

*Should the Site Investigator decide the decline in renal function to ≤30ml /min/1.73m^2 is irreversible then metformin must be permanently stopped

6.2.4.D Metformin: Cautions and contraindications
:: Renal impairment
Metformin is not nephrotoxic, but is exclusively excreted by the kidneys. Therefore treatment should only be started in participants with stable renal function. Metformin should be only started when the GFR ≥45ml/min/1.73m^2, as per the metformin comparison-specific eligiblity criteria in section **4.6.1**.

Additional renal monitoring is required in any participant at risk of deteriorating renal function. In line with published prescribing recommendations, if the GFR falls to between 30-45 ml/min/1.73m^2 a **dose reduction** is required to ensure the maximum 24hr dose is 1000mg or less and monitoring of renal function is required at least 3 monthly (44).

Metformin should be **permanently stopped** if the GFR falls to ≤30ml/min/1.73m^2 and is irreversible.

See **Table 24** for situations when metformin treatment should be paused due to the risk of deteriorating renal function.

Table 24: Situations when metformin treatment should be paused due to risk of deteriorating renal function

SITUATIONS	RISK FACTOR
Iodinated contrast agents	Metformin should be paused for 24 hours prior to receiving contrast and re-started 48 hours post-administration(45).
Anaesthesia (peridural; spinal or general)	Pause metformin 48 hours prior to procedure and re-start no earlier than 48 hours following procedure, providing oral intake re-established and renal function is stable and at baseline.
Surgery	Pause metformin 48 hours prior to procedure and re-start no earlier than 48 hours following procedure, providing oral intake re-established and renal function is stable and at baseline.
Dehydration<br>e.g. nausea, vomiting or diarrhoea	Pause metformin and re-start only when oral intake is re-established and renal function is stable and at baseline.
Obstructive uropathy<br>e.g. urinary retention or ureteric obstruction	Pause metformin and re-start only when renal function confirmed to be stable and at baseline.

6.2.4.E Metformin: Special warnings

:: Metformin overdose

Hypoglycaemia has not been reported with even significant metformin overdoses although lactic acidosis has occurred in such circumstances. Participants should be urgently assessed in the event of an overdose and hospital admission considered. The management of metformin overdoses should be as per standard clinical care by the local team. The most effective way to remove lactate and metformin is haemodialysis.

6.2.4.F Metformin: Interactions (medications)

Caution is needed when initiating potential nephrotoxic drugs as metformin is renally excreted and therefore may accumulate if renal function deteriorates. Please refer to **Table 25** for more information on drugs which may require additional monitoring of renal function, at the discretion of the treating clinician.

Metformin does **not** interact with any of the other treatments for prostate cancer and **should be continued** during all further treatments given for disease progression, **provided clinicians feel it remains in the participants best interests**, as per **Table 9**.

As metformin is being given as an IMP in the context of a clinical trial, continued use will not be permitted if participants participate in other interventional clinical trials for prostate cancer (i.e. CRPC setting).

Table 25: Drugs which may require additional monitoring of renal function

Clinical use	Drug	Recommendation
Anti-hypertensives and other cardiac disease	ACE inhibitors/angiotensin II receptor blockers e.g. ramipril, lisinopril, irbesartan	Increased frequency of renal function monitoring until confirmed to be stable
	Diuretics e.g. furosemide, bumetanide	
Antibiotics	Aminoglycoside antibiotics e.g. gentamicin or amikacin	Pause metformin during treatment Re-start once treatment complete
Analgesia	NSAIDs e.g. Ibuprofen, diclofenac, naproxen	Avoid if possible If used increased frequency of renal function monitoring is required until confirmed to be stable

6.2.4.G Metformin: Undesirable effects

:: Gastrointestinal disturbance

Gastrointestinal disturbances are very common with metformin and include nausea, vomiting, diarrhoea, abdominal pain and loss of appetite. These are most common when first starting treatment (occur in >1/10 individuals).

If toxicities occur, a dose reduction and/or a switch to a sustained release (SR) preparation, if available, is recommended (see **Table 22**).

Other possible metformin-related toxicites included taste disturbance, skin reactions and B12 deficiency resulting in megaloblastic anaemia. If a participant becomes anaemic whilst taking metformin, investigators should consider measuring haematinics, including vitamin B12, and replace if deficient.

:: Lactic acidosis

Lactic acidosis is a very rare (3/100,000 patient years), but serious metabolic consequence. Reported cases have occurred primarily in diabetic patients with significant renal impairment who are also dehydrated. It is unclear whether this is due to the underlying diabetes or metformin. This is supported by a meta-analysis demonstrating similar rates of lactic acidosis in people with diabetes taking metformin compared with diabetic participants not taking metformin (46). This evidence suggests this side effect may be a complication of diabetes and may not be associated with metformin treatment.

The risk factors for lactic acidosis are: renal impairment, prolonged fasting or malnutrition, excessive alcohol intake, hepatic insufficiency or any condition associated with hypoxia e.g. cardiac or respiratory failure or circulatory shock due to any cause.

The risk of lactic acidosis should be considered in the event of non-specific symptoms such as muscle cramps, abdominal pain and/or severe weakness or lethargy. Any participant with a suspected metabolic acidosis requires **immediate discontinuation of metformin** and evaluation. Lactic acidosis is characterised by metabolic acidosis (decreased blood pH, high lactate above 5mmol/L and an increased anion gap and lactate/pyruvate ratio). The most effective way to remove lactate and metformin is haemodialysis.

6.2.5 Research Transdermal Oestradiol (Arm L)

:: Timeframe for commencing treatment
For all participants allocated to transdermal oestradiol, treatment should start as soon as possible after randomisation (and ideally within 1 week after randomisation). It is not necessary to wait for completion of the 4-week (or 1-month) duration of the LHRHa injection if this was previously given prior to randomisation. For those prescribed bicalutamide or flutamide prior to randomisation, this treatment should be discontinued before treatment with transdermal oestradiol can commence (no washout period is needed).

:: Transdermal oestradiol with SOC treatments
If SOC docetaxel has not been started before randomisation, it is recommended that it is started, for suitable participants, once established on transdermal oestradiol for around 4 weeks, when most participants are likely to have completed the induction period).

From protocol v21.0 onwards, participants randomised to the transdermal oestradiol comparison are also eligible to receive SOC abiraterone, enzalutamide or apalutamide in the upfront setting, as an alternative to docetaxel (see **Section 6.1.4**). However, the use of enzalutamide, abiraterone or apalutamide *prior to starting treatment* with transdermal oestradiol patches is not authorised (see **Section 4.3.3**).

Participants randomised to receive transdermal oestradiol may also receive SOC radiotherapy (see Section **6.1.2**) as clinically appropriate, as has been done in the PATCH trial.

6.2.5.A Transdermal oestradiol: Clinical particulars – posology and administration

Transdermal oestradiol is delivered either as Progynova TS 100 mcg/24 hours or since protocol v21.0 as Femseven 100 mcg/24hours transcutaneous oestradiol patches. Administration should proceed according to the following dose regimen which has been shown within the PATCH trial to be sufficient for achieving castrate levels of testosterone.

The changing of patch brand should be avoided unless absolutely necessary and should only be done following discussion with the CTU trial team.

Other type of patches should only be used in expectional circumstances and after discussion with the CTU team, as we currently do not have sufficient pharmacokinetic and clinical data to recommend the use of other types of patches.

6.2.5.B :: Induction regimen

Four transdermal transdermal oestradiol patches to be changed twice weekly (e.g. Monday and Thursday) for four weeks. A confirmatory testosterone and oestradiol sample should be taken at 4 weeks with the sample drawn the **day before** the patches are changed.

6.2.5.C :: Maintenance regimen

If the participant has achieved a testosterone value of ≤1.7nmol/L and has an oestradiol level >300pmol/L at 4 weeks, then treatment may be changed to a **maintenance regimen** of **three** patches changed twice weekly. However, current observations from the PATCH trial suggest castrate levels of testosterone are typically achieved with a plasma oestradiol level ≥500pmol/L, and sites can opt to wait until oestradiol reaches this level before switching if they prefer.

If a participant's testosterone is >1.7nmol/L or the oestradiol level is <300pmol/L at four weeks then they should remain on the induction regimen for another 4 week period, with monitoring of testosterone and oestradiol samples taken at around the week 8 time point, the day before patches

are changed. Once the participant achieves a castrate level of testosterone ≤1.7nmol/L and an oestradiol level of ≥300pmol/L, they can be switched to the maintenance regimen.

It is expected that participants remain on the prescribed dose, and any potential dose modifications other than those indicated in **Section 6.2.5** should be first discussed with the CTU team.

6.2.5.D :: Administration guidelines

Consecutive patches should be applied to different sites. It is recommended that patches are placed on dry, intact and hairless skin and on areas where little wrinkling occurs, at the following sites only:

- Shoulder girdle
- Upper body
- Hip
- Abdomen
- Back
- Upper arms
- Buttocks

Patches should not be placed on or near the breast area, or on areas of the body where there are large amounts of subcutaneous fat, particularly around the abdomen, as this could affect absorption. Please note that these recommendations are mainly based on studies in women using the patches.

To apply the patch, remove the protective liner and press on to the skin immediately, holding for at least 30 seconds to ensure proper adhesion. If necessary, tape can be used to fix the patch in place. If applied correctly, the participant can bath or shower as normal; however, the patches might come off in very hot water or in a sauna.

Prior treatment start, participants should be provided with the STAMPEDE (Arm L) Study Hormone Patch Application Information for Participants to promote good treatment compliance.

6.2.5.E Transdermal oestradiol: Monitoring Hormone Levels

During enrolment of the first cohort of participants starting treatment with a combination of abiraterone, enzalutamide or apalutamide alongside transdermal oestradiol, the CTU will monitor the changing testosterone, oestradiol and PSA levels in real time. This is to ensure that the combination of treatment does not impact the efficacy of the transdermal oestradiol patches. **Therefore please send the updated hormone results log to the CTU urgently following any blood test in the first 3 months to facilitate this safety analysis.**

Oestradiol and testosterone levels should continue to be monitored throughout follow-up, while the participant remains on transdermal oestradiol treatment, to assess for evidence of compliance and to also ensure the participant is on the appropriate dose. **Table 2** describes when these values are required, noting also that the samples can be taken at the same time as scheduled PSA measurements.

Scenarios when additional oestradiol and testosterone monitoring is required are given below.

:: Oestradiol <300pmol/L or >2000pmol/L or testosterone >1.7nmol/L while on the maintenance regime

A repeat blood test should be carried out within 4 weeks if, at any time, the participant's oestradiol level is found to be <300pmol/L or >2000pmol/L or the testosterone level is >1.7nmol/L while on the maintenance regime, with particular attention paid to the day that the patches are changed

compared to when the blood sample is drawn (should be the day before changing patches). If the participant continues to have out of range oestradiol levels, and/or persistent testosterone >1.7nmol/L, then the CTU team should be contacted for advice.

:: Change of maintenance patch dose
If the maintenance patch dose is changed at any time (for example, reducing from 3 to 2 patches changed twice weekly), then additional oestradiol and testosterone tests are required around 4 weeks after dose modification.

:: Change of patch brand
The changing of patch brand should be avoided unless absolutely necessary (see **Section 6.2.5.A**) but if advised by the CTU trial team, then additional oestradiol, testosterone and PSA tests are required following the change (see **Table 26**). It is important that participants are then monitored in real-time during this initial period, with the CTU team to be contacted if the hormone results are out of range as it may be necessary to modify the dose regimen.

Table 26: Additional assessments required following change of maintenance patch dose or brand

Assessments required	Weeks from dose modification or change in patch brand*			
	Week 0 (Prior to change)	Week 4 (Post change)	Week 8 (Post change)	Week 12 (Post change)
	**Change of maintenance dose**			
Oestradiol<br>Testosterone<br>PSA	X	X		
	**Change of patch brand**			
Oestradiol,<br>Testosterone<br>PSA	X	X	X	X**

* These additional tests are timed from the day of dose modification or day of starting new patch brand. However, if the post-change tests coincide within 1 week of scheduled tests (see **Table 2**), it is not necessary to repeat the tests.

** Dependent on prior results, 12 week test may be requested by CTU trial team

6.2.5.F Transdermal oestradiol: Cautions and contraindications

Tamoxifen should not be prescribed for participants receiving transdermal oestradiol.

6.2.5.G Transdermal oestradiol: Special warnings

If a participant has a cardiovascular event (see **Section 7.1.4.A**), discontinuation of treatment with transdermal oestradiol may be considered at the discretion of the treating clinician.

6.2.5.H Transdermal oestradiol: Interactions (medications)

The metabolism of oestrogens may be increased by concomitant use of substances known to induce drug metabolising enzymes, specifically CYP450 enzymes. However, with transdermal administration, the first-pass effect in the liver is avoided and, thus, transdermally applied oestrogens might be less affected than oral hormones by enzyme inducers. Oestradiol levels are already monitored as part of trial follow-up while participants are on transdermal oestradiol, therefore no additional monitoring is required when combining enzyme inducers with transdermal oestradiol.

6.2.5.I Transdermal oestradiol: Undesirable effects

Dermatitis can be a common side-effect of using the patches, especially in the induction period, which can usually be controlled by alternating the site of patch application. Participants should be advised that if patches become dislodged they should not put on extra patches, but apply their next set of patches when they are next due to be applied.

Prophylactic irradiation of the breast area, shown to reduce risk of gynaecomastia is permitted: a single fraction of 8Gy is recommended preferably before treatment with transdermal oestradiol (47).

6.3 CONCOMITANT TREATMENTS

All concomitant medications should be continued throughout the trial unless the responsible clinician decides otherwise, or there is a potential interaction with the trial treatment, in which case it is the responsibility of the responsible clinician to take the advised action. Please refer to each individual trial treatment section to see a list of drugs which may potentially interact.

6.3.1.A Data collection on concomitant treatments for participants in arms A, G, H, J, K, L

Long-term (>6 months) use of the following concomitant medications of classes of interest is collected:

- Statins
- Metformin (except as Arm K trial treatment)
- Aspirin
- Bisphosphonates or denosumab

This information is of interest both in terms of baseline use and ongoing use through the trial; as such it should be recorded on the Randomisation CRF and will be collected at each follow-up assessment (see **Table 1** and **Table 2**).

6.4 TRIAL PRODUCTS

Details of the procedures for obtaining the drugs within the trial, dispensing and disposal of unused drug are given in **STAMPEDE Pharmacy Information Sheet**. Arrangements for free or discounted drugs are given in the Finance section (**Section 15**).

6.5 TREATMENT DATA COLLECTION

Data will be recorded on case report forms (CRFs); the original should be sent to CTU for data entry and a copy kept at the local site. Current versions of all CRFs can be found on the trial website (http://www.stampedetrial.org/) and sites will be notified of any changes throughout the course of the trial. The type of data to be recorded is detailed in the Assessments and Procedures section (**Section 7**).

6.6 MEASURES OF COMPLIANCE/ADHERENCE

Date of treatment, dose, delays and reasons for delays or dose modifications of all trial treatments will be recorded. The estimated number of abiraterone tablets, enzalutamide capsules or metformin tablets taken in a given time period will also be recorded as well as any dose reductions. See **Table 28** for a description of the treatment logs.

Oestradiol levels will be collected for participants in the transdermal oestradiol arm and used to assess compliance to treatment (see **Section 6.2.5.C**).

Evidence of compliance with safety monitoring is required for participants on research abiraterone and research enzalutamide e.g. potassium and LFTs, or metformin treatment e.g. renal function, as described in sections **6.2.2.C**, **6.2.3.C**, and **6.2.4.C**. Site investigators should document in the participant's medical records the date of the blood test or review of blood pressure measurements and confirmation that the results were known to be within acceptable limits and if not, the toxicity should be graded according to CTCAE V4.0 and the action described. This should be available at on-site monitoring visits and used to verify the information provided on the follow-up CRF and treatment logs.

Note, safety monitoring for SOC abiraterone, enzalutamide and apalutamide is as per local practice and compliance data is not required by the trial.

7 ASSESSMENTS AND PROCEDURES

7.1 SCHEDULE FOR ASSESSMENTS

7.1.1 Follow-up Schedules

An individualised form with a follow-up schedule will be provided for each randomised participant. Which follow-up schedule applies depends on which comparison the participant was randomised to as summarised in Table 27.

Table 27: Summary of follow-up schedules by participant group

COMPARISON	PARTICIPANT DETAILS	FOLLOW-UP SCHEDULE
"Original"	Arms B, C, D, E, F and Arm A recruited between trial start (2005) and 15-Nov-2011	Active follow-up discontinued in Q3 2018
"Abiraterone"	Arms A and G randomised between 15-Nov-2011 and 17-Jan-2014	See **Table 1**
"Abiraterone and enzalutamide"	Arms A and J randomised between 29-Jul-2014 and 31-March 2016	See **Table 1**
"M1|RT"	Arms A and H randomised between 22-Jan-2013 and 02-Sep-2016	See **Table 1**
"Metformin"	Arms A and K randomised since 05-Sep-2016	See **Table 2**
"Transdermal oestradiol"	Arms A and L randomised since 20-Jun-2017	See **Table 2**

7.1.2 PSA, Testosterone And Oestradiol Measurements

All participants should have PSA measured prior to starting ADT and at every subsequent trial follow-up visit, regardless of allocated treatment arm. For participants who do not have a scheduled hospital visit, it is acceptable for arrangements to be made for blood samples to be drawn at their GP surgery.

For arm L participants, oestradiol and testosterone levels should continue to be monitored while the participant remains on transdermal oestradiol treatment. The first follow-up visit post-randomisation can be scheduled at 4 instead of 6 weeks to coincide with the 4-week hormone tests (see **Section 6.2.5**). These samples could be taken at the same time as the PSA tests, unless additional tests are required as detailed in **Section 6.2.5**. Blood samples should to be taken the day before the oestradiol patches are changed, to allow consistent measurements of testosterone and oestradiol with respect to the pharmacokinetic profile of the patches.

7.1.3 Assessment Of Treatment Failure (Definition Of Progression)

All participants should have baseline radiological examinations as detailed in **Section 4.2.1**. Participants are not routinely assessed for response. However, in order that objective progression can be assessed, it is recommended to have imaging taken at time of best response as judged by the treating clinician. The frequency of imaging is at the discretion of the treating clinician.

The following outcomes should be reported on the Progression log:

- Biochemical failure

- Local progression
- Lymph node progression
- Progression or development of new distant metastases, defined as lymph nodes outside the pelvis, bone or organ involvement
- Skeletal-related events confirmed as progression (see below)

7.1.3.A Biochemical Failure

For the purposes of the STAMPEDE trial, a unique threshold PSA value for biochemical failure is calculated, referred to as the **PSA progression value**.

This value is derived for each participant based on their **PSA nadir,** defined as the lowest PSA value reported between *randomisation* and 24 weeks on trial. Please refer to the PSA progression value calculator on the STAMPEDE website.

The exact method for deriving the progression value for a participant depends on the value of their PSA nadir, and how this compares to their pre-treatment PSA value (i.e. the extent of the fall in PSA from the starting point).

The PSA progression values are shown within **Figure 3**, they are calculated in one of three ways:

A. If the lowest recorded PSA value in the 24 weeks following randomisation is more than 4ng/ml and more than 50% of the pre-treatment PSA level then the participant fulfils the criteria for immediate treatment failure.

B. For participants whose PSA nadir in the 24 weeks following randomisation is less than or equal to 50% of the pre-treatment PSA level but remains above 4ng/ml, biochemical failure will be defined as a rise of 50% above the nadir level.

C. For participants whose PSA nadir is less than or equal to 4ng/ml, biochemical failure is defined as at least a 50% rise above the nadir value that is also above 4ng/ml.

Figure 3 PSA progression example scenarios

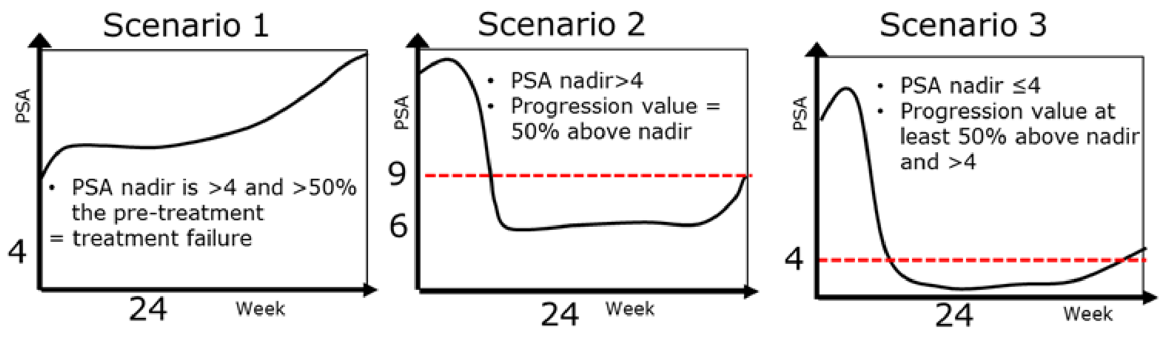

Confirming biochemical failure: the timing of assessments needs to be considered because spurious rises in PSA can occur e.g. following procedures involving the urinary tract. For this reason, any isolated rise in PSA should be confirmed before reporting biochemical failure.

In the case that the raised PSA value reaches the progression value, a confirmatory PSA test should be performed between one week and 3 months later. Biochemical failure is confirmed if the second value is around the same level or higher i.e. the trend is confirmed. The date of PSA progression

should be provided on the Progression Log as the date of the **first** raised PSA that fulfilled the trial definition of progression. Only the first instance of biochemical failure needs to be reported.

A confirmatory PSA is not required if there are other signs of progression e.g. progression of cancer related symptoms (clinical progression) or new radiological progression.

Second-line treatment commenced specifically for biochemical failure should not start until the trial definition for biochemical failure has been met. However, if second-line treatment does start before the trial definition is met then report the closest PSA value prior to the treatment start date as the progression value. This is not required if second-line treatment is being started for other signs of progression e.g. clinical or radiological.

Testosterone levels: are only required when reporting biochemical progression whilst receiving hormone treatment to confirm the diagnosis of castrate-resistant prostate cancer. Testosterone levels are not required when reporting biochemical progression in participants not receiving hormone therapy e.g. participants who presented with non-metastatic disease have relapsed following completion of treatment.

7.1.3.B Local, Lymph Node And Metastatic Failure

For each of local, lymph node and distant metastases progression, **both of** the following should be reported:

- Date of first clinical/symptomatic progression
- Date of first objective/radiological progression

7.1.3.C Skeletal-related Events

- Pathological Fracture
- Spinal cord compression
- Requirement for RT to bone (e.g. for pain or impending fracture)
- Requirement for surgery (e.g. for prevention or management of fracture)

SREs are a secondary outcome measure and a disease event of interest. SREs may represent disease progression but can also occur due to treatment-related effects e.g. osteoporotic fracture due to treatment-related bone-mineral density loss. All SREs should be recorded on the Follow-up form.

All SREs should be investigated further to establish whether or not the participant has progressed and, if confirmed as progression, a Progression Log should be completed to record this along with an Additional Treatment Log to give details of any treatment received (e.g. radiotherapy or surgical). The summary of timing of Case Report Forms can be viewed in Table 28 and Table 29.

7.1.3.D Objective/Radiological progression

Investigator determined radiological progression should be reported. For specific comparisons it may be necessary to centrally review baseline and progression scans e.g. CT scans and bone scans. Requests for scans will be made if and when these are required for a proportion of relevant participants and processes put in place for electronic transfer and site reimbursement.

7.1.4 Additional Metabolic And Cardiovascular Outcomes

A number of metabolic and cardiovascular (CVS) outcomes are being assessed in the “metformin comparison” and “transdermal oestradiol comparison” as outlined below. From protocol v17.0 onwards, a metabolic profile (lipids, glucose and HbA1c) will be measured for all participants randomised from 05-Sep-2016 onwards to capture data on metabolic and cardiovascular outcomes for both comparisons. This is collected to improve the understanding of the metabolic impacts of

ADT, and in those participants on metformin, whether any of these effects are mitigated. As this is independent of progression, testing continues post progression. See **Table 2** for a schedule of assessments, please note it is permitted to obtain these measurements within 12 weeks of the scheduled follow-up visit. The summary of timing of Case Report Forms can be viewed in **Table 28** and **Table 29**.

7.1.4.A Cardiovascular Outcomes: Transdermal Oestradiol Comparison

Cardiovascular morbidity and mortality was the primary outcome measure for the first stage in the PATCH trial (completed in 2010), which showed similar rates of CVS events in participants receiving transdermal oestradiol compared to those receiving LHRHa injections (31). These results have been confirmed by longer-term data within the trial (see **Appendix I**). Continued monitoring of CVS outcomes will be undertaken by the PATCH IDMC for both the PATCH trial, as well as for the participants in STAMPEDE allocated to transdermal oestradiol together with their contemporaneous controls.

While Arm L participants are undergoing treatment with transdermal oestradiol, the majority of these CVS events will fall under the definitions of Serious Adverse Events (see **Section 11**). Once a participant has a cardiovascular event, the discontinuation of treatment with transdermal oestradiol may be considered at the discretion of the treating clinician and the participant switched to standard of care hormone therapy.

An increased risk of venous thromboembolism has been observed when docetaxel is used in combination with certain agents for the treatment of prostate cancer. Therefore, the rate of VTE and CVS events will be closely monitored among participants within Arm L who are receiving docetaxel as part of their first-line treatment. For more details see **Appendix I**. However, within the PATCH trial (based on data up to 17-Sep-2017), no cardiovascular endpoint events had been reported among participants on transdermal oestradiol receiving upfront docetaxel.

7.1.5 Additional Safety Assessments

The comparison specific follow-up schedules are summarised in **Table 1** and **Table 2.**
These summarise all the required additional safety assessments that are required whilst participants are receiving research treatments: abiraterone, enzalutamide, metformin and transdermal oestradiol. All safety assessments are required until research treatments have been permanently stopped for more than 30 days.

The summary of the timing of Case Report Forms also can be viewed in **Table 28** and **Table 29**.

7.1.5.A Additional Safety Assessment: Research Abiraterone with or without Enzalutamide

Due to the risk of liver toxicity and secondary hyperaldosteronism with abiraterone, all participants require regular monitoring of **potassium, liver function tests and blood pressure** whilst receiving research abiraterone with or without enzalutamide. Refer to **Section 6.2.2.C** for required frequency of monitoring. Participants from Arm J who remain on enzalutamide alone require regular monitoring of blood pressure. Refer to **Section 6.2.3.C** for required frequency of monitoring.

Confirmation that potassium and liver functions test have been performed regularly and blood pressure control reviewed will be required at each follow-up visit. Any abnormalities should be graded according to CTCAE version 4.03 and recorded on the toxicity section of the follow-up CRF; any abnormalities fulfilling the criteria for a SAE (e.g. requiring hospital admission) should also be reported on a SAE CRF (see **Section 11**).

Please note, the protocol guidance relates to research abiraterone i.e. treatment received by participants allocated to arms G and J. This may be used as a guide when using SOC abiraterone, but investigators should adhere to local practice.

7.1.5.B Additional Safety Assessment: Enzalutamide

Participants in Arm J who stop abiraterone but continue with enzalutamide require ongoing monitoring of **blood pressure every 3 months** whilst receiving research enzalutamide. Safety blood tests are not routinely required for patients who remain on enzalutamide alone but can be completed at the discretion of the treating clinician.

7.1.5.C Additional Safety Assessment: Metformin

Participants with normal and stable renal function receiving metformin require monitoring of **renal function (U&Es) every 6 months** whilst on treatment. More frequent monitoring is required in participants with declining renal function, or when initiating new potentially nephrotoxic medications or at times of intercurrent illness (see **Section 6.2.4.C**). Changes in renal function (eGFR, graded according to CTCAEv.4) are recorded on the Follow-up CRF. It is acceptable for blood sampling to be arranged via the GP at the participant's home or local hospital.

7.1.5.D Additional Safety Assessment: Transdermal Oestradiol

Hormone levels are monitored while participants are on transdermal oestradiol, and if oestradiol levels are found to be >2000pmol/L with confirmed repeat test, please contact CTU for advice (see **Section 6.2.5.B**).

Real-time monitoring of testosterone, oestradiol and PSA levels is required in the first cohort of participants starting treatment with a combination of abiraterone, enzalutamide, or apalutamide alongside transdermal oestradiol (see **Section 6.2.5.C**).

7.2 DATA COLLECTION PROCEDURES

Treatment-related data are collected on Treatment Specific Logs. It is important that **all** treatments given for progressive disease are recorded on the Additional Treatment Log. The summary of timing of Case Report Forms can be viewed in **Table 29.**

7.2.1 Data Collection For SOC Hormone Therapy

Information relating to SOC hormone therapy is recorded on the SOC Hormone Therapy Log, unless it is a treatment change for disease progression. The SOC Hormone Therapy Log should be updated with any changes in long-term hormone therapy e.g. if anti-androgens are being added to LHRHa for dual androgen blockade in the **absence of progression**. If however, anti-androgens are being added as an additional treatment for progressive disease, then this should be recorded on the Additional Treatment Log. Please note SOC hormone therapy refers to LHRHa, anti-androgens or orchidectomy.

If a participant allocated to receive transdermal oestradiol switches to receiving SOC Hormone Therapy i.e. LHRHa, in the **absence of progression**, then this information should be recorded on the SOC Hormone Therapy Log. However, any changes in hormone therapy initiated to treat disease progression should be recorded on the Additional Treatment Log e.g. switching from transdermal oestradiol to LHRH due to progressive disease.

7.2.2 Data Collection For SOC Systemic therapy: docetaxel, abiraterone, enzalutamide, or apalutamide

The decision to use docetaxel, abiraterone, enzalutamide or apalutamide as part of the standard-of-care (SOC) must be made before randomisation and should be recorded on the Randomisation CRF

to ensure the use of SOC agents is balanced between the control and research arms. The date of the starting systemic treatment should be recorded at the time of randomisation; this can be a planned date when randomisation occurs prior to systemic treatment commencing but should be within 12 weeks of starting hormone therapy (see Section 6.1.3). For participants allocated to arms A, K or L all further details should be recorded on the SOC Systemic Treatment CRF and this form sent to the CTU by the 24 week follow-up appointment. If a participant does not receive the planned systemic therapy, this must also be recorded on the SOC Systemic Treatment CRF, together with the reason why. For all participants who have started or planned to start SOC abiraterone, enzalutamide, or apalutamide, details relating to starting date of treatment, dose, and permanent stopping of SOC abiraterone, enzalutamide or apalutamide must be recorded on the SOC Systemic Treatment Log. If a participant does not receive the planned SOC treatment, this must also be recorded on the SOC Systemic Treatment CRF together with the reason why.

7.2.3 Data Collection And Non-Administration Of Standard Radiotherapy

The Radiotherapy Detail CRF should be completed for **all STAMPEDE participants** regardless of being planned for, or subsequently receiving, primary radiotherapy. Where radiotherapy is not reported as planned at randomisation, this form should still be received for confirmation it was not given. For participants where radiotherapy was reported as planned at randomisation but not given, a reason should be provided on the Radiotherapy Detail CRF for example, due to early metastatic progression or participant refusal, whether this is standard-of-care radiotherapy for participants (on any research arm) or research RT to the prostate for Arm H participants.

All radiotherapy and details should be recorded on the Radiotherapy Detail CRF upon completion of the RT schedule. We will now collect acute and late RT side-effects alongside other adverse events on the toxicity form.

7.2.4 Data Collection For Palliative Radiotherapy

Details of any radiotherapy given for progressive disease should be recorded on the Additional Treatment Log and if necessary (e.g. RT for bone pain) as a Skeletal related event (SRE) on the follow-up form.

7.2.5 Data Collection for Research (M1) Radiotherapy

Arm H only: all radiotherapy and acute side-effects details will be recorded on the Radiotherapy Detail and Radiotherapy Acute Toxicity CRFs upon completion of the RT schedule; any RT late side-effects should be recorded on the Follow-up CRF under the section for RTOG Toxicities.

In those cases where RT is not given (for example, due to early metastatic progression or participant refusal), this should be stated on the Radiotherapy Detail CRF together with the reason for non-administration of the treatment.

7.2.6 Data Collection for Additional Treatments Given for Disease Progression

All treatments given for disease progression are recorded on the Additional Treatment Log. Additional treatment should not be given in the absence of disease progression. This log should be updated with all subsequent changes to treatment. Only treatments for progressive disease need to be recorded; details of supportive treatments such as pain killers or bone-strengthening agents e.g. zoledronic acid, given to relieve symptoms, do not need to be provided.

In some scenarios, SOC hormone therapies such as LHRHa or anti-androgens may be given as a treatment for progressive disease. For example, LHRHa may be re-started on relapse for participants with M0 disease who discontinued hormone therapy and commenced surveillance. In addition,

participants allocated to trandermal oestradiol may switch to LHRHa on progression. Historically, some participants progressing on LHRHa will have commenced anti-androgens (dual androgen blockade) as a treatment for progression. In all cases, if treatment is being started for disease progression, treatment data are collected on the Additional Treatment Log and the details of the progression event recorded on the Progression Log.

Please note that any change in ADT which are solely a change in the participant's long-term hormone therapy, and not for disease progression, should be reported on the SOC Hormone Therapy Log only and **not** on the Additional Treatment Log.

7.3 FOLLOW-UP PROCEDURE

Every effort should be made to follow-up all participants who have been randomised up until formal closure of a comparison. Participants should, if possible, remain under the care of an oncologist or urologist for the duration of the trial. If care of a participant is returned to the GP, it is the responsibility of the responsible clinician who obtained the participant's consent to participate in the trial to ensure that all relevant data collection forms are completed. Nurse-led follow-up is permitted and should be conducted in line with local practice and procedures. This can also be performed by a suitably qualified individual who is delegated by the Principal Investigator.

If the participant moves away from the local area, arrangements should be made for trial follow-up to be undertaken by their new local site. Details of other participating site can be obtained from the STAMPEDE Trial Team. Information on participant transfer procedures is detailed in **Section 8.2.** If the responsible clinician moves, appropriate arrangements should be made to arrange for trial follow-up to continue at the site.

All efforts should be made to preserve the initial participant's consent for long-term survival information to be flagged through national registries, for example NHS Digital (previously the Health and Social Care Information Centre); Office of National Statistics (ONS) in England/Wales; General Register Office in Scotland; Hospital Episode Statistics (HES) or Public Health England.

Please see **Section 8** for more information on early stopping of follow-up.

7.3.1 Follow-up Telephone Consultations

In certain circumstances it may be appropriate to replace hospital visits with telephone consultations providing that it is still possible to collect all the necessary follow-up information. In these instances, it is acceptable to replace appointments with telephone consultations providing the required blood results and safety tests are available to the research team. All necessary information required to complete the Follow-up CRF is still required. All details on the telephone consultation must be recorded in the participants' notes as per in person assessments.

7.3.2 Follow-up Using Electronic Healthcare Records

All participants are asked to provide consent to enable the CTU to improve the reliability of long-term follow-up data through linking to other sources of electronic healthcare data. This may include hospital based record systems, NHS digital and national registers, such as the office of national statistics or data held by public health England or other sources which hold relevant information about treatment or outcomes. To ensure study data is updated with accurate data held by others the CTU will collect direct identifiers (participant name and NHS number) and securely store this data for this purpose only, and separately from the trial database. This information will be securely transferred and used to verify the data received by the CTU.

7.4 TRIAL & COMPARISON CLOSURE

For the purpose of complying with UK the clinical regulations (UK Medicines for Human Use Act [Clinical Trials]), each comparison will only be considered 'closed' when active follow-up has ceased. Active follow-up is defined as hospital-based or telephone assessments required to be able to complete the scheduled follow-up assessments. This will be reviewed separately for each comparison after the point of the primary analysis and, if appropriate, later, updated analyses. Longer-term outcome data beyond this time point may be sought through linkage with national registers where possible (and where adequate consent has been obtained) and/or via site research teams. The end of the STAMPEDE Trial is defined as 12 months after the most recent participant, randomised to the last remaining comparison, has completed follow up. Follow-up may include the use of registry data.

7.4.1 Comparisons for which Follow-up has Ceased

In Q3-2018, active follow-up stopped for all trial participants allocated to the research arms within the "original comparisons"; this is defined as all participants allocated to arms B, C, D, E, and F together with all participants allocated to arm A recruited before 15-Nov-2011.

Active follow-up will be stopped to participants in the "M1|RT comparison" (i.e. those recruited to Arm H and the contemporaneously randomised metastatic participants allocated to Arm A), except for those participants who are in the control arm of the "enzalutamide + abiraterone comparison". This follow-up will stop between Q4-2020 and Q1-2021 after sufficient data cleaning has been completed to allow reliable publication of long-term follow-up results. Targeted lists of all participants covered by this change will be disseminated to sites when the date is confirmed.

Active follow-up is defined as hospital-based or telephone assessments required to be able to complete the scheduled follow-up assessments. It should be noted that there may still be some data collection requested from sites to support ongoing sub-studies on closed comparisons. These requests may be for confirmation of health status or data already collected at sites e.g. baseline imaging data and FFPE tumour blocks.

For M1 participants on arm C and contemporaneous arm A, the PSA at baseline will be collected retrospectively where available. Further details regarding this data collection will be disseminated to the respective sites.

Given the above, any longer term analyses of data beyond comparison closure will be performed using observational data collected through national registers and NHS Digital or other datasets, providing such data are accessible.

CRFs, clinical notes and administrative documentation should be kept in a secure location (for example, locked filing cabinets in a room with restricted access). It is permissible to archive this information providing that it can be made accessible and available to the competent or equivalent authorities, the Sponsor, and other delegated authorities with suitable notice as the data may be subject to audit or inspection from any of the above. Information must be held for 25 years after the end of the trial as per **Section 13.1.**

Table 28: Summary of timing of case report forms (CRFs)

Case Report Forms	Timing Of Assessment and CRF
Baseline	
Eligibility Checklist	At Randomisation
Randomisation	At Randomisation
Saliva Pathology	At randomisation or any point on trial. When saliva sample has been taken and sent to Sponsor's designated laboratory.
Treatment	
SOC Hormone Therapy Log	To be completed for participants A-K, when treatment is first started and subsequently every time there is a change in SOC hormone therapy to report (including when Arm L participants switch to SOC HT pre-progression). To be sent in with the corresponding Follow-up CRF.
SOC Systemic Therapy CRF (replaces SOC docetaxel CRF which is no longer in use)	To be completed for all participants randomised to STAMPEDE:<br>To be sent at 20 weeks after randomisation. A form is required for all participants to confirm which treatment received, including if no SOC systemic therapy received<br>Re-send upon completion of SOC abiraterone, enzalutamide or apalutamide treatment
Abiraterone and Enzalutamide Treatment Log (research treatment)	To be completed for participants on arms G or J, when treatment is first started and subsequently every time there is a dose change, treatment pause and re-start.<br>To be sent in with the corresponding Follow-up CRF.
Metformin Treatment	To be completed for participants on arm K, when treatment is first started and subsequently every time there is a dose change, treatment pause and re-start.<br>To be sent in with the corresponding Follow-up CRF.
Transdermal Oestradiol Treatment Log	To be completed for participants on arm L, when treatment is first started and subsequently when reporting change in dose or type of patch.<br>For the transdermal oestradiol arm, the 6-week follow-up form can be completed at the same time as the 4-week visit for the hormone tests (see **Section 6.2.5.B**)
RT Detail	To be completed for all participants randomised to STAMPEDE:<br>• Upon completion of SOC RT<br>• If planned RT is no longer planned (at 10 months after randomisation)<br>• Arm H participants when research RT completed<br>• Arm A participants with newly-diagnosed M1 disease at 3 months to confirm RT was not given
Blood Form	For arm J and contemporaneous A participants only. Taken at progression and end of first line treatment and pre-progression if participant has metastatic disease. Refer to the Sample Collection and Handling Manual for time points.
Metabolic sub study sample CRF	For arm K participants at selected sites recruited on protocol v21.0 that are participating in the metabolic sub study.

CASE REPORT FORMS	TIMING OF ASSESSMENT AND CRF
Assessments	
Follow-up	To be completed at every comparison specific follow-up until comparison closure (See **Table 1** and **Table 2** for comparison specific assessment schedules)
Toxicity	Required at each follow-up until 30 days after permanent stopping of protocol treatment (IMP).
Transdermal Oestradiol Treatment Hormone Results Log	To be completed whenever there are testosterone and oestradiol test results while arm L participants are on transdermal oestradiol.<br><br>For the first cohort of participants starting treatment with a combination of abiraterone, enzalutamide, or apalutamide alongside transdermal oestradiol, please immediately send the log to the CTU following any blood test in the first 3 months of treatment (see **Section 6.2.5.C**).
End of Research Treatment	To be completed when (each) allocated research treatment is permanently stopped or in the event that allocated research treatment is never started (in each case a reason for stopping/never starting should be provided).
Progression Log	To be completed at the first occurrence of each progression event (PSA, local, nodal, distant metastases) and for each method of detection (clinical/symptomatic and objective/radiological).<br>Skeletal-related events confirmed as progression should also be reported here.
Additional Treatment Log	To be completed each time a participant who has progressed starts or completes any additional treatment for progression.
Serious Adverse Event	To be completed following any Serious Adverse Event having confirmed none of the trial specific expedited reporting exemptions are met
Death	At Death
Administration	
Consent form	At Randomisation and when re-consenting following transfer procedure
Participant Transfer Confirmation Form	To be completed when a participant is transferred to a different hospital for the administration of trial treatment and follow-up
Tissue Sample Form	To be completed when sending tumour blocks to Sponsor's designated laboratory.
Co-enrolment	To be completed when a participant is co-enrolled in a post-progression interventional prostate cancer trial. Please see **Section 5.2** for more information.

Table 29: Schedule For Completion Of Treatment Forms For All Comparisons By Arm.

Timing From Randomisation Years	Months	Weeks	Treatment Log[1]
**6-Weekly**			
0	-	6[2]	G, J, K, L
-	-	12	G, J, K, L
-	-	18	G, J, K, L
-	6	24	G, J, K, L
**12-Weekly**			
-	9	36	G, J, K, L
1	12	48	G, J, K, L
-	15	60	G, J, K, L
-	18	72	G, J, K, L
-	21	84	G, J, K, L
-	-	96	G, J, K, L
**6-Monthly**			
2	24	104	G, J, K, L
	30	130	G, J, K, L
3	36	156	G, J, K, L
	42	182	G, J, K, L
4	48	208	G, J, K, L
	54	234	G, J, K, L
5	60	260	G, J, K, L
**Annual**			
6	72	-	G, J, K, L
7	84	-	G, J, K, L
Etc.	-	-	G, J, K, L

Key:

G = SOC + abiraterone
J = SOC + enzalutamide + abiraterone
K = SOC + metformin ± RT ± SOC docetaxel/abiraterone/enzalutamide/apalutamide
L = Transdermal oestradiol ± RT ± SOC docetaxel/abiraterone/enzalutamide/apalutamide

[1] For participants in Arm L on transdermal oestradiol, the hormone tests results are to be reported on the Transdermal Oestradiol Treatment Hormone Results Log

[2] For the transdermal oestradiol arm, the 6-week follow-up form can be completed at the same time as the 4-week visit for the hormone tests (see Section 6.2.5.B)

8 STOPPING OF TREATMENT OR FOLLOW-UP

Participants should be given every encouragement to adhere to their allocated protocol treatment and follow-up schedule, in order to reduce bias. However, a participant has the right to withdraw consent for participation in any aspect of this trial at any time.

8.1 STOPPING RESEARCH INTERVENTIONS

A participant may stop **any STAMPEDE research treatment** for the following reasons:

- Unacceptable toxicity
- Intercurrent illness which prevents further treatment
- Participant refusal
- Any alteration in the participant's condition which justifies the discontinuation of treatment in the clinician's opinion

In all cases, the reason for permanent stopping of research treatment should be recorded on the End of Research Treatment CRF.

In the event of stopping research treatment, unless a participant states otherwise, consent is assumed for continued recording of trial data.

8.1.1 Stopping Research Treatment: Abiraterone, Enzalutamide + Abiraterone

For **participants randomised to Arm G or J**, research treatment should also be discontinued for the following reasons:

- Disease progression whilst on therapy. As detailed in Section 7.1.3, the disease event for stopping treatment may be after the first reportable Failure-Free Survival event. Treatment must be stopped once all three types (biochemical, radiological and clinical) of progression have occurred.
- Intention to commence a new systemic anti-cancer treatment due to evidence of relapse

Trial abiraterone must stop if other systemic treatments are initiated at any time for disease progression control (including chemotherapy, radium-223 etc). Anti-androgens (e.g. bicalutamide) should not be given in combination with abiraterone or enzalutamide due to the risk of toxicity. However, participants may continue on abiraterone or abiraterone and enzalutamide if they receive radiotherapy on a single occasion for a skeletal-related event. Sites must contact the STAMPEDE trial team for further guidance as appropriate.

8.1.2 Stopping Research Treatment: Metformin

For **participants** randomised to Arm K, treatment duration is detailed in Table 9.
Please note that in contrast to other treatments tested in STAMPEDE metformin does **not** need to be stopped following progression. Metformin treatment should aim to **continue post-progression** whilst participants continue to receive ADT.

Reasons for early stopping of metformin include:

- Decline in renal function (metformin must be stopped if GFR ≤30ml/min/1.73m^2, see Section 6.2.4.C)
- Decline in performance status (WHO PS >2)
- Unacceptable toxicity
- Participant refusal

- Intercurrent illness preventing continued metformin treatment
- Investigator decision e.g. administration of IMP within a CTIMP in CRPC setting

If treatment is paused for >3 months or >50% of doses are missed for any reason, it is at the discretion of site investigators as to whether it is appropriate to restart trial treatment with metformin. Contact the CTU for any advice if required.

8.1.3 Stopping Research Treatment: Transdermal Oestradiol

For **participants randomised to Arm L**, treatment with transdermal oestradiol may be discontinued for the following main reasons:

- Unacceptable toxicity
- Participant refusal
- Intercurrent illness
- Investigator decision
- Cardiovascular event (see **Section 7.1.4.A**)

For participants who stop transdermal oestradiol patches due to unacceptable toxicity or intercurrent illness, site investigators can consider changing treatment to LHRHa or allowing a break from hormone therapy. On re-initiation of hormone therapy, the investigator can choose whether it is in the participant's best interests to recommence transdermal oestradiol patches or LHRHa.

If transdermal oestradiol patches are chosen to restart, it is important to recommence with the loading regimen (See **Section 6.2.5.B**) and monitor oestradiol and testosterone levels closely. The process outlined for monitoring hormone levels and titrating doses at randomisation should be used when restarting transdermal oestradiol patches in order to ensure the correct dose is achieved.

In addition, if there is evidence of disease progression, subsequent therapy is at the discretion of the treating clinician with references to any relevant guidelines (see **Table 10**).

8.2 BREAKS IN SOC ADT

The SWOG trial (48) comparing intermittent versus continuous ADT in hormone sensitive metastatic prostate cancer did not find evidence to support that intermittent therapy was non-inferior for overall survival (hazard ratio for death with intermittent therapy 1.10; 90% confidence interval 0.99 to 1.23). Thus, STAMPEDE does not support intermittent androgen therapy as an appropriate upfront treatment approach.

Some participants will experience toxicity or report their QL is adversely impacted by ADT. In these instances we would recommend trying to ameliorate any symptoms with appropriate lifestyle or medical interventions, as per local or national guidelines. Please check that any treatment for symptoms will not interact with the trial treatment.

However, if the participant continues to struggle with ADT, treatment breaks can be considered, although this may impact the trial treatment as well – see below.

Participants who require breaks in SOC ADT due to unacceptable toxicity or an intercurrent illness can restart ADT as long as the investigators deems it is safe to do so, and as long as it remains in the participant's best interests.

All treatment stop and start dates must be recorded, with the reason for the break, on the hormone therapy and trial treatment logs so they can be considered during any data analyses.

8.2.1.A Impact on trial treatment if participant has a break in SOC ADT

Abiraterone:
Participants allocated to receive abiraterone as part of Arm G cannot continue their trial treatment whilst SOC ADT is stopped.

Abiraterone in combination with Enzalutamide, or either treatment alone:
Participants allocated to receive abiraterone with enzalutamide, or remain on either agent as monotherapy, as part of Arm J cannot continue their trial treatment whilst SOC ADT is stopped.

Metformin:
Participants allocated to receive metformin as part of Arm K should continue metformin as per the proposed treatment length at randomisation after stopping SOC ADT. This is as long as it remains in their best interests as assessed by the local clinician. See **Table 9** for specific instructions about the proposed length of metformin treatment for participants who receive radical treatment versus those who do not.

Transdermal Oestradiol:
Transdermal oestradiol patches replace SOC ADT treatment. Please see transdermal oestradiol section 8.1.3 for further details about breaks or stopping trial transdermal oestradiol patch treatment.

8.3 PARTICIPANT TRANSFERS

For participants moving away from the area and planning to transfer care, every effort should be made for the participant to be followed-up at another trial site. The participant will need to sign a new consent form at the new trial site. Once this has been done, the new trial site will take over responsibility for their ongoing participation in the trial.

To document the transfer process the main contact person at both the current and receiving hospitals should complete and sign the Patient Transfer Confirmation form. A fully completed form must be returned to the CTU prior to the participant transfer and any outstanding data queries for the participant should be completed prior to transfer.

On receipt of the completed transfer form, a member of the STAMPEDE team will confirm the database has been updated and request confirmation of the name of the participant's new clinician. Photocopies of the following documents may then be sent to the new hospital to complete the transfer and originals must also be retained at the original site for monitoring purposes:

- Consent form
- Completed CRFs
- Any documentation relating to the participant's participation in STAMPEDE (participant names must be removed from any documentation).

8.4 EARLY CESSATION OF TRIAL PARTICIPATION

If a participant explicitly withdraws consent to have any further trial data recorded, their decision must be respected and the CTU must be informed in writing in the form of a letter, a template is available upon request. All communication surrounding the early cessation of trial participation should be noted in the participant's records. Please note, data for the participant prior to this decision will still be required.

In the majority of cases, participants continue to give permission for their data and information on their health to continue to be collected via clinical notes and national registries. Any information on the follow-up status, however minimal, would be helpful. Investigators are encouraged to facilitate ongoing collection of follow-up data for example, through considering telephone consultations (see **Section 7.3.1**).

Early cessation of trial participation should not be undertaken lightly and the site must consider the implications for the trial and the participant in reaching such a decision. Without long-term data, the efficacy of trial treatments would be less reliable and could lead to inconclusive results. The early stopping of trial treatment should not lead to the early cessation of trial participation and in such cases follow-up assessments should be continued as per trial protocol.

Participants can change their minds about withdrawal at any time and reaffirm their consent to participate in the trial. Follow-up data should be collected only from the point of when consent was re-instated.

9 STATISTICAL CONSIDERATIONS

9.1 METHOD OF RANDOMISATION

Participants will be randomised centrally using a computerised algorithm developed and maintained by CTU. Randomisation will be performed using the method of minimisation over a number of clinically important stratification factors with an additional random element. To decrease determinability, the factors are not listed here but can be found in the Statistical Analysis Plan.

Participants will be randomised between arms as follows:

- All participants who fulfil both comparison-specific eligibility criteria for metformin and transdermal oestradiol will be allocated between A:K:L.
- All participants ineligible for metformin, but eligible for transdermal oestradiol, will be allocated between A:L
- All participants ineligible for transdermal oestradiol, but eligible for metformin, will be allocated between A:K.

See **Appendix H** for the allocation weighting of each arm by previous protocol version; this also shows allocation weighting for research arms previously closed to recruitment.

9.2 OUTCOME MEASURES

The definitive primary outcome measure for each comparison in the trial is overall survival (all-cause mortality), unless otherwise stated. The design of the trial is such that it is important to have additional intermediate primary outcome measures to assess activity in each research arm as the trial progresses.

For comparisons involving research arms B to J the intermediate primary outcome measure is failure-free survival (FFS); this and other outcome measures are listed in **Table 30.** Note that this reflects the original analysis plan for research arm J.

Table 30: Trial Outcome Measures by Comparison Stage (Arms B-J)

COMPARISON STAGE	PRIMARY OUTCOME MEASURE	SECONDARY OUTCOME MEASURES
Pilot phase	Safety*	Feasibility
Activity Stages (AS)	Failure-free survival (FFS)†	Overall survival (OS)<br>Toxicity<br>Symptomatic skeletal events (SSE)
Efficacy Stage (ES)	Overall survival<br>Metastatic progression- free-survival (mPFS) – Arm J M0	Quality-of-life<br>Cost effectiveness<br>Failure-free survival†<br>Toxicity<br>Symptomatic skeletal events (SSE)

*Based on toxicity
†Including biochemical failure (see Section 7.1.3)

For the “enzalutamide + abiraterone comparison” the original plans for the final Efficacy Stage analysis were updated in late 2019, after the earlier analysis stages had been completed. The updated efficacy stage analysis will use metastatic progression-free-survival (mPFS) as the definitive primary outcome measure for participants with baseline M0 disease, and overall survival as the primary outcome measure for participants with baseline M1 disease.

For the “metformin comparison” the intermediate and definitive primary outcome measure are the same, being overall survival; see **Table 33** for full details of all outcome measures for that comparison.

For the “transdermal oestradiol comparison”, overall survival and progression-free survival are the definitive co-primary outcome measures, and the intermediate primary outcome measure is progression-free survival (PFS); see **Table 35**. The rationale for choosing progression-free survival rather than failure-free survival as the outcome measure for this comparison is outlined in **Section 9.7.3**.

The reasons for different emphases in each recruitment stage are explained in **Section 9.3**.

9.3 SAMPLE SIZE: PRINCIPLES

The design is a multi-arm multi-stage, multi-centre, platform, randomised controlled trial. There are a number of stages for each research arm: a Pilot/Feasibility/Safety Phase, Activity Stages and a final Efficacy Stage. Full details of the methodology underlying the trial design are given by Royston et al. (49, 50) The original sample size calculations were performed using the stage2 (version 1.2.0, Mar-2002) and stagen (version 1.1.1, May-2004) programs, both implemented in Stata (Stata Corp, TX) and updated using the later nstage program (version 1.0.3, Jun-2007; version 2.1.0, Jun-2009; version 3.0.1, Sep-2014). (51)

Other than transdermal oestradiol, we have adequately powered each comparison to detect an appropriate improvement in overall survival at the final Efficacy Stage, with high power at each of the planned interim Activity Stages to detect a pre-defined target difference in the intermediate primary outcome. For example, in a cohort with 2 years median FFS and 4 years median overall survival (OS) a target HR of 0.75 for research arm relative to control would translate into an absolute improvement in FFS of 10%, from approximately 50% to 60% at two years, and in OS of 10%, from approximately 50% to 60%, at four years.

The “transdermal oestradiol comparison” is powered only for contributing to a meta-analysis of participants from the STAMPEDE “transdermal oestradiol comparison” and the PATCH trial. It will assess non-inferiority of transdermal oestradiol in terms of overall and progression-free survival which are co-primary outcome measures. For details of the sample size calculations, planned analyses and corresponding operating characteristics, see version 10 of the PATCH protocol.

As each comparison is powered to detect a relative difference in survival, the analyses will be performed when the pre-planned number of events has been reported in the control arm, rather than after a certain number of participants have been recruited to the comparison or a certain amount of time has elapsed. Further details of the sample size calculations and varying assumptions for each research comparison are summarised in the relevant **Sections 9.4-9.8** and detailed in a separate Statistical Design Document which is available on request.

As with all trials, changes in both the standard-of-care and second-line therapies over time are possible which improve outcomes and thus will affect the observed control arm event rates and

associated reporting timelines. In particular, from protocol v8.0, standard-of-care RT was mandated for all participants with N0 M0 disease and no RT contraindication (this is likely to improve outcomes for this subgroup) and standard-of-care docetaxel permitted from protocol v14.0. Further agents are starting to be licensed for participants with castrate-refractory disease which may also improve survival rates. Improved FFS rates would delay the intermediate analyses, for comparisons where FFS is the intermediate primary outcome measure; while improved OS would delay the definitive analyses. Similarly, improved PFS rates could delay both the time of intermediate and definitive analysis for the "transdermal oestradiol comparison". For each comparison event rates are estimated based on data which are publicly available at the time of design. The Statistical Design Document for arms A-K includes models where median survival is varied around such estimated rates.

Figure 4: Schema of progress of STAMPEDE through the trial*

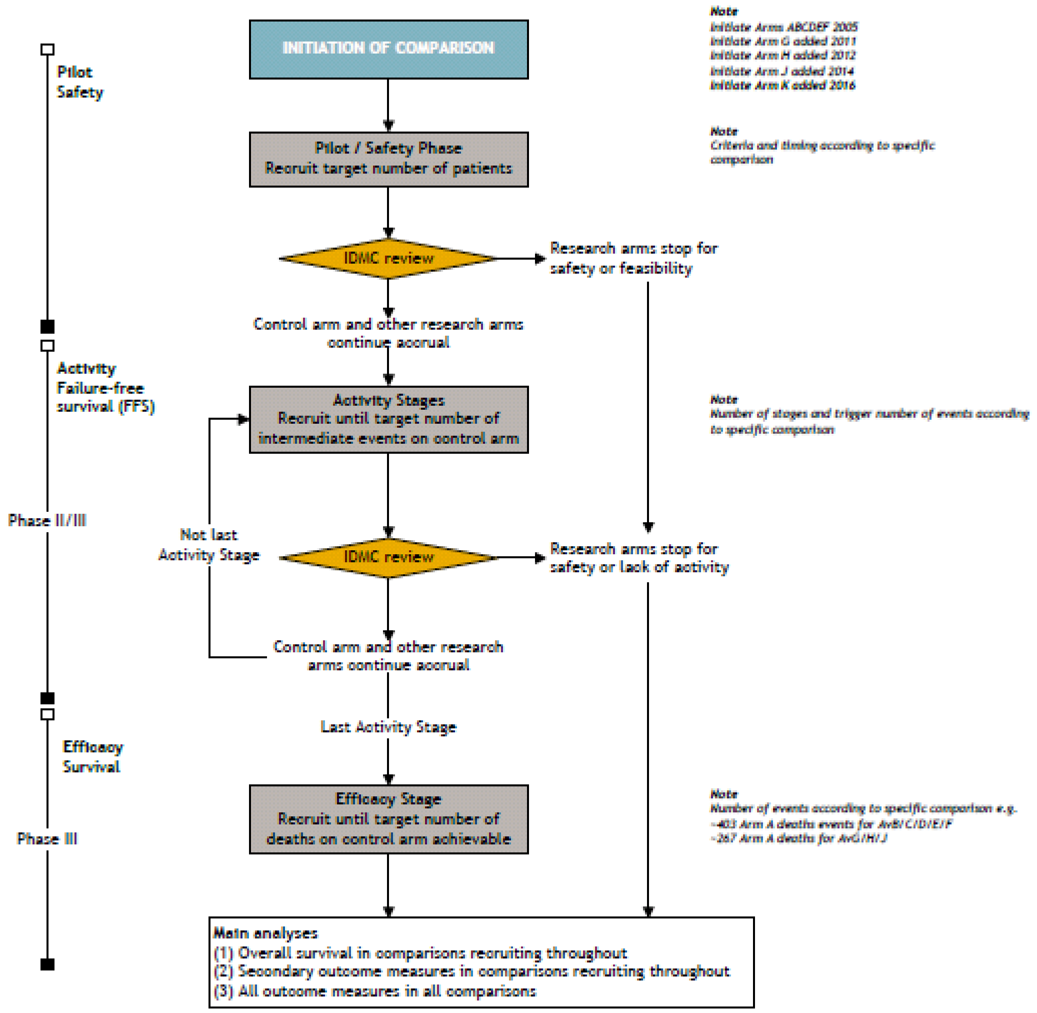

Key
FFS: Failure-free survival
HR: Hazard ratio
IDMC: Independent Data Monitoring Committee
Pts: Patients

Notes
Exact accrual depends on many factors including accrual rate, event rate and arms recruiting in each stage

* Except for the “transdermal oestradiol comparison”

9.4 SAMPLE SIZE ISSUES & TRIAL STAGES: ADDITIONAL RESEARCH ARM H

This is the “M1|RT comparison” and includes participants allocated to research Arm H (SOC+RT) and newly-diagnosed M1 participants with no contraindication to RT allocated to the control Arm A whilst Arm H was open to recruitment. Suitability for allocation to the comparison was assessed before randomisation to ensure comparability with contemporaneous control arm participants.

Table 31: Guidelines for stopping accrual to additional research Arm H

Activity Stage	Sig Level	Power	Targeted HR	Number of control arm events	Consider discontinuation if (HRobserved) is…
I	0.50	95%	0.75	~75	>1.00
II	0.25	95%	0.75	~142	>0.92
III	0.10	95%	0.75	~221	>0.89

9.4.1 Pilot Phase: Additional Research Arm H

The IDMC reviewed safety data, in the context of data from the control arm, when the first 30 participants allocated to Arm H had been on trial for around six months.

9.4.2 Activity Stages I-III: Additional Research Arm H

The same principles were applied to this new comparison as to previous comparisons and an equal allocation ratio of control arm participants to participants allocated to Arm H was employed, as for Arm G. The number of control arm events required to trigger the intermediate analyses are the same as for the “abiraterone comparison” (see Table 31).

9.4.3 Efficacy Stage IV: Additional Research Arm H

The analysis of Efficacy Stage IV for this comparison was planned for when ~267 deaths had been observed in the relevant control arm participants. This was to give 90% power to detect the targeted hazard ratio of 0.75 at one-sided significance level of 0.025.

9.4.4 Sample Size For Additional Research Arm H

Consideration was given to ceasing further randomisations to Arm H if it was not showing sufficient evidence of activity on the intermediate primary outcome measure (FFS), as for the other research arms. This research comparison is relevant to around 60% of participants joining STAMPEDE. At the point of the scientific approval, accrual was averaging around 80 participants per month to the trial; therefore, up to approximately 48 participants a month would be eligible for the comparison. If accrual to the trial was slower at 70 participants per month, then accrual to this comparison could be between 18 and 42 participants per month, depending on which other trial arms are open to recruitment at the time.

We were targeting a 25% relative improvement in overall survival following local radiotherapy to the prostate in this participant group. This is the same size of effect targeted with the other research arms in STAMPEDE. This relative improvement can be further justified in the light of MRC PR07 which demonstrated an improvement of this magnitude for adding radiotherapy to ADT in locally-advanced disease, with a hazard ratio for overall survival of 0.77 (95% CI 0.61 to 0.98). In that trial, fewer than half of the deaths were from prostate cancer, whereas in newly-diagnosed metastatic participants nearly all people will die of their disease. Therefore, it is relevant to note the relative benefit of radiotherapy in PR07 in terms of prostate cancer-specific survival, where the hazard ratio was 0.46 (95% CI 0.34 to 0.61) after a median follow-up time of 8 years (52).

We anticipated that around 1250 participants were required over 4 years to observe 267 control arm deaths after 5.25 years. This assumed that (i) recruitment was constantly 70 pts/m to the trial overall; (ii) the original research arms stopped accrual within 6 months after activation of the RT arm; (iii) the abiraterone arm stopped accrual around 24 months after activation of the RT arm; and (iv) a further new research arm with an equal allocation ratio was introduced 18 months after activation of the RT arm. In Protocol version 13.0, we reflected on these four points: (i) recruitment to the trial has been faster; (ii) the original research arms completed accrual 2 months after activation of the RT arm; (iii) the abiraterone arm stopped accrual 12 months after activation of the RT arm; and (iv) Arm J was activated 18 months after activation of the RT arm, Arm H.

Of participants joining STAMPEDE during this time, 60% have been eligible for the "M1|RT comparison". Prior to randomisation, a RT schedule had to be nominated: Weekly or Daily. We have observed that around half of participants in the comparison are nominated for RT with the Daily schedule and half for the Weekly schedule, primarily chosen by trial site with participant groups nominated for each schedule observed to be comparable at baseline. There will likely be interest to know the effect of each RT schedule when the main results are reported. This will be explored by "within schedule" comparisons of participants randomised to research vs control (arms H vs A) within each nominated RT schedule.

To ensure adequate power for these "within schedule" analyses, in Protocol version 13.0, the target sample size was increased from 1,250 participants up to around 1,800 participants, resulting in an approximate increase in the split by planned RT schedule from 625 to 900 in each "within schedule" analysis. A FFS analysis "within schedule" was carried out at the time of the "main analysis"; this was predicted to have ~300 control arm FFS events by schedule (FFS "within schedule" analysis parameters: target HR=0.75, power 90%, 1-sided α=0.015). For either of the RT schedules showing evidence of an effect on FFS, a comparative "within schedule" analysis was planned to be carried out on survival when ~199 control arm deaths are observed in that schedule comparison. This is a closed test with OS only formally compared within schedule if there is an advantage in FFS for that RT schedule at the main analysis. Thus, extending recruitment enables a secondary analysis of the impact of RT on survival by planned "RT schedule" to happen within around 18 months from the first main analysis.

All sample scenarios are documented in the Trial Master File.

All participants joining the trial will be starting long-term ADT for the first time. The focus of this comparison is on the newly-diagnosed, metastatic participants (with no contraindications to RT), which is the largest subgroup of participants in the trial and the group of participants at highest risk of death from prostate cancer. Participants with non-metastatic disease will be excluded from this particular comparison as there are already randomised data demonstrating the survival benefit from radiotherapy in participants with locally-advanced disease. Radiotherapy is now mandatory in node negative participants; it is also recommended in the node-positive, non-metastatic (N+ M0) group. Relapsing participants are also excluded from this comparison.

For the control arm of the whole trial, we constructed sample size scenarios based on median failure-free survival being 18, 24 or 30 months; the event rate would depend on the participant mix. We now know that around 60% of participants have M1 disease at trial entry and we have reported that FFS at 24 months is 51% across the whole of the control arm participant sample.(53)

For the updated sample size calculation for the "M1|RT comparison", we based our estimates on the subgroup of participants with newly-diagnosed M1 disease in the control arm. Therefore, we

estimated median FFS for control arm participants in this comparison to be 1 year and estimated that median overall survival would be around 3.5 years.

9.5 SAMPLE SIZE ISSUES & TRIAL STAGES: ADDITIONAL RESEARCH ARM J

This is the “enzalutamide + abiraterone comparison” and includes participants allocated to research Arm J (SOC + enzalutamide + abiraterone) and participants contemporaneously allocated to the control Arm A.

Note that as of Protocol version 20.0 the details of the Efficacy Stage analysis for the “enzalutamide + abiraterone comparison” have changed to reflect separate analyses for non-metastatic (M0) and metastatic (M1) participants and a broadening of the therapeutic intervention being tested. See below for further information.

9.5.1 Pilot Phase: Additional Research Arm J

The IDMC first reviewed safety data for this combination when the first 50 participants allocated to Arm J had been on trial around 6 weeks (i.e. to the first follow-up visit).

Furthermore, an additional review of safety was performed when these 50 Arm J participants had been on trial for around 6 months. Safety is routinely reviewed at regular intervals and additional safety reviews will be performed if the IDMC raises any concerns.

Direct comparison will be available with contemporaneously randomised participants on Arm A (SOC alone). Contextual data will be provided from Arm G (SOC plus abiraterone). Indicative safety data may also be available on the combination from other studies in CRPC.

9.5.2 Activity Stages I-II: Additional Research Arm J

The principles of intermediate analyses were applied to this new comparison as to previous comparisons, but some of the details were different, and an equal allocation ratio of control arm participants to participants allocated to Arm J was employed; as for Arms G and H. Owing to the expected accrual rate to the trial (>100 pts/m), the expected slower event rate in all participants given improvements to SOC and specifically in participants randomised to this comparison. Given the simultaneous recruitment of M1 (but not M0) participants to the “M1|RT comparison”, only two activity stages were planned before accrual completed. These are set out in **Table 32**.

The IDMC intermediate activity stage reviews were completed in Nov-2015 and Mar-2016 for Arm J and recommended continuation of the comparison.

Table 32: Guidelines for stopping accrual to the additional research Arm J

Activity Stage	Sig Level	Power	Targeted HR	Number of control arm events	Consider discontinuation if (HRobserved) is…
I	0.40	95%	0.70	~66	>0.957
II	0.12	95%	0.70	~139	>0.869

9.5.3 Efficacy Stage III: Additional Research Arm J

The analysis of the final Efficacy Stage for this comparison was originally planned to be performed when around 267 deaths had been observed in the control arm. This would give 90% power to detect the targeted hazard ratio of 0.75 at a one-sided significance level of 0.025.

In late 2019 the plans for the Efficacy Stage III analysis of the "enzalutamide + abiraterone comparison" were updated. See section 9.5.5 for further details.

9.5.4 Sample Size For Additional Research Arm J

Consideration was given to ceasing further randomisations to Arm J if it was not showing sufficient evidence of activity on the intermediate primary outcome measure (FFS), just as for the other research arms.

The participant mix for this comparison is likely to represent a more favourable prognosis on average than in the original comparisons, due to concurrent recruitment of M1 but not M0 participants, to Arm H.

We anticipated that around 1,800 participants were required within 3.5 years to observe ~267 control arm deaths within 6 years. The default scenario assumes that (i) recruitment is constantly 70pts/m to the trial overall, (ii) Arm H (M1|RT) accrues throughout and (iii) a further new research arm with an equal allocation ratio is introduced 18 months after activation of Arm J. The stopping date for Arm G is no longer an assumption.

Variations on these factors are documented in a Statistical Design Document. If accrual rates to the trial were at 150pts/m (as observed during summer 2013), accrual of around 1,800 participants to the comparison could be achieved within 2 years. These sample scenarios are documented in the Trial Master File.

Updating the standard-of-care to include docetaxel has minimal impact on the projected time to maturity of the "enzalutamide + abiraterone comparison".

9.5.5 Update to Efficacy Stage Analysis For Additional Research Arm J

In Protocol version 20.0 the planned Efficacy Stage analysis of the "enzalutamide + abiraterone comparison" was updated. This followed the larger-than-expected improvement in overall survival seen in the primary analysis of the "abiraterone comparison"; evidence from other trials combining enzalutamide with abiraterone did not result in further efficacy gains; and continued divergence in the aims of first-line therapy for patients with baseline metastatic and non-metastatic disease since the comparison was conceived.

In late 2019, the STAMPEDE TMG and TSC approved an updated analysis plan designed to test the impact of the addition of the more broadly-defined therapeutic intervention of androgen receptor (AR)-targeted therapy on patient outcomes, compared to SOC treatment alone. Patients from the "abiraterone comparison" (A vs G) and the "enzalutamide + abiraterone comparison" (A vs J) will be included in a combined analysis comparing the addition of AR-targeted therapy (abiraterone or the combination of enzalutamide and abiraterone) to SOC with SOC alone. Those with baseline M0 disease at entry to the study will be analysed separately to those with baseline M1 disease.

<u>Non-metastatic (M0) patients</u>
The Efficacy Stage analysis for these patients will test whether adjuvant AR-targeted therapy improves survival compared to SOC alone, using the primary outcome of metastatic progression-free-survival (mPFS).

A total of 1,982 patients with M0 disease were randomised to arms G or J or contemporaneously to the control arm. We plan to perform the Efficacy Stage analysis when a total of 315 mPFS events have been observed in the control arm patients for this sub-group. This will provide 90% power to

confirm a treatment effect equivalent to a HR of 0.75 for AR-targeted therapy, at the 1.25% one-sided significance level, based on an assumption of 70% survival in the control arm at 66 months.

<u>Metastatic (M1) patients</u>
The Efficacy Stage analysis for these patients will validate whether a new biomarker that includes lobular vs basal transcriptomic sub-classification (PAM50) is predictive of differential response to adjuvant AR-targeted therapy. Prior to the data freeze for the analysis, as many M1 patients as possible will be classified into one of two subgroups, biomarker positive (luminal B) and biomarker negative (basal), based on the results of the biomarker assay. The primary outcome measure will be overall survival.

A total of 1,916 patients with M1 disease were randomised to arms G or J or contemporaneously to the control arm. The timing and power for the planned analysis will depend on the proportion of these patients who are successfully classified using the biomarker assay. For example, if 50% of patients are classified, we expect to have 80% power to confirm an interaction between allocation to adjuvant AR-targeted therapy and biomarker classification at the two-sided 5% significance level if the analysis is performed in the spring of 2022, based on a minimum follow-up duration of 70 months. This assumes that 40% of patients will be classified as ‘positive’ and the remaining 60% as ‘negative’, with an anticipated treatment effect equivalent to a HR of 0.85 for AR-targeted therapy in the ‘positive’ group and a treatment effect HR of 0.45 for patients in the ‘negative’ biomarker group, equivalent to a hazard ratio ratio (HRR) of 1.9.

Further details of the calculations and assumptions underpinning the Efficacy Stage analysis planned for both subgroups can be found in the SAP for the “enzaluatmide + abiraterone comparison”.

9.6 SAMPLE SIZE ISSUES & TRIAL STAGES: ADDITIONAL RESEARCH ARM K

This is the “metformin comparison” and includes participants allocated to research Arm K (SOC + metformin) and the equivalent non-diabetic participants with no contraindication to metformin contemporaneously allocated to the control Arm A whilst Arm K is open to recruitment. Suitability for allocation to the comparison is assessed before randomisation to ensure comparability with contemporaneous control arm participants

9.6.1 Implementation: Additional Research Arm K

The implementation of the MAMS principles are different in this comparison for the following reasons:

- Although all non-diabetic participants will be eligible for allocation to the “metformin comparison”, the timing of the analyses will be driven only by the M1 participants. (See Section 9.6.4 for discussion of the implications for power overall and in M0/M1 subgroup analyses.)

- Failure-free survival will not be used as the intermediate primary outcome measure; overall survival will be used as both the intermediate and definitive primary outcome measure. This is because we are not convinced that any comment on metformin’s usefulness should be determined from an ability to act on a PSA-driven outcome measure. Furthermore, treatment with metformin is intended to continue throughout long-term hormone therapy which may include going well beyond an FFS event, particularly in M1 participants.

- The target HR is 0.80 for overall survival (a 20% relative improvement). This is a smaller relative improvement in survival than targeted for previous comparisons because of metformin’s known low toxicity profile, the low cost of the drug and the potential positive

effects on metabolic parameters and morbidity; a smaller impact on overall survival may still have clinical benefit.

9.6.2 Outcome Measures: Additional Research Arm K

Table 33 lists the outcome measures for this comparison and can be compared with the outcome measures for the other comparisons in Table 30.

Table 33: Trial outcome measures by stage for the "metformin comparison"

COMPARISON STAGE	PRIMARY OUTCOME MEASURES	SECONDARY OUTCOME MEASURES
Pilot phase	Safety*	Feasibility<br>Metabolic effects§<br>Cardiovascular event: major adverse cardiac events‡
Activity Stage (AS) I	Overall survival	Failure-free survival† (FFS)<br>Symptomatic skeletal events (SSE)<br>Toxicity<br>Metabolic effects §<br>Cardiovascular event: major adverse cardiac events‡
Efficacy Stage (ES) II	Overall survival	Metastatic progression-free survival (M0 participants)<br>Progression free survival (M1 participants)<br>Toxicity<br>Symptomatic skeletal events (SSE)<br>Failure-free survival† (FFS)<br>Metabolic effects §<br>Quality-of-life<br>Cost effectiveness<br>Correlative outcomes ₳<br>Cardiovascular event: major adverse cardiac events‡

*Based on toxicity
§Including changes in: BMI; Haemoglobin A1c (HbA1c); waist circumference and a new diagnosis of diabetes mellitus
‡MACE; nonfatal MI, nonfatal stroke, & death from CVS causes
†Including biochemical failure (see Section 6.1.2 and Appendix J)
₳Plasma lipid and fasting triglyceride levels, fasting plasma glucose
Sarcopenia and/or radiological progression free survival (rPFS)
Plasma insulin
AMP Kinase

Note: All arms are unblinded so primary outcome measures for this comparison are objectively measured with caution to be taken around interpretation of more subjective secondary outcome measures such as symptomatic skeletal events

9.6.3 Pilot Phase: Additional Research Arm K

The IDMC reviewed safety data for this comparison when the first 50 participants allocated to Arm K had been on trial around 12 months. Furthermore, analyses were conducted on metabolic parameters (see Table 33). If there was harm observed in metabolic effects, or any serious concerns regarding the toxicity profile, recruitment would be stopped; there were no formal criteria to guide this.

Safety is routinely reviewed at regular intervals and additional safety reviews will be performed if the IDMC raises any concerns.

9.6.4 Activity Stage I: Additional Research Arm K

The principles of intermediate analyses will be applied to this new comparison as to previous comparisons, but some of the details will be different, and an equal allocation ratio of control arm participants to participants allocated to Arm K is employed; as for Arms G, H and J. Owing to the expected accrual rate to the trial overall (>100 pts/m) and the interim primary outcome being overall survival, only one intermediate activity stage is planned before accrual is completed; this is set out in Table 34.

Although analyses are triggered by events in M1 participants, they will include all participants in the "metformin comparison"; this will have high power. A separate subgroup analysis in M1 participants (conventionally-powered) and M0 participants (limited power) will then look at consistency of effect; few deaths in M0 participants are expected at this time. The IDMC recommendation will be based on the totality of the available data, including safety, metabolic and compliance data.

The IDMC reviewed the intermediate activity stage data for Arm K in May 2020 and recommended continuation of the comparison.

Table 34: Guidelines for stopping accrual to the additional research Arm K

Activity Stage	Sig Level	Power	Targeted HR	Number of control arm events	Consider discontinuation if HR_K (observed) is…
I	0.40	92%	0.80	~121 M1 deaths	>0.965

9.6.5 Efficacy Stage II: Additional Research Arm K

The analysis of the final Efficacy Stage for this comparison will be performed when around 473 deaths have been observed for M1 participants randomised contemporaneously to the control arm. This would give 92% power to detect the targeted hazard ratio of 0.80 at a one-sided significance level of 0.025 at the final Efficacy Stage, and 86% pairwise power overall.

As with the intermediate activity, this analysis will include all participants in the comparison, with a separate subgroup analysis in M1 and M0 participants looking at consistency of effect. At this time point we predict approximately 100 control arm M0 deaths will be observed. Further subgroup analyses, defined by the stratification factors, are planned to check for consistency of effect at intermediate and final analyses. Due to this comparison being powered for overall survival in M1 patients, the relatively high OS for M0 patients means that anaysis of OS in this subgroup will not have high power. As such, an additional outcome measure of metastasis-free survival will be analysed as part of the subgroup analysis.

9.6.6 Sample Size For Additional Research Arm K

Consideration would be given to ceasing further randomisations to Arm K if it did not show sufficient evidence of improvement on overall survival at the intermediate analysis.

We anticipate that around 2,800 participants, including around 1,700 M1 participants, are required over 3 years to observe ~473 control arm M1 deaths over around 7 years. (This is a revision from the initial target – see Section 9.6.7). This number and time will be dependent on the observed overall survival. The default scenario assumes (i) recruitment is constantly 100pts/m to the trial overall, (ii) co-recruitment throughout of the equivalent of one other research arm, and (iii) the majority of metastatic participants will also have docetaxel but non-metastatic participants will not. Variations on these factors are documented in a Statistical Design Document. Sample scenarios are documented in the Trial Master File.

Updating the standard-of-care to permit first-line use of docetaxel was assumed within the sample size scenarios and is reflected in the projected time to maturity of the “metformin comparison”.

9.6.7 Further Sample Size Issues For Additional Research Arm K

Analyses for the “metformin comparison” will be timed from randomisation. The point of randomisation compared to the start of hormone therapy may differ, depending on the planned use of docetaxel. This practical information will be reviewed by the TMG and IDMC.

For the development of Protocol v19, the sample size calculations for the “metformin comparison” were discussed by the TMG and revised to the estimates as presented in Section 9.6.4 – Section 9.6.6.

The original sample size estimates for this comparison were based on a lower target for power than the previously-added comparisons, with 90% power for the interim analysis, 85% power for the final analysis and 80% pairwise power overall. The observed accrual to the “metformin comparison” is higher than forecast. Therefore the TMG took the opportunity to revisit the sample size target for the “metformin comparison”.

The revised sample size estimates aim for a higher target power of 92% at both interim and final analysis with 86% pairwise power overall, increasing the analysis power for this comparison in line with that of previous STAMPEDE comparisons. These revisions have resulted in the overall sample size for the comparison increasing from 1800 patients in Protocol v18 to 2800 patients in Protocol v19. This was determined to be achievable within the forecast timelines for recruitment i.e. by the end of 2019, and has the benefit of bringing forward the reporting timelines by approximately one year.

9.7 SAMPLE SIZE ISSUES & TRIAL STAGES: ADDITIONAL RESEARCH ARM L

This is the "transdermal oestradiol comparison" and includes participants allocated to research Arm L (transdermal oestradiol ± RT ± docetaxel) and the equivalent, eligible participants contemporaneously allocated to the control Arm A (SOC).

The phase III evaluation of the clinical efficacy of transdermal oestradiol will ultimately be based on the relevant data from this comparison within STAMPEDE and the PATCH trial, combined using an individual participant data meta-analysis. The overall evaluation is based on a non-inferiority design.

9.7.1 Implementation And Outcome Measures: Additional Research Arm L

The transdermal oestradiol evaluation is based on the following approach.

9.7.1.A Earlier Stages In The PATCH Trial

- The early stages of the PATCH trial already demonstrated the safety and early activity of transdermal oestradiol in comparison to LHRH therapy (see **Appendix I**) (31). The pilot phase (completed in 2010, n=254) showed the rates of cardiovascular events in the transdermal oestradiol and LHRH arms were similar, and the castration rates were equivalent. These results were confirmed by longer-term data including nearly 900 patients enrolled up to Oct-2015.

- A pre-planned, confidential interim analysis undertaken in Jun-2013, based on progression-free survival, at the end of the Phase II component of the PATCH trial, led the PATCH IDMC to recommend further recruitment for an extension to Phase III. That analysis included 638 patients with 206 PFS events, and reviewed data against a pre-specified non-inferiority margin hazard ratio of 1.25 with a 1-sided alpha 0.25.

9.7.1.B STAMPEDE And PATCH Meta-analysis

- To assess the clinical efficacy of transdermal oestradiol, the relevant data from the STAMPEDE "transdermal oestradiol comparison" will be combined with that data from all patients recruited into PATCH; the data from STAMPEDE will not be analysed alone.

- As the eligibility criteria with respect to the timing of start of ADT differs between the STAMPEDE "transdermal oestradiol comparison" and the PATCH trial (see **Section 4.3.1**), the "transdermal oestradiol comparison" will undergo an initial Pilot Phase to assess castration rates and safety among those participants on Arm L. This will also include a safety review of participants receiving transdermal oestradiol in combination with docetaxel. The data will be reviewed by the PATCH IDMC when there are 30 participants in Arm L who have been followed up for at least 18 weeks. A feasibility review will also be performed at the same time.

- The pre-planned Activity Stage II, on intermediate primary outcome measure progression-free survival, will take place based on combined data from the STAMPEDE "transdermal oestradiol comparison" participants and PATCH patients.

- The same approach will be used at the final Efficacy Stage, with progression-free and overall survival as definitive co-primary outcome measures (see PATCH Protocol v13.0for further details). The rationale for choosing progression-free survival as both the intermediate primary outcome measure and as part of the definitive co-primary outcome measure for the "transdermal oestradiol comparison" is outlined in **Section 9.7.3**

Table 35 summarises the outcome measures for each stage of this research comparison. The target sample size for the meta-analysis of the "transdermal oestradiol comparison" is approximately 2,500 participants, with around 700 to be recruited through the STAMPEDE "transdermal oestradiol comparison". By Feb-2017, around 1,200 patients had been recruited directly to the PATCH trial.

Table 35: Trial outcome measures by stage for the "transdermal oestradiol comparison"

COMPARISON STAGE	DATA SOURCE(S)	PRIMARY OUTCOME MEASURES	SECONDARY OUTCOME MEASURES
Pilot phase (completed 2010)	PATCH trial	Cardiovascular morbidity and mortality	Castration rates<br>Other toxicities<br>Metabolic effects
Activity Stage I (completed 2013)	PATCH trial	Progression-Free Survival*	Cardiovascular and other toxicities<br>Castration rates<br>Metabolic effects
Activity Stage II+$	PATCH and STAMPEDE trials	Progression-Free Survival*	Cardiovascular & other toxicities
Efficacy Stage III$	PATCH and STAMPEDE trials	Progression-Free Survival*<br>Overall survival	Cardiovascular & other toxicities<br>Prostate cancer specific survival<br>Quality-of-life

* Defined as the earliest among biochemical failure, clinical progression (local progression, lymph node progression, distant metastases), or death from any cause (see Section 9.7.3).

+ In addition, there is Pilot Phase to assess castration rates and safety among Arm L participants within STAMPEDE, since the eligibility criteria with respect to timing of start of ADT differs between the transdermal oestradiol comparison within STAMPEDE and the PATCH trial (see Section 4.3.1).

$ The timing of these analyses is determined by when a pre-specified number of events for the primary outcome measure have been observed in the control arms for the PATCH and STAMPEDE trials combined. Please see the PATCH Protocol v10.0 for further details.

9.7.2 Additional Use of Outcome Data from the "transdermal oestradiol comparison"

Participants allocated to the "transdermal oestradiol comparison" may provide additional consent to participate in translational sub-studies, see Section 4.7 for details. Subsequent correlative analysis using outcome data from these participants will be undertaken by the STAMPEDE team and collaborators, overseen by the STAMPEDE BRG and other STAMPEDE oversight committees.

9.7.3 Definition of PFS and Use As Co-primary Outcome Measure: Additional Research Arm L

Note that the definition of progression-free survival (PFS) used within the "transdermal oestradiol comparison" analyses differs slightly to that of failure-free survival used for other research comparisons within STAMPEDE. This is because it includes death from any cause as an event- i.e. both PCa deaths and non-PCa deaths (see Appendix C for further details of the definition of progression). Progression-free survival is hence defined as time from randomisation to the first of: biochemical failure, clinical progression or death from any cause.

The use of PFS rather than FFS for the “transdermal oestradiol comparison” has no practical impact on STAMPEDE. The rationale for choosing PFS as part of the co-primary outcome measure for the “transdermal oestradiol comparison” is to capture any potential effects on survival due to the different toxicity profiles between transdermal oestradiol and LHRH.

Although PFS and survival are co-primary endpoints, their respective primary analyses will be triggered at different timepoints particularly because PFS is likely to contain a relatively low proportion of deaths as the contributing first PFS event.

9.7.4 Abiraterone/Enzalutamide/Apalutamide enhanced safety monitoring

Most patients with metastatic disease entering the trial prior to the COVID-19 pandemic received docetaxel as part of their standard treatment. However, following the COVID-19 pandemic, clinicians have the option to offer alternative therapies including enzalutamide, abiraterone, or apalutamide. There is no experience of combining these agents with transdermal oestradiol patches (though no significant interaction is anticipated) an enhanced safety monitoring study will be conducted. This will involve close monitoring of all patients who receive these agents in combination with transdermal oestrogen including testosterone, oestradiol, PSA levels and any events reported through SAE forms in real time.

In addition, the IDMC will formally review the enhanced safety monitoring data utilising a Simon Two Stage design, based on the castration rates at twelve weeks among patients still undergoing treatment with patches, with a significance level of 5% and power of 80%. The enhanced safety monitoring study will test a null response of 78% (P0) against an alternative response of 93% (P1), the level observed in patients receiving patches alone. The optimal design requires at least 8/10 patients to be castrate at stage I, and 37/43 patients to be castrate at stage II. Alongside efficacy data, toxicity data will undergo clinical review, and both aspects will be considered before deciding whether further patients should be treated with the combination. Any concerns raised by any aspect of the data will be discussed between the IDMC and appropriate TMG members, with any recommendations discussed with the TSC.

Clinicians have the option to treat patients with any of enzalutamide, abiraterone, or apalutamide. Initial analyses will consider patients who receive any of these treatments. If sufficient patients receive any particular one of these treatments, secondary analyses will look within each treatment.

9.8 FURTHER NOTES ON TRIAL DESIGN

9.8.1 Overall Sample Size

Given the adaptive nature of the study, there is no formal overall sample size target, but the numbers of participants required for each comparison are detailed in **Sections 9.4-9.7**. To date, more than 11,000 participants have been recruited overall.

9.8.2 Factorial Design

We note here that we did not employ a factorial design in the original design of this trial because we anticipated the possibility of synergy between SOC, zoledronic acid and docetaxel and between SOC, zoledronic acid and celecoxib.

It would not be possible to assess any such interactions reliably in a factorial trial (see the Statistical Design Document for further details).

9.9 INTERIM MONITORING AND ANALYSES

The accumulating data will be reviewed at regular intervals (approximately annually) by an Independent Data Monitoring Committee (IDMC), including pre-specified formal intermediate analyses of activity data (see also **Section 16**). These analyses will be performed by the trial team at CTU. Only participants randomised contemporaneously, and eligible for that comparison, will be included in the comparison of each research arm against control e.g. participants allocated to the control arm prior to Protocol version 15.0 will not contribute to the "metformin comparison" (Arm A vs Arm K). For the “transdermal oestradiol comparison”, the relevant STAMPEDE data will only be analysed as a meta-analysis in combination with the PATCH trial. Therefore, interim data from this comparison will be reviewed by the PATCH IDMC.

The IDMC will be asked to give advice on whether the accumulating data from the trial justifies continuing recruitment of further participants or further follow-up; guidelines for discontinuation of accrual for the relevant Activity Stages, together with results from any other relevant trials will aid them in this. A decision to discontinue recruitment, either in all participants or in selected subgroups, will be made only if the result is likely to convince a broad range of clinicians including those entering participants into the trial and the general clinical community. The intermediate stopping guidelines apply to the intermediate primary outcome measure.

To stop accrual early for benefit in any comparison would require convincing data in terms of the definitive primary outcome measure, overall survival. For example, this could be one-sided $p<0.0005$ as proposed by Haybittle-Peto.(54, 55) The use of such a guideline for stopping for benefit has a minimal impact on the operating characteristics.

If a decision is made to continue without change, the IDMC will advise on the frequency of future reviews of the data on the basis of accrual and event rates. The IDMC will make recommendations to the Trial Steering Committee (TSC, see **Section 16**) as to whether the trial should continue in its present form. While the trial is ongoing the accumulating data will generally remain confidential, unless the TSC and IDMC agree that the data should be made public.

9.10 OUTLINE ANALYSIS PLAN

Analyses will be performed on an intention-to-treat basis.

For comparisons involving arms A-K, the standard unadjusted log-rank approach will be applied to analyses of intermediate and definitive primary outcome measures. The impact of potential confounders including the stratification factors used at randomisation will be considered in a Cox proportional hazards model.

Flexible parametric models will be used to calculate the absolute differences between the arms to show treatment differences over time and to estimate restricted mean “survival” times (RMST). The estimated difference in RMST will be used preferentially to compare treatment arms if the proportional hazards assumptions required for hazard ratios cannot be supported.

In the “transdermal oestradiol comparison,” a meta-analysis approach will be used to combine data from the STAMPEDE and PATCH trials. The analysis will also take into account the change in randomisation ratio partway through the PATCH trial (from 2:1 for transdermal oestradiol versus LHRH before Feb-2011, to 1:1 thereafter). In addition, as the comparison uses a non-inferiority design, sensitivity analyses will be conducted based on a number of pre-defined descriptions for the per-protocol population.

9.10.1 Pilot / Safety Phases

Feasibility of the trial originally, and now of individual research comparisons, was and still is considered in terms of acceptability of the trial randomisation, reported toxicities and adherence to trial medication. Sites participating in the Pilot Phase for the original research arms were required to keep an anonymised log of all participants assessed for trial eligibility (see protocol v2.0) so that the number of participants who did not participate in the study and the number of eligible participants who chose to not participate in the study could be summarised (reasons for non-participation were collected where the participant was willing). The anonymised logs are no longer needed for new research arms (since protocol v8.0).

For each research comparison we shall describe the incidence of expected and unexpected severe toxicities and adverse events/reactions (see **Section 11.1.1**) amongst the participants who are randomised to the comparison to decide whether to continue beyond this Pilot/safety Phase.

9.10.2 Activity And Efficacy Stages

The approach to analysis of these stages is summarised within the sample size calculations (see earlier subsections of **Section 9**). Each research arm will be compared in a pairwise fashion against the contemporaneously recruited control arm, except for the planned Efficacy Stage analyses for the "enzalutamide + abiraterone comparison", in which patients allocated to AR-targeted therapy (arms G and J) will be compared against contemporaneously recruited control arm patients.

Full details are available in the relevant Statistical Analysis Plan. See **Figure 4** for an overview of the schema of progress.

10 MONITORING AND QUALITY ASSURANCE

10.1 DATA MONITORING

To ensure patient safety and data integrity is maintained to a high standard, remote and on-site monitoring will be conducted throughout the lifetime of the study.

10.1.1 Central Monitoring Of Consent

Anonymised copies of the participant's initial consent form (including the additional research consent) should be sent to the STAMPEDE team at the CTU, as soon as randomisation has been completed. Once the consent has been received and reviewed by the CTU the participants "treatment and follow up schedule" can be released to sites.

Any subsequent re-consent forms should be sent as soon as possible to enable central monitoring and recording of consent. The dates and signatures should be visible on the copies sent to the CTU; however the name of the participant must be omitted. Any queries resulting after central monitoring will be redirected to sites for clarification. The original non-anonymised consent forms should be kept at site in the Investigator Site File.

10.1.2 Central Monitoring Of Data

Data provided to the CTU will be checked for data errors, inconsistent and missing data. The STAMPEDE team will issue data clarification requests, query reports or address issues identified via email with site staff. Data Quality and site performance sites will be reviewed, issues identified when appropriate will be fed back to sites. Sites may be identified for training or onsite monitoring through central monitoring checks.

10.1.3 Direct Access to Patient Data

Participating investigators should agree to allow trial-related monitoring, including audits, ethics committee review and regulatory inspections by providing direct access to source data and documents as required. Patients' consent for this must be obtained. A list of source data use for the trial and their locations should be maintained by the site.

10.1.4 Monitoring Visits to Investigator Sites

A selection of institutions will be visited during the course of the STAMPEDE trial. The CTU will give the responsible investigator prior notice of the monitoring visit to allow adequate time, space and staff for these visits. The standard operating procedures (SOPs) for monitoring are available from the CTU.

After the monitoring visit the monitor will complete a site visit report. Once the TMT have reviewed the report and agreed on any recommendations the monitor will finalise the report and send a copy to the Principal Investigator (PI) at the site. A copy will be kept in the CTU STAMPEDE Trial Master File.

Remote or self- monitoring could be utilised through the course of the trial. Site staff may be asked to scan and send anonymised sections of a participant's medical record to the CTU for remote verification or asked to complete a form to confirm compliance with protocol procedures.

10.2 CONFIDENTIALITY

All information collected during the course of the research will be kept strictly confidential. In addition, all procedures for handling, processing, storage and destruction of data are compliant with the Data Protection Act 1998. No individual participants will be identified when results from the trial are published.

Participants are asked to give their permission for information about their health status to be obtained from the Office of National Statistics (ONS), via NHS Digital (formerly HSCIC), Public Health England, National Cancer Research Advisory Service, or any similar or national equivalent. This will facilitate data collection and verification and reduce the burden on sites. In addition, participants will be asked for permission to inform their GP of their involvement in the STAMPEDE trial.

11 SAFETY REPORTING

The principles of GCP require that both investigators and sponsors follow specific procedures when reporting adverse events/reactions in clinical trials. These procedures are described in this section of the protocol and in **Section 7.1.5**.

Further information on the expected toxicities for the protocol treatments (investigational medicinal products (IMPs)) being tested in arms on active follow-up can be found in the reference safety information (RSI) accessible via the STAMPEDE website: http://www.stampedetrial.org/centres/essential-documents/reference-safety-information-rsi/.

11.1 SAFETY REPORTING DEFINITIONS

The definitions of the EU Directive 2001/20/EC Article 2 based on the principles of GCP apply to this trial protocol. These definitions are given in **Table 36**.

Table 36: Event Terms and Definitions

TERM	DEFINITION
Adverse Event (**AE**)	Any untoward medical occurrence in a clinical trial participant to whom a medicinal product has been administered. These include occurrences which are not necessarily caused by the product.
Adverse Reaction (**AR**)	Any untoward and unintended reaction to an investigational medicinal product related to any dose administered.
Unexpected Adverse Reaction (**UAR**)	An adverse reaction, the nature or severity of which is not consistent with the information about the medicinal product in question set out in reference safety information (summary of product characteristics or Investigator brochure) for that product.
Serious Adverse Event (**SAE**) or Serious Adverse Reaction (**SAR**) or Suspected Unexpected Serious Adverse Reaction (**SUSAR**)	Respectively any adverse event, adverse reaction or unexpected adverse reaction that fulfils the definition of **serious:**<br>• results in death<br>• is life-threatening*<br>• requires hospitalisation or prolongation of existing hospitalisation**<br>• results in persistent or significant disability or incapacity<br>• consists of a congenital anomaly or birth defect<br>• Other important medical condition***

Clarifications and Exceptions

*The term 'life-threatening' in the definition of 'serious' refers to an event in which the participant was at risk of death at the time of the event; it does not refer to an event which hypothetically might have caused death if it were more severe.

**Hospitalisation is defined as an inpatient admission, regardless of length of stay, even if the hospitalisation is a precautionary measure for continued observation. A&E attendances are not defined as a hospitalisation unless participants are admitted. Hospitalisations for a pre-existing condition, not thought to have been exacerbated by STAMPEDE protocol treatment or IMPs (including elective procedures that have not worsened) do not constitute an SAE.

*** Medical judgement should be exercised in deciding whether an AE/AR is serious in other situations. Important AE/ARs that are not immediately life-threatening or do not result in death or hospitalisation but may jeopardise the

participant or may require intervention to prevent one of the other outcomes listed in the definition above, should also be considered serious.

11.1.1 Adverse event definitions

Adverse events (AE) include:

- An exacerbation of a pre-existing illness
- An increase in frequency or intensity of a pre-existing episodic event or condition
- A condition (even though it may have been present prior to the start of the trial) detected after trial drug administration
- Continuous persistent disease or a symptom present at baseline that worsens following administration of the study treatment

NB: Within STAMPEDE non-melanoma skin cancers (e.g.: basal cell carcinoma and squamous cell carcinoma) are not considered important medical conditions and therefore are considered adverse events, unless they fulfil any of the other "serious" criteria – detailed in Table 36.

Serious Adverse Events (SAE) are AEs that fulfil the definition of serious as detailed in Table 36**.** SAEs are reported using the SAE CRF. If the event is assessed as possibly, probably or definitely related to a protocol treatment (IMP), it is categorised as a Serious Adverse Reaction (SARs). If the reaction is unexpected based on the approved reference safety information, it is categorised as a Suspected Unexpected Serious Adverse Reaction (SUSAR), see Table 37**.**

Notable Adverse Events (NAE) these include pregnancy occurring in a partner of a STAMPEDE participant. Pregnancies must be followed up until outcome, whether this is a live birth, stillbirth, or planned or spontaneous abortion. NAEs should be reported on the SAE CRF, in the same manner as SAEs.

11.1.2 Defining "treatment" for the purposes of safety reporting

STAMPEDE is an adaptive platform protocol in which research treatments are given in addition to standard-of-care (SOC) therapies, or as alternatives in the case of transdermal oestradiol. As per MHRA recommendations **all protocol treatments** (i.e. both protocol SOC and protocol research treatments) and are regarded as **investigational medicinal products** (IMPs) within the STAMPEDE platform for the purposes of safety reporting.

Protocol treatments (IMPs):

- **Protocol SOC treatments** are IMPs that are standard forms of treatment permitted as part of the STAMPEDE protocol.
    - Licensed ADT (e.g. LHRH analogues) given in the setting of hormone-sensitive prostate cancer
    - Docetaxel given in hormone-sensitive prostate cancer
    - Abiraterone given in hormone-sensitive prostate cancer
    - Enzalutamide given in hormone-sensitive prostate cancer
    - Apalutamide given in hormone-sensitive prostate cancer

Please note, if a participant allocated to transdermal oestradiol switches to standard ADT in the absence of progression, this would still be considered as being on protocol treatment (IMP).

- **Protocol research treatments** are the IMPs that are additional or alternative treatments participants allocated to research arms on active follow-up (G-L) receive as part of the STAMPEDE protocol:

- o Arm G: abiraterone
- o Arm J: abiraterone & enzalutamide
- o Arm K: metformin
- o Arm L: transdermal oestradiol

Note, the research treatment in arm H (prostate RT) is not an IMP, but safety reporting requirements to the CTU are the same.

Non-protocol treatments:

- All prostate cancer treatments commenced post disease progression (as defined in the protocol – **Section 7.1.3**).
- ADT given after progression, (e.g.: commenced in HSPC setting and now continues for the management of CRPC, or ADT given after progression after completing M0 course of treatment - See **Figure 5**).

11.2 SITE INVESTIGATOR RESPONSIBILITIES

The Site Investigator may be any medically qualified individual delegated to undertake safety reporting for the STAMPEDE trial. It is recommended that the Principal Investigator delegate safety reporting to at least one other individual in order to ensure reporting cover during their absence.

11.2.1 Notification period

All events that fall within the notification period must be reported, events outside the notification period do not need reporting.

Adverse Events (AEs): All AEs are reportable from the time of randomisation until 30 days after discontinuation of protocol treatment (IMPs)* (refer to **Section 11.1.2**). All AEs should be recorded in the participant's medical notes and on the Toxicity (AE) CRF linked to the Follow-up CRF. The Toxicity (AE) CRF should be sent to the CTU within one month of the corresponding Follow-up CRF being due.

Serious Adverse Events (SAEs): All unrelated events i.e. SAEs are reportable from the time of randomisation until the participant has progressed AND is 30 days after discontinuation of all protocol treatment (IMPs)* (refer to **Section 11.1.2**) or comparison closure (see **Section 7.4** for definition of comparison closure).

*N.B. ADT before progression is a protocol SOC treatment (IMP). However, even though ADT following progression is not protocol SOC treatment (IMP) the reporting period continues until depot expiry of the last dose before progression + 30 days is completed. Therefore when the participant is on ADT in the form of LHRHa, this is assumed to be 30 days after the depot expiration date (e.g. up to 8 weeks after administration of a 4-week depot or 16 weeks after administration of a 12-week depot) following the final dose given before progression was diagnosed (See **Figure 5**).

Serious Adverse Reactions (SARs and SUSARs): All related SAEs i.e. all SARs and SUSARs are reportable from the time of randomisation until comparison closure (see **Section 7.4** for definition of comparison closure).

Notable Adverse Events: All notable adverse events are reportable from randomisation until comparison closure, using the SAE CRF. Notable Adverse Events must be reported within 24 hours of the investigator being made aware of the event using the SAE CRF.

All SAEs must be reported within 24 hours of the investigator being made aware of the event using the SAE CRF and the Investigator is responsible for providing follow up information for SAEs until resolution.

Figure 5: Diagram to show notification/reporting period for AEs and SAEs occuring on LHRHa

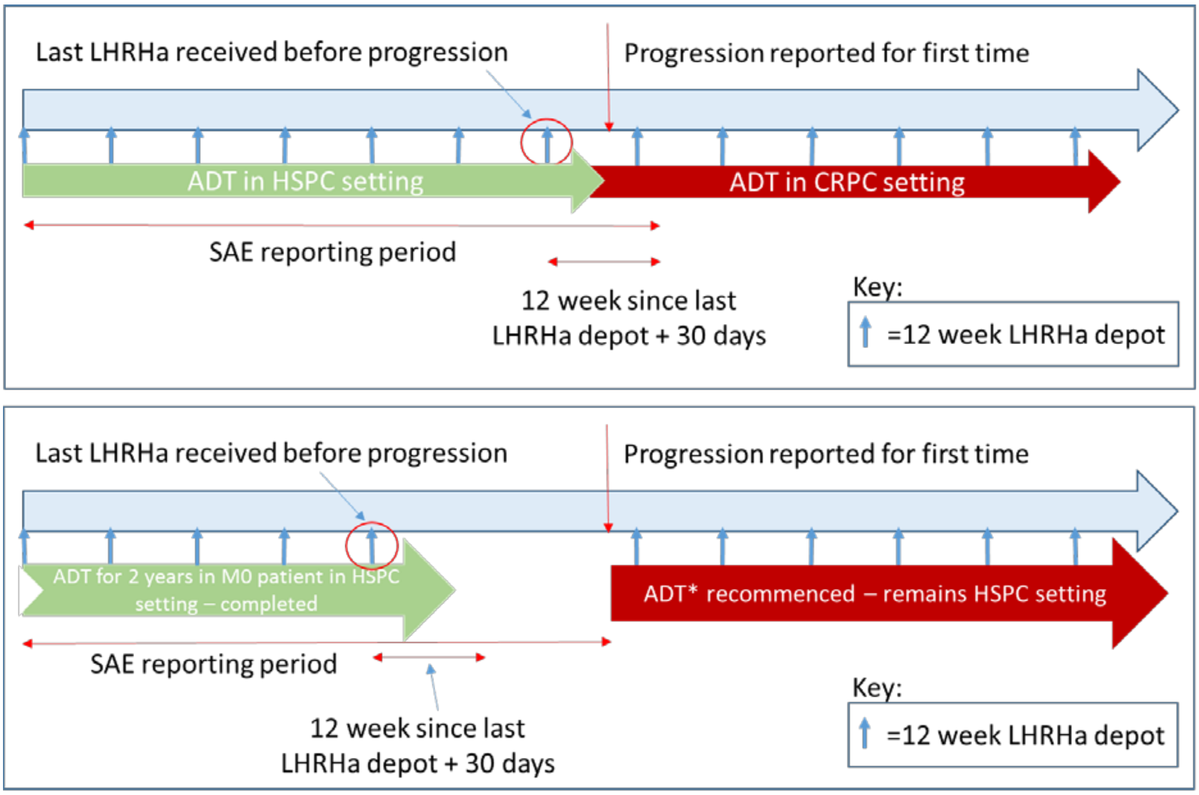

*Any additional treatment started at this stage eg: abiraterone would not be considered IMP

11.2.2 Trial-Specific "Expedited Reporting" Exemptions

The following events which may fulfil the definition of "serious" are exempt from expedited reporting. They are still require to be reported as an AE on the Toxicity (AE) CRF, or on an alternative CRF e.g. progression log, which will be used to report these events to the MHRA, but an SAE CRF is not required.

- **Death as a result of disease progression or disease-related deaths:** Do not complete an SAE CRF, unless death is considered to be caused by trial treatment (i.e. a SAR). The details should be reported on the Death Form.

- **Non-fatal progression events:** events that fulfil the definition of serious e.g. result in hospital admission, but are due to disease progression are exempt from reporting as an SAE, instead details should be provided on the Progression Log.

- **Elective hospitalisation** and surgery for treatment of locally-advanced or metastatic prostate cancer or its complications. These should be recorded as a non-trial inpatient admission on the follow-up form under Non-Trial visits.

- **Elective hospitalisation** to simplify treatment or procedures. If related to prostate cancer, record as non-trial inpatient admission on the follow-up form.

11.2.3 Investigator Assessment

11.2.3.A Seriousness

When an AE occurs the investigator or delegate **must** assess whether the event is serious. Refer to **Table 31** for what fulfils the criteria of serious and **Section 11.2** for a list of exemptions from expedited reporting.

11.2.3.B Grading severity of adverse event

The severity (i.e. intensity) of all AEs **must** be graded using Common Terminology Criteria for Adverse Events (CTCAE) v4.03. The complete CTCAE v4.03 can be found at:
http://www.stampedetrial.org/centres/tools-training/training-materials-resouces/
Any questions concerning this process should be directed to the CTU team in the first instance.

11.2.3.C Causality

The Investigator **must** assess the causal relationship of all serious events or reactions in relation to protocol treatment using the definitions in **Table 32**.

11.2.3.D Notification responsibilities for non-protocol treatments

It should be noted that ADT, docetaxel, abiraterone, enzalutamide and apalutamide may be given as non-trial treatments in the management of CRPC. It is not necessary to report AEs or SAEs relating to this non-trial use where the treatment commenced post progression. Instead the yellow card system should be used to notify the regulatory authorities of adverse drug reactions in this setting: (https://yellowcard.mhra.gov.uk/)

Figure 6: SAE reporting flowchart

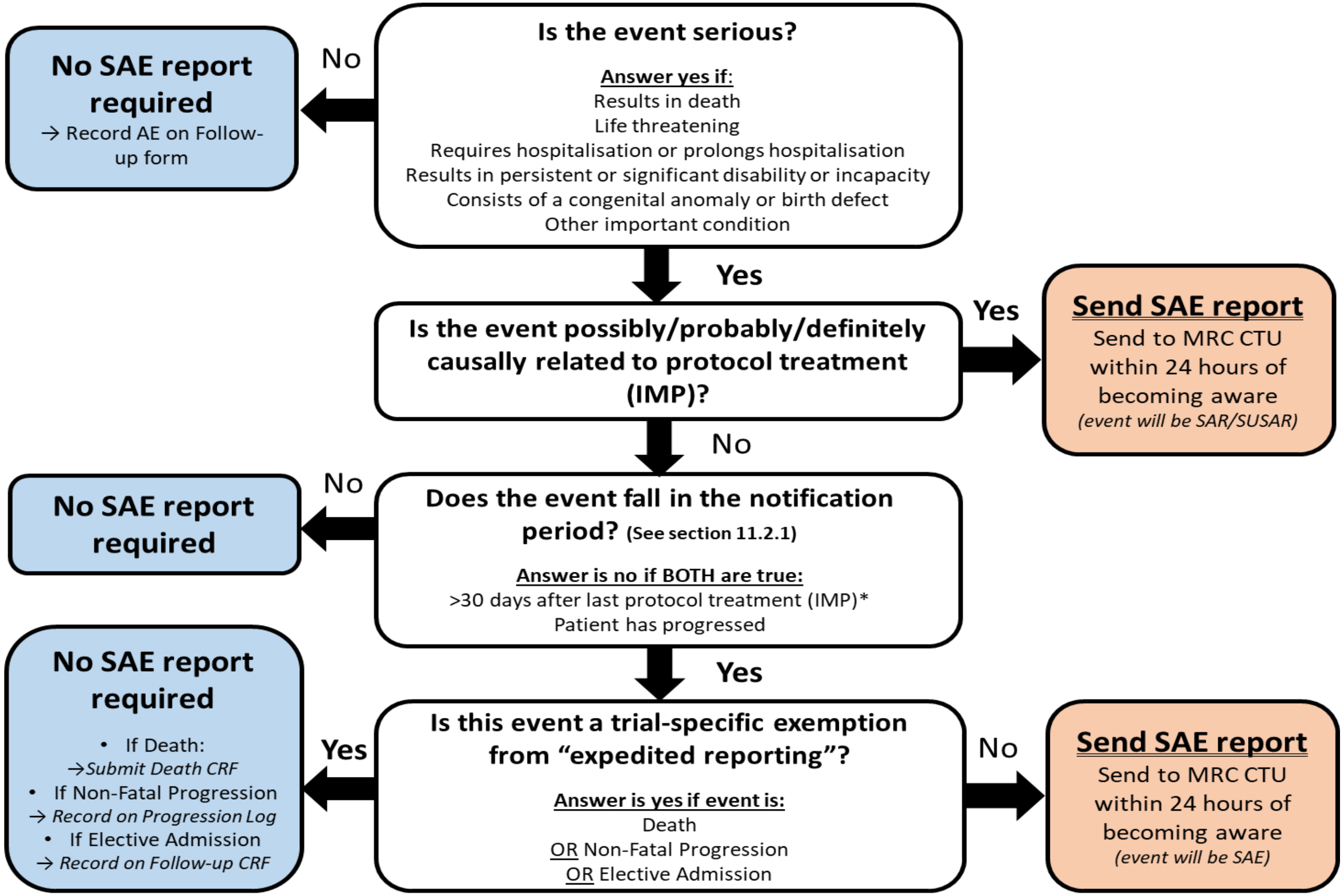

**Exposure to LHRHa is assumed to be until the depot expiration date, therefore unrelated SAEs are reportable up until 8 weeks after the administration of a 4-week depot or 16 weeks after the administration of a 12-week depot.*

Box 1: SAE report notification checklist

Before sending the SAE CRF please check that the event falls within the notification period and does not meet any of the "exemption from expedited reporting criteria", see **Section 11.2**. The SAE CRF must be submitted within 24 hours of an Investigator becoming aware of the event. The following are the minimum criteria required for initial processing and review:

1. At least **two** patient identifiers
2. **One event term** that can be coded to CTCAE version 4.03
3. Indication of why the event was **serious**
4. **Grade** severity of event/reaction according to CTCAE version 4.03
5. Date of **onset** when the event met the criteria of serious. Please refer to **Table 31**
6. Provide details for **all protocol treatments (IMPs) allocated** (i.e. both protocol SOC and protocol research treatments, including any allocated protocol research treatment that has not started) whether ongoing or completed at time of event onset.
7. Assessment of **causality** in relation to **each** protocol treatment (i.e.: both protocol SOC and protocol research treatments, including any allocated protocol research treatment that has not started) – *please note this can be provided later if clinician is not available within 24 hours of becoming aware of the event. This can be completed by the trial team based on correspondence with site clinician, and signed by the clinician at a later date.*
8. **Signature** (This can be a site trial team member in the first instance to meet the reporting timelines, but the CRF must be re-sent once a clinician has reviewed and signed the form)

SAE REPORTING
Fax to 020 7670 4818 within 24 hours of becoming aware of the event
Or send via **encrypted** email to mrcctu.stampede@ucl.ac.uk

11.2.4 Event Follow-up

Participants must be followed up until clinical recovery is complete or stabilised (resolution of the event – this can include an outcome that the event is "resolved with ongoing sequela"). Follow-up should continue after completion of protocol treatment if necessary. The Investigator is responsible for providing follow up information for SAEs until resolution. Follow-up information should be updated on the original SAE CRF by ticking the box marked "follow-up" and faxing to the CTU as information becomes available. Extra information and/or copies of test results may be provided separately but must be anonymised. The participant must be identified by trial ID and initials only. The participant's name should not be used on any correspondence.

11.3 CTU RESPONSIBILITIES

The STAMPEDE trial team will acknowledge receipt of all SAEs via email. Please contact the STAMPEDE trial team if an acknowledgement email is not received within 3 working days.

At least one medically qualified person at the CTU, or comparison chief-investigator or another appropriate TMG member will review all SAE reports received. The rationale for answers provided can be discussed between the site and CTU, however, ultimately the causality assessment given by the local Investigator at the hospital cannot be overruled.

The CTU is undertaking the duties of trial sponsor and is responsible for the reporting of SUSARs and other SARs to the regulatory authorities (through the MHRA to competent authorities in other European member states) and the UK research ethics committees. Additionally, the CTU has sponsor oversight for reporting in other countries in which the trial is taking place. The CTU is responsible for reporting fatal and life-threatening SUSARs to the UK competent authorities within 7 days of the CTU becoming aware of the event; other SUSARs must be reported within 15 days.

SAKK (Schweizerische Arbeitsgemeinschaft für Klinische Krebsforschung), the Swiss Group for Clinical Cancer Research coordinate site participation for STAMPEDE, and are responsible for reporting SUSARs to the relevant Swiss competent authority and lead ethics in accordance to their local regulations, on behalf of the CTU.

The CTU will also keep all investigators informed of any safety issues that arise during the course of the trial.

The CTU will submit Annual Safety Reports in the form of a Developmental Safety Update Report (DSUR) to Competent Authorities (Regulatory Authority and Ethics Committee).

Any drug companies involved will also be notified of reportable (serious and unexpected and drug-related/unknown relationship) events as per their agreement with the sponsor. CTU will also provide companies with a copy of the Annual Safety Report in the required format.

11.3.1 Sponsor (CTU) Assessment

11.3.1.A Expectedness

If there is at least a reasonable possibility of causal relationship to the protocol treatment (all IMPs i.e. SOC and research), an assessment of the expectedness of the event will be made by the Sponsor (the STAMPEDE team at the CTU). This determines whether a reaction is a SAR or SUSAR, see **Table 37**.

Expectedness is determined using the current reference safety information (RSI) (i.e. summary of product characteristics section 4.8 or current investigator brochure) approved for the trial. An event is considered unexpected if it is:

- Not listed in the RSI
- If severity exceeds that listed in the RSI
- If frequency exceeds that listed in the RSI
- If event outcome exceeds that listed in the RSI

Table 37: How causality and expectedness determine event outcome (SAE/SAR/SUSAR)

<table>
<tr><th rowspan="2">CAUSAL RELATIONSHIP (RELATEDNESS)</th><th rowspan="2">DESCRIPTION</th><th colspan="2">EXPECTEDNESS ASSESSED BY CTU</th></tr>
<tr><th>EXPECTED REACTION</th><th>UNEXPECTED REACTION</th></tr>
<tr><td>Definitely</td><td>There is clear evidence to suggest a causal relationship and other possible contributing factors can be ruled out.</td><td rowspan="3">SAR</td><td>SUSAR</td></tr>
<tr><td>Probably</td><td>There is evidence to suggest a causal relationship and the influence of other factors is unlikely.</td><td>SUSAR</td></tr>
<tr><td>Possibly</td><td>There is some evidence to suggest a causal relationship (e.g. because the event occurs within a reasonable time after administration of the trial medication). However, the influence of other factors may have contributed to the event (e.g. the participant’s clinical condition, other concomitant treatments)</td><td>SUSAR</td></tr>
<tr><td>Unlikely</td><td>There is little evidence to suggest there is a causal relationship (e.g. the event did not occur within a reasonable time after administration of the trial medication). There is another reasonable explanation for the event (e.g. the participant’s clinical condition, other concomitant treatment).</td><td colspan="2">Unrelated SAE
No assessment required as unrelated to treatment</td></tr>
<tr><td>Unrelated</td><td>There is no evidence of any causal relationship</td><td colspan="2">Unrelated SAE
No assessment required as unrelated to treatment</td></tr>
</table>

12 ETHICAL CONSIDERATIONS AND APPROVAL

12.1 ETHICAL CONSIDERATIONS

12.1.1.A Randomisation

This is a randomised trial therefore neither the participants nor their physicians will be able to choose the participants' treatment. Treatment will be allocated randomly using a computer-based algorithm. This is to ensure that the groups of participants receiving each of the different treatments are as similar as possible.

All participants, with the exception of those allocated to transdermal oestradiol (Arm L), will receive standard hormone treatment. All participants, including those allocated to Arm L, may also receive other standard-of-care (SOC) treatments such as prostate radiotherapy and/or docetaxel, abiraterone, enzalutamide or apalutamide. Use of these SOC treatments will be unaffected by trial participation and is left to the discretion of the treating clinician and participant.

Participants may be randomised to receive additional treatment (metformin) given with standard-of-care treatments, or an alternative form of hormone treatment (transdermal oestradiol). An even allocation ratio is being currently being used which means all eligible participants have an equal chance of being randomised to the control or research arms.

Through the introduction of a "transdermal oestradiol comparison" into the STAMPEDE trial platform, sufficient data will be collected to evaluate this treatment approach more rapidly. By undertaking a meta-analysis using data collected in both PATCH and STAMPEDE trials, fewer participants overall are allocated the control arm i.e. more participants gain access to novel treatments and results will be available sooner.

12.1.1.B Evaluation of Novel Therapeutic Strategies

There is some evidence to suggest that the newer treatment options may have advantages over standard treatment alone with regards to clinical outcome, but this is not confirmed and toxicity may be increased. This trial will follow a large group of people who have been randomly allocated to either the standard treatment(s) or the novel treatment strategies in order to measure the benefits of these approaches. All participants will be followed-up for toxicity and safety issues, so that any benefits can be weighed against any negative aspects, including the impact treatments have on other aspects of medical health e.g. cardiovascular disease, as well as quality-of-life and value for money (health economic analysis).

12.1.1.C Additional Tests and Hospital Visits

Trial participants will have some additional hospital visits and some extra blood samples compared with standard practice, the exact requirements depend on the allocated treatment and stage of disease. Efforts are made to reduce the burden of extra visits and tests, for example extra blood tests can be performed at a time when a blood draw would be performed as part of standard care, or participants can have the blood samples taken at their GP's surgery instead.

12.1.1.D Facilitating Participant Feedback From Investigations and Additional Analyses

For participants who choose to take part in additional sub-studies, biological samples including blood, saliva and remaining stored FFPE tumour samples will be used in research projects. These projects will enable the study of genetic factors and other biomarkers that can help identify individuals who serve to benefit most from the treatments tested in STAMPEDE, and to further understand why and how treatment resistance develops. All samples will remain anonymised and only made accessible to approved collaborators granted access by the STAMPEDE oversight

committees. We will make every effort to protect the confidentiality of this information and make sure personal identities are protected.

From protocol v16.0 onwards, participants may opt to receive feedback regarding genetic results that may arise from the research analyses of genetic material extracted from any of the biological samples collected as part of the trial e.g. saliva, FFPE tumour blocks or circulating tumour DNA extracted from blood. Only results which are of established clinical relevance and for which testing would be available under standard NHS genetic testing guidelines will be fed back. Any genetic analysis undertaken as part of additional research associated with STAMPEDE does not replace clinically indicated investigations as only a proportion of STAMPEDE participants will undergo prospective testing and therefore it cannot be guaranteed that results will be fed back in a timely fashion.

This change has been made in response to emerging data that demonstrates a small proportion of people may have genetic faults in genes such as Breast Cancer Gene 2 (BRCA2). This has implications for both participants and potentially their biological relatives. For participants and their treating clinician, knowledge of this information may facilitate access into further clinical trials and may potentially impact on the choice of treatment following progression.

Any participant who consents to receive feedback and in whom a known pathogenic mutation of clinical significance is detected on testing of research samples collected as part of STAMPEDE will be told of this. Participants will be recommended to undergo genetic counselling accessed via clinical genetics services and consider confirmatory testing. This is necessary to determine if the defect is germline (inherited) and ensures access to appropriate ongoing support. If confirmed as a germline (inherited) abnormality, this will enable biological relatives to also access appropriate genetic counselling and testing if they wish.

The introduction of the “metformin comparison” means that all participants, not known to be diabetic, will be screened for diabetes prior to trial entry. This is to enable the effect of metformin to be studied in non-diabetic participants. All participants in whom screening bloods are abnormal will be referred for confirmatory tests and further management according to local guidelines e.g. via their GP. Screening is expected to lead to a small proportion of potential trial participants receiving a new diagnosis of diabetes but will ensure appropriate management of both conditions.

12.1.1.E Considering the Impact of Emerging Data

If new information emerges during the course of the trial which may affect the treatment or follow-up of participants all Principal Investigators (PIs) will be informed of this and required to inform trial participants.

12.1.1.F Electronic health records

Participants are requested to provide consent to permit linkage of trial data to other sources of electronic health data to improve the reliability of long-term follow-up data. Explicit consent is requested for the CTU to store direct identifiers (name and NHS number) securely and seperately from anonymised trial data. This is to permit verification of the information held by others and received by the CTU, ensuring that the trial database is only updated with accurate information.

12.2 ETHICAL APPROVAL

The protocol has a Favourable Opinion from an appropriate Research Ethics Committee, according to national guidelines. Additionally, each site must also obtain management permission for research (Local R&D approval or equivalent) from the relevant host organisations before participants can be entered into the trial. The participant’s informed consent to participate in the trial should be

obtained after a full explanation has been given of the treatment options, including the conventional and generally accepted methods of treatment. Participant information sheets and participant consent forms are available on the STAMPEDE website (www.stampedetrial.org).

The right of the participant to refuse to take part in the trial without giving reasons must be respected. After the participant has entered the trial, the clinician must remain free to give alternative treatment to that specified in the protocol, at any stage, if he feels it to be in the best interest of the participant. However, the reason for doing so should be recorded and the participant will remain within the trial for the purpose of follow-up and data analysis according to the treatment option to which he has been allocated. Similarly, the participant must remain free to withdraw at any time from the protocol treatment without giving reasons and without prejudicing his further treatment.

A statement of MRC policy on ethical considerations in clinical trials of cancer therapy, including the question of informed consent, is available from the MRC Head Office web site (http://www.mrc.ac.uk). In addition, the MRC and the Wellcome Trust framework on the feedback of health-related findings in research is readily available (https://www.mrc.ac.uk/documents/pdf/mrc-wellcome-trust-framework-on-the-feedback-of-health-related-findings-in-researchpdf/) and has been used when developing the trial specific processes.

13 REGULATORY APPROVAL

This trial has been approved in the UK by the MHRA and will be conducted under a Clinical Trials Authorisation CTA 20363/0404/001 in the UK.

The trial has been approved in Switzerland by Swissmedic (Ref: 2009 DR 3235).

13.1 DATA COLLECTION & RETENTION

CRFs, clinical notes and administrative documentation should be kept in a secure location (for example, locked filing cabinets in a room with restricted access) and held for 25 years after the end of the trial. During this period, all data should be accessible to the competent or equivalent authorities, the Sponsor, and other delegated authorities with suitable notice as it may be subject to audit or inspection from any of the above.

14 INDEMNITY

University College London holds insurance against claims from participants for injury caused by their participation in this clinical trial. Participants may be able to claim compensation if they can prove that UCL has been negligent. However, as this clinical trial is being carried out in a hospital, the hospital continues to have a duty of care to the participant of the clinical trial. University College London does not accept liability for any breach in the hospital's duty of care, or any negligence on the part of hospital employees. This applies whether the hospital is an NHS Trust or otherwise.

Participants may also be able to claim compensation for injury caused by participation in this clinical trial without the need to prove negligence on the part of University College London or another party. Participants who sustain injury and wish to make a claim for compensation should do so in writing in the first instance to the Chief Investigator, who will pass the claim to the managing organisation's Insurers, via the managing organisation's office.

Hospitals selected to participate in this clinical trial must provide clinical negligence insurance cover for harm caused by their employees and a copy of the relevant insurance policy or summary should be provided on request.

15 FINANCE

STAMPEDE is funded by Cancer Research UK's Clinical Research Committee (formerly the Clinical Trials Advisory Awards Committee; CTAAC). It is also funded by the MRC through the MRC Clinical Trials Unit at UCL. The trial has National Institute for Health Research Clinical Research Network (NIHR CRN) approval and, therefore, local NCRN funds may be available at each site to support entry of participants into this trial.

Funding arrangements for research arms and sub studies now closed to recruitment can be found in earlier protocols.

Standard therapies including **ADT**, **prostate radiotherapy** and **docetaxel** will be administered as per routine clinical care using local NHS supplies.

Abiraterone is manufactured by Janssen Pharma PV (pharmaceutical companies of Johnson & Johnson). They have agreed to provide free drug, funds to distribute drug to participating sites and to help support the conduct and management of the trial.

If abiraterone is required to be given to participants as standard of care, funding will not be provided and the drug should be administered as per routine clinical care using local NHS supplies.

Enzalutamide is manufactured by Astellas Pharma. They have agreed to provide free drug and funds to distribute drug to participating sites and to help support the conduct and management of the trial.

If enzalutamide is required to be given to participants as standard of care, funding will not be provided and the drug should be administered as per routine clinical care using local NHS supplies.

Metformin will be administered using local NHS supplies.

Transdermal oestradiol will be administered as either Progynova TS 100 patches, manufactured by Bayer, or Femseven 100 patches, manufactured by Theramex who have agreed to supply these patches at a trial-specific discounted price. All accredited STAMPEDE sites will be able to order Progynova for use in the STAMPEDE trial through Alliance Healthcare Ltd wholesalers and Femseven patches through AAH Pharmaceuticals Ltd wholesalers.

Apalutamide given to participants as standard of care will be administered using local NHS supplies.

16 TRIAL COMMITTEES

16.1 TRIAL MANAGEMENT GROUP (TMG)

A Trial Management Group (TMG) has been formed comprising: the Chief Investigator; each comparison lead investigator; other co-investigators and members of MRC CTU at UCL internal Trial Management Team. The membership of the TMG may be expanded if other groups of trialists wish to participate. It will also be amended during the trial if other circumstances require e.g. retirement.

The TMG will be responsible for the day-to-day running and management of the trial. The TMG will meet by teleconference at least on a monthly basis where possible and in person as needed.

Further details of TMG functioning are provided in the TMG charter (available on request).

16.2 TRIAL STEERING COMMITTEE (TSC)

A Trial Steering Committee (TSC) has been formed to provide overall supervision for the trial and provide advice through its independent chair. The ultimate decision for the continuation of the trial lies with the TSC. The TSC will meet regularly, as required by the trial, and at least annually.

The relationship of the TSC with the other STAMPEDE working groups is detailed in **Figure 7**. Further details of TSC functioning are provided in the TSC charter (available on request).

16.3 INDEPENDENT DATA MONITORING COMMITTEE (IDMC)

An Independent Data Monitoring Committee (IDMC) has been formed. The IDMC will be the only group who sees the confidential, accumulating data to the trial. Reports to the IDMC will be produced by the CTU. The IDMC will meet within 6 months of the trial opening with the frequency of meetings dictated by the IDMC. The IDMC will consider data in accordance with the analysis plan (see **Section 9**) and will be advisory to the TSC. The IDMC can recommend premature closure or reporting of the trial, or that recruitment to any research arm is discontinued.

From protocol v8.0 onwards, any recommendation from the IDMC to stop recruitment to one or more trial arms will be acted upon immediately, pending ratification from the TSC. As this period between meetings should be very short, sites would not be notified until after the TSC have made a decision. IDMC recommendations based on emerging safety issues will be discussed with sites promptly.

The relationship of the IDMC with the other STAMPEDE working groups is detailed in **Figure 7**. Further details of IDMC functioning and the procedures for interim analysis and monitoring are provided in the IDMC charter (available on request).

Data from the “transdermal oestradiol comparison” are viewed by the PATCH IDMC, in meta-analysis with PATCH, rather than by the STAMPEDE IDMC. Recommendations of any actions relating to STAMPEDE would be made to the STAMPEDE TSC.

16.4 TMG SUB-GROUPS AND EXPERT PANELS

The trial has a number of TMG sub-groups and expert panels, each comprising of specific members of the TMG, MRC CTU at UCL, field experts and other STAMPEDE clinicians and site staff. The groups are all chaired by TMG members and report directly into the TMG.

- The **Biological Research Group** (BRG), the **Bone and Imaging Group** (BIG) and the **Metabolic Translational Group** (MTG) all input and provide expert oversight of relevant translational aspects of the trial and associated sub-studies.
- The **STRATOSPHere Consortium Management Group** (STRATOSPHere: STratification for RAtional Treatment-Oncomarker pairings of STAMPEDE Participants starting long-term Hormone treatment) coordinates the parallel translational programme funded by Prostate Cancer UK.
- The **Comparison Management Groups** (CMGs) were developed to input into the running of each comparison and to propose, plan and develop new comparisons as required. The CMGs are comprised of:
    - Arm G – Abiraterone CMG
    - Arm H – M1|RT CMG
    - Arm J - Abiraterone and Enzalutamide CMG
    - Arm K – Metformin CMG
    - Arm L – tE2 CMG
    - Future proposals CMG
    - Original comparisons CMG
- The **Site Advisory Team** (SAT) includes STAMPEDE site research staff to provide advice to the TMG concerning the running of the trial, including how proposed amendments to the protocol and CRFs directly affect staff practices.
- The **Outcome Review Group** (ORG) conducts cause of death reviews as required for secondary end point analysis.
- The **Clinical Safety Committee** (CSC) review all SAEs of STAMPEDE participants and provide guidance to site clinicians and research staff in regards to clinical safety aspects of the trial.
- The **Genetic Sub-Group** (GSG) provides oversight of all results arising from genetic testing.
- The **Quality of Life** group (QOL group) will advise on how to optimise use of QOL data within the STAMPEDE trial

The relationship of each of these groups with the other STAMPEDE working groups is detailed in **Figure 7.**

16.5 MRC CTU AT UCL INTERNAL GROUPS

CTU requires a number of internal working groups to run a platform protocol. These internal groups assist the TMG in the operation of STAMPEDE, providing guidance on scientific strategies of research and publication, research governance in regulatory information and protocol review and the management of research quality within the STAMPEDE trial.

The relationship of each of these groups with the other STAMPEDE working groups is detailed in **Figure 7**.

Figure 7: Organigram of the relationships between STAMPEDE working groups

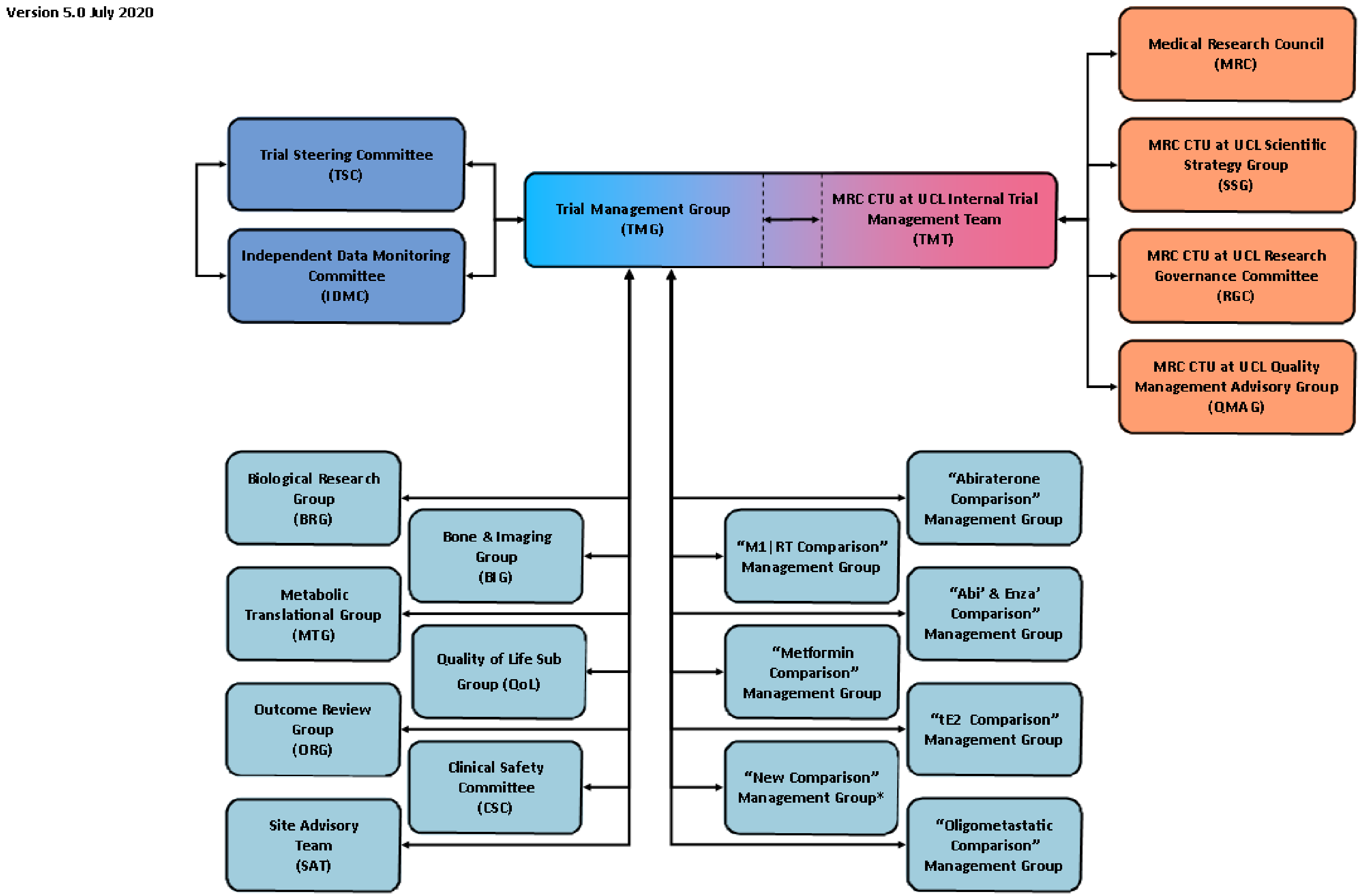

*The number and timeline of current and planned comparisons will dictate the need for the number of CMGs in operation. At any point there may be one or more.

17 ANCILLARY STUDIES

17.1 PATIENT REPORTED OUTCOMES

STAMPEDE collects patient reported outcomes in the form of the EORTC QLQ-30 Quality of Life form and the EQ-5D Health Economics Form.

The research nurse should approach participants at appropriate clinical visits to complete a questionnaire. If no clinical visit is scheduled for the participant (with a window of 4 weeks around the expected date) the nurse should organise the completion of the questionnaire e.g. by post or secure e-mail.

Questionnaires should be self-administered; participants should be encouraged to complete the questionnaires without conferring with friends or relatives and all questions should be answered even if the participant feels them to be irrelevant. The research teams should encourage the participants to answer all questions but should not review the responses as these should remain confidential. Copies of questionnaires should not be retained at site.

17.1.1 Quality of life (QL)

The EORTC QLQ-C30 with the prostate-specific module QLQ PR25 will be used. Key items for assessment are pain reduction for participants with metastatic disease and urinary symptoms for participants with locally-advanced disease. In addition, specific hypotheses will be generated for each of the research arms.

17.1.1.A Changes in QL data collection from protocol v19.0 onwards

Initial participation in the QL sub-study was limited to the first 700 participants recruited (this was reached in Sep-2008). After a pause, the QL sub-study re-opened from the implementation of protocol version 8.0 (Nov 2011 onwards).

From protocol v19.0, QL and HE data collection changed, as laid out in **Table 33.** QL and HE collection stopped for most participants but continued as planned in participants in the “abiraterone comparison” and “abiraterone and enzalutamide comparison” and became lifelong in participants in the “metformin comparison”. HE collection (without QL) also continued in participants in the “M1|RT comparison” randomised after Apr 2016.

Table 33 summarises the participant reported outcome data collection (QL and HE) by comparison. Going forward, for each new comparison within STAMPEDE, a pre-defined sample size for the participant reported outcomes will be described and a sampling approach considered where appropriate.

17.1.2 Health Economics

The EuroQol (EQ-5D) will be used in the study as a generic measure of health-related quality-of-life which can be linked to public preferences. This data will be used to calculate quality-adjusted life-years as part of the economic evaluation. Healthcare resource use will be collected at each follow-up. This includes non-trial inpatient days, non-trial outpatient, GP visits and data on concomitant medications. Information on participants’ use of primary care and community-based services will also be collected as additional questions in the questionnaire. Costs will be calculated on the basis of representative UK unit costs at the point of analysis. A cost-effectiveness analysis will compare all regimens that continue to recruit into their final Efficacy Stage. For further details please refer to **Appendix G**.

Table 38: Patient reported outcome data collection by comparison

COMPARISON	PARTICIPANT DETAILS	COLLECTION OF PATIENT REPORTED OUTCOMES<br>E.G. EORTC QLQ-C30, EQ-3D
“Original”	Arms B, C, D, E, F and Arm A recruited between trial start (2005) and 15-Nov-2011	No further collection of participant reported outcomes as comparisons have closed to follow-up
“Abiraterone”	Arms A and G randomised between 15-Nov-2011 and 17-Jan-2014	Data collection to continue until disease progression or 5 years post randomisation (i.e. all data collection stops 17-Jan-2019).
“Abiraterone and enzalutamide”	Arms A and J randomised between 29-Jul-2014 and 31-March 2016	Data collection to continue until disease progression or 5 years post randomisation whichever occurs first.
“M1|RT”	Arms A and H randomised between 22-Jan-2013 and 02-Sep-2016	From protocol v19.0 QL and HE data collection will stop for all participants recruited to Arm H prior to April-2016.<br>From protocol v21.0 HE (EQ-5D) data collection will stop along with active follow up for all A and H participants randomised between Apr-2016 to Sep-2016.
“Metformin”	Arms A and K randomised since 05-Sep-2016	From protocol v19.0 the QL and HE sub-studies are closed to newly randomised participants.<br>For all existing arm A and K participants (i.e. randomised prior to activation of protocol v19.0) data collection continues at each follow-up lifelong.
“Transdermal oestradiol”	Arms A and L randomised since 20-Jun-2017	From protocol v19.0 the QL and HE sub-studies are closed to newly randomised participants within this comparison. QL data will be collected through the PATCH trial.

17.2 TRANSLATIONAL SUB-STUDIES

Samples obtained from consenting STAMPEDE participants are analysed as part of separate translational sub-studies. These are conducted through collaborations with other academic and industry partners. All applications for collaboration and sample access are reviewed by the STAMPEDE oversight committees and overseen by the STAMPEDE BRG. For details on eligibility criteria for each translational sub-study refer to **Section 4.7.** For details regarding sample collection refer to the **Sample collection and handling manual** available via the website.

17.2.1 Germline DNA Analysis

DNA is being extracted from saliva samples provided by consenting participants enrolled in STAMPEDE. The purpose of this sub-study is to examine the germline (inherited) genetic changes present in people with high-risk localised or metastatic prostate cancer. The aim is to determine the prevalance of germline genetic aberrations present pre-diagnosis and to correlate prostate cancer risk single-nucleotide-polymorphisms (SNP) genetic profiles, identified in Genome-wide Association Studies (GWAS) and other sequence variants from next generation sequencing (NGS), with duration of response to ADT and the experimental treatments tested in STAMPEDE.

All newly randomised trial participants who join arms A, K or L are eligible to join this sub-study. For details relating to Saliva sample collection and shipping refer to the **Sample collection and handling manual.**

17.2.2 Circulating Tumour-DNA Analysis (Sequential Blood Samples)

The aims of this analysis include identification of molecular subgroups with differential treatment effects and, through sequential sampling, identification of molecular changes associated with disease progression to explore resistance mechanisms and early detection of treatment failure.

Sequential samples are required in order to detect genetic changes within tumours over time. The most important sampling timepoint is at progression, as it is hoped this can inform the potential mechanisms of treatment resistance. The sampling schedule is different for M0 and M1 participants and is detailed in the **Sample collection and handling manual**.

For details relating to blood sample collection including eligibility criteria and shipping refer to the **Sample collection and handling manual.**

17.2.3 Tissue Sample Analysis (FFPE Blocks)

As the clinical outcome data matures for several of the treatments comparisons evaluated within STAMPEDE, correlative analysis of the archival formalin-fixed paraffin-embedded (FFPE) tumour tissue will be undertaken, aiming to identify if genetic mutations present in prostate cancer cells pre-treatment predict how well each treatment works. In addition, projects providing preliminary prevalence and feasibility data to inform future biomarker-directed randomisations will be conducted.

From 2016 onwards, the CTU has been coordinating the retrieval of archival tumour blocks from selected consenting STAMPEDE participants. These samples are usually stored as FFPE tissue blocks at the hospital where the procedure was performed. Randomising sites will be asked to assist in the retrieval of FFPE samples when these are requested. Research teams will be required to confirm sufficient consent has been provided and to provide an anonymised copy of the relevant consent form. If not done so already, an anonymised copy of the consent form should also be sent to the CTU, as per **Section 10.1.1.**

For further details on where to check sufficient informed consent, sample processing and shipping and reimbursement, see the **Sample collection and handling manual.**

17.2.4 Biomarker-Screening Pilot

A biomarker-screening pilot was conducted in a subset of STAMPEDE sites. This ran from Dec 2017 to Jun 2018 and has now been completed.

17.2.5 Informed consent to receive results arising from genetic sub-studies

The consent process was updated for participants joining the trial from protocol v16.0 onwards (activated June-2017). Trial participants are asked to provide explicit informed consent if they wish to receive feedback of any results that arise from research analyses of genetic material extracted from any of the biological samples collected as part of the trial e.g. saliva, FFPE tumour blocks or circulating tumour DNA extracted from blood.

Only results which are of established clinical relevance and for which testing would be available under standard NHS genetic testing guidelines will be fed back e.g. pathogenic BRCA2 mutations. Any genetic analysis undertaken as part of additional research associated with STAMPEDE does not replace clinically indicated investigations as only a proportion of STAMPEDE participants will undergo prospective testing. Analyses are conducted on a purely research basis and it cannot be guaranteed that results will be fed back immediately.

STAMPEDE investigators are strongly recommended to refer all participants in whom a clinically relevant genetic result is detected during research analyses to a clinical geneticist. This is to facilitate access to genetic counselling and the required confirmatory testing. This is also necessary in order to offer appropriate advice to biological relatives in the event of confirmatory testing detecting a germline (inherited) abnormality. The list of clinically relevant gene mutations to be fed back will be based on current clinical guidelines. The STAMPEDE Biological Research Group will review this periodically to ensure it remains current and to oversee this process.

Information provided to STAMPEDE participants who joined the trial prior to Protocol version 16.0, stated that any subsequent genetic results would not be linked to them or their families and therefore results will not be provided in this instance. It is possible for trial participants to update their consent by re-consenting to the current Additional Research Consent Form. This should be anonymised and sent to the CTU as per standard procedures, see **Section 10.1.1.**

17.3 DISEASE VOLUMETRIC ANALYSIS SUB-STUDY

Baseline imaging obtained from STAMPEDE participants are accessed and analysed as part of the trial data collection. Collection and analysis will be undertaken in collaboration with partners on the TMG, initially, in ordered to determine disease volume. For details partaking to retrospective imaging centralisation and image handling, please refer to the individual sub study Working Practices available from the CTU. All subsequent applications for collaboration and imaging access are reviewed by the STAMPEDE oversight committees following the usual processes.

17.4 USING ROUTINE DATA TO IDENTIFY CLINICAL TRIAL OUTCOMES

This sub-study is developing methods to explore whether routine data can be used to quickly and accurately capture trial-related events in centrally-held datasets. These methods need to be developed and validated using different sources of routine data and to identify different types of events. These data sources include, but are not limited to, data from the Public Health England (PHE) National Cancer Registration and Analysis Service (NCRAS), the National Radiotherapy dataset (RTDS)

and the Systemic Anti-Cancer Therapy dataset (SACT) and NHS Digital, Hospital Episode Statistics (HES), and Office of National Statistics (ONS) data.

The overall aim of this sub-study is to develop a clinically useable tool, to accurately identify disease driven events and trial outcomes, to help reduce the burden of collecting trial data from traditional participant-investigator contact. By using data that has already been accurately collected in patients that have given appropriate consent, or for whom the appropriate permissions are in place (e.g. via the Confidentiality Advisory Group), it may be possible to improve timeliness, reduce costs and save resources. Development of enhanced ways to obtain trial data is being undertaken, to recalculate analyses already carried out but also to perform secondary analyses not possible with conventionally collected trial data. The projected aim is to utilise validated methods for routine follow-up and/or analysis in the future, as outlined in the protocol, longer term outcome data may be sought via routine data sources.

17.5 METFORMIN METABOLIC SUBSTUDY

The aim of this project is to explore the heterogeneity of metabolic changes associated with ADT and the effect that metformin has on these changes. Multiple blood markers of metabolic and disease status and sarcopenia assessed by cross-sectional imaging will be examined and linked with baseline characteristics and clinical outcomes.

ADT is standard of care for patients with advanced prostate cancer. It is effective but has side effects, one of them being metabolic dysfunction including obesity, sarcopenia, hyperinsulinemia, and insulin resistance. We will assess whether metformin will alter the percentage of patients with a poor prognostic lipid signature. We will explore whether adding metformin improves oncological outcomes through metabolic reprogramming of the host. In addition we want to determine whether the side effects of ADT can be mitigated by metformin, thus potentially decreasing cardiovascular morbidity and mortality. If we find a decrease in sarcopenia by adding metformin this will also be of importance since sarcopenia does not only affect the self-esteem of men, but also correlates with increased morbidity and mortality.

If metformin is associated with an improvement in metabolic parameters or sarcopenia, it could change clinical standard of care very rapidly, even independently of a benefit in cancer-specific or overall survival.

From protocol v21.0 we will collect sequential blood samples at baseline, regular time points throughout the trial and at progression. In addition we will request CT scans and FFPE tissue blocks from sites. The metabolic sub-study will be for participants allocated to the “metformin comparison”.

For eligibility criteria for the metabolic sub-study refer to **Section 4.7.4**.

For details relating to blood sample collection and shipping, please request the **Metabolic Sub-study Sample Collection and Handling Manual** from the CTU.

18 PATIENT AND PUBLIC INVOLVEMENT

Patient and Public Involvement (PPI) in research is defined by INVOLVE (an advisory group established by the NIHR) as research being carried out 'with' or 'by' members of the public rather than 'to', 'about' or 'for' them. INVOLVE intends 'public' to include patients, potential patients, carers and other users of health and social care services, as well as people from organisations that represent people who use services. In some cases, this may include involvement of a trial's participants in guidance or oversight of a trial.

18.1 POTENTIAL IMPACT OF PPI

PPI is in place to have constant patient overview and investment to guide research. Ultimately STAMPEDE has been created to test whether alterations in treatment help to improve outcomes and quality of life of patients. It is essential to have patients' input as they understand what other patients are going through

The nature of STAMPEDE is such that, even after a main analysis of a comparison has been performed, other participants are still being recruited to other arms. We have a duty to participants and the public to disseminate findings and results, both negative and positive. With this in mind, participants are periodically provided with study findings and updates. Study findings are also presented at conferences.

18.2 PATIENT REPRESENTATIVES

Patient representatives are actively involved in the management of STAMPEDE including updates and alterations. Part of their role is to review all material that will enter the hands of a patient or family member. This is to ensure all documentation used is clear, concise and has wording that is appropriate for everyone, as well as conveying the intended information. Patient representatives sit on the Trial Management Group (TMG).

19 PUBLICATIONS

The results from different sites will be analysed together and published as soon as possible. Individual clinicians must not publish data concerning their participants that are directly relevant to questions posed by the study until the TMG has published its report. The TMG together with the STAMPEDE collaborators will form the basis of the writing committee and decide on the nature of publications.

For the “transdermal oestradiol comparison”, as the efficacy analyses will based on relevant data from the STAMPEDE and PATCH trials, TMGs for the two studies will form the writing committee. Any release, of efficacy or safety data, presentation or publication will be agreed with the TSC according to the terms of their charter.

All publications will acknowledge the participating sites and clinicians, and these will be detailed in an appendix to the main report. Papers will have named authors determined by the TMG according to the following principles:

- To be as inclusive as possible where this is practicable
- To ensure that there is justification for anyone to be named as an author
- Reasons for nomination for authorship may include: trial design; grant holding; day-to-day trial oversight (TMG membership); analysis; discussion and interpretation of data; representation for key groups; active participation at large recruiting sites.
- It should be accepted that the people qualifying for authorship will vary over time. In addition, key positions will vary depending on the nature of the publication: clinical lead for clinical papers, statistician lead for methodology papers, translational papers may be led by authors not on the main TMG if appropriate (e.g. the bone sub-study). In the event of any dispute related to authorship or data release, the TSC will be responsible for making the executive decision.

In the presentations, this list of sites will also be shown. The term “the STAMPEDE investigators” will clearly be stated and relevant names included in the presentation credits.

A detailed **Publication Plan** is documented separately.

20 DATA AND/OR SAMPLE SHARING

Data will be shared according to the CTU's controlled access approach and Standard Operating Procedure, based on the following principles:

- No data should be released in response to a data release request that would compromise an ongoing trial, unless specifically for safety reasons.
- There must be a strong scientific or other legitimate rationale for the data to be used for the requested purpose.
- Investigators who have invested time and effort into developing a trial or study should have a period of exclusivity in which to pursue their aims with the data, before key trial data are made available to other researchers.
- The resources required to process requests should not be under-estimated, particularly successful requests which lead to preparing data for release. Therefore adequate resources must be available in order to comply in a timely manner or at all, and the scientific aims of the study must justify the use of such resources.
- Data exchange complies with Information Governance and Data Security Policies in all of the relevant countries.

Data will be available for sharing on successful request and after the main publication for each comparison. Researchers wishing to access STAMPEDE data should contact the TMG via the CTU team in the first instance. All requests must be reviewed and approved by the TMG and TSC prior to release of data. Investigators should in term ensure the CTU team are regularly updated on the progress of their project and any presentation and publication must be in accordance to the agreements in place.

21 PROTOCOL AMENDMENTS

21.1 PROTOCOL

21.1.1 Amendments Made To Protocol Version 1.0 (May-2004)

Administrative changes such as typos, word change etc.

Name additions/changes to:

TMG members

TSC members

IDMC members

'General Information' Section – additional information re. Abridged version of protocol

Section 1.2 – Figure 1, Celecoxib duration amended

Section 1.3 – Figure 2, addition of cardiovascular assessment form, name and timings amended

Section 2.3 – Docetaxel information updated

Section 2.4 – Additional text re dose and duration justification for Celecoxib use.

Section 3 – Title change and content updated

Section 4.2 – New exclusion criteria added

Section 4.3.1 – New investigations added and additional text re testosterone measurements and additional text re. prior celecoxib treatment

Section 6.1.4 – Celecoxib duration amended

Section 6.1.5 – Additional text re. Co-administration of docetaxel and bisphosphonates

Section 6.1.6 – Celecoxib duration amended

Section 6.2.2 – additional docetaxel information

Section 6.2.3 – addition of CVS event history

Section 11 – Safety reporting updated

Section 12.1 – Additional text re. the collection of blood for genetic and serum marker studies

Section 15 – Additional information re. Central Subvention for docetaxel arms

21.1.2 Amendments Made To Protocol Version 1.1 (May-2005)

Section 6.2 Administration and Dose Modifications, subsection 6.2.1 Zoledronic Acid

21.1.3 Amendments Made To Protocol Version 2.0 (Jun-2005)

General Information section – SAE reporting fax number and timeframe added.

Section 1.2 – Addition of anti-androgen use for M0 patients as a method of HT

Section 1.2 – Increase in amount of blood needed & addition tissue sample request.

Section 1.3 Trial Documentation updated to include new table detailing trial documentation ahead of accreditation, the inclusion of the radiotherapy forms and correct case report form timings

Section 2.1 – Addition of anti-androgen use for M0 patients as a method of HT

Section 4.1.3 – Inclusion criteria Vii "Normal testosterone prior to hormone treatment" removed.

Section 4.1.3 - φnote has been omitted and moved to section 4.2 (see number 8)

Section 4.2 – Exclusion criteria added to exclude patients with active peptic ulceration, gastrointestinal bleeding and inflammatory bowel disease.

Section 4.2 – Exclusion Criteria added to exclude patients with planned major dental work

Section 4.3.1 - All blood test timelines changed from 14 days to 28 days.

Section 4.3.1 – Hormone Therapy pre-randomisation deadline extended from 4 weeks to 12 weeks.

Section 4.3.1 – Additional information regarding the use of NSAIDs and cox-2-inhibitors before coming on to the STAMPEDE study and once commenced on study treatment

Section 4.3.2 – Updated to ask for all vitamins and minerals the patient is taking to be recorded.

Section 4.3.3 – Updated to include the extra blood required and the request for consent of patients' tissue samples.

Section 6.1.1 – Addition of anti-androgen use for M0 patients as a method of HT

Section 6.1.6 – Addition of the calcium & vitamin name "calcichew".

Section 6.6.2 – asking also to collect vitamins and minerals under concomitant medication.

Section 6.6.3 – New section to inform investigators that patient's, who they wish to give radiotherapy to, are also eligible for STAMPEDE

Section 6.6.4 – New section to detail what data is being collected on the radiotherapy given to patients.

Section 7.1; figure 4 – Addition of radiotherapy form and in note, addition of AA alone

Section 7.1.2 – omission of repeated scans and x-rays at 24 weeks, also omitted in note under figure 4.

Chapter 11 – Safety reporting section updated

Section 17.3 – Increase in amount of blood needed & additional tissue sample request.

21.1.4 Amendments Made To Protocol Version 3.0 (Jul-2006)

Front Cover - NCRN logo added for accuracy

Front Cover - Clarification that protocol developed with NCRI rather than on behalf of

Front Cover - Clarification that it is a 6 arm trial

General Information section - MRC CTU staff section updatedyyyy

Section 1.2 – Statistics section updated.

Section 1.2 - Additional research paragraph updated to reflect additional studies and for clarification of terms

Section 1.2 - Blood collection volume changed to reflect new technique used

Section 1.3 (figure 3) - Table showing case report form schedule updated to reflect clarification of follow-up schedule and addition of new CRF (End of Treatment)

Section 2.2 - AS changed to HT (clarification of terms)

Section 2.3 - Updated in information in regard to use of docetaxel added to reflect up to date practice

Section 2.5 - Sub-headings numbered for consistency

Section 3.0 - Information in regard to the Pilot Phase now written in past tense as Pilot Phase has now been completed

Section 4.1.1 - Inclusion criteria extended so that patients who fulfil 2 out of the three of the first inclusion criteria can be eligible.

Section 4.3.1 - Change in time scales by which baseline investigations need to be completed.

Section 4.3.1 - Clarification that chest X-ray is only required if chest is not included in the CT

Section 4.3.1 - Removal of 12 week timeline for baseline PSA test to be performed. (Stipulation that it must be performed before start of HT)

Section 4.3.2 – Information added in regard to time allowed from randomisation to start of treatment

Section 4.3.3 - Additional research paragraph updated to reflect additional studies and for clarification of terms

Section 4.3.3 - Blood collection volume changed to reflect new technique used

Sections 6.1.2-6.1.6 - Androgen Suppression replaced with hormone therapy for consistency of terms

Section 6.2.2 - '(Taxotere)' Removed for consistency

Section 6.2.2 _ information added in regard to the need to closely monitor liver function prior to docetaxel administration

Section 7.1 - Page number reference updated

Section 7.1.1 - PSA measurement timings updated to accurately reflect follow-up schedule

Section 7.3 (Table 4) - Table and key updated to accurately reflect follow-up schedule and to include information about new CRFs and removal of withdrawal CRF

Section 8 - Rewording for clarification of definition of trial withdrawal

Section 8.1 - Instruction that withdrawal from trial treatment should be recorded on End of Treatment Form rather than withdrawal form

Section 8.1 - Information updated to emphasise that trial treatment must be discontinued following a progression

Section 8.2- Information added in regard to patient transfers

Section 8.3 - Instruction that withdrawal from trial completely must be notified in writing to the MRC CTU rather than included on withdrawal form

Section 9 and Summary – Target event numbers updated to reflect the slightly revised numbers obtained by using –nstage- which is the new, recommended program for MAMS trials

Sections 11.1 and 11.2 - Form numbers removed to allow for future changes in numbering

Section 11.2 – Reference to toxicity grading website added

Section 11.2.1 - Reference to table in appendix G added

Section 12.2 - 'Suggested' removed from 'Suggested patient information sheets'

Section 13 - CTA reference added

Section 17.3 - Information added to reflect new blood collection method for DNA analysis and in regard to additional translational studies for which funding has recently been approved

21.1.5 Amendments Made To Protocol Version 4.0 (Dec-2007)

General Information Section - Randomisation and SAE reporting details sections clarified

Section 1.2 and throughout protocol - Efficacy Stages 1-111 renamed to Activity Stages 1-111 for accuracy and clarity

Section 1.2 - Follow schedule corrected

Section 4.1.2 - Inclusion criteria widened to include high risk relapsing patients, that would not have met the previous PSA based criteria

Section 4.1.3 - Note added to reference location of WHO performance status definitions

Section 4.2 - Notes added to reference locations of toxicity gradings and NYHA classifications

Section 4.3.1 - Timings of baseline scan information changed to accurately reflect most common current practice

Section 6.1.1 - Information about use of LHRH antagonists to ensure that the protocol accurately reflects current and future practice

Section 6.1.1 - Information about suggested duration of hormone therapy added to ensure that the protocol accurately reflects current practice

Section 6.2.2 - Additional information added about the timing of liver function tests prior to docetaxel administration added for clarity

Section 6.6.4 - Information on radiotherapy data collection added

Section 7.1.1 - Erroneous information about the timing of PSA measurements removed

Figure 3 - Moved to new section in protocol for clarity and extended to include current information on data collection

Figure 3b - Added to describe how extent of data collection during follow-up should change, post treatment and post progression

Figure 4 - Notes added to explain the changes in data collected at follow-up and to information that the quality-of-life study will be applicable to the first 700 patients randomised only

Figure 4 - Note added to include palliative radiotherapy CRF

Section 11.3 - SAE reporting information updated

Section 19 - Protocol amendments list updated

21.1.6 Amendments Made To Protocol Version 5.0 (Aug-2008)

1. General Information Section – Randomisation phone line number updated – non UK extension added
2. Section 3 – Information about QL study removed to reflect closure of QL study after first 700 patients
3. Section 4.2 – Exclusion criteria clarified to explain that only patients with severe poor cardiovascular history should be excluded
4. Section 4.3.1 – Information on co-administration of NSAIDS with celecoxib changed based on clinical advice.
5. Section 5 - Randomisation phone line number updated – non UK extension added
6. Section 6.2.1. – Information added to clarify that patients who develop an osteonecrosis of the jaw should stop zoledronic acid treatment
7. Section 6.2.3 – 'severe' text added to accurately reflect which patients should be excluded based on their cardiovascular history
8. Section 7.1.2 – Definition of disease progression extended for clarity
9. Figure 3 – Updated to include reference to newly created skeletal related event form

10. Figure 4 – Previous error in table amended to show that the 4th Zoledronic Acid form that is submitted contains information about 3 cycles rather than 2 as previously indicated

11. Table 4 – 'Other important medical condition' added to definition of serious in the SAE section, to accurately reflect SAE form and current practice

12. Section 11.1 – Information added on reporting or pregnancies

13. Section 17 - Information about QL study removed to reflect closure of QL study after first 700 patients

21.1.7 Amendments Made To Protocol Version 6.0 (Jul-2009)

1. General Information Section – Trial Pharmacist removed and changes of:

Co-Investigator

Patient Representatives

Trial Manager

Data Manager

General Information Section - Coordinating Centre – address change

General Information Section – change of Sponsor address

Section 1.1 – ratio of patients randomised to the investigational arms updated

Section 1.2 – figure 1b added to clarify trial design from Apr-2011 onwards

Section 1.2 – paragraph added to explain trial changes after the second activity analysis

Section 1.2 – wording added to clarify that QL data only collected for first 700 patients randomised

Section 1.3 – SSA Favourable Opinion removed from list of trial documentation required ahead of site accreditation

Section 2.1 – Amount of people diagnosed with prostate cancer annually updated

Section 2.4 –note added to explain completion of recruitment to celecoxib- containing arms

Section 2.5.2 - note added to explain completion of recruitment to celecoxib- containing arms

Section 3 – SSA Favourable Opinion removed

Section 4.2 – Exclusion criterion xiii greyed out

Section 4.3.1 – paragraph removed regarding potential randomisation to celecoxib-containing arms

Section 5 – Randomisation instructions expanded to exclude public holidays or dates when notice has been given by the CTU

Section 6.1.4 – formatting changed to grey font to reflect recruitment completion for arm D

Section 6.1.6 - formatting changed to grey font to reflect recruitment completion for arm F

Section 6.2.3 – recruitment note added

Section 6.6.3 – radiotherapy statement changed to reflect data from recent trials

Section 7.1.2 – removal of reference to SRE- specific CRF

Section 7.3 – Figure 3 - Addition of Bone Density Risk Factor Form and BMD sub-study assessment forms to summary of timing table

Section 7.3 – Figure 4 – Weeks added to timings of assessments post 2 years

Section 7.3- Figure 4 – note added to explain recruitment completion for arms D and F

Section 12.1 – Wording changed to reflect change to randomisation allocation ratio

Section 12.1 – Addition of statement regarding new information emerging during the trial

Section 12.2 – Reference to SSA removed

Section 16.3 – Statement added regarding actioning IDMC recommendation ahead of TSC ratification

21.1.8 Amendments Made To Protocol Version 7.0 (Jul--2011)

1. General Information Section- SAE reporting fax number corrected

2. Section 11- SAE reporting fax number corrected

21.1.9 Amendments Made To Protocol Version 7.1 (Jul-2011)

Throughout protocol – numbering has been updated in some sections new accommodate new information that has been added.

General Information Section – contact details updated

General Information Section – Funding information updated to include involvement from additional company

General Information Section – Wording on compliance and regulations updated to reflect current MRC CTU standard wording

General Information Section – Abbreviations list updated

Section 1.1 – The number of investigational agents being studied updated from three to four

Section 1.1 – Information regarding celecoxib updated to reflect that recruitment to these arms was discontinued in Apr-2011

Section 1.1 – Information about new IMP, Abiraterone inserted

Section 1.1 – Sample size and trial duration information updated to reflect changes brought about by additional trial arm

Section 1.2 – Summary information updated to reflect the discontinuation of recruitment to celecoxib arms and the addition of abiraterone

Figures 1a, b and c - Updated to reflect the discontinuation of recruitment to celecoxib arms and the addition of abiraterone

Section 1.2 – Information on trial stages updated to reflect changes brought about by additional trial arm

Section 1.2 – Information updated regarding the re-opening of the quality-of-life sub-study from implementation of protocol version 8.0

Section 2.1 – Wording related to hormone therapy updated for clarity

Section 2.1 - Updated to reflect the discontinuation of recruitment to celecoxib arms and the addition of abiraterone

Section 2.2 – Updated references added

Section 2.3 – Updated references added

Section 2.5 – Section added to give background information on new IMP, abiraterone

Section 2.6.1 – Updated references added

Section 2.7 – Section added to give information regarding radiotherapy which is to be given as part of standard care following recently published trial data.

Section 3 – Wording updated regarding selection of investigators to reflect current MRC CTU practice

Section 4.1 – Inclusion criteria updated with new criterion regarding radiotherapy use

Section 4.1 - Inclusion criteria updated with new criterion regarding contraceptive use

Section 4.1 – Wording of inclusion and exclusion criteria updated for clarity

Section 4.1 – Exclusion criteria updated with new criterion regarding acceptable liver function for trial entry

Section 4.1 – Exclusion criteria updated with specifics related to blood pressure levels

Section 4.1 - Exclusion criteria updated with new criterion regarding concomitant medications

Section 4.1 - Exclusion criteria updated with new criterion regarding prior treatment with abiraterone

Section 4.1 - Exclusion criteria updated with new criterion regarding prior treatment with chemotherapy

Section 4.1 - Exclusion criteria updated with new criterion regarding prior treatment with zoledronic acid

Section 4.3 – Wording updated to reflect that patients who initially fail screening can be re-screened at a later date

Section 4.3.2 – Wording updated regarding prior anti-androgen and LHRH use updated for clarity

Section 5.1 – Co-enrolment guidelines information updated to describe newly created co-enrolment CRF

Section 6.1 – Trial treatment information updated to reflect the fact that anti-androgens alone will be no longer permitted as hormone therapy

Section 6.1.1 – Updated to describe patients for whom radiotherapy should be given as standard practice

Section 6.1.1 a and b - Sections added to give information regarding radiotherapy treatment

Section 6.1.1-6.1.6 – References to further sections updated

Section 6.1.7 – Section added to describe abiraterone treatment

Section 6.2.4 - Section added to describe abiraterone treatment

Section 6.6 - Section added to give information regarding radiotherapy treatment

Section 7.1.1 – Reference to blood being taken at patient's home removed as this does not occur in practice

Section 7.1.2 – Wording updated regarding the reporting of biochemical failures for clarity

Section 7.1.2 – Wording updated regarding skeletal-related events for clarity

Section 7.1.3 – Section added to describe additional assessments required related to abiraterone treatment

Section 7.1.4 – Section added to provide information on when treatment should commence

Figure 4 – Updated for clarity regarding return of BMD sub-study forms, the addition the co-enrolment CRF and the description of the re-opening of the QL Sub-study.

Figure 5 – Updated with reference to abiraterone and co-enrolment form

Section 7.3 - Wording on trial closure updated to reflect current MRC CTU standard wording

Section 8.1 – Additional criteria for definition of progression added for clarity

Section 8.1 – Definition of progression for abiraterone patients added.

Section 9 – Statistical information updated to describe the addition of the new trial arm

Section 11 – Safety reporting wording updated for clarity

Section 11 – SAE reporting fax number updated

Section 12 – Ethical information updated to describe the unequal randomisation allocation ratio

Section 12 – Ethical information updated to describe that the visit schedule will vary according to trial arm

Section 12.2 – Wording updated to reflect international participation in the trial

Section 13 – Wording updated to reflect international participation in the trial

Section 14 – Wording updated to reflect international participation in the trial

Section 15 - Updated to reflect the discontinuation of recruitment to celecoxib arms and the addition of abiraterone

Section 16 – Reference to trial committee charters added for information

Section 17.1 – Information added to reflect re-opening of quality-of-life sub-study

Section 17.2 – Timing of health economics analysis updated to previous error

Section 18 – Information on publication policy expanded for clarity

Section 19 – Information regarding amendments to protocol appendices moved to the separate appendices document

Section 20 – References extensively updated

21.1.10 Amendments Made To Protocol Version 8.0 (Sep-2011)

Throughout protocol – numbering, sections headings, tables, figures and bibliographical references have been updated in some sections to accommodate new information that has been added

Throughout protocol – Androgen Deprivation Therapy has replaced Hormone Therapy as deemed more representative of the type of hormone therapy used in the study

General Information Section – New staff members of the MRC CTU and Co-Investigators added and contact details updated

General Information Section – Abbreviations list updated

Section 1.1 – Information regarding the new research radiotherapy treatment inserted

Section 1.1 – Information regarding docetaxel updated

Section 1.2 – Wording updated to reflect the addition of the new research comparison arm

Section 1.3 – Additional criteria for the re-accreditation of participating centres (for protocol version 9.0 only)

Section 2.1.1 – Wording updated to clarify the use of anti-androgen in trial patients

Section 2.1.2 – Information added to describe the rationale for the RT comparison arm

Section 2.8 – Information added to describe research RT treatment to prostate for patients with newly diagnosed metastatic disease

Section 3.1 – Information added to describe RT Quality Assurance procedures and centre accreditation

Section 4.1.1 to 4.1.3 – Wording updated to clarify inclusion criteria for all patients groups (newly diagnosed non-metastatic, metastatic and relapsing patients)

Section 4.2 – Clarification added on cardiovascular exclusion criteria

Section 4.2 – New exclusion criterion added concerning patients with prior exposure to hormone therapy

Section 4.2 – New exclusion criterion added to reflect the addition of the new RT comparison arm

Section 4.4.1 – Clarification added regarding pre-randomisation checks

Section 4.4.2 – Clarification added regarding permissible hormone therapy duration prior to randomisation

Section 4.4.5 – Information added regarding starting research radiotherapy treatment

Section 4.4.6 – Information updated on concomitant medications

Section 5 – Clarification regarding randomisation allocation added to reflect the addition of the new RT research arm

Section 6.1.8 – Information added to describe the administration of research radiotherapy

Section 6.2.1 – Clarification added regarding the measurement of serum creatinine levels prior to the administration of zoledronic acid

Section 6.2.3 – Clarification regarding the completion of recruitment to the celecoxib containing arms

Section 6.25 – Information added regarding the administration of research radiotherapy treatment

Section 6.6 – Clarification incorporated to describe the administration of standard-of-care radiotherapy

Section 7.1.4 – Information added regarding data collection and non-administration of standard radiotherapy

Section 7.2 – Section updated to include new treatment specific CRFs and timing of CRFs

Section 8.1 – Clarification added for the criteria to stop treatment for patients randomised to arm G

Section 8.2 – Section expanded to include additional details on study patient transfer to different centres

Section 8.3 – Additional sentence inserted to reinforce the importance of compliance with follow-up assessments

Section 9.1 – Additional paragraph inserted to clarify the method of randomisation and allocation distribution in the light of the introduction of the new RT arm

Section 9.4 – Wording updated to clarify the assessment of safety data

Section 9.5.4 – Wording updated concerning the end of randomisations to arm G

Section 9.6 to 9.6.4 – Section added describing sample size issues and trial stages for arm H

Section 9.8 – Clarification on intermediate stopping guidelines

Section 9.9 – Clarification on the outline analysis plan

Section 11 – Information on safety reporting updated to reflect the addition of the research RT comparison arm

Section 11 – Clarification added regarding arm A safety reporting timelines

Section 12.1 – Clarification added regarding the Principal Investigator's responsibilities

Section 14 – Indemnity section updated to reflect current MRC policy

Section 16 – Clarification regarding TMG membership

Section 17.3 – Section on Bone Mineral Density sub-study removed

Section 19 – Information regarding amendments to protocol appendices moved to the separate appendices document

Section 20 – References updated

21.1.11 Amendments Made To Protocol Version 9.0 (Oct-2012)

Throughout protocol – numbering, sections headings, tables, figures and bibliographical references have been updated in some sections to accommodate the completion of recruitment to original research arms B, C and E.

Throughout protocol – Tenses have been changed to reflect activities that were in the future and which have now been passed.

Section 1 – Figure added and clarifications added to each figure

Section 2 – Previous reference 8 removed

Section 4 – Clarification of acceptable alternatives to bone scans

Section 6.2.5 – Correction of an error defining the PTV: the wording has been reordered

Table 4 – Dose-volume objectives corrected: order swapped

Table 5- Correction CRFs names

Section 17.3.2 – Clarification that DNA may be extracted

21.1.12 Amendments Made To Protocol Version 10.0 (Apr-2013)

Throughout protocol – numbering, sections headings, tables, figures and bibliographical references have been updated in some sections

Throughout protocol – typos have been corrected

Section 4 –Clarification of exclusion criteria V (now V and VI)

Section 6 – Timing of orchidectomy prior to randomisation extended to 12 weeks

Section 6 – Clarification of hypokalaemia, blood pressure and fluid retention management

Section 9 – Statistical considerations amended in light of the recruitment extension for the abiraterone comparison

Section 14 - Section updated to reflect the changes in the structure of the MRC CTU (now MRC CTU at UCL) and indemnity arrangements

21.1.13 Amendments Made To Protocol Version 11.0 (Sep-2013)

Throughout protocol – numbering, sections headings, tables, figures and bibliographical references have been updated in some sections

Throughout protocol – typos have been corrected

Co-investigators list updated to reflect the addition of the "enzalutamide + abiraterone comparison" lead

Section 1.2 – Enzalutamide added as trial treatment

Section 1.2 – Protocol version 12.0 added to the list of amendments

Section 2.10 – Rationale for the combination of enzalutamide and abiraterone

Section 4.2 – Eligibility criteria amended to reflect the addition of enzalutamide + abiraterone arm

Section 4.4.2 – Wording clarified

Section 6.8 – Clarification regarding end of trial treatment after starting trial therapy

Section 6.10 – Section added to describe enzalutamide and abiraterone treatment for the new research arm (Arm J)

Section 6.11.4.A – Section added to describe the management of toxicities from trial abiraterone

Section 6.11.4.B - Section added to describe the management of toxicities from trial enzalutamide

Section 9.1.4 – Section added to describe the statistical considerations concerning the introduction of Arm J

Section 9.3 – Principles and assumption for the introduction of Arm J added

Section 9.7 and sub-sections – Sample size issues and trial stages for Arm J

Section 9.9 – Details on interim monitoring and analyses for Arm J added

Section 11.2.1.D – Wording clarified regarding safety reporting requirements for control arm

Section 12.1 – Wording clarified

Section 15 – Details on funding for the "enzalutamide + abiraterone comparison" added

Section 19 - Amendments made to protocol updated

Reference list updated

21.1.14 Amendments Made To Protocol Version 12.0 (Jan-2014)

Throughout protocol – typos have been corrected

Section 4.4.2. Wording clarified

Section 4.3. Wording clarified for eligibility to M1|RT comparison

Section 6.10. Addition of use of dexamethasone post-biochemical progression for Arm J patients

Section 6.11.4.A. Correction of CTCAE version

Section 6.11.4.C. Clarification on enzalutamide dose modification to be in line with current SmPC

Section 9.6. Sample size increase for M1|RT comparison

Section 11. Correction of safety reporting timelines for Arm A patients

Section 17. Addition of saliva samples collection for DNA analysis

Table 4, 5 and 6. Clarification on Case Report Forms and Follow-up schedule

21.1.15 Amendments Made To Protocol Version 13.0 (Feb-2015)

Throughout protocol – typos have been corrected

Throughout protocol – clarification on the new definition of standard-of-care

Table of contents updated to reflect any changes to the protocol

Section 1.1. Wording added throughout section to include reference to survival results from “original comparisons”

Section 2.1.1. Section improved to include reference to survival results from “original research comparisons”

Section 2.1.2. Section improved to include reference to survival results from “original research comparisons”.

Section 2.1.3. Additional section added to describe the role of docetaxel for people with M0 or M1 disease

Section 2.9. Clarification on treatment completion and primary results for “original research comparisons”

Section 4.2. Clarification of Exclusion criteria XIII and XVI

Section 4.4.2. Clarification on HT prior to randomisation

Section4.4.3. New section to clarify standard-of-care docetaxel treatment prior to randomisation

Section 4.4.7. Clarification on concomitant medication and contra-indicated concomitant medications

Section 4.5. Clarification provided on tissue block collection

Section 6. Inclusion of docetaxel into the standard-of-care

Section 6.2.3 New section to describe standard-of-care docetaxel administration

Section 6.11. Improvement throughout sections and sub-sections for abiraterone and enzalutamide-related toxicity management

Section 6.12. Section improved throughout to incorporate clearer details on concomitant medications and drug-to-drug interactions

Section 7.1.4. New section to describe data collection for standard-of-care docetaxel

Section 9.7.4. Clarification provided about implications for “enzalutamide+ abiraterone comparison” following change of standard-of-care treatment

Section 11.2.1.D Clarification on SAE notification timelines to reflect change in standard-of-care treatments (addition of docetaxel)

Figure 1. Figure updated to reflect change in standard-of-care

Figure 2. Figure updated to reflect trial history and recruitment over time

Figure 3. Figure updated to reflect changes in standard-of-care and recruiting arms

Table 1. Table updated to remove repetition

Table 13. Table updated to include new CRF to report standard-of-care docetaxel treatment

Table 15. Table updated to include only active trial treatments

21.1.16 Amendments Made To Protocol Version 14.0 (Oct-2015)

Throughout protocol – typos have been corrected

Throughout protocol – clarification on the new definition of standard-of-care

Table of contents updated to reflect any changes to the protocol

Section 1. Wording added throughout section to include reference “metformin comparison”

Section 2. Section updated to include reference “metformin comparison”

Section 4.2. Exclusion criteria review to reflect Arm J closure and instruction of “metformin comparison”

Section 4.3. Clarification of comparison specific eligibility (M1|RT and metformin)

Section 4.5.7. Clarification on concomitant medication and contra-indicated concomitant medications

Section 6. Treatment sections improved throughout

Section 6.11. Section updated to include details on metformin treatment

Section 6.12. Amendment throughout sections and sub-sections for metformin treatment

Section 6.13. Amendment throughout sections and sub-sections for metformin treatment

Section 6.13. Improvement throughout sections and sub-sections for abiraterone and enzalutamide treatment

Section 7.0. Amendment throughout sections and sub-sections to include assessment and procedures specific to “metformin comparison”

Section 9.0. Section updated and streamlined to capture statistical considerations on each comparison

Section 9.0. Details on “metformin comparison” added

Section 11. Safety processes updated and clarified

Section 16.0 Membership to oversight groups updated

Section 11.2.1.D Clarification on SAE notification timelines to reflect change in standard-of-care treatments (addition of docetaxel)

21.1.17 Amendments Made To Protocol Version 15.0 (Mar-2017)

Throughout protocol – re-structure of the treatment-related information for ease of use

Throughout protocol – clarification on the definition of standard-of-care

Throughout protocol – typos have been corrected

Addition of TMG members

Table of contents updated to reflect any changes to the protocol

New section for summary of trial added in table format

Section 1. Revised format for the summary of treatment groups, with the new transdermal oestradiol arm also added

Section 2. Clarification regarding research treatments that have previously reported or completed recruitment, section updated to include the "transdermal oestradiol comparison"

Section 3. New sections added for the "transdermal oestradiol comparison" and future planned biomarker-selected comparisons

Section 4.1.4. Change in definition of adequate renal function

Section 4.3. New section added for the biomarker screening pilot, selection criteria removed for "research RT comparison"

Section 4.4.1. Change in definition of adequate renal function

Section 4.4.2. New section added for the patient selection criteria specific to the "transdermal oestradiol comparison"

Section 4.5. Screening procedure tables and figure added for clarification.

Section 4.5.1. New section added for biomarker screening pilot investigations prior to randomisation.

Section 5.1.1. New section added for the biomarker screening pilot registration.

Section 6. New sections added for the "transdermal oestradiol comparison"

Section 7. Amendment throughout sections and sub-sections to include assessment and procedures specific to "transdermal oestradiol comparison"

Section 7.1.4.B. Section added on cardiovascular outcomes for the "transdermal oestradiol comparison"

Table 18. Table added to clarify follow-up assessments

Section 8. Section updated for "transdermal oestradiol comparison"

Section 9. Section updated for "transdermal oestradiol comparison"

Section 12.1.1.D. Section added on participant feedback from investigations and additional analyses

Section 15. Section updated for "transdermal oestradiol comparison" and biomarker screening pilot

21.1.18 Amendments Made To Protocol Version 16.0 (Oct-2017)

Summary of trial- Table 1: Schedule of Assessments has been added

Abbreviations & Glossary- new terms have been added

Section 1- Table 4: Abiraterone information updated as results of primary analysis published

Section 4.3 - Biomarker timelines redefined, the length of prior hormone therapy has increased to reflect change in turnaround time for testing

Section 4.6 - Biomarker screening information updated

Section 6.2 – Clarification on safety monitoring required for patients receiving trial abiraterone added . Abiraterone overdose information altered for clarity.

Section 6.3.4 - Drug interactions updated to specify that tamoxifen is contraindicated in combination with abiraterone, enzalutamide and transdermal oestradiol.

Section 6.5 – Detail on requirements at site to demonstrate compliance with per-protocol required safety monitoring added

Section 7 – Schedule for assessments updated, removal of table 19

Section 7.1 - Clarification on additional safety monitoring required for patients receiving trial abiraterone added

Section 7.4 - Table 20 QL information removed and added to Table 1: Schedule for Assessments

Section 10.1.1- Central monitoring of consent information added

Section 11 – Re-structured and re-worded for clarity on reporting requirements for safety data captured on the SAE CRF. Explanation provided for exempted events and definitions added. Table 28 and Box 1 updated and Figure 1 added.

Section 11.2- Updated SAE exceptions, SAE flow chart added for clarity

Section 11.3 - Update of investigator assessments and notification checklist for expedited safety reporting

Section 11.4 - Update of wording of CTU responsibilities

Section 17.4 - Sub-study information added to include Disease Volumetric sub-study

21.1.19 Amendments Made To Protocol Version 17.0 (Feb-2017)

Throughout protocol - Typos have been corrected, abbreviations & glossary & table of contents updated

Throughout protocol - Addition of abiraterone as SOC & original comparisons closed to active follow-up

Throughout protocol – Update of and removal of biomarker pilot information now randomisation to the rucaparib comparison is to be activated

Trial administration – Information updated, full contact list linked to website, all comparison chief-investigators added as co-signatories

Summary of trial – Updated, "rucaparib comparison" added and "original comparisons" closed to active follow-up; figure 1 updated with new randomisation schema and S-STAMPEDE Cohort study

Schedule of Assessments updated– Table 1a removed, Tables 1, 2 and 3 added

Lay Summary – Re-drafted, "rucaparib comparison" added

Section 2 – Role of SOC abiraterone added, reported comparisons updated and rationale for comparisons that have completed recruitment removed. Rational for the "rucaparib comparison" added

Section 3.1 – Addition of site and investigator criteria

Section 4 – Complete restructuring of section, addition of biomarker screening and registration information.

Section 4.2 – Additional information about proposed approach to staged informed consent

Section 4.4 – Clarification as to required pre-randomisation screening by comparison

Section 4.5.4 – Detail regarding SOC abiraterone (permitted in metformin comparison only)

Section 4.9.3 – Eligibility to be randomised to the "rucaparib comparison" added

Section 4.10 - Sub-study eligibility criteria clarified; new germline blood sub-study added (PAXgene for S1A and S1M) and stratified – STAMPEDE cohort study added

Section 5 – additional information relating to registration and randomisation to the "rucaparib comparison" added

Section 6.1.4- SOC abiraterone detail added

Section 6.2.7.C – Table 19: Additional assessments required following change of transdermal oestradiol patch or dose added

Section 6.2.9 - Rucaparib treatment specific information added

6.3 – Concomitant medications updated: clarification that spironalactone is contraindicated with abiraterone and rucaparib drug interactions added

Section 7.1 – Table 27: summary of follow-up schedules by participant group added

Section 7.1.5.D – Additional Safety assessment required for participants receiving rucaparib added

Section 7.2.3 – Data collection for SOC abiraterone clarified

Section 7.2.8 & 7.3.2 - Data collection & Follow-up procedures for S-STAMPEDE Cohort participants described

Section 7.3.3 – Clarification added regarding procedures to use linked follow-up information obtained from sources of electronic health data

Table 29 and 28 updated with new CRFs

Section 8.1.4 – Reasons to stop rucaparib

Section 9.8 – addition of statisitcal considerations relating to the "rucaparib comparison"

Section 11 – Stopping of SARs and SUSARs reporting for "original comparisons" closed to active follow up and addition of rucaparib-specific notable events

Section 12- ethical considerations updated with detail relating to data to permit linkage with sources of electronic health data

Section 13 – Data archiving and retention guidance added

Section 16 – Updates of STAMPEDE oversight committees including expanded TMG sub-groups

Section 17.1.1 A- Closure of HE & QL sub-studies to new participants and stopping of data collection for several comparisons; summarised in Table 40.

Section 17.2 – S-STAMPEDE cohort study & additional germline data collection added

Section 17.4 – New sub-study: Using routine data to identify clinical trial outcomes added

Section 18 – New section regarding patient and public involvement in STAMPEDE added

Section 20 - New section regarding data and sample sharing added

21.1.20 Amendments made to Protocol Version 18.0 (Jun-2018)

Throughout protocol: Removal of "rucaparib comparison" information

Throughout protocol: Redraft of biomarker screening pilot study into ancillary studies section

Summary of Trial – Figure 1 updated; removal of registration information for S-STAMPEDE Cohort study

Schedule of Assessments – Removal of registration from figure 1; Table 2 updated to include PSA within 8 weeks of randomisation; Table 3 S-STAMPEDE Schedule of Assessments removed

Abbreviations & Glossary - Terms relating to the "rucaparib comparison" have been deleted

Section 1 – Lay summary "rucaparib comparison" information removed

Section 2 – Rationale for incorporating molecular stratificaion and "rucaparib comparison" removed

Section 3.2 – "Rucaparib comparison" comparison-specific site accreditation removed

Section 4 – Removal of biomarker screening and registration information

Section 4.2 – Removal of staged informed consent process

Section 4.3 – Removal of biomarker screening eligibility information

Section 4.4.3 – Removal of "rucaparib comparison" screening investigations prior to randomisation

Section 4.5 – Removal of prior permitted SOC treatments for "rucaparib comparison"

Section 4.7 – Removal of and clarification to the general inclusion & exclusion criteria of Serum Pottasium & Cardiovascular disease respectively.

Section 4.9.3 – Removal of "rucaparib comparison" specific eligibility criteria

Section 4.10 – Addition of information regarding biomarker pilot screening

Section 4.10.1 – Removal of S-STAMPEDE Cohort sub-study

Section 4.10.2 – Removal of PAXgene sample collection

Section 5 – Removal of information relating to registration and randomisation to the "rucaparib comparison"

Section 6.2.6.B – Addition of additional metformin dose reduction stages

Section 6.2.9 – Removal of "rucaparib comparison" research treatment information

Section 6.3.1 – Removal of "rucaparib comparison" therapeutic interactions information

Section 7 - Table 30 removal of "rucaparib comparison" specific CRFs; table 31 – removal of arm S1M schedule for completion of treatment forms

Section 7.1.5.D – Removal of rucaparib additional safety assessments

Section 7.2.8 – Removal of data collection for S-STAMPEDE Cohort participants

Section 7.3.2 – Removal of follow-up for S-STAMPEDE cohort participants

Section 8.1.1 – Clarification of metformin, abiraterone and enzalutamide use post progression

Section 8.1.4 – Removal of "rucaparib comparison" stopping trial treatment information

Section 9.6.7 – Revised sample size for "metformin comparison"

Section 9.8 – Removal of statistical consideration relating to the "rucaparib comparisons"

Section 10.1.1 – Updated central monitoring of consent process

Section 11 – Removal of rucaparib-sepcific notable events

Section 11.1.1 – Clarification of trial-specific exemptions and notable adverse events

Section 16 – Addition of Genetic Sub-Group

Section 17.1.1.A – Clarification of Qulaity of Life and Health Economics data collection

Section 17.2 – Details of biomarker screening pilot study information moved here

Section 17.2.1 – removal of S-STAMPEDE Cohort study information

21.1.21 Amendments made to Protocol Version 19.0 (Aug-2020)

Throughout protocol: Section headings and table numbers renumbered, references updated, Centre changed to Site for consistency

General information - Sponsor updated to UCL, Trial contacts updated, Glossary updated

Summary of Trial – M1RT status updated, transdermal oestradiol number of participants updated

Figure 1 updated to include new SOC options and clarify options based on HbA1c level.

Figure 2 updated to reflect extended recruitment in metformin and te2 comparisons

Schedule of Assessments –remove height and upadte footnotes

Abbreviations – minor updates

Section 1 – updated comparisons closed to recruitment – addition of Arm H. Arms E and F added to Table 6.

Section 2.1.3 – Addition of new SOC options abiraterone, enzalutamide and apalutamide

Section 2.3 – Addition of M1RT results

Section 2.5.2.B – Updated target recruitment for “transdermal oestradiol” comparison

Section 3.1.2 – Addition to recommend additional investigators be delegated for safety reporting to cover absences

Section 4.2 – Minor rewording clarification

Section 4.1 – Update to screening investigations, addition of timeframes in days, M1 imaging clarified, several baseline investigations moved to pre-randomisation. ECG removed, option for no fasting glucose added.

Section 4.3 – Table 8 - Addition of new SOC options abiraterone, enzalutamide and apalutamide

Section 4.3.2 – Added oligometastatic disease

Section 4.3.3 –Clarification SOC docetaxel cannot be given if SOC abiraterone, enzalutamide and apalutamide is planned

Section 4.3.4 – New section for SOC abiraterone, enzalutamide and apalutamide. Addition of enhanced monitoring for “transdermal oestradiol” comparison

Section 4.4.4 – General inclusion criteria III removed, haematological value thresholds clarified

Section 4.5 – General exclusion criteria II added (consolidates previous VII-IX), clarification where both AST and ALT results required, VI clarified exclusion is for unhealed surgical wounds rather than surgerical intervention

Section 4.6.1 – Added metabolic substudy to “metformin“ comparison requirements

Section 4.6.2 – Added eligibility criteria for participants not yet started on SOC abiraterone, enzalutamide and apalutamide

Section 4.7.2 – Clarification circulating tumour DNA sub study not recruiting

Section 4.7.4 – New section for Eligibility for metformin sub study

Section 5.1 – Addition of new instruction to provide randomisation documents to CTU after randomisation.

Section 5.2 – Clarification of wording for co-enrolment in other trials (interventional and non-interventional)

Section 6 – Reformatted throughout, new subheadings and layout, multiple new tables inserted

Section 6.1 –Addition of SOC combination table to replace text list

Section 6.1.1 – Section renamed Androgen Deprivation Therapy (previously Hormone Therapy)

Section 6.1.2 – Updated wording on Radiotherapy for M0 participants

Section 6.1.2.C – New section for oligometastatic participants

Section 6.1.3 – Administration of SOC RT moved up from 6.7

Section 6.1.5 – New title SOC upfront systemic therapy – addition of SOC abiraterone, enzalutamide and apalutamide

Section 6.2 –Research treatment broken down per IMP instead of per comparison.

Section 6.2.1 – Addition of Table 9 treatment duration for all research treatments and Table 10 management of trial treatment post progression

Section 6.2.2 – Some text now in tables. Updated wording about associated toxicities and contraindications

Section 6.2.3 – Some text now in tables. Updated wording about associated toxicities and contraindications

Section 6.2.3.C – New table for hypertension monitoring.

Section 6.2.4 - Some text now in tables. Updated wording about associated toxicities and contraindications

Section 6.2.5 - Reformatted, some text now in tables. Updated wording about associated toxicities and contraindications. Additional detail on moving from induction to maintenance dose on oestradiol level, and changing brands of patches

Section 6.3 – Clarification of wording. Details of drug interactions and additional safety monitoring moved up to sections 6.2.2, 6.2.3, 6.2.4 and 6.2.5 respectively

Section 7 – Added Figure 3 - PSA progression example scenarios

Section 7.1.4 – Clarification added to explain rationale for continuing to collect metabolic test results post progression

Section 7.1.3.A –Addition of Figure 3 example progression schenarios

Section 7.1.4 – Addition of rationale for continuing metabolic tests beyond progression

Section 7.1.5.A Clarification of required tests for abiraterone and enzalutamide, specifically when one or the other is discontinued.

Section 7.1.5.C - Addition of real time monitoring of hormone results for te2 participants to check safety of combination with new SOC abiraterone, enzalutamide and apalutamide

Section 7.2.3 – Addition of SOC systemic therapy log

Section 7.2.4 – Addition of requirement to submit radiotherapy CRF even if radiotherapy not given

Section 7.3 – Nurse led follow up expanded to allow for other appropriately qualified individuals

Section 7.3.1 – Minor changes

Section 7.4.1 – Table 28 updated with new CRFs for SOC systemic therapy, and metabolic sub study CRF. Removal of baseline form, bone density risk factor. Blood form moved. Updated key for Table 29.

Section 8.1 – Clarification of consent for data collection

Section 8.1.3 – Additional wording on stopping te2 and options for pausing, switching and restarting.

Section 8.2 – New section about permitted breaks in SOC ADT treatment

Section 9.2 – Additional wording regarding analysis of “enzalutamide + abiraterone comparison”

Section 9.5 – Additional wording regarding analysis of “enzalutamide + abiraterone comparison”

Section 9.7.4 – New section detailing enhanced safety monitoring of combination new SOC and transdermal oestradiol

Section 9.10 – removal of Mann-Whitney test as relevant for original comparisons now closed.

Section 9.10.2 - Additional wording regarding analysis of “enzalutamide + abiraterone comparison”

Section 10.1 – Minor clarification to wording

Section 10.1.2 – Clarification of central monitoring processes

Section 10.1.3 – Wording updated in line with current protocol template

Section 11.1.1 – Clarification of notable events (NEs) to be collected, new cancers no longer included.

Section 11.1.2 – Addition of new SOC enzalutamide and apalutamide

Section 11.2 – Update to add requirement for investigator absence cover, updated AE, SAE and NE notification period. Figure 5 added.

Section 11.2.2 – Updated expedited reporting exemptions

Section 11.2.3.C – Expectedness removed from site investigator responsibilities. Table 37 updated

Section 11.2.3.D – Figure 6 updated. Box 1 updated

Section 11.3 – Updated process for causality queries. Clarification of Swiss reporting responsibilities

Section 11.3.1 – Expectedness added to sponsor responsibilities

Section 12.1.1.A – Updated with new SOC enzalutamide and apalutamide

Section 13 – CTA number updated

Section 15 - Clarification for SOC enza and addition of SOC apalutamide

Section 16.2 - Clarification on frequency of TSC meetings

Section 16.4 – subgroups updated

Section 16.5 – Figure 7 updated

Section 17.1 – Addition that secure email now permitted for sending CRFs

Section 17.2.2 – Minor changes, sub study is now closed

Section 17.2.4 – Removed details of biomarker sub study as now closed

Section 17.5 – New section added for metformin metabolic sub study

Section 20 – Clarification that any data released must be approved by TMG, TSC and subject to agreements

Section 22 – References updated, removal of several references linked to protocol v18 which was not released to sites

21.1.22 Amendments made to Protocol Version 20.0 (Oct-2020)

Minor amendment to correct typograhical, spelling, formatting and cross-reference error, or clarify wording throughout.

Minor updates to Summary of Trial table

Table 2 – ECG removed from Cardiac asseessment row, weight added to waist measurement row

4.2.2 – Timeframe for pre - SOC docetaxel bloods updated from 4 months to 16 weeks in line with other timeframes.

4.4 Header title changed

4.6 Footnote 1 restored

5.2 Wording revised to allow participants to continue on STAMPEDE research treatment while co-enrolled in IMP trial, providing no interactions.

6.1.2 Header title changed

6.1.3.A Header title changed

6.2 Footnote 2 removed from Table 10

6.2.3.C – Restoration of Table 11, deleted in error when accepting tracked changes. Table 11 outlines 3 monthly blood pressure monitoring as specified in section 7.1.5.B

6.3 Header title changed

11.2.2 – Correction to bullet list exemption, text removed

22 REFERENCES

1. James ND, Sydes MR, Clarke NW, Mason MD, Dearnaley DP, Spears MR, et al. Addition of docetaxel, zoledronic acid, or both to first-line long-term hormone therapy in prostate cancer (STAMPEDE): survival results from an adaptive, multiarm, multistage, platform randomised controlled trial. Lancet. 2016;387(10024):1163-77.
2. Mason MD, Clarke NW, James ND, Dearnaley DP, Spears MR, W.S.R. A, et al. Adding celecoxib with or without zoledronic acid for hormone-naive prostate cancer: long-term survival results from an adaptive, multi-arm, multi-stage, platform, randomised controlled trial. 2017. In press. DOI: 10.1200/JCO.2016.69.0677.
3. James ND, de Bono JS, Spears MR, Clarke NW, Mason MD, Dearnaley DP, et al. Abiraterone for Prostate Cancer Not Previously Treated with Hormone Therapy. N Engl J Med. 2017;377(4):338-51.
4. Parker CC, James ND, Brawley CD, Clarke NW, Hoyle AP, Ali A, et al. Radiotherapy to the primary tumour for newly diagnosed, metastatic prostate cancer (STAMPEDE): a randomised controlled phase 3 trial. Lancet. 2018;392(10162):2353-66.
5. cancerresearchuk. Prostate cancer statistics
2015 [Available from: http://www.cancerresearchuk.org/health-professional/cancer-statistics/statistics-by-cancer-type/prostate-cancer.
6. Sharifi N, Gulley JL, Dahut WL, Sharifi N, Gulley JL, Dahut WL. An update on androgen deprivation therapy for prostate cancer. Endocrine-Related Cancer. 2010;17(4):R305-15.
7. Widmark A, Klepp O, Solberg A, Damber JE, Angelsen A, Fransson P, et al. Endocrine treatment, with or without radiotherapy, in locally advanced prostate cancer (SPCG-7/SFUO-3): an open randomised phase III trial. Lancet. 2009;373(9660):301-8.
8. Warde PR, Mason MD, Sydes MR, Gospodarowicz MK, Swanson GP, Kirkbride P, et al. Intergroup randomized phase III study of androgen deprivation therapy (ADT) plus radiation therapy (RT) in locally advanced prostate cancer (CaP) (NCIC-CTG, SWOG, MRC-UK, INT: T94-0110; NCT00002633). J Clin Oncol. 2010;28(18s Supplement: Proceedings of ASCO 2010):Abstr CRA4504.
9. Warde P, Mason M, Ding K, Kirkbride P, Brundage M, Cowan R, et al. Survival Benefit with Combined Androgen Deprivation and Radiation Therapy in Locally Advanced Prostate Cancer – Results of a Phase III Trial. The Lancet. 2011 - in press.
10. Mason M, Sydes M, Parulekar W, Parmar M, Anderson J, Barber J, et al. Final analysis of intergroup randomized phase III study of androgen deprivation therapy (ADT) + radiation therapy (RT) in locally advanced prostate cancer (CaP) (NCIC-CTG, SWOG, MRC-UK, INT: T94-0110). National Cancer Research Institute (NCRI) Cancer Conference 2012. 2012;2012.
11. Warde P, Mason M, Ding K, Kirkbride P, Brundage M, Cowan R, et al. Combined androgen deprivation therapy and radiation therapy for locally advanced prostate cancer: a randomised, phase 3 trial. The Lancet. 2011;378:2104-11.
12. Nicholas D James MRS, Noel W Clarke, David P Dearnaley, Malcolm D Mason, Christopher C Parker, Alastair W S Ritchie, J. Martin Russell, Francesca Schiavone, Gerhardt Attard, Johann S de Bono, Alison Birtle,Daniel S Engeler,Tony Elliott, David Matheson, Joe O'Sullivan, Delia Pudney, Narayanan Srihari, Jan Wallace, Jim Barber, Isabel Syndikus, Mahesh K B Parmar, Matthew R Sydes. Failure-free survival and the impact of radiotherapy in patients with newly diagnosed non metastatic prostate cancer: Data from patients in the control arm of the STAMPEDE trial (MRC PR08, CRUK/06/019). JAMA Oncology. 2015.
13. Sweeney CJ, Chen YH, Carducci M, Liu G, Jarrard DF, Eisenberger M, et al. Chemohormonal Therapy in Metastatic Hormone-Sensitive Prostate Cancer. N Engl J Med. 2015;373(8):737-46.
14. Vale CL, Burdett S, Rydzewska LH, Albiges L, Clarke NW, Fisher D, et al. Addition of docetaxel or bisphosphonates to standard of care in men with localised or metastatic, hormone-sensitive

prostate cancer: a systematic review and meta-analyses of aggregate data. Lancet Oncol. 2016;17(2):243-56.
15. James ND, Ingleby FC, Clarke NW, Amos C, Attard G, Cross W, et al. Docetaxel for hormone-naive prostate cancer (PCa): Results from long-term follow-up of non-metastatic (M0) patients in the STAMPEDE randomised trial. Annals of Oncology [Internet]. 2019 Oct; 30. Available from: https://doi.org/10.1093/annonc/mdz248.008a.
16. Fizazi K, Tran N, Fein L, Matsubara N, Rodriguez-Antolin A, Alekseev BY, et al. Abiraterone plus Prednisone in Metastatic, Castration-Sensitive Prostate Cancer. N Engl J Med. 2017;377(4):352-60.
17. Hoyle AP, Ali A, James ND, Cook A, Parker CC, de Bono JS, et al. Abiraterone in "High-" and "Low-risk" Metastatic Hormone-sensitive Prostate Cancer. Eur Urol. 2019;76(6):719-28.
18. Davis ID, Martin AJ, Stockler MR, Begbie S, Chi KN, Chowdhury S, et al. Enzalutamide with Standard First-Line Therapy in Metastatic Prostate Cancer. N Engl J Med. 2019;381(2):121-31.
19. Chi KN, Agarwal N, Bjartell A, Chung BH, Pereira de Santana Gomes AJ, Given R, et al. Apalutamide for Metastatic, Castration-Sensitive Prostate Cancer. N Engl J Med. 2019;381(1):13-24.
20. James ND, Sydes MR, Clarke NW, Mason MD, Dearnaley DP, Spears MR, et al. Addition of docetaxel, zoledronic acid, or both to first-line long-term hormone therapy in prostate cancer (STAMPEDE): survival results from an adaptive, multiarm, multistage, platform randomised controlled trial. Lancet. 2015.
21. Smith MR, Finkelstein JS, McGovern FJ, Zietman AL, Fallon MA, Schoenfeld DA, et al. Changes in body composition during androgen deprivation therapy for prostate cancer. J Clin Endocrinol Metab. 2002;87(2):599-603.
22. Eriksson A, Attvall S, Bonnier M, Eriksson JW, Rosander B, Karlsson FA. Short-term effects of metformin in type 2 diabetes. Diabetes Obes Metab. 2007;9(4):483-9.
23. Bailey CJ. Treating insulin resistance in type 2 diabetes with metformin and thiazolidinediones. Diabetes Obes Metab. 2005;7(6):675-91.
24. Ohira M, Miyashita Y, Ebisuno M, Saiki A, Endo K, Koide N, et al. Effect of metformin on serum lipoprotein lipase mass levels and LDL particle size in type 2 diabetes mellitus patients. Diabetes Res Clin Pract. 2007;78(1):34-41.
25. Wulffele MG, Kooy A, de Zeeuw D, Stehouwer CD, Gansevoort RT. The effect of metformin on blood pressure, plasma cholesterol and triglycerides in type 2 diabetes mellitus: a systematic review. J Intern Med. 2004;256(1):1-14.
26. Lubik AA, Gunter JH, Hendy SC, Locke JA, Adomat HH, Thompson V, et al. Insulin increases de novo steroidogenesis in prostate cancer cells. Cancer Res. 2011;71(17):5754-64.
27. Venkateswaran V, Haddad AQ, Fleshner NE, Fan R, Sugar LM, Nam R, et al. Association of diet-induced hyperinsulinemia with accelerated growth of prostate cancer (LNCaP) xenografts. J Natl Cancer Inst. 2007;99(23):1793-800.
28. Noto H, Goto A, Tsujimoto T, Noda M. Cancer risk in diabetic patients treated with metformin: a systematic review and meta-analysis. PLoS One. 2012;7(3):e33411.
29. Rothermundt C, Hayoz S, Templeton AJ, Winterhalder R, Strebel RT, Bartschi D, et al. Metformin in chemotherapy-naive castration-resistant prostate cancer: a multicenter phase 2 trial (SAKK 08/09). Eur Urol. 2014;66(3):468-74.
30. Krupski TL, Smith MR, Lee WC, Pashos CL, Brandman J, Wang Q, et al. Natural history of bone complications in men with prostate carcinoma initiating androgen deprivation therapy. Cancer. 2004;101(3):541-9.
31. Langley RE, Cafferty FH, Alhasso AA, Rosen SD, Sundaram SK, Freeman SC, et al. Cardiovascular outcomes in patients with locally advanced and metastatic prostate cancer treated with luteinising-hormone-releasing-hormone agonists or transdermal oestrogen: the randomised, phase 2 MRC PATCH trial (PR09). Lancet Oncol. 2013;14(4):306-16.
32. Byar DP. Proceedings: The Veterans Administration Cooperative Urological Research Group's studies of cancer of the prostate. Cancer. 1973;32(5):1126-30.

33. Hedlund PO, Henriksson P. Parenteral estrogen versus total androgen ablation in the treatment of advanced prostate carcinoma: effects on overall survival and cardiovascular mortality. The Scandinavian Prostatic Cancer Group (SPCG)-5 Trial Study. Urology. 2000;55(3):328-33.
34. Gilbert DC, Duong T, Kynaston HG, Alhasso AA, Cafferty FH, Rosen SD, et al. Quality-of-life outcomes from the Prostate Adenocarcinoma: TransCutaneous Hormones (PATCH) trial evaluating luteinising hormone-releasing hormone agonists versus transdermal oestradiol for androgen suppression in advanced prostate cancer. BJU Int. 2016.
35. Smith DC, Redman BG, Flaherty LE, Li L, Strawderman M, Pienta KJ. A phase II trial of oral diethylstilbesterol as a second-line hormonal agent in advanced prostate cancer. Urology. 1998;52(2):257-60.
36. Hsueh AJ, Peck EJ, Jr., clark JH. Progesterone antagonism of the oestrogen receptor and oestrogen-induced uterine growth. Nature. 1975;254(5498):337-9.
37. Hsueh AJW, Peck Jr EJ, Clark JH. Progesterone antagonism of the oestrogen receptor and oestrogen-induced uterine growth. Nature. 1975;254:337.
38. Janssen. L. ZYTIGA® (abiraterone acetate) Investigator Brochure. 13 ed2017.
39. BNF. Abiraterone interactions [Available from: https://www.medicinescomplete.com/#/content/bnf/_293081460_interactions.
40. Woo HH, Begbie S, Gogna K, Mainwaring PN, Murphy DG, Parnis F, et al. Multidisciplinary consensus: a practical guide for the integration of abiraterone into clinical practice. Asia Pac J Clin Oncol. 2014;10(3):228-36.
41. Zentiva. Tamsulosin 400 microgram prolonged-release hard capsules, Summary of Product Characteristics [Available from: https://www.medicines.org.uk/emc/product/9245/smpc.
42. Tucci M, Roca E, Ferrari L, Pia A, Dalla Volta A, Bedussi F, et al. Abiraterone and prednisone therapy may cause severe hypoglycemia when administered to prostate cancer patients with type 2 diabetes receiving glucose-lowering agents. Endocrine. 2019;64(3):724-6.
43. Astellas. Xtandi®(enzalutamide, MDV3100) Investigator Brochure. 11 ed2019.
44. Lipska KJ, Bailey CJ, Inzucchi SE. Use of metformin in the setting of mild-to-moderate renal insufficiency. Diabetes Care. 2011;34(6):1431-7.
45. Merck. Glucophage 500 mg film coated tablets Summary of Product Characteristics [Available from: https://www.medicines.org.uk/emc/product/987/smpc.
46. Salpeter SR, Greyber E, Pasternak GA, Salpeter EE. Risk of fatal and nonfatal lactic acidosis with metformin use in type 2 diabetes mellitus: systematic review and meta-analysis. Arch Intern Med. 2003;163(21):2594-602.
47. Viani GA, Bernardes da Silva LG, Stefano EJ. Prevention of gynecomastia and breast pain caused by androgen deprivation therapy in prostate cancer: tamoxifen or radiotherapy? International journal of radiation oncology, biology, physics. 2012;83(4):e519-24.
48. Hussain M, Tangen CM, Berry DL, Higano CS, Crawford ED, Liu G, et al. Intermittent versus continuous androgen deprivation in prostate cancer. N Engl J Med. 2013;368(14):1314-25.
49. Royston P, Parmar MKB, Qian W. Novel designs for multi-arm clinical trials with survival outcomes with an application in ovarian cancer. Statistics in Medicine. 2003;22(14):2239-56.
50. Royston P, Barthel FMS, Parmar MKB, Choodari-Oskooei B, Isham V. Designs for clinical trials with time-to-event outcomes based on stopping guidelines for lack of benefit. Trials. 2011;12(1):81.
51. Royston P. nstage: MAMS trial sample size calculator. MRC Clinical Trials Unit, London2009.
52. Mason MD, Parulekar WR, Sydes MR, Brundage M, Kirkbride P, Gospodarowicz M, et al. Final Report of the Intergroup Randomized Study of Combined Androgen-Deprivation Therapy Plus Radiotherapy Versus Androgen-Deprivation Therapy Alone in Locally Advanced Prostate Cancer. J Clin Oncol. 2015;33(19):2143-50.
53. James ND, Sydes MR, Mason MD, Clarke NW, Anderson J, Dearnaley DP, et al. Celecoxib plus hormone therapy versus hormone therapy alone for hormone-sensitive prostate cancer: first results from the STAMPEDE multiarm, multistage, randomised controlled trial. The Lancet Oncology. 2012;13(5):549-58.

54. Haybittle JL. Repeated assessment of results in clinical trials of cancer treatment. Br J Radiol. 1971;44(526):793-7.
55. Peto R, Pike MC, Armitage P, Breslow NE, Cox DR, Howard SV, et al. Design and analysis of randomized clinical trials requiring prolonged observation of each patient. I. Introduction and design. Br J Cancer. 1976;34(6):585-612.

STAMPEDE

APPENDICES

**Version:**	**19.0**
**Date:**	**18 August 2020**

**MRC CTU AT UCL ID:**	**PR08**
**ISRCTN #:**	**ISRCTN78818544**
**NCT #:**	**NCT00268476**
**EUDRACT#**	**2004-000193-31**
**CTA #:**	**20363/0404/001**
**MREC #:**	**04/MRE07/35**

CONTENTS

CONTENTS 1

APPENDIX A: ADDITIONAL DEFINITIONS 2

APPENDIX B: REFERENCE SAFETY INFORMATION FOR PROTOCOL TREATMENT 4

APPENDIX C: EVALUATION OF BASELINE AND NEW LESIONS 5
C.1 MEASURABLE DISEASE 5
C.2 BASELINE DOCUMENTATION OF "TARGET" AND NON-TARGET LESIONS 5
C.3 DEFINITION OF PROGRESSION (PATIENTS WITH MEASURABLE DISEASE AT RANDOMISATION) 5
C.4 DEFINITION OF PROGRESSION (FOR PATIENTS WITHOUT MEASURABLE DISEASE AT RANDOMISATION) 6

APPENDIX D: QUALITY OF LIFE AND HEALTH ECONOMICS 7
D.1 OVERVIEW 7
D.2 ESTIMATING COSTS 7
D.2.1 Hospital resource use 7
D.2.2 NHS non-hospital resource use 7
D.3. MEASURING EFFECTS 7
D.4. ANALYSIS 8

APPENDIX E: ALLOCATION RATIO BY PROTOCOL VERSION 9

APPENDIX F: EXTRA INFO FOR TRANSDERMAL OESTRADIOL COMPARISON 11
F.1 RESULTS FROM THE PATCH TRIAL TO DATE 11
F.2 CARDIOVASCULAR SAFETY MONITORING WITHIN THE PATCH TRIAL FOR TRANSDERMAL OESTRADIOL USED IN COMBINATION WITH DOCETAXEL 12

APPENDIX G: AMENDMENTS MADE TO APPENDICES 13
G.1 AMENDMENTS MADE TO APPENDICES VERSION 1.0 MAY-2004 13
G.2 AMENDMENTS MADE TO APPENDICES VERSION 1.1 MAY-2005 13
G.3 AMENDMENTS MADE TO APPENDICES VERSION 2.0 JUN-2005 13
G.4 AMENDMENTS MADE TO APPENDICES VERSION 3.0 JUL-2006 14
G.5 AMENDMENTS MADE TO APPENDICES VERSION 4.0 DEC-2007 15
G.6 AMENDMENTS MADE TO APPENDICES VERSION 5.0 AUG-2008 15
G.7 AMENDMENTS MADE TO APPENDICES VERSION 6.0 JUL-2009 16
G.8 AMENDMENTS MADE TO APPENDICES VERSION 7.0 JUN-2011 16
G.9 AMENDMENTS MADE TO APPENDICES VERSION 8.0 SEP-2011 16
G.10 AMENDMENTS MADE TO APPENDICES VERSION 9.0 OCT 2011 17
G.11 AMENDMENTS MADE TO APPENDICES VERSION 10.0 APR-2013 17
G.12 AMENDMENTS MADE TO APPENDICES VERSION 11.0 JAN-2014 17
G.13 AMENDMENTS MADE TO APPENDICES VERSION 12.0 FEB-2015 17
G.14 AMENDMENTS MADE TO APPENDICES VERSION 13.0 OCT-2015 18
G.15 AMENDMENTS MADE TO APPENDICES VERSION 14.0 MAR-2016 18
G.16 AMENDMENTS MADE TO APPENDICES VERSION 15.0 MAR-2017 19
G.17 AMENDMENTS MADE TO APPENDICES VERSION 16.0 OCT-2017 19
G.18 AMENDMENTS MADE TO APPENDICES VERSION 17.0 FEB-2018 19
G.19 AMENDMENTS MADE TO APPENDICES VERSION 18.0 JUN-2018 19
G.20 AMENDMENTS MADE TO APPENDICES VERSION 19.0 AUG-2020 20

APPENDIX A: ADDITIONAL DEFINITIONS

Table 1: WHO Performance Status

GRADE	PERFORMANCE STATUS
**0**	Able to carry out all normal activity without restriction
**1**	Restricted in physically strenuous activity but ambulatory and able to carry out light work
**2**	Ambulatory and capable of all self-care but unable to carry out any work; up and about more than 50% of waking hours
**3**	Capable of only limited self-care; confined to bed or chair more than 50% of waking hours
**4**	Completely disabled; cannot carry on any self-care; totally confined to bed or chair

Table 2: TNM Classification: Primary Tumour

CATEGORY	DESCRIPTION
**Tx**	Primary tumour cannot be assessed
**T0**	No evidence of primary tumour
**T1**	Clinically unapparent tumour not palpable or visible by imaging<br>▪ T1a: Tumour incidental, found in ≤5% resected tissue<br>▪ T1b: Tumour incidental, found in > 5% resected tissue<br>▪ T1c: Tumour identified by needle biopsy found in one or both sides (because of high PSA)
**T2**	Tumour is palpable but remains confined within the prostate gland<br>▪ T2a: Tumour involves ≤½ of one side<br>▪ T2b: Tumour involves >½ of one side<br>▪ T2c: Tumour involves both sides
**T3**	Tumour extends through the prostatic capsule (but not fixed and does not invade adjacent structures apart from seminal vesicles)<br>▪ T3a: Extracapsular extensions (unilateral or bilateral)<br>▪ T3b: Tumour invades seminal vesicles
**T4**	Tumour is fixed or invades adjacent structures other than seminal vesicles: bladder neck, external sphincter, rectum, levator muscles and/or pelvic wall

Table 3: TNM Classification: Regional Lymph Nodes

CATEGORY	DESCRIPTION
**Nx**	Regional lymph nodes have not been assessed
**N0**	No regional lymph node metastasis
**N1**	Regional lymph node metastasis

Table 4: TNM Classification: Distant Metastasis

STAGE	DESCRIPTION
**Mx**	Distant metastasis has not been assessed
**M0**	No distant metastasis
**M1**	Distant metastasis<br>• M1a: Non-regional lymph nodes only<br>• M1b: Bone<br>• M1c: Other sites (with or without bone disease)

Reference

AJCC Cancer Staging Manual. 8th ed. New York

Table 5: NYHA classification of heart failure

CLASS	DESCRIPTION
**Class I: asymptomatic**	No limitation in physical activity despite presence of heart disease. This can be suspected only if there is a history of heart disease which is confirmed by investigations - for example, echocardiography
**Class II: mild**	Slight limitation in physical activity. More strenuous activity causes shortness of breath - for example, walking on steep inclines and several flights of steps. Patients in this group can continue to have an almost normal lifestyle and employment
**Class III: moderate**	More marked limitation of activity which interferes with work. Walking on the flat produces symptoms
**Class IV: severe**	Unable to carry out any physical activity without symptoms. Patients are breathless at rest and mostly housebound

APPENDIX B: REFERENCE SAFETY INFORMATION FOR PROTOCOL TREATMENT

Reference Safety Information (RSI) are documents containing a list of expected terms for Investigational Medicinal Products (IMPs) to be used in the assessment of expectedness for Serious Adverse Reactions (SARs).

It is the Sponsor's responsibility to identify appropriate RSI and obtain the necessary regulatory approvals required. The Sponsor also will perform the assessment expectedness from the release of Protocol version 20.

However a list of RSI, their documents and the periods for which they relevant are available via the STAMPEDE website: http://www.stampedetrial.org/centres/essential-documents/reference-safety-information-rsi/

Figure 1: Expected side effects for Radiotherapy

Body system	Side-effect
Blood and bone marrow	Myelosuppression
Gastrointestinal disorders	Diarrhoea (including increase in stool frequency, loose stool), constipation, proctitis, proctalgia, rectal ulcer, rectal bleeding, rectal urgency, fistula, bowel obstruction, bowel perforation, abdominal pain, nausea, vomiting *(rare)*
General disorders	Fatigue/tiredness, anorexia
Infections	Urinary tract infection
Musculoskeletal and connective tissue disorders	Bone fractures
Neoplasms	Bladder cancer *(rare)*, bowel cancer *(rare)*
Renal and urinary disorders	Nocturia, hesitancy, urinary urgency, urinary frequency, urinary incontinence, urethral obstruction or stricture (causing poor urethral stream or urinary retention), dysuria, haematuria, bladder spasms
Reproductive system disorders	Erectile dysfunction, decreased libido, decreased volume of ejaculate/absence of ejaculate, infertility, prostate spasms or pain
Skin and subcutaneous tissue disorders	Pubic hair loss, skin irritation, skin redness
Surgical and medical procedures	Need for urinary catheter

APPENDIX C: EVALUATION OF BASELINE AND NEW LESIONS

These guidelines are based on the RECIST criteria (1) but have been modified to include progression based on PSA measurements.

C.1 MEASURABLE DISEASE

Measurable disease is defined as at least one lesion that can be accurately measured in at least one dimension (longest dimension to be recorded). Each lesion must >20mm when measured by conventional techniques, including palpation, CT, and MRI, or >10mm when measured by spiral CT.

C.2 BASELINE DOCUMENTATION OF "TARGET" AND NON-TARGET LESIONS

All measurable lesions up to a maximum of 5 lesions per organ and 10 lesions in total representative of all involved organs should be identified as target lesions and will be recorded and measured at baseline. Target lesions should be selected on the basis of their size (lesions with the longest dimension) and their suitability for accurate repetitive measurements by one consistent method of assessment (either by imaging techniques or clinically). A sum of the longest dimension (LD) for all target lesions will be calculated and reported as the baseline sum LD. The baseline sum LD will be used as reference to further characterise the objective tumour.

All other lesions (or sites of disease) should be identified as non-target lesions and should also be recorded at baseline. Measurements are not required.

All baseline evaluations of disease status should be performed as close as possible to the start of the treatment and not more than 4 weeks before the beginning of the treatment.

C.3 DEFINITION OF PROGRESSION (PATIENTS WITH MEASURABLE DISEASE AT RANDOMISATION)

Progression is defined as **any** of the following:

- At least a 20% increase in the sum of LD target lesions taking as reference the smallest sum LD recorded since study entry
- The appearance of one or more new lesions
- Death due to disease without prior objective documentation of progression
- Global deterioration in health status attributable to the disease requiring a change in therapy without objective evidence of progression
- Unequivocal progression of non-target lesions, (other than pleural effusions without cytological proof of neoplastic origin) in the opinion of the treating physician (in this case an explanation must be provided).
- Progression will be most usually based on PSA measurements (see **Section 7.1.3**) but tumour measurements should take precedence over PSA response. If measurable disease is shrinking during treatment, but the PSA is rising the patient should continue to receive protocol treatment.

Note that for the "transdermal oestradiol comparison", death from causes other than prostate cancer is also a criterion for progression (rationale given in **Section 9.8.3**), though this does not affect the normal data collection within STAMPEDE.

C.4 DEFINITION OF PROGRESSION (FOR PATIENTS WITHOUT MEASURABLE DISEASE AT RANDOMISATION)

Progression (for patients with non-measurable disease at randomisation) is defined as increasing clinical or radiological evidence of disease since study entry. Progression can also be based on PSA measurements (**Appendix E**).

Reference

1. P Therasse, SG Arbuck, EA Eisenhauer et al. New Guidelines to Evaluate the Response to Treatment in Solid Tumors. J Natl Cancer Inst (2000) 92 (3): 205-216.

APPENDIX D: QUALITY OF LIFE AND HEALTH ECONOMICS

D.1 OVERVIEW

The economic evaluation will take the form of a cost-effectiveness analysis in which the differential cost of the alternative treatments will be related to their differential benefits in terms of quality-adjusted life years (QALYs). Incremental analysis will be undertaken and cost-effectiveness acceptability curves will be used to show the probability of one option being more cost-effective than the others.

D.2 ESTIMATING COSTS

A cost analysis will be undertaken from the perspective of the National Health Service. Resource use measurement during the trial will be divided into that relating to the hospital and NHS non-hospital. These are dealt with in turn below.

D.2.1 Hospital resource use

Within the trial, hospital resource use data will be collected on all patients entering the trial. Specifically, this will include in-patient nights in hospital, distinguishing intensive care from stay on a general ward. These will be collected using case record forms completed by clinical review at the follow-up points shown in the main document. Some visits to, and stays in, hospital may relate to non-study hospitals. To ensure that data on this resource use are captured, a questionnaire will be administered to patients as part of the quality of life assessments.

These resources will be valued in monetary terms using unit costs representative of UK practice at the time of analysis. For drugs, this will be based on British National Formulary prices. For hospital procedure and hotel costs, unit costs will, if available, be based on NHS Reference Costs. Otherwise, they will be estimated from a sample of UK centres randomising patients into the trial.

D.2.2 NHS non-hospital resource use

Data on patients' use of community-based NHS (and complementary health) services will be collected from patients as part of the quality of life assessments. The resources will include visits to and from a GP or district nurse. Costing of community-based resources will be based on published unit costs. Other services will be costed using data available at the point of analysis.

D.3. MEASURING EFFECTS

This clinical trial is estimating a range of clinical and health-related quality of life effects within trial patients. The purpose of an economic evaluation will be to set these in context of the resource costs incurred in achieving them. A cost-effectiveness analysis will relate differential cost to an aggregated measure of effect in the form of a QALY. QALYs will be based on observed mortality and patients' responses to the EQ-5D questionnaire. The latter asks patients to categorise their health, with 3 levels of response (no problems, moderate problems, severe problems) on 5 dimensions (mobility, self care, usual activities, pain/discomfort, depression anxiety). Each of the 245 possible health states has been 'valued' on a zero to one 'utility' based on the preferences of 3,395 members of the UK public.

D.4. ANALYSIS

All resource use data will be valued in monetary terms, as described above, such that each patient has an associated cost over their period of follow-up. A full stochastic analysis will be undertaken to allow for sample variation in resource use and effect data. Methods are developing quickly in this area and, by the time of the analysis, 'best practice' may have altered markedly from today. If such an analysis were to be undertaken now, the general methods would be as follows.

A QALY profile will be estimated for each patient based on their survival duration weighted by their responses to the EQ-5D Health Related QL questionnaire, which generates a single index value for health at each point of follow-up. The profiles will assume a straight-line relationship between the index value at time t and the value at time t+1. The number of QALYs they experience during the period of follow-up in the trial will be the area under the QALY profile.

In the primary analysis, only data collected in the trial will be used in the analysis; in other words, the estimate of QALYs for each group is likely to reflect the fact that some patients are still alive after the follow-up (i.e. the survival curve is truncated and survival analysis techniques will be used to estimate QALYs).

As a secondary analysis, extrapolation techniques will be used to estimate the final portion of the survival curve so as to provide a full estimate of differential life expectancy. A number of extrapolation techniques will be used to provide a range of estimates of differential QALYs over a lifetime time horizon.

Cost-effectiveness acceptability curves will be used to facilitate a measure of uncertainty around cost-effectiveness estimates. These curves show the probability of one form of management being more cost-effective than the others assuming alternative levels of the maximum amount decision-makers are willing to pay for an extra QALY.

Sensitivity analysis will be used to consider the importance of sources of uncertainty other than sample variation (e.g. unit costs, discount rates, method of extrapolation). Multiple regression techniques will be employed to provide as precise a measure of cost-effectiveness as possible and to undertake sub-group analysis using baseline patient characteristics, which will be defined in advance in the analysis plan.

A full health economics analysis plan (HEAP) will be developed for each comparison.

Reference

Dolan, P, Gudex, C, kind, P, and et al. A Social Tariff for EuroQol: Results from a UK General Population Survey. Centre for Health Economics Discussion Paper 138. Centre for Health Economics, University of York: CHE. 1995

APPENDIX E: ALLOCATION RATIO BY PROTOCOL VERSION

The following table shows the allocation ratio to each arm by the protocol version open at the time. The multi-arm, multi-stage platform design means that allocation to arms has changed over time. The allocation ratio within each comparison (e.g. A vs B) has, of course, remained constant.

Allocation ratios represent the probability of allocation at each time point for a patient who is eligible for all currently-open trial arms. This has varied over the duration of the trial. For example, for protocol version 10 the allocation ratio to arms A, G and H for a patient with M1 disease at baseline was 2:2:2; however, for an M0 patient this was 2:2:0 due to them not being eligible for entry to the "M1|RT comparison".

Table 6: History of allocation at randomisation by protocol version

Protocol Version	Activation Date	Protocol Change	A	B	C	D	E	F	G	H*	J	K†	L	S1A	S1M
V1	n/a	Not used	2	1	1	1	1	1	-	-	-	-	-	-	-
V2	Jun-05	Activated	2	1	1	1	1	1	-	-	-	-	-	-	-
V3	Jul-06		2	1	1	1	1	1	-	-	-	-	-	-	-
V4	Dec-07		2	1	1	1	1	1	-	-	-	-	-	-	-
V5	Aug-08		2	1	1	1	1	1	-	-	-	-	-	-	-
V6	Jul-09		2	1	1	1	1	1	-	-	-	-	-	-	-
V7	Jun-11	DF stopped	2	1	1	0	1	0	-	-	-	-	-	-	-
V8	Sep-11	G added	2	1	1	0	1	0	2	-	-	-	-	-	-
V9	Oct-12	H added	2	1	1	0	1	0	2	2	-	-	-	-	-
V10	Apr-13	BCE stopped	2	0	0	0	0	0	2	2	-	-	-	-	-
V11	Sep-13	G stopped	2	0	0	0	0	0	0	2	-	-	-	-	-
V12	Jan-14	J added	2	0	0	0	0	0	0	2	2	-	-	-	-
V13	Feb-15		2	0	0	0	0	0	0	2	2	-	-	-	-
V14	Oct-15	SOC change	2	0	0	0	0	0	0	2	2	-	-	-	-
V15	Mar-16	K added	2	0	0	0	0	0	0	0	0	2	-	-	-
V16	Mar-17	L added	2	0	0	0	0	0	0	0	0	2	2	-	-
V17	Oct-17		2	0	0	0	0	0	0	0	0	2	2	-	-
V18	n/a¥	S1 added	2	0	0	0	0	0	0	0	0	2	2	X	X
V19	Jul-18	S1 removed	2	0	0	0	0	0	0	0	0	2	2	-	-
V20	Aug-20	SOC change	2	0	0	0	0	0	0	0	0	2	2	-	-

1 or 2 Allocation weight while open to recruitment
\- Before allocation started (future accrual)
x Not applicable as an allocation – with respect to stratified arms S1A and S1M, eligible patients would have been allocated 1:1 between these arms

* Introduction of the "M1|RT comparison" meant allocation had to be split by baseline metastatic status, as the research question is not relevant to men without newly-diagnosed metastases or with a contraindication to RT.
† Introduction of the "metformin comparison" meant allocation had to be split, as the research question is not suitable for patients with diabetes.
¥ Protocol version 18 received MHRA, REC and HRA approval but was not activated at sites

APPENDIX F: EXTRA INFO FOR TRANSDERMAL OESTRADIOL COMPARISON

F.1 RESULTS FROM THE PATCH TRIAL TO DATE

There have been some encouraging results from the randomised PATCH trial comparing transdermal oestradiol (tE2) versus LHRH agonists (n=875 patients recruited up to 6-10-2015).

- Transdermal oestradiol achieves similar castration rates as LHRH (1).
- Risk of cardiovascular morbidity and mortality is similar in the two arms, suggesting transdermal oestradiol avoids the cardiovascular risk seen with oral oestrogen (2).
- The bone health sub-study showed patients on transdermal oestradiol avoid the loss in bone mineral density associated with LHRH (3).
- Patients treated with transdermal oestradiol have a number of improved quality of life outcomes at 6 months compared to those on LHRH, particularly less fatigue and improved physical functioning (4).
- Transdermal oestradiol results in more favourable metabolic profiles than LHRH (2).
- In a pre-planned, confidential interim analysis in Jun-2013 (n=638), transdermal oestradiol met the pre-specified criteria for non-inferiority compared to LHRH based on progression-free survival (leading to the phase III extension of the study).

References:

[1] Langley RE, Godsland IF, Kynaston H, et al. Early hormonal data from a multicentre phase II trial using transdermal oestrogen patches as first-line hormonal therapy in patients with locally advanced or metastatic prostate cancer. *BJU international* 2008; **102**(4): 442-5.

[2] Langley RE, Cafferty FH, Alhasso AA, et al. Cardiovascular outcomes in patients with locally advanced and metastatic prostate cancer treated with luteinising-hormone-releasing-hormone agonists or transdermal oestrogen: the randomised, phase 2 MRC PATCH trial (PR09). *The Lancet Oncology* 2013; **14**(4): 306-16.

[3] Langley RE, Kynaston HG, Alhasso AA, et al. A Randomised Comparison Evaluating Changes in Bone Mineral Density in Advanced Prostate Cancer: Luteinising Hormone-releasing Hormone Agonists Versus Transdermal Oestradiol. *European urology* 2016; **69**(6): 1016-25.

[4] Gilbert DC, Duong T, Kynaston HG, et al. Quality-of-life outcomes from the Prostate Adenocarcinoma: TransCutaneous Hormones (PATCH) trial evaluating luteinising hormone-releasing hormone agonists versus transdermal oestradiol for androgen suppression in advanced prostate cancer. *BJU Int.* 2017 May; **119**(5):667-675

F.2 CARDIOVASCULAR SAFETY MONITORING WITHIN THE PATCH TRIAL FOR TRANSDERMAL OESTRADIOL USED IN COMBINATION WITH DOCETAXEL

An increased risk of venous thromboembolism has been observed when docetaxel is used in combination with certain agents for the treatment of prostate cancer. However, within the PATCH trial so far (based on data up to 17-Sep-2017), no cardiovascular endpoint events have been reported among participants on transdermal oestradiol receiving upfront docetaxel.

The rate of cardiovascular (CVS) events is being closely monitored among patients in the transdermal oestradiol arm who are receiving docetaxel as part of their first-line treatment within the PATCH trial, and this will similarly be done for Arm L patients in STAMPEDE.

An early review by the IDMC would be triggered if there is strong evidence that the proportion of patients experiencing a CVS event by 1 year is 8% or higher; this corresponds to the lower limit of the 95% confidence interval being ≥8%.

A review will therefore be triggered by the following boundaries to be applied to the accumulating data:

- ≥5 of the first 20 patients experiencing a CVS event within the first year of follow-up
- ≥8 of 45 patients
- ≥11 of 70 patients
- ≥15 of 100 patients

In addition, the rate of CVS events based on all follow-up data from patients on transdermal oestradiol with docetaxel (not just within the first year) will be assessed.

APPENDIX G AMENDMENTS MADE TO APPENDICES

G.1 AMENDMENTS MADE TO APPENDICES VERSION 1.0 MAY-2004

Appendix A – Addition of NYHA classifications

Appendix B – General PIS - Docetaxel information updated, additional information explaining cardiovascular risk related to celecoxib and celecoxib duration amended

Appendix B – PIS C – Docetaxel information updated

Appendix B – PIS D – Additional information explaining cardiovascular risk related to celecoxib and celecoxib duration amended

Appendix B – PIS E – Docetaxel information updated

Appendix B – PIS F – Additional information explaining cardiovascular risk related to celecoxib and celecoxib duration amended

Appendix C – GP letter – product name changed from Celebrex to Onsenal

Appendix E – Drug supply/ordering procedures amended

Appendix F – 'Administration of Zoledronic acid' – updated dose modification section

Appendix F – Additional section added 'Co-administration of docetaxel + zoledronic acid'

Appendix G – Celecoxib – updated from revised celecoxib SPC

Appendix G – Table G.2 - Comparative table of undesirable effects of docetaxel, Zoledronic Acid and Celecoxib – updated information from revised celecoxib SPC

Appendix J – Common Toxicity Criteria – additional clarification and change in table structure

Appendix M – Accreditation documents – addition of new documents

Appendix P – Assessing and notifying CTU of adverse events flow diagram added

G.2 AMENDMENTS MADE TO APPENDICES VERSION 1.1 MAY-2005

Appendix F – 'Administration of Zoledronic acid' – updated section on recommend dose reduction in patients with mild to moderate kidney dysfunction (defined as a creatinine clearance of 30-60ml/min) who are receiving zoledronic acid.

Appendix G – Drug Safety Information for Drugs used in the Trial; section on zoledronic acid. Additional text informing clinicians that rare cases of Osteocronosis (primarily of the jaws) have been reported in patients treated with bisphosphonates.

G.3 AMENDMENTS MADE TO APPENDICES VERSION 2.0 JUN-2005

Appendix B – Patient information sheets & consent form – Increase in amount of blood needed & additional tissue sample request.

Appendix B – General PIS – Addition of the use of bicalutamide alone as a Hormone treatment.

Appendix B – General PIS – paragraph detailing to patients that they may receive radiotherapy along side their allocated treatment in STAMPEDE.

Appendix B – General PIS – Side effects of osteoporosis added to "What are the side effects"

Appendix B – General PIS – Will my taking part be kept confidential – now asking for NHS number and mention of NHS Strategic Tracing Service.

Appendix B – STAMPEDE Additional Research – Increase in amount of blood needed & addition tissue sample request.

Appendix B – Consent form – additional consent asked for urine and tumour samples, name to be stored on computer database and an extra copy of consent form to be taken and stored at the MRC CTU.

PIS A, B, C, D, E & F – addition of information about side effects of osteoporosis

PIS A, B, C, D, E & F – Addition of bicalutamide alone as an option for hormone therapy for M0 patients

PIS B, E & F – New side effects added, kidney failure and Osteoncrosis of the jaw

Appendix E– drug ordering - number of bottles of celecoxib sent to pharmacies changed from 15 to 24

Addition of Appendix P – Detailing radiotherapy guidelines for investigators.

G.4 AMENDMENTS MADE TO APPENDICES VERSION 3.0 JUL-2006

Appendix B - Patient Information Sheets (PIS) & Consent Forms - all translational additional now research refered to as 'translational sub-studies'

Appendix B – Information added that there will be different additional research PIS and consent forms for centres taking part in the bone mineral density study (BMD) compared with those that are not

Appendix B - All PIS - 'family doctor' replaced by GP to ensure consistency of terms

Appendix B - General PIS section 12 – Information about collection of patient names re-worded for clarity

Appendix B Additional Research PIS – Type A (for centres NOT participating in the BMD study) and Type B (for centres that are participating in the BMD study) created

Appendix B -Additional Research PIS section 2 - Information on when to complete questionnaires updated to accurately reflect follow-up schedule

Appendix B - Additional Research PIS - Information added to reflect new blood collection method for DNA analysis and in regard to additional translational studies for which funding has recently been approved

Appendix B - Consent Form - Type A (for centres NOT participating in the BMD study) and Type B (for centres that are participating in the BMD study) created

Appendix B Consent form - Information changed in section L in regard to volume of blood sample required to reflect new collection practice

Appendix B -Consent Form – Information about allowing consent form to be sent to MRC CTU and GP, moved to the optional section

Appendix B -All Arm Specific PIS – Wording changed in HT section to more clearly explain how different treatments may affect bone in different ways.

Appendix C -GP letter - 'study' replaced by 'trial' and 'androgen suppression' replaced by 'hormone therapy' to ensure consistency of terms

Appendix C – GP letter – Specification on number of calcichew tablets removed to allow for variations in local practice

Appendix D - DNA analysis sub-study sample collection information changed to reflect new technique (FTA elute cards) being used for blood collection

Appendix E – Specificity on the contents of the drug starter pack removed to allow for changes in the future

Appendix F - Clarification that all docetaxel treatment delays should be reported on the CRF.

Appendix F – Information added in regard to the need to closely monitor liver function prior to administration of docetaxel

Appendix L - MRC CTU staff updated and IDMC members updated

Appendix M - Investigator Statement updated to reflect current version

Appendix P - 'AS' replaced with 'HT' to ensure consistency of terms

Appendix P - Description of hormone therapy clarified

Appendix P – Recommended dose of radiotherapy amended to better reflect current practice

Appendix Q – New appendix added to give information in regard to BMD study.

G.5 AMENDMENTS MADE TO APPENDICES VERSION 4.0 DEC-2007

Appendices spilt for main protocol for ease of use

Appendix B - General PIS - Information about osteonecrosis of the jaw risk added to zoledronic acid section

Appendix B - All arm specific PIS - Information about LHRH antagonists added to the hormone therapy section

Appendix K - Instructions in regard to confirming biochemical failure amended

Appendix L - Trial Steering Committee contact details updated

G.6 AMENDMENTS MADE TO APPENDICES VERSION 5.0 AUG-2008

Appendix B – Guidance for administrators – QL Information removed

Appendix B – General PIS – QL information removed

Appendix B – Additional Research PIS (type A) – QL Information removed

Appendix B – Additional Research PIS (type B) – QL Information removed

Appendix B – Consent form (type A) – QL Information removed

Appendix B – Consent form (type B) – QL Information removed

Appendix F - Information added to clarify that patients who develop an osteonecrosis of the jaw should stop zoledronic acid treatment

Appendix L – TMG Contact details updated

G.7 AMENDMENTS MADE TO APPENDICES VERSION 6.0 JUL-2009

Appendix B – patient information sheets for arms D and F greyed out

Appendix B – General PIS updated to v7.0

Appendix C - GP letter updated to v7.0

Appendix E – reference to SSA favourable assessment removed

Appendix L – trial contacts updated

Appendix P – radiotherapy guidelines updated

G.8 AMENDMENTS MADE TO APPENDICES VERSION 7.0 JUN-2011

Appendix B – General Patient Information Sheet – Updated to reflect addition of new trial arm

Appendix B – General Patient Information Sheet – Updated with text to better describe randomisation as per current MRC CTU standard practice

Appendix B – Arm specific information patient information sheet for arm G added

Appendix B – Wording in all patient information sheets regarding hormone therapy updated for clarity and to reflect that anti-androgen monotherapy is no longer permitted

Appendix B - All patient information sheets updated with information regarding who reviews the trial, as per current MRC CTU standard practice

Appendix B – Wording in all patient information sheets updated for clarity as per current MRC CTU standard practice

Appendix C – GP Letter - Updated to reflect addition of new trial arm

Appendix E – Drug Supply Information - Updated to reflect addition of new trial arm

Appendix G – Drug Safety Information – Updated to include information on abiraterone

Appendix F – Zoledronic Acid dose reduction information clarified

Appendix H – Previous appendix H removed to reflect that the treatment at home with zoledronic acid is not an option in the trial

Appendix L – Reference to CTCAE toxicity grading website updated

Appendix M – Contact details updated

Appendix O – Previous appendix O containing a diagram relating to flow of safety data has been removed as this has been deemed not useful for centres. Subsequent appendices renamed accordingly.

Appendix P - – Previous appendix P regarding radiotherapy removed and added to the main protocol

Appendix O – Added to summarise changes to appendices in each version

G.9 AMENDMENTS MADE TO APPENDICES VERSION 8.0 SEP-2011

Throughout appendices – numbering and sections headers have been updated in some sections to accommodate new information that has been added

Appendix A – Wording updated

Appendix B – Patient Information Sheets removed and submitted as separate documents

Appendix C – GP letter removed and submitted as separate document

Appendix F.4 – Information added on administration of RT

Appendix G.3 – Details on Celecoxib removed

Appendix G.4 Information on abiraterone updated to reflect changes in the Investigator Brochure

Appendix G.3 – Information on undesirable effects for abiraterone updated to reflect changes in the Investigator Brochure

Appendix G.5 – Section on RT contraindications, special warnings and precautions added

Appendix K – Section on RT Quality Assurance added

Appendix L – Information on hormone therapy prior to randomisation added

Appendix M – Trial Management Group contact details updated

Appendix N – Institution statement, signature and delegation of responsibilities log and site personnel list removed

Appendix P – Section on Bone Mineral Density sub-study removed

Appendix Q – Appendices updated

G.10 AMENDMENTS MADE TO APPENDICES VERSION 9.0 OCT 2011

Appendix J – Typo corrected in section J2

Appendix M - Trial Management Group contact details updated

G.11 AMENDMENTS MADE TO APPENDICES VERSION 10.0 APR-2013

Appendix G.4 – Section updated to reflect the new abiraterone IB

Appendix G.5 –Section added to include the combination of enzalutamide and abiraterone

Appendix M - Trial Management Group, Trial Steering Committee, Independent Data Monitoring Committee and trial team contact details updated

Appendix Q - Appendices updated

G.12 AMENDMENTS MADE TO APPENDICES VERSION 11.0 JAN-2014

Typos corrected throughout the Appendices

Appendix D. Addition of saliva sample collection

Appendix G.4.3. Update of abiraterone interactions with other medicinal products

Appendix L. Clarification on use of MAB for Arm J patients

G.13 AMENDMENTS MADE TO APPENDICES VERSION 12.0 FEB-2015

Typos corrected throughout the Appendices

Appendix F. Details on primary analysis for zoledronic acid and docetaxel containing arms included

Appendix G. Details on trial treatments removed and incorporated into main Protocol

Appendix J. Wording improved to clarify definition of biochemical progression

Appendix L. Clarification on use of MAB for Arm J patients

Appendix M. Details on TMG, TSC and IDMC members updated

G.14 AMENDMENTS MADE TO APPENDICES VERSION 13.0 OCT-2015

Typos corrected throughout the Appendices

Appendix G. Table of undesirable effects updated to reflect inclusion of metformin

Appendix I. Table remove and reference to CTCAE updated

Appendix M. Details on TMG, TSC and IDMC members updated

Appendix Q. Table included to describe history of allocation at randomisation

G.15 AMENDMENTS MADE TO APPENDICES VERSION 14.0 MAR-2016

Typos corrected throughout

Appendices re-labeled following removal of outdated information or information that is now replicated in STAMPEDE protocol version 16.0

Appendix B. PATIENT INFORMATION SHEETS AND CONSENT FORM removed as information contained within STAMPEDE protocol version 16.0

Appendix C. GP LETTER removed as information contained within STAMPEDE protocol version 16.0

Appendix D. DNA ANALYSIS SUB-STUDIES removed as information contained within STAMPEDE Sample collection and handling manual version 1.0

Appendix E. DRUG SUPPLY INFORMATION renamed Appendix B

Appendix F. Removed as docetaxel and zoledronic acid no longer being administered as a trial treatment

Appendix G. Renamed Appendix C. Table of undesirable effects updated to reflect inclusion of transdermal oestradiol

Appendix H. EVALUATION OF BASELINE AND NEW LESIONS renamed Appendix D

Appendix I. COMMON TOXICITY CRITERIA removed as information contained within STAMPEDE protocol version 16.0

Appendix J. (Renamed Appendix E) updated for clarity and inclusion of transdermal oestradiol

Appendix K. RADIOTHERAPY QUALITY ASSURANCE removed as no longer relevant

Appendix L. HORMONE THERAPY PRIOR TO RANDOMISATION removed as updated information now contained within STAMPEDE protocol version 16.0

Appendix M. (Renamed Appendix F) TMG and MRC CTU at UCL members updated

Appendix N. PARTICIPATING SITE ACCREDITATION FORM removed as information contained within STAMPEDE protocol version 16.0

Appendix O. Renamed Appendix G

Appendix P. BONE MINERAL DENSITY SUB STUDY removed as no longer relevant

Appendix Q. (Renamed Appendix H) allocation ratio of randomisation updated to include the transdermal oestradiol arm

Appendix I. EXTRA INFORMATION FOR TRANSDERMAL OESTRADIOL COMPARISON added

Appendix S. Renamed Appendix J

G.16 AMENDMENTS MADE TO APPENDICES VERSION 15.0 MAR-2017

Appendix C. Renamed to "DRUG SAFETY INFORMATION FOR ALL PROTOCOL TREATMENT". Recognised undesirable effects tables updated for all remaining drugs.

Appendix F. Updated contact details for staff members

G.17 AMENDMENTS MADE TO APPENDICES VERSION 16.0 OCT-2017

Appendix C. Deletion and insertion of new tables relating to undesirable effects for all treatments.

Appendix F. Updated contact details for staff members

G.18 AMENDMENTS MADE TO APPENDICES VERSION 17.0 FEB-2018

Appendix C2. Updating of enzalutamide information to fall in line with updated IB.

Appendix C3. Addition of rucaparib medicinal product interaction.

Appendix C4. Removal of Docetaxel and zoledronic acid from table 6.

Appendix C4. Removal of table of undesirable effects for celecoxib, zoledronic acid and addition of Rucaparib.

Appendix F. Updated contact details for staff members.

Appendix I. Updated to included allocation ratio for version 18 of the protocol

G.19 AMENDMENTS MADE TO APPENDICES VERSION 18.0 JUN-2018

Appendix C3. Removal of rucaparib medicinal product interaction

Appendix C5. Removal of summary tables 10-13 of recognised undesirable effects of protocol treatment

Appendix C6. Removal of summary tables 14-18 of recognised undesirable effects of permitted standard-of-care treatments.

Appendix H. Removal of "rucaparib comparison" allocation ratio

G.20 AMENDMENTS MADE TO APPENDICES VERSION 19.0 AUG-2020

Appendix A. TNM staging edition updated

Appendix B . Removed as this duplicated information contained in the SPC and Investigator Brochures freely available to participating sites.

Appendix C (now B). Majority detail removed. Updated to briefly refer to the location of external RSI document provided to sites. Expected adverse events for radiotherapy added, these were present in earlier versions of the protocol but were removed in error

Appendix D (now C)

Appendix E. Removed as duplicated information provided in protocol

Appendix F. Removed as contact details are provided on trial website

Appendix G (now D)

Appendix H (now E). Table 6 updated

Appendix I (now F). Diagrams removed and references inserted. I3 types of patches recommended for the trial removed as information is in protocol

Appendix J (now G)

STAMPEDE OVERSIGHT COMMITTEES, STAFF AND COLLABORATORS

Version: 15-Sep-2021

CONTENTS

NOTES ... 2
TRIAL MANAGEMENT GROUP ... 3
INDEPENDENT DATA MONITORING COMMITTEE ... 4
TRIAL STEERING COMMITTEE ... 5
MRC CLINICAL TRIALS UNIT AT UCL STAFF ... 6
SWISS GROUP FOR CANCER CLINICAL RESARCH (SAKK) STAFF ... 10
BIOLOGY AND IMAGING SUBGROUPS ... 11
INVESTIGATORS AND COLLABORATORS: SITE STAFF ... 12
INDUSTRY COLLABORATORS ... 151
PARTICIPANTS ... 152

NOTES

Investigators and site staff are those who have formally appeared at any time on a site's Delegation Logs.

CTU staff are those who have worked on or contributed to the trial any time from the outset until the date of this report.

The independent members of the Independent Data Monitoring Committee and Trial Oversight Committee play an important role in the conduct of the trial.

Industry collaborators are a subset of the people who have worked, on relevant sections, with the trial staff to ensure the trial runs efficiently.

TRIAL MANAGEMENT GROUP
(Listing only member external to the trials unit or senior staff at the trials unit)

Area	Status	Member	Geography	Role
Clinical/Surgical	Current	Gerhardt Attard	London (UCL), UK	CCI
~	~	Simon Chowdhury	London (Guys), UK	
~	~	Noel Clarke	Manchester, UK	Deputy CI, Co-CCI
~	~	William Cross	Leeds, UK	
~	~	David Dearnaley	Sutton, UK	
~	~	Silke Gillessen	Lugano, Switzerland[1]	CCI
~	~	Nicholas James	London (ICR), UK[2]	CI
~	~	Rob Jones	Glasgow, UK	
~	~	Zafar Malik	Wirral, UK	
~	~	Chris Parker	Sutton, UK	CCI
~	~	J Martin Russell	Glasgow, BOC	
~	Previous	Daniel Aebersold	Berne, Switzerland	
~	~	John Anderson	Sheffield, UK	
~	~	Johann de Bono	Sutton, UK	
~	~	Malcolm Mason	Cardiff, UK	
~	~	John Masters	London, UK	
~	~	Rick Popert	London (Guys), UK	
~	~	Alastair Ritchie	Gloucester, UK	
~	~	George Thalmann	Berne, Switzerland	
PPI	Current	David Matheson	Other	
~	~	Robin Millman	~	
~	Previous	John Dwyer	~	
~	~	David Hoe-Richardson	~	
~	~	Jim Stansfeld	~	
Senior CTU	Current	Claire Amos	MRC CTU at UCL	
~	~	Nafisah Atako	~	
~	~	Louise Brown	~	
~	~	Adrian Cook	~	
~	~	Duncan Gilbert	~	
~	~	Ruth Langley	~	CCI
~	~	Mahesh Parmar	~	Programme Lead[3]
~	~	Matthew Sydes	~	
~	Previous	Cheryl Pugh	~	
Clinical Fellow	Current	Hoda Abdely-Aty	~	
~	Previous	Clare Gilson	~	
~	~	Archie MacNair	~	
~	~	Hannah Rush	~	

Key: CI = Chief Investigator
CCI = Comparison CI
CoCCI = Comparison Co-CI

Note: The full list of MRC CTU at UCL staff is detailed below in a subsequent section.

[1] Previously Manchester, UK & St Gallen, Switzerland
[2] Previously Birmingham, UK & Warwick, UK
[3] Also CTU Director

INDEPENDENT DATA MONITORING COMMITTEE
(All members independent)

Member	Status	Role
Richard Emsley	Current	
Bertrand Tombal	~	Chair 3
Ronald de Wit	~	
Chris Williams	Previous	Chair 1
John Yarnold	~	Chair 2
Doug Altman	~	
Reg Hall	~	

TRIAL STEERING COMMITTEE
(Listing only independent members)

Member	Status	Role
Paula Ghaneh	Current	Chair 3
Tim Clayton	~	
Jan-Erik Dember	~	
Jonathan Ledermann	Previous	Chair 1
James Larkin	~	Chair 2
Richard Emsley	~	
John Fitzpatrick	~	
Alan Horwich	~	
David Kirk	~	
Jim Paul	~	

MRC CLINICAL TRIALS UNIT AT UCL STAFF

Area	Status	Name
Statisticians	Current	Christopher Brawley
~	~	Louise Brown
~	~	Adrian Cook
~	~	Laura Murphy
~	~	Matthew Nankivell
~	~	Mahesh Parmar
~	~	Matthew Sydes
~	Previous	Sophie Barthel
~	~	Daniel Bratton
~	~	Babak Choodari-Oskooei
~	~	Trinh Duong
~	~	Andrew Embleton
~	~	Melissa Gannon (nee Spears)
~	~	Fiona Ingleby
~	~	Elizabeth James
~	~	Rachel Jinks (nee Morgan)
~	~	Gordana Jovic
~	~	Patrick Royston
Project and Trial Management	Current	Claire Amos
~	~	Mazna Anjum
~	~	Nafisah Atako
~	~	Michelle Buckner
~	~	Sofeya Ishqa
~	~	Claire Murphy
~	~	Malissa Richmond
~	Previous	Shabinah Ali
~	~	Alanna Brown
~	~	Joanna Calvert
~	~	Charlene Carvalho
~	~	Tom Fairfield
~	~	Silvia Forcat
~	~	Michelle Gabriel
~	~	Charlene Green
~	~	Anna Herasimtschuk
~	~	Caroline Hogan
~	~	Sarah Jackson
~	~	Neil Kelk
~	~	James Latham
~	~	Dymphna Lee
~	~	Sarah Miller
~	~	Sharon Naylor
~	~	Dipa Noor
~	~	Jacqui Nuttall
~	~	Jenny Petrie
~	~	Orla Prendiville
~	~	Cheryl Pugh
~	~	Karen Sanders
~	~	Francesca Schiavone
~	~	Aminata Sy
~	~	Charlotte Tyson
~	~	Hannah Vaughan

MRC CLINICAL TRIALS UNIT AT UCL STAFF

Area	Status	Name
~	~	Christopher Wanstall
~	~	Katie Ward
~	~	Melanie Weiss
~	~	Arlen Wilcox
Clinicians	Current	Hoda Abdel-Aty
~	~	Duncan Gilbert
~	~	Ruth Langley
~	Previous	Clare Gilson
~	~	Archie Macnair
~	~	Sarah Meredith
~	~	Alastair Ritchie
~	~	Hannah Rush
Data Scientists and Programmers	Current	Carlos Diaz-Montana
~	~	Lindsey Masters
~	~	Nadine Vanlooy
~	Previous	Carly Au
~	~	Will Cragg
~	~	Carlos Diaz Montana
~	~	Dominic Hague
~	~	Zaheer Islam
~	~	Sajad Khan
~	~	Dominic Mounsey
~	~	Mary Rauchenberger
~	~	Nancy Tappenden
~	~	Stephen Townsend
~	~	Nadine Van-Looy
Data Management	Current	Ify Ejizu-Allen
~	~	Margaret Hook
~	~	William Hudson
~	~	Tasheeka Jeyapalan
~	~	Alexander Lawton
~	~	Meghna Pandya
~	~	Nazia Parkar
~	Previous	Eva Ades
~	~	Carly Au
~	~	Katherine Beaney
~	~	Nargis Begum
~	~	Katharine Bellenger
~	~	Lina Bergstrom
~	~	Veronica Birzu
~	~	Elizabeth Clark
~	~	Emma Donoghue
~	~	Amy Fiddament
~	~	Shree Gajjar
~	~	Hannah Gardner
~	~	Jenna Grabey
~	~	Richard Gracie
~	~	Charlene Green
~	~	Adam Gregory

MRC CLINICAL TRIALS UNIT AT UCL STAFF

Area	Status	Name
~	~	Dominic Hague
~	~	Shama Hassan
~	~	Jordan Hedges
~	~	Robyn Henry-Cockles
~	~	Sofeya Ishaq
~	~	Danielle Johnson
~	~	Saba Khan
~	~	Zohrah Khan
~	~	Adele Mabley
~	~	Georgia Mannion-Krase
~	~	Jacque Millett
~	~	Brendan Murphy
~	~	Myfanwy Nicholas
~	~	Sara Peres
~	~	Tasmin Philips
~	~	Philip Pollock
~	~	Tim Smith
~	~	Hannah Sweeney
~	~	Laura Van Dyck
~	~	Hannah Vaughan
~	~	Peter Vaughan
~	~	Steph Wetton
~	~	Andrew Whitney
Other Operations	Current	Fleur Hudson
~	~	Nicola Joffe
~	~	Macey Murray
~	Previous	Michelle Gabriel
Trial Assistants	Current	Elizabeth Adesanya
~	~	Yumna Ali
~	~	Atma Amin
~	~	Hannah Babiker
~	~	Bryony Bathie
~	~	Helen Chapman
~	~	Georgia Cowley
~	~	Leigh Dobson
~	~	James Dunn
~	~	Robbie Dunn
~	~	Amy Fiddament
~	~	Tracey Fisher
~	~	Tracy Fisher
~	~	Ben Forson
~	~	Adam Gregory
~	~	Nasir Jamil
~	~	Tasheeka Jeyapalan
~	~	Harry Kitson
~	~	Rebecca Lo
~	~	Joseph Martin
~	~	Nour Merzouki
~	~	Lynda Micklewright
~	~	Ray Phillips

MRC CLINICAL TRIALS UNIT AT UCL STAFF

Area	Status	Name
~	~	Jamie Simmons
~	~	Shanaz Sohail
~	~	Jeevan Sohal
~	~	Nat Thorogood
~	~	Stephanie Tsenti
~	~	Alexandra Wadia
~	~	Stephanie Wetton
STOPCAP Meta-Analysis team	Current	Sarah Burdett
	~	David Fisher
	~	Peter Godolphin
	~	Larysa Rydzewska
	~	Jayne Tierney
	~	Claire Vale

SWISS GROUP FOR CANCER CLINICAL RESARCH (SAKK) STAFF

Area	Status	Name
SAKK operations		Estelle Cassolly<br>Eloïse Kremer<br>Corinne Schar

BIOLOGY AND IMAGING SUBGROUPS
(Members of translational subgroups or work packages; TMG members not repeated here)

Person	Status	Geography
Adnan Ali	Current	Manchester, UK
Radhi Anand	~	London (UCL), UK
Dan Berney	~	London (Barts), UK
Hassan Douis	~	Birmingham, UK
Ros Eeles	~	London (ICR), UK
Stephenie Friedrich	~	London (UCL), UK
Emily Grist	~	London (UCL), UK
Anis A Hamid	~	Melbourne, Aus & Boston (DFCI), USA
Aine Haran	~	Manchester, UK
A M Mahedi Hassan	~	London (UCL), UK
Alex Hoyle	~	Manchester, UK
Sakunthala Kudahetti	~	London (Barts), UK
Sharanpreet Lall	~	London (UCL), UK
Gianmarco Leone	~	London (UCL), UK
Hing Leung	~	Glasgow, BOC
Stefano Lise	~	London (UCL), UK
Larissa Mendes	~	London (UCL), UK
Karolina Nowakowska-Pawelkowicz	~	London (UCL), UK
Charles Parker	~	London (UCL), UK
Marina Parry	~	London (UCL), UK
Alison Parry-Jones	~	Cardiff, UK
Chris Sweeney	~	Boston (DFCI), USA
Suparna Thakali	~	London (UCL), UK
Nina Tinariu	~	London (ICR), UK
Maria Vico	~	London (UCL), UK
Sara Santos Vidal	~	London (Barts), UK
Daniel Wetterskog	~	London (UCL), UK
Anna Wingate	~	London (UCL), UK
Carla Bautista	Past	London (UCL), UK
Paolo Cremaschi	~	London (UCL), UK
Thomas Hambrock	~	Manchester, UK
Alex Landless	~	London (UCL), UK
Nik Matthews	~	London (ICR), UK
Mariana Buongermino Pereira	~	London (UCL), UK
Kamila Sychowska	~	London (UCL), UK
David Waugh	~	Belfast, UK
Leila Zakka	~	London (UCL), UK

INVESTIGATORS AND COLLABORATORS: SITE STAFF

Staff on site delegation logs

City	Care_Site	Person_Name	Site_PI
Abergavenny, UK	Nevill Hall Hospital	Christian Smith	
Aberystwyth, UK	Bronglais General Hospital	Elin Jones	PI
~	~	Russel Canavan	
~	~	Kirsty Marie Dennett	
~	~	Claire Duggan	
~	~	Sajid Durrani	
~	~	Bleddyn Edwards	
~	~	John Edwards	
~	~	Sandra Evens	
~	~	Abigail Hynes	
~	~	Basharat Jameel	
~	~	Gwenan Parry Jones	
~	~	Philip Jones	
~	~	Rhian Elin Jones	
~	~	Sarah Jones	
~	~	Christine Kotonya	
~	~	Ronda Loosley	
~	~	Heather McGuinness	
~	~	Cerith Morgan	
~	~	Geraint Morgan	
~	~	Mark Narain	
~	~	Emma Nurse	
~	~	Donna Robson	
~	~	Llinos Strange	
~	~	Helen Tench	
~	~	Sean Thomas	
~	~	Toby Frederick Trugeion-Smith	
~	~	Kenneth Richard Williams	
~	~	Rebecca Wolf-Roberts	
Ashford, UK	William Harvey Hospital	Carys Thomas	PI
~	~	Albert Edwards	Co-I
~	~	Jessica Little	Co-I
~	~	Natasha Mithal	Co-I

INVESTIGATORS AND COLLABORATORS: SITE STAFF
Staff on site delegation logs

City	Care_Site	Person_Name	Site_PI
~	~	Rakesh Raman	Co-I
~	~	Jennifer Turner	Co-I
~	~	Louise Allen	
~	~	Bonny Appleby	
~	~	Sharon Beesley	
~	~	Hayley Blackgrove	
~	~	Tracy Boakes	
~	~	Patryk Brulinski	
~	~	Julie Buckley	
~	~	Miguel Capo-Mir	
~	~	Natalie Catt	
~	~	Mathilda Cominos	
~	~	Denise Crawford	
~	~	Nikki Crisp	
~	~	Steve Dann	
~	~	Julie-Ann Davies	
~	~	Susan Drakeley	
~	~	Clary Evans	
~	~	Sam Gibson	
~	~	Andrew Gillian	
~	~	Louise Gladwell	
~	~	Coral Greenstreet	
~	~	Tessa Hammond	
~	~	Sandra Holness	
~	~	Laura Kehoe	
~	~	Sue Kelly	
~	~	Rachel Larkins	
~	~	Kathryn Lees	
~	~	Sarah Lightfoot	
~	~	Sarah Lines	
~	~	Margaret Lipsham	
~	~	Sydnie Loveland	
~	~	Rohit Malde	

INVESTIGATORS AND COLLABORATORS: SITE STAFF
Staff on site delegation logs

City	Care_Site	Person_Name	Site_PI
~	~	Kim Mears	
~	~	Sharon Middleton	
~	~	Arafat Mirza	
~	~	Kannon Nathan	
~	~	Udaiveer Panwar	
~	~	Claire Pelham	
~	~	Karen Robinson	
~	~	Susan Rogers	
~	~	Lesley Rose	
~	~	Cindy Slater	
~	~	Mathini Sridharan	
~	~	Stephane Tankoua	
~	~	Katy Taylor	
~	~	Kim Travis	
~	~	Alba Tubau	
~	~	Ifigenia Vasiliadou	
~	~	Kathleen (Kathy) Walsh	
~	~	Paula Whichelo	
~	~	Claire White	
~	~	Joanne Williams	
~	~	Elizabeth Williamson	
~	~	Victoria Williamson	
~	~	Marian Wood	
~	~	Linda Wray	
~	~	Hilary Zurakovsky	
Aylesbury, UK	Stoke Mandeville Hospital	Katherine Hyde	PI
~	~	Philip Camilleri	Co-I
~	~	Thinn Pwint	Co-I
~	~	Christopher Alcock	
~	~	Maggie Aldersley	
~	~	Gerard Andrade	
~	~	Bhavna Badiani	
~	~	Jasvinder Bains	

INVESTIGATORS AND COLLABORATORS: SITE STAFF
Staff on site delegation logs

City	Care_Site	Person_Name	Site_PI
~	~	Margaret Bowerbank	
~	~	Joanne Brady	
~	~	Chrissie Butcher	
~	~	Janice Carpenter	
~	~	Prabir Chakraborti	
~	~	Christine Collins	
~	~	Siobhan Gettings	
~	~	Jonathan Greenland	
~	~	Kathryn Herbert	
~	~	Iram Husain	
~	~	Manisha Joshi	
~	~	Roisin Kavanagh	
~	~	Rahul Kurup	
~	~	Rossana Mancinelli	
~	~	Sarah Manyangadze	
~	~	Moncy Mathew	
~	~	Alice Ngumo	
~	~	Sean O'Cathail	
~	~	Anna Osadcow	
~	~	Cheryl Padilla-Harris	
~	~	Niki Panakis	
~	~	Andrew Protheroe	
~	~	Ami Sabharwal	
~	~	Tracey Stammers	
~	~	Michelle Taylor-Siddons	
~	~	Andy Theobold	
~	~	Neil Trew-Smith	
~	~	Gail Varley	
~	~	Janet Weir	
~	~	Hazel Wynn	
Ayr, UK	Ayr Hospital	Hilary Glen	PI
~	~	Xia Ren	Co-I
~	~	Jawaher Ansari	

INVESTIGATORS AND COLLABORATORS: SITE STAFF
Staff on site delegation logs

City	Care_Site	Person_Name	Site_PI
~	~	Helena Belikova	
~	~	Philip Cannon	
~	~	Deborah Dunn	
~	~	Danielle Gilmour	
~	~	Dianne Hunter	
~	~	Ricky Hunter	
~	~	Jennifer Keith	
~	~	Esfandiyar Khan	
~	~	Christina Lai	
~	~	Kirsten Laws (nee Borthwick)	
~	~	Clare Love	
~	~	Nicholas Macleod	
~	~	Rana Mahmood	
~	~	Jane McClements	
~	~	Brian McGlynn	
~	~	David McIntosh	
~	~	Margaret McKernan	
~	~	Lynne McNeil	
~	~	Sharon Meehan	
~	~	Jenna Mitchell	
~	~	Rebecca Muirhead	
~	~	Alison Murphy	
~	~	Stefan Nowich	
~	~	Kirsty O'Hara	
~	~	Kristy Ross	
~	~	Kathleen Smith	
~	~	Maureen Templeton	
~	~	Lye Mun Tho	
~	~	Aisha Tufail	
~	~	Claudia Turley	
~	~	Susan Walton	
~	~	Elaine Watson	
~	~	Lillian White	

INVESTIGATORS AND COLLABORATORS: SITE STAFF

Staff on site delegation logs

City	Care_Site	Person_Name	Site_PI
~	~	Mark Wilson	
~	~	Diane Woodburn	
~	~	Danna Yorston	
Barnet, UK	Barnet General Hospital	Sarah Needleman	PI
~	~	Ursula McGovern	Ex-PI
~	~	Kimberley Durno	Co-I
~	~	Magdalena Kubiak	Co-I
~	~	Kate Smith	Co-I
~	~	Anita Amadi	
~	~	Alice Coady	
~	~	Danielle Collier	
~	~	Veronica Conteh	
~	~	Andie David	
~	~	Andrew Eichholz	
~	~	Christine Ellis	
~	~	Annette Hawkins	
~	~	Heather Hughes	
~	~	Gillian Marks	
~	~	Anita Mitra	
~	~	Panayiotis Panayiotou	
~	~	Prital Patel	
~	~	Emily Scott	
Barnstaple, UK	North Devon District Hospital	Denise Sheehan	PI
~	~	Victoria Ford	Co-I
~	~	Peter Stephens	Co-I
~	~	Lynsey Balmbra-Jenks	
~	~	Maria Beaumont	
~	~	Helen Black	
~	~	Andy Bull	
~	~	Susan Collard	
~	~	Jenna Furse	
~	~	Henry Goss	
~	~	Joshua Gregory	

INVESTIGATORS AND COLLABORATORS: SITE STAFF
Staff on site delegation logs

City	Care_Site	Person_Name	Site_PI
~	~	Laura Hanson	
~	~	Becky Holbrook	
~	~	Katherine Horder	
~	~	Faisal Hussain	
~	~	Natalie Kemp	
~	~	Elizabeth Kershaw	
~	~	Michal Ian Lamparski	
~	~	Samantha Ley	
~	~	Judyta Lomza	
~	~	Ajaz Lone	
~	~	Nyasha Manomano	
~	~	Maria Martinez	
~	~	Martin Moody	
~	~	Chantal Oelofse	
~	~	Eng Ong	
~	~	Hannah Ong	
~	~	Sarah Park	
~	~	Chloe Peters	
~	~	Rufus Smith	
~	~	Amy Thomas	
~	~	Fiona Thomas	
~	~	Elizabeth Toy	
~	~	Lynne Van Koutrik	
~	~	Lynne Van-Koutrik	
~	~	Faye Windsor	
Basingstoke, UK	Basingstoke and North Hampshire Hospital	Sangeeta Paisey	PI
~	~	Richard Shaffer	Ex-PI
~	~	Katherine Aitken	
~	~	David Barlow	
~	~	Nanda Basker	
~	~	Louise Beattie	
~	~	Godfrey Bownie-Mukumbu	
~	~	Rachel Bryan	

INVESTIGATORS AND COLLABORATORS: SITE STAFF
Staff on site delegation logs

City	Care_Site	Person_Name	Site_PI
~	~	Jo-Anna Conyngham	
~	~	Duncan Cooke	
~	~	Victoria Corner	
~	~	Abigail Edwards	
~	~	Sara Fawcitt	
~	~	Adrienn Fazekasne Fulep	
~	~	Angela Frith	
~	~	Teresa Guerrero-Urbano	
~	~	Julie Gwilt	
~	~	Liz Happle	
~	~	Roger Hudson	
~	~	Lauriane Kerwood	
~	~	Kathryn Leach (nee Noake)	
~	~	Eva Letalova	
~	~	Christina Narh	
~	~	Jenny Nobes	
~	~	Bintha Paruthickal	
~	~	Christine Podesta	
~	~	Pennie Porter	
~	~	Helen Richards	
~	~	Catherine Rimington	
~	~	Fasar Sarwar	
~	~	Jackie Smith	
~	~	Joanna Stokoe	
~	~	Sree Susaria	
~	~	Rao Vuyyuru	
~	~	Katharine Webb	
~	~	Rosalyne Westley	
~	~	Ingrid White	
~	~	Claire Williams	
~	~	Rebecca Wills	
~	~	Katie Wood	
~	~	Carmen Wu	

INVESTIGATORS AND COLLABORATORS: SITE STAFF
Staff on site delegation logs

City	Care_Site	Person_Name	Site_PI
~	~	Hilawati Yusof	
Bath, UK	Royal United Hospital	Mark Beresford	PI
~	~	Olivera Frim	Co-I
~	~	Catherine McDonald	Co-I
~	~	Nathalie Webber	Co-I
~	~	Tom Wilson	Co-I
~	~	Rowan Appleby	
~	~	Joanne Avis	
~	~	Gareth Ayre	
~	~	Claire Barron	
~	~	Hannah Blades	
~	~	Rachael Bolitho	
~	~	Ruth Brydon-Hill	
~	~	Shaolin Chidavaenzi	
~	~	Vicki Clarke	
~	~	Christine Cox	
~	~	Claire Craige	
~	~	Jane Crozier	
~	~	Samantha Curtis	
~	~	Michael Daly	
~	~	Jackie Davies	
~	~	Claire Davis	
~	~	Frances Du Feu	
~	~	Claire Dyke	
~	~	Christine Elwell	
~	~	Rachael Exley	
~	~	Yuko Francis	
~	~	Beatrice Hamilton	
~	~	Leonie Harrison	
~	~	Lorna Hawley	
~	~	Abigail Jenner	
~	~	Penny Kehagioglou	
~	~	Carly Laxon-Takooree	

INVESTIGATORS AND COLLABORATORS: SITE STAFF
Staff on site delegation logs

City	Care_Site	Person_Name	Site_PI
~	~	Guillaume Livera	
~	~	Jill MacDonald-Burn	
~	~	Katarzyna Machura	
~	~	Margaret Macmillan	
~	~	Susan Masson	
~	~	Carey Milsom (nee Logan)	
~	~	Kate Moloney	
~	~	Sarah Murdoch	
~	~	Joseph Needham	
~	~	Hugh Newman	
~	~	Abigail Pocock	
~	~	Vicki Portingale	
~	~	Bryony Robertson	
~	~	Matthew Sephton	
~	~	Eve Tomlinson	
~	~	Tom Tylee	
~	~	Kristelle Vassallo	
~	~	Rebecca Wassall	
~	~	Jess White	
~	~	Chris Williams	
~	~	Samantha Williams	
~	~	Tania Williams (Née Allen)	
~	~	Joanna Wilson	
Bebington, UK	Clatterbridge Centre for Oncology	Zafar Malik	PI
~	~	Azman Ibrahim	Co-I
~	~	Ian Allen	
~	~	Wesley Artist	
~	~	Lisa Dobson (nee Child)	
~	~	Caroline Dunn	
~	~	Sharon Dunn (nee Johnson)	
~	~	Annemieke Earnshaw	
~	~	Diane Fildes	
~	~	Helen Flint	

INVESTIGATORS AND COLLABORATORS: SITE STAFF
Staff on site delegation logs

City	Care_Site	Person_Name	Site_PI
~	~	Elizabeth Gallimore	
~	~	Pat Gillis	
~	~	Sue Green	
~	~	Paul Griffiths	
~	~	Elizabeth Harrison	
~	~	Alison Hassall	
~	~	Jodie Henderson	
~	~	Kathryn Hughes	
~	~	Jess Hulse	
~	~	Helen Innes	
~	~	John Littler	
~	~	Laurie Lomax	
~	~	Linda Lyons	
~	~	Suzanne Maloney	
~	~	Laura McAllister	
~	~	Amir Montazeri	
~	~	Priyank Patel	
~	~	Dawn Porter	
~	~	Sandra Robinson	
~	~	Peter Robson	
~	~	Katie Sloan	
~	~	Matthew Stott	
~	~	Isabel Syndikus	
~	~	Shaun Tolan	
~	~	Emma Whitby	
~	~	Burhan Zavery	
Belfast, UK	Belfast City Hospital	Joe O'Sullivan	PI
~	~	Suneil Jain	Co-I
~	~	Swati Ray	Co-I
~	~	Poh Lin Shum	Co-I
~	~	Melvyn Ang	
~	~	Ruth Boyd	
~	~	Ellen Brown	

INVESTIGATORS AND COLLABORATORS: SITE STAFF

Staff on site delegation logs

City	Care_Site	Person_Name	Site_PI
~	~	Aishleen Brunton	
~	~	Patricia Calisaya	
~	~	Karen Campfield	
~	~	Peter Clarke	
~	~	Aiden Cole	
~	~	Wendy Cunningham	
~	~	Benedict Dadebo	
~	~	Prantik Das	
~	~	Catherine Davidson	
~	~	Mairead Devine	
~	~	Eileen Dillon	
~	~	Geraldine Douris	
~	~	Ruth Eakin	
~	~	Rachel Ellis	
~	~	Rhun Evans	
~	~	Ciaran Fairmichael	
~	~	Rebecca Goody	
~	~	Chris Hagan	
~	~	Emma Hanna	
~	~	Michael Hanna	
~	~	Jackie Harney	
~	~	Barbara Harvey	
~	~	Eimear Henry	
~	~	Stacey Hetherington	
~	~	Naomi Hill	
~	~	Sharon Hynds	
~	~	Lucy Jellett	
~	~	Ruth Johnston	
~	~	Sai Jonnada	
~	~	Patrick Keane	
~	~	Grace Lavery	
~	~	Diane Law	
~	~	Alison Logie	

INVESTIGATORS AND COLLABORATORS: SITE STAFF
Staff on site delegation logs

City	Care_Site	Person_Name	Site_PI
~	~	Jonathan McAleese	
~	~	Chryelle McAlister	
~	~	Seosamh McCauley	
~	~	Sharon McClean	
~	~	Paula McCloskey	
~	~	Kairen McCloy	
~	~	Sara McCusker(nee Stokes)	
~	~	Sarah McGahey	
~	~	Ciara McIlmunn	
~	~	Karen McKenna	
~	~	Shirley McKenna	
~	~	Aine McKeown	
~	~	Michael McMahon	
~	~	Linda McNeice	
~	~	Darren Mitchell	
~	~	Laura Mooney	
~	~	Angela Morrison	
~	~	Lynsey Morrow	
~	~	Lois Mulholland	
~	~	Kerry Nicholls	
~	~	Adrina O'Donnell	
~	~	Karen Parsons	
~	~	Jemma Robinson	
~	~	Claire Rooney	
~	~	Keith Rooney	
~	~	Angela Rosbotham	
~	~	William Snelling	
~	~	David Stewart	
~	~	Stephen Stranex	
~	~	Fiona Tarpey	
~	~	Jonathan Thompson	
~	~	Joanne Todd	
~	~	Phil Turner	

INVESTIGATORS AND COLLABORATORS: SITE STAFF
Staff on site delegation logs

City	Care_Site	Person_Name	Site_PI
~	~	Salil Vengalil	
Birmingham, UK	Birmingham Heartlands Hospital	Anjali Zarkar	PI
~	~	Kamaldeep Ajimal	
~	~	Chen Bartlett	
~	~	Madhura Chandrashekara	
~	~	Ellen Drew	
~	~	Mary (Ellen) Drew	
~	~	Penny Goodby (nee Harbach	
~	~	Samarah Haq	
~	~	Adrian Kelly	
~	~	Jill Lyons	
~	~	Alison Maidment	
~	~	Janet Prentice	
~	~	Julia Sampson	
~	~	Ann Schumacher	
~	~	Frances Shaw	
~	~	Michael Tarn	
~	~	James Whitehouse	
Birmingham, UK	City Hospital (Birmingham)	Emilio Porfiri	PI
~	~	Robert Stevenson	Co-I
~	~	Sachin Trivedi	Co-I
~	~	Laura Butler	
~	~	Yin May Chin	
~	~	Joanne Dasgin	
~	~	Debbie Devonport	
~	~	Daniel Ford	
~	~	Brian Gammon	
~	~	Harriet Goddard	
~	~	Jasbinder Kaur	
~	~	Alice Longe	
~	~	Amy Orme	
~	~	Lalit Pallan	
~	~	Steven Shanu	

INVESTIGATORS AND COLLABORATORS: SITE STAFF

Staff on site delegation logs

City	Care_Site	Person_Name	Site_PI
~	~	Julie Simpson	
~	~	Marion Tatman	
~	~	Angela Williams	
Birmingham, UK	Queen Elizabeth Hospital (Birmingham)	David Fackrell	PI
~	~	Nicholas James	Ex-PI
~	~	Daniel Ford	Co-I
~	~	Emilio Porfiri	Co-I
~	~	Kathryn Adams	
~	~	Salma Afzal	
~	~	Nicola Anderson	
~	~	Jay Ansari	
~	~	Biruk Asfaw	
~	~	Maria Bandeira	
~	~	Erica Beaumont	
~	~	Mahmoda Begum	
~	~	Shaleen Bishop	
~	~	Lea Booth	
~	~	Trish Brady	
~	~	Emma Bruce	
~	~	Laura Butler	
~	~	Laura Caley	
~	~	Helen Clarke	
~	~	Gemma Cole	
~	~	Jane Cook	
~	~	Amanda Davies	
~	~	Sara Diffley	
~	~	Claire Draycott	
~	~	Alison Grant	
~	~	Joanna Gray (nee Finney)	
~	~	Daniel Henderson	
~	~	Rosie Henvey	
~	~	Jenny Hiley	
~	~	Sharon Holmes	

INVESTIGATORS AND COLLABORATORS: SITE STAFF
Staff on site delegation logs

City	Care_Site	Person_Name	Site_PI
~	~	Sam Hopkins (nee Poole)	
~	~	Sameed Hussain	
~	~	Heather Jones	
~	~	Helen Jones	
~	~	Pamela Jones	
~	~	Alice Longe	
~	~	Daniella Lynch	
~	~	Fahd Niaz	
~	~	Andrew Palmer	
~	~	Stephanie Palmer	
~	~	Jenny Pascoe	
~	~	Zhane Peterkin	
~	~	Helen Preston	
~	~	Charlotte Sabine	
~	~	Rosemarie Seadon	
~	~	Amna Shah	
~	~	Tracy Soulsby	
~	~	Catherine Stead	
~	~	Lisa Thomas	
~	~	Syed Tirmazy	
~	~	Hannah Tolson	
~	~	Charlotte Trinham	
~	~	Arvind Tripathy	
~	~	Hannah Tween	
~	~	Vishy Veeranna	
~	~	Abel Zachariah	
~	~	Anjali Zarkar	
Blackburn, UK	Blackburn Royal Infirmary	Natalie Charnley	
Blackburn, UK	Royal Blackburn Hospital	Omi Parikh	PI
~	~	Zhu Oong	Co-I
~	~	Sophie Raby	Co-I
~	~	Danya Abdulwahid	
~	~	Ilyas Ahmed	

INVESTIGATORS AND COLLABORATORS: SITE STAFF
Staff on site delegation logs

City	Care_Site	Person_Name	Site_PI
~	~	Sarah Ainsworth	
~	~	Sue Ashworth	
~	~	Hazel Aston	
~	~	Ana Batista	
~	~	Karen Beard	
~	~	Gaynor Bowen	
~	~	Andrew Brocklehurst	
~	~	Fatima Butt	
~	~	Jackie Carey	
~	~	Naomi Charlton	
~	~	Helene Chorley	
~	~	Jenny Cockerill-Taylor	
~	~	Ruth Conroy	
~	~	Anthea Cree	
~	~	William Croxford	
~	~	Falalu Danwata	
~	~	Parth Desai	
~	~	Joseph Dykes	
~	~	Bethany Fielding	
~	~	Jan Flaherty	
~	~	Diane Forrest	
~	~	Helen Frankland	
~	~	James Grunshaw	
~	~	Samatha Guy	
~	~	Imran Haidar	
~	~	Hani Hanna	
~	~	Jeanette Hargreaves	
~	~	Kathryn Hayes	
~	~	Angela Hugill	
~	~	Andrew Hunnisett	
~	~	Rizwana Hussain	
~	~	Karen Jewers	
~	~	Sarah Keith	

INVESTIGATORS AND COLLABORATORS: SITE STAFF
Staff on site delegation logs

City	Care_Site	Person_Name	Site_PI
~	~	Prasad Kellati	
~	~	Tracey Kilduff	
~	~	Stephen Kilroy	
~	~	Jennifer King	
~	~	Andrew Lancaster	
~	~	Jasima Latif	
~	~	Matthew Lovell	
~	~	Jennifer McCallum	
~	~	Alexandra McCarrick	
~	~	Ajay Mehta	
~	~	Twesige Mugisa	
~	~	Tanmay Mukhopadhyay	
~	~	Jackie Nuttall	
~	~	Farzana Patel	
~	~	Karan Patel	
~	~	Graham Read	
~	~	Zia Rehman	
~	~	Karen Riley	
~	~	Christina Robinson	
~	~	Darren Rusk	
~	~	Janet Ryan-Smith	
~	~	Ahmed Salah	
~	~	Win Soe	
~	~	Helen Spickett	
~	~	Philippa Springle	
~	~	Dayle Squires	
~	~	Debbie Sutton	
~	~	Victoria Taylor	
~	~	Marianna Theodoulou	
~	~	Jacqueline Thomas	
~	~	Vivienne Tickle	
~	~	Richard Walshaw	
~	~	Lynsey Waring	

INVESTIGATORS AND COLLABORATORS: SITE STAFF
Staff on site delegation logs

City	Care_Site	Person_Name	Site_PI
~	~	Jessica Whiston	
~	~	Deborah Williamson	
~	~	Marcus Wise	
~	~	Maricica Zabrautanu	
Bolton, UK	Royal Bolton Hospital	Ling Lee	PI
~	~	Julie Chadwick	
~	~	Shirley Cocks	
~	~	Louise Dawson	
~	~	Tony Elliott	
~	~	Debbie Forkin	
~	~	Zoe Gall	
~	~	Robert Hull	
~	~	Collette Hunt	
~	~	Janine Hurst	
~	~	Karen Jewers	
~	~	Richard Jones	
~	~	Janet Keegan	
~	~	Karen Lee	
~	~	Charlotte Lever	
~	~	Ajay Mehta	
~	~	Raksha Mistry	
~	~	Gillian Mobb	
~	~	Michael Pantelides	
~	~	Hemant Patel	
~	~	Lindsay Rawlinson	
~	~	Sally Shaw	
Boston, UK	Pilgrim Hospital	Thiagarajan Sreenivasan	PI
~	~	Christian Arias	Co-I
~	~	David Ballesteros-Quintail	Co-I
~	~	Ana Fernandez-Ots	Co-I
~	~	Sekar (DV) Kittappa	Co-I
~	~	Miguel Panades	Co-I
~	~	Simon Archer	

INVESTIGATORS AND COLLABORATORS: SITE STAFF

Staff on site delegation logs

City	Care_Site	Person_Name	Site_PI
~	~	Giuseppe Banna	
~	~	Jayne Borley	
~	~	Eileen Busby	
~	~	Helen Carolan	
~	~	Prantik Das	
~	~	Jo Fletcher	
~	~	Andrew Judd	
~	~	Amy Kirkby	
~	~	Victoria Knight (n. Sherburn)	
~	~	Alice Latty	
~	~	Tara Lawrence nee Palmer	
~	~	Carol Lockwood	
~	~	Beverley Mashegede	
~	~	Karen Metcalf	
~	~	Sally Ann Molsher	
~	~	Kimberley Netherton	
~	~	Helen Palmer	
~	~	Kerry Pettitt	
~	~	Gunjan Phalod	
~	~	Sindhu Ramamurthy	
~	~	Amanda Roper	
~	~	Jenny Salmon	
~	~	Andrew Sloan	
~	~	Rebecca Spencer	
~	~	Kinga Szymiczek	
~	~	Isobel Thomas	
~	~	Laura Walsh	
~	~	Anita Young	
Bournemouth, UK	Royal Bournemouth Hospital	Sue Brock	PI
~	~	George Astras	
~	~	Natalya Boyd	
~	~	Eve Broadley	
~	~	David Chrastek	

INVESTIGATORS AND COLLABORATORS: SITE STAFF
Staff on site delegation logs

City	Care_Site	Person_Name	Site_PI
~	~	Joe Davies	
~	~	Deborah Hands	
~	~	Alison Hogan	
~	~	Lynsey Houlton	
~	~	Stephanie Jones	
~	~	Tiffany Joyce	
~	~	Katherine Major	
~	~	Rebecca Miln	
~	~	Nicky Naraine	
~	~	Natasha Ottley	
~	~	Kate Preece	
~	~	Laura Purandare	
~	~	Linda Purandare	
~	~	Cathie Purnell	
~	~	Taslima Rabbi	
~	~	Carlton Rowlands	
~	~	Sarah Savage	
~	~	Julie Thomson	
~	~	Luke Vamplew	
~	~	Rao Vuyyuru	
~	~	Min Wu	
Bradford, UK	Bradford Royal Infirmary	Simon Brown	PI
~	~	Michael Flatley	Co-I
~	~	Adel Jebar	Co-I
~	~	Lucy Jones	Co-I
~	~	Eldho Joseph	Co-I
~	~	Louise Karsera	Co-I
~	~	Sally Martin	Co-I
~	~	Sohail Mughal	Co-I
~	~	Lisa Owen	Co-I
~	~	Andrew Viggars	Co-I
~	~	Qamar Akbar	
~	~	Linda Bamford	

INVESTIGATORS AND COLLABORATORS: SITE STAFF
Staff on site delegation logs

City	Care_Site	Person_Name	Site_PI
~	~	Richard Benton	
~	~	Ian Boon	
~	~	Samuel Briggs	
~	~	Wendy Cardozo	
~	~	Sue Cheeseman	
~	~	Osman Chohan	
~	~	Ee Siang Choong	
~	~	Katy Clarke	
~	~	Kay Cockroft	
~	~	Victoria Drew	
~	~	Emma Dugdale	
~	~	Carol Firth	
~	~	Robina Ghulam	
~	~	Umair Hamid	
~	~	Catherine Handforth	
~	~	Ann Henry	
~	~	Hayley Inman	
~	~	Laura Jaques	
~	~	Ganesan Jeyasangar	
~	~	Charlotte Johnson-Smith	
~	~	Anne Marie Kay	
~	~	Lucille Kenyon	
~	~	Sophia Khan	
~	~	Leila Koudsi	
~	~	Jannika Lazarte	
~	~	Dan Lee	
~	~	Carmel Loughrey	
~	~	Reem Mahmood	
~	~	Leslie Masters	
~	~	Elizabeth McIntosh	
~	~	Dawn McNulty	
~	~	Chandran Nallathambi	
~	~	Gail Opio-Te	

INVESTIGATORS AND COLLABORATORS: SITE STAFF
Staff on site delegation logs

City	Care_Site	Person_Name	Site_PI
~	~	Shefali Parikh	
~	~	Mohammed Patel	
~	~	Charlotte Richardson	
~	~	Helen Robertshaw	
~	~	Sree Rodda	
~	~	Declan Ryan-Wakeling	
~	~	Jane Sewell	
~	~	Finbar Slevin	
~	~	Sophie Stephenson	
~	~	Kelvin Stewart	
~	~	Kim Storton	
~	~	Sarah Tinker	
~	~	Manitha Vinod	
~	~	Eleanor Waldron	
~	~	Lucy Ward	
~	~	Christopher Williams	
~	~	Helen Wilson	
~	~	You Yone	
~	~	Jamal Zekri	
~	~	Anthi Zeniou	
Bradford, UK	St Luke's (Bradford)	Susan Cheeseman	
Brighton, UK	Royal Sussex County Hospital	Angus Robinson	PI
~	~	George Plataniotis	Co-I
~	~	Dorota Bak-Blaz	
~	~	Lisa Barrott	
~	~	David Bloomfield	
~	~	Kirsty Bracewell	
~	~	Stephen Brown	
~	~	Maggie Cole	
~	~	Elizabeth Corbett	
~	~	Lucy Curtis	
~	~	George Devtsch	
~	~	Jane Dexter	

INVESTIGATORS AND COLLABORATORS: SITE STAFF

Staff on site delegation logs

City	Care_Site	Person_Name	Site_PI
~	~	Tarun Durga	
~	~	Rachel Rose Edmunds	
~	~	Emma Foreman	
~	~	Paul Frattaroli	
~	~	Lisa Furnival	
~	~	Jane Hanson	
~	~	Andrew Hart	
~	~	Daniel Henderson	
~	~	Samantha Hodges	
~	~	Catherine Hunter	
~	~	Summer Ibrahim	
~	~	Tamsin Kent	
~	~	Ranee Lactao	
~	~	Katie Langford	
~	~	Poppy Lavender	
~	~	Joanne Magennis	
~	~	Angela Man	
~	~	Pauline Martin	
~	~	Sebastien Martin	
~	~	Simon Matthews	
~	~	Helen Mitchell	
~	~	Amy Murray	
~	~	Monika Musiol	
~	~	Elaine Noon	
~	~	Annie Oliver	
~	~	Jane Peterson	
~	~	George Plantaniotis	
~	~	Alison Porges	
~	~	Tiago Rodrigues	
~	~	Tenesa Sargent	
~	~	Matthew Seal	
~	~	Victoria Sellick	
~	~	Jackie Sham	

INVESTIGATORS AND COLLABORATORS: SITE STAFF
Staff on site delegation logs

City	Care_Site	Person_Name	Site_PI
~	~	Jodie Smith	
~	~	Julie Smith	
~	~	Jean Tremlett	
~	~	Sue Trotter	
~	~	Vivien Tse	
~	~	Caroline Walker	
~	~	Karen Walker	
~	~	Chritianne Whitfield	
~	~	Marie Wilkins	
~	~	Bobbie Yoong	
Bristol, UK	Bristol Haematology & Oncology Centre	Amit Bahl	PI
~	~	Lloyd Abood	
~	~	Azeem Arshad	
~	~	Lindsay Ball	
~	~	Mark Beresford	
~	~	Sarah Bishop	
~	~	Jyothsna Chennupati	
~	~	Marc Coe	
~	~	Sibusiso Dhladhla	
~	~	Kay Drury	
~	~	Harvey Dymond	
~	~	Emily Foulstone	
~	~	Polly Gingell	
~	~	Tristan Grey	
~	~	Sally-Ann Hall	
~	~	Chris Herbert	
~	~	Serena Hilman	
~	~	Robert Hollister	
~	~	Amy Holloway	
~	~	Hayley Jones	
~	~	Stephen Lang	
~	~	Jayne Leonard	
~	~	Susan Masson	

INVESTIGATORS AND COLLABORATORS: SITE STAFF
Staff on site delegation logs

City	Care_Site	Person_Name	Site_PI
~	~	Shalini Mohan	
~	~	Hugh Newman	
~	~	Bryony Parrish	
~	~	Ian Penwarden	
~	~	Nick Robins	
~	~	Kimberly Rockley	
~	~	Helen Saldanha	
~	~	Sharon Short	
~	~	Beth Thorne	
~	~	Eve Watson	
~	~	Sandra Williams (nee Price)	
~	~	Paula Wilson	
~	~	Seonaid Wright	
Bristol, UK	Bristol Royal Infirmary	Lindsay Ball	
Burnley, UK	Burnley General Hospital	Omi Parikh	PI
~	~	Danya Abdulwahid	
~	~	Ilyas Ahmed	
~	~	Sarah Ainsworth	
~	~	Sue Ashworth	
~	~	Ana Batista	
~	~	Karen Beard	
~	~	Gaynor Bowen	
~	~	Andrew Brocklehurst	
~	~	Fatima Butt	
~	~	Jackie Carey	
~	~	Natalie Charnley	
~	~	Helene Chorley	
~	~	Ruth Conroy	
~	~	Anthea Cree	
~	~	Louise Dawson	
~	~	Bethany Fielding	
~	~	Jan Flaherty	
~	~	Diane Forrest	

INVESTIGATORS AND COLLABORATORS: SITE STAFF
Staff on site delegation logs

City	Care_Site	Person_Name	Site_PI
~	~	Helen Frankland	
~	~	Samatha Guy	
~	~	Imran Haidar	
~	~	Hani Hanna	
~	~	Jeanette Hargreaves	
~	~	Angela Hugill	
~	~	Rizwana Hussain	
~	~	Karen Jewers	
~	~	Sarah Keith	
~	~	Prasad Kellati	
~	~	Tracey Kilduff	
~	~	Stephen Kilroy	
~	~	Matthew Lovell	
~	~	Alexandra McCarrick	
~	~	Twesige Mugisa	
~	~	Tanmay Mukhopadhyay	
~	~	Farzana Patel	
~	~	Karan Patel	
~	~	Zia Rehman	
~	~	Karen Riley	
~	~	Christina Robinson	
~	~	Darren Rusk	
~	~	Janet Ryan-Smith	
~	~	Ahmed Salah	
~	~	Win Soe	
~	~	Helen Spickett	
~	~	Philippa Springle	
~	~	Dayle Squires	
~	~	Debbie Sutton	
~	~	Victoria Taylor	
~	~	Jacqueline Thomas	
~	~	Vivienne Tickle	
~	~	Richard Walshaw	

INVESTIGATORS AND COLLABORATORS: SITE STAFF

Staff on site delegation logs

City	Care_Site	Person_Name	Site_PI
~	~	Lynsey Waring	
~	~	Deborah Williamson	
~	~	Marcus Wise	
Burton-on-Trent, UK	Queen's Hospital Burton	Mike Smith-Howell	PI
~	~	Ann Adams	
~	~	Shahzad Ahmed	
~	~	Seheli Bandyopahdyay	
~	~	Gill Bell	
~	~	Jo Burns	
~	~	Lorraine Carter	
~	~	Prabir Chakraborti	
~	~	Shan Chetiyawardana	
~	~	Rosemary Corfield	
~	~	Helen Cox	
~	~	Helena Cox	
~	~	Chris Curtis	
~	~	Sudipta Datta	
~	~	Jacqueline Elliott	
~	~	Katy English (nee Parkes)	
~	~	Annette Fleet	
~	~	V Gajek	
~	~	Karzan Hama	
~	~	Sarah Hathaway-Lees	
~	~	Rajeev Kaushal	
~	~	Elizabeth Kemp	
~	~	Christopher Kent	
~	~	Ali Mahmmod	
~	~	Rohit Malde	
~	~	Chandrani Mallik	
~	~	Hanine Medani	
~	~	Clare Mewies	
~	~	Jennifer Moyes	
~	~	Dakshinamoorthy Muthukumar	

INVESTIGATORS AND COLLABORATORS: SITE STAFF
Staff on site delegation logs

City	Care_Site	Person_Name	Site_PI
~	~	Pugazhenthi Pattu	
~	~	Divya Ramadasan	
~	~	Anita Szita	
Bury St Edmunds, UK	West Suffolk Hospital	Cathryn Woodward	PI
~	~	Alex Martin	Co-I
~	~	Cherri Blades	
~	~	Gill Brett	
~	~	Deborah Clements-Dimmock	
~	~	James Curtis	
~	~	Elizabeth Devoy	
~	~	Yvonne Field	
~	~	Frances Flynn	
~	~	Susan Hale	
~	~	Mark Heath	
~	~	David Matter	
~	~	Tracey Murray	
~	~	Amanda Neal	
~	~	Lisa Patterson	
~	~	John Raja Ravendar	
~	~	Yvonne Rimmer	
~	~	Helen Small	
~	~	Jill Thain	
~	~	Fred Tuck	
Camarthen, UK	Glangwili General (formerly West Wales General)	Mau-Don Phan	PI
~	~	Sonya Goriah	Co-I
~	~	Samantha Coetzee	
~	~	Bleddyn Edwards	
~	~	Sandra Evens	
~	~	Ann Hewins	
~	~	Zohra Omar	
~	~	Bryan Phillips	
~	~	Meena Raj	
~	~	Rocio Riba	

INVESTIGATORS AND COLLABORATORS: SITE STAFF
Staff on site delegation logs

City	Care_Site	Person_Name	Site_PI
Cambridge, UK	Addenbrooke's Hospital	Danish Mazhar	PI
~	~	Tatiana Hernandez	Co-I
~	~	Rebecca Bradley	
~	~	Anita Chhabra	
~	~	Ellie Couch	
~	~	Gemma Cullen (née Godsall)	
~	~	Sandra Cunningham	
~	~	Mirela Hategan	
~	~	Carole Hewitt	
~	~	Luke Hughes-Davies	
~	~	Svitlana Iyevkova	
~	~	Gin Lee	
~	~	Rachel Lister	
~	~	Debra Mansergh	
~	~	Vanessa Moreira	
~	~	Isaac Opara	
~	~	Simon Pacey	
~	~	Glynn Rolland	
~	~	Matthew Stone	
~	~	Amy Strong n.Chandradass	
~	~	Andrew Styling	
~	~	James Tanner	
~	~	Safaa Therese	
~	~	Nicola Thompson	
~	~	Amanda Walker	
~	~	James Watson	
~	~	Han Wong	
~	~	Kamarul Zaki	
Canterbury, UK	Kent and Canterbury Hospital	Carys Thomas	PI
~	~	Patryk Brulinski	Co-I
~	~	Albert Edwards	Co-I
~	~	Joao Galante	Co-I
~	~	Jessica Gough	Co-I

INVESTIGATORS AND COLLABORATORS: SITE STAFF
Staff on site delegation logs

City	Care_Site	Person_Name	Site_PI
~	~	Jessica Little	Co-I
~	~	Natasha Mithal	Co-I
~	~	Rakesh Raman	Co-I
~	~	Alice Rendall	Co-I
~	~	Van Sim	Co-I
~	~	Ioannis Trigonis	Co-I
~	~	Jennifer Turner	Co-I
~	~	Ilyas Ahmed	
~	~	Louise Allen	
~	~	Bonny Appleby	
~	~	Sarah Beasley	
~	~	Sharon Beesley	
~	~	Hayley Blackgrove	
~	~	Tracy Boakes	
~	~	Julie Buckley	
~	~	Miguel Capo-Mir	
~	~	Natalie Catt	
~	~	Mathilda Cominos	
~	~	Denise Crawford	
~	~	Nikki Crisp	
~	~	Steve Dann	
~	~	Julie-Ann Davies	
~	~	Susan Drakeley	
~	~	Clary Evans	
~	~	Sam Gibson	
~	~	Andrew Gillian	
~	~	Louise Gladwell	
~	~	Coral Greenstreet	
~	~	Carolyn Hargreaves	
~	~	Gemma Hegarty	
~	~	Sandra Holness	
~	~	Laura Kehoe	
~	~	Sue Kelly	

INVESTIGATORS AND COLLABORATORS: SITE STAFF

Staff on site delegation logs

City	Care_Site	Person_Name	Site_PI
~	~	Rachel Larkins	
~	~	Kathryn Lees	
~	~	Sarah Lightfoot	
~	~	Sarah Lines	
~	~	Margaret Lipsham	
~	~	Diane Long	
~	~	Sydnie Loveland	
~	~	Rohit Malde	
~	~	Kim Mears	
~	~	Sharon Middleton	
~	~	Christos Mikropoulos	
~	~	Arafat Mirza	
~	~	Laura Mould	
~	~	Kannon Nathan	
~	~	Udaiveer Panwar	
~	~	Claire Pelham	
~	~	Karen Robinson	
~	~	Susan Rogers	
~	~	Lesley Rose	
~	~	Cindy Slater	
~	~	Mathini Sridharan	
~	~	Caroline Sunderland	
~	~	Stephane Tankoua	
~	~	Katy Taylor	
~	~	Kim Travis	
~	~	Alba Tubau	
~	~	Ifigenia Vasiliadou	
~	~	Kathleen (Kathy) Walsh	
~	~	Paula Whichelo	
~	~	Claire White	
~	~	Joanne Williams	
~	~	Elizabeth Williamson	
~	~	Victoria Williamson	

INVESTIGATORS AND COLLABORATORS: SITE STAFF
Staff on site delegation logs

City	Care_Site	Person_Name	Site_PI
~	~	Marian Wood	
~	~	Linda Wray	
~	~	Hilary Zurakovsky	
Cardiff, UK	University Hospital of Wales	Krishna Narahari	PI
~	~	Elizabeth Bois (nee Harris)	
~	~	Helen Clark	
~	~	Colette Clements	
~	~	Richard Coulthard	
~	~	Lynne Harry	
~	~	Samantha Holliday	
~	~	Clare Jones	
~	~	Howard Kynaston	
~	~	Kevin Pearse	
Cardiff, UK	Velindre Hospital	Jacob Tanguay	PI
~	~	Jim Barber	Co-I
~	~	Michael Button	Co-I
~	~	Aida Hanim Kamarudin	Co-I
~	~	Satish Kumar	Co-I
~	~	Malcolm Mason	Co-I
~	~	Nachiappan Palaniappan	Co-I
~	~	John Staffurth	Co-I
~	~	Kathy Bishop	
~	~	Clare Boobier	
~	~	Michael Brown	
~	~	Clair Brunner	
~	~	Lucy Chestney	
~	~	Helen Clark	
~	~	Lisa Victoria Jane Clayton	
~	~	Jessica Dermott (nee Platt)	
~	~	Clare Donnithorne	
~	~	Sarah Fry	
~	~	Sandra Greenslade	
~	~	Louise Harris	

INVESTIGATORS AND COLLABORATORS: SITE STAFF
Staff on site delegation logs

City	Care_Site	Person_Name	Site_PI
~	~	Nida Hassan	
~	~	Robert Henley	
~	~	Toby Hiscott	
~	~	Lynda Holman	
~	~	Gareth Hunt	
~	~	Amanda Jackson	
~	~	Rashmi Jadon	
~	~	Catherine John	
~	~	Alison Johnson	
~	~	Necia Jones	
~	~	Colette Kemp	
~	~	Lynette Lane	
~	~	Donna Lear	
~	~	Jason Lester	
~	~	Ross McLeish	
~	~	James Morgan	
~	~	Louise Morgan	
~	~	Phillip Morgan	
~	~	Diana Mort	
~	~	Debbie O'Connor	
~	~	Renata Poole	
~	~	Karen Pow	
~	~	Joanne Preece	
~	~	Leanne Quinn	
~	~	Tracy Rees	
~	~	Vicki Reynolds	
~	~	Cathy Richards	
~	~	Jayne Richards	
~	~	Emily Rumney	
~	~	Christian Smith	
~	~	Lisa Stafford	
~	~	Catherine Sullivan	
~	~	Loretta Sweeney	

INVESTIGATORS AND COLLABORATORS: SITE STAFF
Staff on site delegation logs

City	Care_Site	Person_Name	Site_PI
~	~	Hana Thomas	
~	~	Bethan Tranter	
~	~	Caroline Vitolo	
~	~	Lucy Wilbraham	
~	~	Gillian Willetts	
~	~	Kay Wilson	
~	~	Charlotte Young	
Carlisle, UK	Cumberland Infirmary	Fiona Douglas	PI
~	~	Anil Kumar	PI
~	~	Angela Birt	
~	~	Christopher Brewer	
~	~	Diane Donnelly	
~	~	Charlotte Eyles	
~	~	Grace Fryer	
~	~	Ivor Hughes	
~	~	Patricia Nicholls	
~	~	Jonathan Nicoll	
~	~	Muhammad Rahman	
~	~	Norma Sidek	
~	~	Jenna Wildey	
~	~	Beverley Wilkinson	
~	~	Fergus Young	
Chelmsford, UK	Broomfield Hospital	Abdel Hamid	PI
~	~	Gopalakrishnan Srinivasan	Co-I
~	~	Victoria Apps	
~	~	Christian Barnett	
~	~	Melanie Boxall	
~	~	Donna Briggs	
~	~	Frances Cairns	
~	~	Tracey Camburn	
~	~	Emma Cannon	
~	~	Jennifer Child	
~	~	Lucy Cooper	

INVESTIGATORS AND COLLABORATORS: SITE STAFF
Staff on site delegation logs

City	Care_Site	Person_Name	Site_PI
~	~	Elizabeth Dawson	
~	~	Sarah Ferguson	
~	~	Sian Gibson	
~	~	Jane Giles	
~	~	Dane Goodere-Bennett	
~	~	Kiran Kancherla	
~	~	Priscilla Leone	
~	~	Yvonne Lester	
~	~	Isabella Maund	
~	~	Emma Mitchell	
~	~	Udaiveer Panwar	
~	~	Enca Parsons	
~	~	Melanie Ruben	
~	~	Victoria Scott	
~	~	Bryan Singizi	
~	~	Edel Spruce	
~	~	Amon Wijunamai	
~	~	Lucy Willsher	
~	~	You Yone	
Cheltenham, UK	Cheltenham General Hospital	Jo Bowen	PI
~	~	Peter Jenkins	Co-I
~	~	Julie Allen	
~	~	Susan Anderson	
~	~	Charlotte Ayrton	
~	~	Helen Babbage	
~	~	Rehana Bakawala	
~	~	Sarah Beazer	
~	~	Victoria Bell	
~	~	Vishal Bhalla	
~	~	Lucy Blake	
~	~	Caitlin Bowden	
~	~	Rachel Carter	
~	~	Bethan Cartwright	

INVESTIGATORS AND COLLABORATORS: SITE STAFF
Staff on site delegation logs

City	Care_Site	Person_Name	Site_PI
~	~	Jyothsna Chennupati	
~	~	Jill Chittock	
~	~	Audrey Cook	
~	~	Samuel Croly	
~	~	Lin Crossley	
~	~	Jennifer Dewett	
~	~	Rachel Durrant	
~	~	Chris Ford	
~	~	Janet Forkes	
~	~	Julia Hall	
~	~	Jennifer Healey-Mariano	
~	~	Ian Ingledew	
~	~	Sai Jonnada	
~	~	Louise Kidner	
~	~	Laura Malins	
~	~	Rebecca Mesher	
~	~	Roger Owen	
~	~	Elisabeth Read	
~	~	Rachel Sayers	
~	~	Elaine Sizer	
~	~	Amy Skelton	
~	~	Jennifer Smith	
~	~	Sarah Stanley	
~	~	Duncan Stow	
~	~	Abi Stuart	
~	~	Catherine Stuart-Grumbar	
~	~	Matthew Tan	
~	~	Kate Trigg-Hogarth	
~	~	Richard Wallis	
~	~	Alex Williams	
~	~	Sue Wronski	
Chester, UK	Countess of Chester Hospital	Azman Ibrahim	PI
~	~	Mary Aldous	

INVESTIGATORS AND COLLABORATORS: SITE STAFF
Staff on site delegation logs

City	Care_Site	Person_Name	Site_PI
~	~	Ian Allen	
~	~	Denise Archer	
~	~	Wesley Artist	
~	~	Emma Barry	
~	~	Lucy Beresford	
~	~	Kathryn Cawley	
~	~	Lisa Dobson (nee Child)	
~	~	Helen Eccleson	
~	~	Chelcie Faulkner	
~	~	Elizabeth Gallimore	
~	~	Sue Green	
~	~	Rebecca Grogan	
~	~	Jenny Grounds	
~	~	Rebecca Hopcroft	
~	~	Sarah Illingworth	
~	~	Helen Elizabeth Jeffrey	
~	~	Grace McGrath	
~	~	Jenny Miller	
~	~	Judith Prince	
~	~	Shannon Spicer	
~	~	Janet Spriggs	
~	~	Joshua Williams	
Colchester, UK	Colchester General Hospital	Dakshinamoorthy Muthukumar	PI
~	~	Devy Basu	Co-I
~	~	Rana Mahmood	Co-I
~	~	Bruce Sizer	Co-I
~	~	Anita Szita	Co-I
~	~	Katrina Cooke	
~	~	Nicola Cutmore	
~	~	Celine Driscoll	
~	~	Michelle Fisher	
~	~	Richard Gant	
~	~	Hayley Hewer	

INVESTIGATORS AND COLLABORATORS: SITE STAFF

Staff on site delegation logs

City	Care_Site	Person_Name	Site_PI
~	~	Liz Hunting	
~	~	Jane Ketley-O'Donel	
~	~	Muthar Kumar	
~	~	Louies Mabelin	
~	~	Michelle Marshall	
~	~	Sunil Skaria	
~	~	Daisuke Takeuchi	
~	~	Lucy Thorogood	
Colchester, UK	Essex County Hospital	Devy Basu	
~	~	Lorna Dewar	
~	~	Celine Driscoll	
~	~	Hayley Hewer	
~	~	Liz Hunting	
~	~	Jane Ketley-O'Donel	
~	~	Muthar Kumar	
~	~	Michelle Marshall	
~	~	Pugazhenthi Pattu	
~	~	Bruce Sizer	
~	~	Lucy Thorogood	
Cottingham, UK	Castle Hill Hospital	Matthew Simms	PI
~	~	Faheem Bashir	Co-I
~	~	Mohammad Butt	Co-I
~	~	Mohan Hingorani	Co-I
~	~	Mateen Akhtar	
~	~	Ian Beckley	
~	~	Linzi Bone	
~	~	George Bozat	
~	~	Sarah Brown	
~	~	Suzy Bunton	
~	~	Bob Bush	
~	~	Mary Garthwaite	
~	~	Jonathan Gill	
~	~	John Hetherington	

INVESTIGATORS AND COLLABORATORS: SITE STAFF
Staff on site delegation logs

City	Care_Site	Person_Name	Site_PI
~	~	Carol Hodson	
~	~	Linda Hoggarth	
~	~	Louise Karsera	
~	~	Vicki Lowthorpe	
~	~	Jenny Marsden	
~	~	Sarah Moffat	
~	~	Iqtedar Muazzam	
~	~	Paula O'Reilly	
~	~	Sarah Palmer	
~	~	Kristian Plowman	
~	~	Dulani Ranatunge	
~	~	Julie Rawlings	
~	~	Lucy Richardson	
~	~	Karen Stubbs	
~	~	Adam Wolstencroft	
~	~	A Yousuff	
~	~	Khawaje Zahid	
Coventry, UK	Coventry and Warwickshire Hospital	Leila Fortunato	
Coventry, UK	University Hospital Coventry and Warwickshire	Jane Worlding	PI
~	~	Joanna Hamilton	Co-I
~	~	Shah Rafique	Co-I
~	~	Rebecca Aaron	
~	~	Jason Allen	
~	~	Senthil Kumar Athmanathan	
~	~	Rachel Bazeley	
~	~	Maggie Brown	
~	~	Vikki Browne	
~	~	Dannielle Burgess	
~	~	Luanne Carey	
~	~	Andrew Chan	
~	~	Rajbinder Deol	
~	~	Theresa Griffiths	
~	~	Kieran Jefferson	

INVESTIGATORS AND COLLABORATORS: SITE STAFF
Staff on site delegation logs

City	Care_Site	Person_Name	Site_PI
~	~	Mohammed Khan	
~	~	Yakhub Khan	
~	~	Donald Macdonald	
~	~	Fiona McGurk	
~	~	Lucy Miller	
~	~	Albert Mislang	
~	~	Mohamed Mooradun	
~	~	Su Ngwenya	
~	~	Zoe O'Neill	
~	~	Sarah O'Toole	
~	~	Karandeepu Pachoo	
~	~	Sonia Powell	
~	~	Sue Robinson	
~	~	Sukhbinder Salh	
~	~	Noor Ayesha Shah	
~	~	Elaine Simmons	
~	~	Laura Stanley	
~	~	Andrew Stockdale	
~	~	Vicky Sturgess	
~	~	Charlie-marie Suddens	
~	~	Rachel Thompson	
~	~	Fiona Tranter	
~	~	Jenny Warmington	
~	~	Mark Whitmore	
~	~	Linda Wimbush	
Crewe, UK	Leighton Hospital	Anna Tran	PI
~	~	Vanessa Adamson	
~	~	Carole Bennion	
~	~	Kim Best	
~	~	Michael Braun	
~	~	David Butterworth	
~	~	Lydia Buxton	
~	~	Osman Chohan	

INVESTIGATORS AND COLLABORATORS: SITE STAFF
Staff on site delegation logs

City	Care_Site	Person_Name	Site_PI
~	~	William Croxford	
~	~	Thiraviyam Elumalai	
~	~	Leanne Everall	
~	~	Julia Gemmell	
~	~	Sarah Hoswell	
~	~	Adele Hough	
~	~	Chris Hough	
~	~	P Irwin	
~	~	P Javle	
~	~	Taya Jones	
~	~	Tracy Larcombe	
~	~	Carolyn Mansfield	
~	~	Emma Margerum	
~	~	Julie Meir	
~	~	Gemma Nash	
~	~	Andrew Ritchings	
~	~	Rachel Smith	
~	~	Catherine Thompson	
~	~	Sarah Tinsley	
~	~	Caroline Walker	
~	~	James Wylie	
Croydon, UK	Croydon University Hospital	Cheryl Batish	
~	~	Yvonne Campbell	
~	~	Anne Haldeos	
~	~	Ann Payne	
~	~	Jane Thomson	
Darlington, UK	Darlington Memorial Hospital	Mohammed Kagzi	PI
~	~	Rachel Chatt	
~	~	Alison Chilvers	
~	~	Penny Gamble	
~	~	Helen Haley	
~	~	John Hardman	
~	~	Claire Henderson	

INVESTIGATORS AND COLLABORATORS: SITE STAFF
Staff on site delegation logs

City	Care_Site	Person_Name	Site_PI
~	~	Hyder Latif	
~	~	Julia McBride	
~	~	Lorna Morgan	
~	~	Tanmay Mukhopadhyay	
~	~	Richard Nendick	
~	~	Clive Peedell	
~	~	Calum Polwart	
~	~	Steven Pratt	
~	~	Asia Sarwar	
~	~	Jane Shaw	
~	~	Kimberly Stamp	
~	~	Lynsey Stephenson	
~	~	Jonathan Stoddard	
~	~	Fiona Strong	
~	~	John Vickers	
~	~	Susan Wadd	
Dartford, UK	Darent Valley Hospital	Louise Lacey	
Derby, UK	London Road Community Hospital	Kay Bowdler	
~	~	Prabir Chakraborti	
~	~	Debbie Davis	
~	~	Kristina Duggleby	
~	~	Sarah Hare	
~	~	Sarah Hathaway-Lees	
~	~	Heini Jussila	
~	~	Jane Lawrie	
~	~	Wendy Morrisroe	
~	~	Dakshinamoorthy Muthukumar	
~	~	Karen Simmonds	
~	~	Keeley Smith	
~	~	Colin Ward	
Derby, UK	Royal Derby Hospital	Prantik Das	PI
~	~	Wendy Abbott	
~	~	Shahzad Ahmed	

INVESTIGATORS AND COLLABORATORS: SITE STAFF
Staff on site delegation logs

City	Care_Site	Person_Name	Site_PI
~	~	James Aldous	
~	~	Donna Beal	
~	~	Elizabeth Bedford	
~	~	Liz Bedford	
~	~	Helen Beveridge	
~	~	Sathan Boonyaprapa	
~	~	Sonya Bradshaw	
~	~	Louise Brookes	
~	~	Alison Carrick	
~	~	Prabir Chakraborti	
~	~	Josephine Chmiel	
~	~	Caroline Coulson	
~	~	Kiran Das	
~	~	Julie Dockree	
~	~	Charlotte Downes	
~	~	Julie Edmonds	
~	~	Jodie Fitzgerald	
~	~	Aaron Gallagher	
~	~	Marie Ann Goldsworthy	
~	~	Sarah Hare	
~	~	Margaret Harper	
~	~	Gemma Irvine	
~	~	Christopher Kent	
~	~	Sarah Longhurst	
~	~	Fanuel Magaya	
~	~	Peter Mason	
~	~	Alastair McCabe	
~	~	Lucy McCandless	
~	~	Lorraine McDonald	
~	~	Nicole McKee	
~	~	Nicole McKee (nee Isitt)	
~	~	Jennifer Mitchell	
~	~	Wendy Morrisroe	

INVESTIGATORS AND COLLABORATORS: SITE STAFF
Staff on site delegation logs

City	Care_Site	Person_Name	Site_PI
~	~	Thangarajah Mugunthan	
~	~	Dakshinamoorthy Muthukumar	
~	~	Elizabeth Nadin	
~	~	Ajith Gopinathan Nair	
~	~	Pugazhenthi Pattu	
~	~	Ellie Piggott	
~	~	Timothy Podd	
~	~	Ayman Ramadan	
~	~	Gemma Redfern	
~	~	Manni Sandhu	
~	~	Karen Simmonds	
~	~	Virgil Sivoglo	
~	~	Kashmira Subramanian	
~	~	Sarah Taylor	
~	~	Janet Tomlinson	
~	~	Colin Ward	
~	~	Claire Wintle	
~	~	Chris Worth	
~	~	Georgia Wright	
Doncaster, UK	Doncaster Royal Infirmary	Carmel Pezaro	PI
~	~	Virgil Sivoglo	Ex-PI
~	~	Lucy Smith	Co-I
~	~	Jessica Tay	Co-I
~	~	Sharon Ann Allen	
~	~	Mymoona Alzouebi	
~	~	Sarah Brown	
~	~	Barbara Burlace	
~	~	Robert Chadwick	
~	~	Rachel Codling	
~	~	Joanne Derx	
~	~	Ben East	
~	~	Laura Ellis	
~	~	Catherine Ferguson	

INVESTIGATORS AND COLLABORATORS: SITE STAFF
Staff on site delegation logs

City	Care_Site	Person_Name	Site_PI
~	~	Janet Field	
~	~	Alexandra Firth	
~	~	Meredyth Harris	
~	~	Mark Holliday	
~	~	Nicole Jeffcutt	
~	~	Joanne McNally	
~	~	Amy Neal	
~	~	Muneeb Qureshi	
~	~	Janine Smedley (nee McCabe)	
~	~	Jennifer Taylor	
~	~	Deborah Walstow	
~	~	Lisa Warren	
~	~	Nicola Wilkinson	
~	~	Kim Wood	
Dorchester, UK	Dorset County Hospital	Benjamin Masters	PI
~	~	Naveed Afzal	
~	~	Beverley Anderson	
~	~	Stephen Andrews	
~	~	Pauline Ashcroft	
~	~	Piet Bakker	
~	~	Lynn Billett	
~	~	Robert Blegay	
~	~	Laura Bough	
~	~	Sally Breakspear	
~	~	Susan Carr	
~	~	Ananda Chakrabarti	
~	~	Andrew Cornaby	
~	~	Perric Crellin	
~	~	Andrew Gibbins	
~	~	Jackie Gibbins	
~	~	Tracy Glen	
~	~	Josie Goodsell	
~	~	Sarah Horton	

INVESTIGATORS AND COLLABORATORS: SITE STAFF
Staff on site delegation logs

City	Care_Site	Person_Name	Site_PI
~	~	Stephanie Jones	
~	~	Sally Love	
~	~	Louise O'Shea	
~	~	Andrew Rees	
~	~	Simon Sharpe	
~	~	Delia Whiteman	
~	~	Suzy Wignall	
~	~	Sarah Williams	
Dudley, UK	Russells Hall Hospital	Pek Keng-Koh	PI
~	~	Mano Joseph	Co-I
~	~	Joseph Mano	Co-I
~	~	Paul Anderson	
~	~	Joann Atkinson	
~	~	David Edwards	
~	~	Lesley Edwards	
~	~	Lawrence Emtage	
~	~	Irene Gardner	
~	~	Georgi Georgiev	
~	~	Dee Harris	
~	~	Kath Harrow	
~	~	Nadira Jilani	
~	~	Ruckie Kahlon	
~	~	Jayne Kanwar	
~	~	Karen Kanyi	
~	~	Sally Keates-Porter	
~	~	Julie Matthews	
~	~	Heather McClure	
~	~	Emily McDonald	
~	~	Karen McGarry	
~	~	Vanessa Moore	
~	~	Andrew Moores	
~	~	Jenny O'Grady	
~	~	Manesh Patel	

INVESTIGATORS AND COLLABORATORS: SITE STAFF
Staff on site delegation logs

City	Care_Site	Person_Name	Site_PI
~	~	Hayley Pearson	
~	~	Karen Pearson	
~	~	Prakash Ramachandra	
~	~	Ellen Shirley	
~	~	Lucy Smith	
~	~	Sara Smith	
~	~	Lucie Smith (nee Williams)	
~	~	Anna Summerfield	
~	~	Syed Tirmazy	
~	~	Ellie Traverse	
~	~	Angela Watts	
~	~	Abel Zachariah	
Dundee, UK	Ninewells Hospital	Sangeetha Ponnusamy	
Durham, UK	University Hospital of North Durham	Jean Dent	
~	~	Julie Elliot	
~	~	Julie Elliott	
~	~	Jeanette Maughan	
~	~	Rhona McMenemin	
~	~	Lorna Morgan	
~	~	Andrew Parker	
~	~	Dorothy Turnbull	
~	~	Sarah Welsh	
Eastbourne, UK	Eastbourne District General Hospital	Caroline Manetta	PI
~	~	Shelley Baumber	
~	~	Theresa Baumber	
~	~	Duncan Gilbert	
~	~	Prudence Hobbs	
~	~	Joanna Howard	
~	~	Kay Jones-Skipper	
~	~	William Lawrence	
~	~	Lauren McCrisken	
~	~	Fiona McKinna	
~	~	Peter Rimington	

INVESTIGATORS AND COLLABORATORS: SITE STAFF
Staff on site delegation logs

City	Care_Site	Person_Name	Site_PI
~	~	Neville Sharma	
~	~	David Sharp	
~	~	Aspasia Soultati	
~	~	Graham Watson	
~	~	Mark Whitfield	
Edinburgh, UK	Western General Hospital	Duncan McLaren	PI
~	~	Alistair Law	Co-I
~	~	Jahangeer Malik	Co-I
~	~	Richard Allan	
~	~	Claire Arthur	
~	~	Jennifer Baxter	
~	~	Prasad Bollina	
~	~	Tracy Brear	
~	~	Ewan Brown	
~	~	Caroline Bruce	
~	~	Alison Clark	
~	~	Ann Cochrane	
~	~	Heather Dalrymple	
~	~	Martin Doak	
~	~	Roland Donat	
~	~	Lisa Egan	
~	~	Ben Elliott	
~	~	Olvsola Faluyi	
~	~	Susan Forman	
~	~	Fiona Gardiner	
~	~	Nikki Gilluley	
~	~	Lynn Ho	
~	~	Grahame Howard	
~	~	Heather Howie	
~	~	David Jeffrey	
~	~	Emma Lewis	
~	~	Ailsa Liddle	
~	~	Hannah Lord	

INVESTIGATORS AND COLLABORATORS: SITE STAFF

Staff on site delegation logs

City	Care_Site	Person_Name	Site_PI
~	~	Sanjana Masinghe	
~	~	Barbara Mayne	
~	~	John McGrane	
~	~	Alison McKinlay	
~	~	Alan McNeill	
~	~	Heather McVicars	
~	~	Hazel Milligan	
~	~	Beverley Mitchell	
~	~	Kirsty Peebles	
~	~	Lois Pollock	
~	~	Brian Rogers	
~	~	Fionagh Ross	
~	~	Theresa Savage	
~	~	Andrea Stanton	
~	~	Mark Stares	
~	~	Sarah Thompson	
~	~	David Tulloch	
~	~	Vivienne Wilson	
~	~	Katie Wood	
~	~	Catherine Woods	
Edmonton, UK	North Middlesex Hospital	Nishi Gupta	PI
~	~	Chris Abbott	
~	~	Beatrice Balachandran	
~	~	Girish Bhome	
~	~	Debbie Blois	
~	~	Tom Caumont	
~	~	Bernadette Collins	
~	~	Judy Hill	
~	~	Lorraine Hurl	
~	~	Stephen Karp	
~	~	Ursula McGovern	
~	~	Lucinda Melcher	
~	~	Farhad Neave	

INVESTIGATORS AND COLLABORATORS: SITE STAFF
Staff on site delegation logs

City	Care_Site	Person_Name	Site_PI
~	~	Jackie Newby	
~	~	Kathy O'Farrell	
~	~	Asim Ray	
~	~	Kerri Rees	
~	~	Mausam Singhera	
~	~	Ferrial Syed	
~	~	Anna Thompson	
~	~	Chloe Van Someren	
Exeter, UK	Royal Devon and Exeter Hospital	Denise Sheehan	PI
~	~	San Aung	Co-I
~	~	Rajaguru Srinivasan	Co-I
~	~	Peter Stephens	Co-I
~	~	John Anderson	
~	~	Alison Augstburger	
~	~	Kizzy Baines	
~	~	Alan Betts	
~	~	David Jonathan Chambers	
~	~	Tamika Chapter	
~	~	Ross Curwen	
~	~	Susan Davenport	
~	~	Elizabeth Davey	
~	~	Melissa Davey	
~	~	Susan Downer	
~	~	Dawn Edwards	
~	~	Stephanie Ann Ellis	
~	~	Victoria Ford	
~	~	Tracey Foss	
~	~	Emma Guerin	
~	~	Anne Hong	
~	~	Frances Hood	
~	~	Beverley Kemp	
~	~	Theresa Lawless	
~	~	James Leavy	

INVESTIGATORS AND COLLABORATORS: SITE STAFF
Staff on site delegation logs

City	Care_Site	Person_Name	Site_PI
~	~	Christoph Lohan	
~	~	Anna Lydon	
~	~	Lyndel Moore	
~	~	Ayman Nassar	
~	~	Tim Norris	
~	~	Kate O'Connor	
~	~	Jane Piper	
~	~	Claire Ridler	
~	~	Alison Roantree	
~	~	Emma Robjohns	
~	~	Ingrid Seath	
~	~	Suzy Tasker	
~	~	Shirley Todd	
~	~	Elizabeth Toy	
~	~	Matt Trivett	
~	~	Elaine Vandcandelaere	
~	~	Fiona Walters (nee Hall)	
~	~	Sophie Warren	
~	~	Claire Webb	
Gillingham, UK	Medway Maritime Hospital	Stergios Boussios	PI
~	~	Henry Taylor	Ex-PI
~	~	Charlotte Abson	Co-I
~	~	Christos Mikropoulos	Co-I
~	~	Khalid Abdalla	
~	~	Philip Adeniran	
~	~	Diletta Bianchini	
~	~	Louise Black	
~	~	Corinne Borley	
~	~	Louise Brassington	
~	~	Deirdre Cooke	
~	~	Parool Darbar	
~	~	Charles Davis	
~	~	Tamara Diamond	

INVESTIGATORS AND COLLABORATORS: SITE STAFF
Staff on site delegation logs

City	Care_Site	Person_Name	Site_PI
~	~	Mary Everett	
~	~	Durga Maya Gurung	
~	~	Marie Louise Hollands	
~	~	Kay Jones	
~	~	Afroditi Karathanasi	
~	~	Tessa Lawrence	
~	~	Carol Mayger	
~	~	Peter Milverton	
~	~	Kevin Naicker	
~	~	Elizabeth Newman-Horne	
~	~	Lisa Parker	
~	~	Suzie Reyner	
~	~	Alison Richards	
~	~	Agne Sadauskaite	
~	~	James Sawyer	
~	~	Jodie Seymour	
~	~	Nicola Southwell	
~	~	Emma Sutton	
~	~	Swapna Thomas	
~	~	Richard Thornton	
~	~	Alba Tuban	
~	~	Katarzyna Urbanczyk	
~	~	Gayzel Vallejera	
~	~	Simon Wan	
Glasgow, UK	Beatson West of Scotland Cancer Centre	Rob Jones	PI
~	~	John Graham	Ex-PI
~	~	Kathryn Banfill	Co-I
~	~	Derek Grose	Co-I
~	~	Carolynn Lamb	Co-I
~	~	Tareq Abdullah	
~	~	Abdulla Al-hasso	
~	~	Mohammed Alfayez	
~	~	Jawaher Ansari	

INVESTIGATORS AND COLLABORATORS: SITE STAFF

Staff on site delegation logs

City	Care_Site	Person_Name	Site_PI
~	~	Miranda Ashton	
~	~	Patricia Baird	
~	~	Martin Ball	
~	~	Gillian Barmack	
~	~	Sophie Barrett	
~	~	Lorraine Barwell	
~	~	Karen Bell	
~	~	Jenny Brown	
~	~	Louise Bruce	
~	~	Nicola Cairns	
~	~	Ross Carruthers	
~	~	Almudena Cascales	
~	~	Annette Charlick	
~	~	Maureen Connolly	
~	~	Catriona Cowan	
~	~	Alice Coy	
~	~	Cicely Cunningham	
~	~	Judith Dixon	
~	~	David Dodds	
~	~	Gerard Forrest	
~	~	Ben Fulton	
~	~	Katie Galbraith	
~	~	Hilary Glen	
~	~	Jacqueline Gourlay	
~	~	Jan Graham	
~	~	Janet Graham	
~	~	Kathryn Graham	
~	~	Lynne Grieve	
~	~	Ailsa Griffen	
~	~	Sally Hall	
~	~	Maureen Hamill	
~	~	Maryon Hardie	
~	~	Paula Henry-Stephenson	

INVESTIGATORS AND COLLABORATORS: SITE STAFF

Staff on site delegation logs

City	Care_Site	Person_Name	Site_PI
~	~	Awris Jalil	
~	~	Sai Juan Jia	
~	~	Gemma Johnson	
~	~	Saranya Kakumanu	
~	~	Ashleigh Kerr	
~	~	Esfandiyar Khan	
~	~	Kirsten Laws (nee Borthwick)	
~	~	Graeme Lumsden	
~	~	Antonia MacMillan	
~	~	Nicholas Macleod	
~	~	Rana Mahmood	
~	~	Husam Marashi	
~	~	Brendan McCann	
~	~	Stephen McKay	
~	~	Fiona McQueen	
~	~	Rebecca Muirhead	
~	~	Maria Nicol	
~	~	Stefan Nowicki	
~	~	Ruth Orr	
~	~	Aqilah Othman	
~	~	Jennifer Petrie	
~	~	Linzi Rae	
~	~	Nathan Richardson	
~	~	Patricia Roxburgh	
~	~	Martin Russell	
~	~	Azmat Sadozye	
~	~	Ian Sanders	
~	~	Norma Sidek	
~	~	Claire Steele	
~	~	Kirsteen Stuart	
~	~	Diann Taggart	
~	~	Lye Mun Tho	
~	~	Aisha Tufail	

INVESTIGATORS AND COLLABORATORS: SITE STAFF

Staff on site delegation logs

City	Care_Site	Person_Name	Site_PI
~	~	Balaji Venugopal	
~	~	Jan Wallace	
~	~	Hannah Weir	
~	~	Christina Wilson	
Gloucester, UK	Gloucestershire Royal Hospital	Jo Bowen	PI
~	~	Peter Jenkins	Co-I
~	~	Julie Allen	
~	~	Charlotte Ayrton	
~	~	Sarah Beazer	
~	~	Victoria Bell	
~	~	Bethan Cartwright	
~	~	Audrey Cook	
~	~	Samuel Croly	
~	~	Lin Crossley	
~	~	Chris Ford	
~	~	Janet Forkes	
~	~	Julia Hall	
~	~	Sai Jonnada	
~	~	Laura Malins	
~	~	Sarah Matthews	
~	~	Louise Moore	
~	~	Roger Owen	
~	~	Elisabeth Read	
~	~	Claire Salter	
~	~	Rachel Sayers	
~	~	Elaine Sizer	
~	~	Amy Skelton	
~	~	Sarah Stanley	
~	~	Abi Stuart	
~	~	Kate Trigg-Hogarth	
~	~	Richard Wallis	
~	~	Sue Wronski	
Guildford, UK	Royal Surrey County Hospital	Carla Perna	PI

INVESTIGATORS AND COLLABORATORS: SITE STAFF
Staff on site delegation logs

City	Care_Site	Person_Name	Site_PI
~	~	Leslie Cheng	Co-I
~	~	Mahwish Karim	Co-I
~	~	Richmond Abeseabe	
~	~	Kavita Bhat	
~	~	Caterina Bissa	
~	~	Melanie Boafo-Yirenkyi	
~	~	Fiona Butler	
~	~	Marianne Dabbs	
~	~	Veronica Davis	
~	~	Sarah De Swert	
~	~	Maria Drzymala	
~	~	Daisy Floyd	
~	~	Teresa Guerrero-Urbano	
~	~	Lesley Harden	
~	~	Celia Harris	
~	~	Imogen Heenan	
~	~	Adele Hugg	
~	~	Stephy Joseph	
~	~	Jen Julius	
~	~	Teresa Keating	
~	~	Sara Khaksar	
~	~	Zephyrine King	
~	~	Robert Laing	
~	~	Emmanuel Larbi	
~	~	James Lowe	
~	~	Catherine Medcalf	
~	~	Julian Money-Kyrle	
~	~	Mahomed Moosa	
~	~	Angela Morgan	
~	~	Linda Nardone	
~	~	Kathrin Narvaez-Vega	
~	~	Jenny Nobes	
~	~	Kate Penhaligon	

INVESTIGATORS AND COLLABORATORS: SITE STAFF
Staff on site delegation logs

City	Care_Site	Person_Name	Site_PI
~	~	Nick Pilkington	
~	~	Sue Sargent	
~	~	Richard Shaffer	
~	~	Charlotte Shelley	
~	~	Frances Sidi	
~	~	Joanna Stokoe	
~	~	Sree Susaria	
~	~	Miriam White	
~	~	Julia Whittle	
~	~	Katie Wood	
~	~	Jane Woods	
Harlow, UK	Princess Alexandra Hospital (Harlow)	Lucinda Melcher	PI
~	~	Tasia Aghadiuno	Co-I
~	~	Nishi Gupta	Co-I
~	~	Anna Lerner	Co-I
~	~	Hamoun Rozati	Co-I
~	~	Zainab Wasim	Co-I
~	~	Gemma Cook	
~	~	Amelia Daniel	
~	~	Reena Davda	
~	~	Shroma De Silva	
~	~	Albert Edwards	
~	~	Sunjalee Fernando	
~	~	Ahmed Hnoosh	
~	~	Evelyn Holmes	
~	~	Jodie Johnson	
~	~	Paul Kabuubi	
~	~	Joanne Kellaway	
~	~	Amanda Lewis	
~	~	Amy Lewis	
~	~	Teresa Light	
~	~	Cait Rees	
~	~	Ervin Shpuza	

INVESTIGATORS AND COLLABORATORS: SITE STAFF
Staff on site delegation logs

City	Care_Site	Person_Name	Site_PI
~	~	Tracey White	
~	~	Nikki White (nee Staines)	
Haverford West, UK	Withybush General Hospital	Sandra Evens	
Hereford, UK	Hereford County Hospital	Warren Grant	PI
~	~	Susan Anderson	
~	~	Anita Ashton	
~	~	Vishal Bhalla	
~	~	Caitlin Bowden	
~	~	Sophie Boyd	
~	~	Sophie Boyd (nee Evans)	
~	~	Serrafina Carini	
~	~	Jagdish Chana	
~	~	Audrey Cook	
~	~	Sophie Cooper	
~	~	Melanie Evans	
~	~	Maxine Flubacher	
~	~	Janet Forkes	
~	~	Kate Hammerton	
~	~	Andy Hedges	
~	~	Gill Horsfield	
~	~	Jenny Howls	
~	~	Claire Hughes	
~	~	Janine Jones (Birch)	
~	~	Lisa King	
~	~	Laura Lees	
~	~	Rachel Lowe	
~	~	Linda Moseley	
~	~	Naeem Musani	
~	~	Jolanta Pueskacz	
~	~	Catherine Reed	
~	~	Nina Reeve	
~	~	Zara Roberts	
~	~	Timothy Spencer	

INVESTIGATORS AND COLLABORATORS: SITE STAFF

Staff on site delegation logs

City	Care_Site	Person_Name	Site_PI
~	~	David Stow	
~	~	Duncan Stow	
~	~	Harriet Taylor	
~	~	June Thomas	
~	~	Stacey Turner	
~	~	Cara Watson	
~	~	Terry Watson	
~	~	Bethany Wellington	
~	~	Nicola Williamson	
High Wycombe, UK	Wycombe Hospital	Katherine Hyde	PI
~	~	Ami Sabharwal	Ex-PI
~	~	Gerard Andrade	Co-I
~	~	Philip Camilleri	Co-I
~	~	Prabir Chakraborti	Co-I
~	~	Sean O'Cathail	Co-I
~	~	Thinn Pwint	Co-I
~	~	Maggie Aldersley	
~	~	Bhavna Badiani	
~	~	Jasvinder Bains	
~	~	Amarjit Bdesha	
~	~	Ans-Mari Bester	
~	~	Nicola Bowers	
~	~	Chrissie Butcher	
~	~	Janice Carpenter	
~	~	Penny Carter	
~	~	Evelyn Chan	
~	~	Tiffany Chan	
~	~	Christine Collins	
~	~	Anita Cserbane	
~	~	Benjamin Fairfax	
~	~	Claire Fernandez	
~	~	Siobhan Gettings	
~	~	Avinash Gupta	

INVESTIGATORS AND COLLABORATORS: SITE STAFF

Staff on site delegation logs

City	Care_Site	Person_Name	Site_PI
~	~	Neil Haldar	
~	~	Kathryn Herbert	
~	~	Emma Hogbin	
~	~	Manisha Joshi	
~	~	Roisin Kavanagh	
~	~	John Patrick Kelleher	
~	~	Rahul Kurup	
~	~	Erica Lieberman	
~	~	Rossana Mancinelli	
~	~	Sarah Manyangadze	
~	~	Moncy Mathew	
~	~	Susan McLain-Smith	
~	~	Vivek Mohan	
~	~	Aruna Nair	
~	~	Alice Ngumo	
~	~	Ileana Nguyen	
~	~	Catherine Northey	
~	~	Niki Panakis	
~	~	Andrew Protheroe	
~	~	Wasiru Saka	
~	~	Tracey Stammers	
~	~	Helena Stone	
~	~	Michelle Taylor-Siddons	
~	~	Samantha Thomas	
~	~	Sally Trent	
~	~	Neil Trew-Smith	
~	~	Gail Varley	
~	~	Janet Weir	
~	~	Hazel Wynn	
Huddersfield, UK	Huddersfield Royal Infirmary	Uschi Hofmann	PI
~	~	Nicolas Bryan	Co-I
~	~	Lucy Jones	Co-I
~	~	Deivasikamani Ramanujam	Co-I

INVESTIGATORS AND COLLABORATORS: SITE STAFF
Staff on site delegation logs

City	Care_Site	Person_Name	Site_PI
~	~	Samantha Turnbull	Co-I
~	~	Mohammad Irfan Alam	
~	~	Karen Bicknell	
~	~	Barbara Crosse	
~	~	Nicky Daker	
~	~	Stacey Freeth	
~	~	Lisa Gledhill	
~	~	Paula Gomes	
~	~	Lindsay Greenhalgh	
~	~	Denise Hancock	
~	~	Jane Hook	
~	~	Ibrar Hussain	
~	~	Hayley Inman	
~	~	Diane Kelly	
~	~	Mandy Madigan	
~	~	Lear Matapure	
~	~	Adam Mawer	
~	~	Belinda McLean	
~	~	Julie Millward	
~	~	Naledi Mzwimbi	
~	~	Monica Narasimham	
~	~	Rachel Parker	
~	~	Melanie Quesne	
~	~	Hannah Riley	
~	~	Kully Sandhu	
~	~	Lisa Shaw	
~	~	Kathryn Smith	
~	~	Katherine Tighe	
~	~	Christine Turner	
~	~	Rob Turner	
~	~	Miranda Usher	
~	~	Hayley Webster	
~	~	Tracy Wood	

INVESTIGATORS AND COLLABORATORS: SITE STAFF

Staff on site delegation logs

City	Care_Site	Person_Name	Site_PI
~	~	Emma Woodward	
~	~	Sharon Woolley	
Hull, UK	Princess Royal Hospital (Hull)	Robert Dealey	Ex-PI
~	~	Emma Bertram	
~	~	Suzy Bunton	
~	~	Christopher Hamilton	
~	~	Linda Hoggarth	
~	~	Claire Levesley	
~	~	Sarah Moffat	
Inverness, UK	Raigmore Hospital	Neil McPhail	PI
~	~	Anglise Addison	
~	~	Florence Anderson	
~	~	Seonaid Arnott	
~	~	Susan Bain	
~	~	Sudhir Borgaonkar	
~	~	Sandra Brown	
~	~	Karen Callum	
~	~	Audrey Campbell	
~	~	Denise Campbell	
~	~	Fiona Campbell	
~	~	Jane Campbell	
~	~	Margaret Chisholm	
~	~	Kay Kelly	
~	~	Charles Kodikara	
~	~	Michael Loynd	
~	~	Alison Macdonald	
~	~	Angela Macgregor	
~	~	Carol Macgregor	
~	~	Rachel Mackay	
~	~	Laura Maclennan	
~	~	Jude Madeleine	
~	~	Melanie McIlroy	
~	~	Mary McKenzie	

INVESTIGATORS AND COLLABORATORS: SITE STAFF
Staff on site delegation logs

City	Care_Site	Person_Name	Site_PI
~	~	Karina McQuiston	
~	~	Catriona Morrison	
~	~	Sean Neville	
~	~	Alison Nicholls	
~	~	Steve Nicholson	
~	~	Aristoula Papakostidi	
~	~	Marion Paterson	
~	~	Anne Marie Pollock	
~	~	Martin Russell	
~	~	Azmat Sadozye	
~	~	Ian Shread	
~	~	Georgina Simpson	
~	~	Glenda Sinclair	
~	~	Jane Sinclair	
~	~	Anna Skene	
~	~	Joan Stewart	
~	~	Una Taylor	
~	~	Zoe Urquhart	
~	~	David Whillis	
Ipswich, UK	Ipswich Hospital	Robert Brierly	PI
~	~	William Ine	Co-I
~	~	TJ Podd	Co-I
~	~	Deborah Abrams	
~	~	Debbie Austin	
~	~	Gautam Banerjee	
~	~	Sheen Cherian	
~	~	Jennifer Collins	
~	~	Peter Donaldson	
~	~	Charlotte Etheridge	
~	~	Ian Floodgate	
~	~	Mohsen Habib	
~	~	Adiba Hoodbhoy	
~	~	Kerry Howlett (nee Brown)	

INVESTIGATORS AND COLLABORATORS: SITE STAFF
Staff on site delegation logs

City	Care_Site	Person_Name	Site_PI
~	~	Sonia Kerridge	
~	~	Natalie Lloyd	
~	~	Matt Mendoza	
~	~	John Parry	
~	~	Paul Ridley	
~	~	Mandy Riley (nee Evans)	
~	~	Chris Rose	
~	~	Christopher Scrase	
~	~	Julie Simpson	
~	~	Julie Spurgeon	
~	~	Sarah Treece	
~	~	Yvonne Tricker	
~	~	Susan Upson	
~	~	Ramachandran Venkitaraman	
~	~	Joe Wells	
~	~	Angharad Williams	
~	~	Jo Woor	
Keighley, UK	Airedale General Hospital	Simon Brown	PI
~	~	Sohail Mughal	Co-I
~	~	Hayley Bates	
~	~	Louise Binns	
~	~	Carl Booth	
~	~	Lisa Bullough	
~	~	Nathalie Casanova	
~	~	Sue Cheeseman	
~	~	Katy Clarke	
~	~	Michael Crawford	
~	~	Gillian Darnbrook	
~	~	Fiona Farquhar	
~	~	Andrew Gash	
~	~	Jasmine Hartley	
~	~	Ann Henry	
~	~	Helen Henson	

INVESTIGATORS AND COLLABORATORS: SITE STAFF
Staff on site delegation logs

City	Care_Site	Person_Name	Site_PI
~	~	Pip Hill	
~	~	Ganesan Jeyasangar	
~	~	Ruth Johnson	
~	~	Rachel Kennedy	
~	~	Dan Lee	
~	~	Judy McAlister	
~	~	Sharron Parkinson	
~	~	Amy Pendrill	
~	~	Joseph Quinn	
~	~	Charlotte Richardson	
~	~	Satti Saggu	
~	~	Clara Sentamans	
~	~	Alison Shaw	
~	~	Liz Shenton	
~	~	Josie Snell	
~	~	Mandy Swanepoel	
~	~	Alison Swindells	
Kidderminster, UK	Kidderminster General Hospital	Lisa Capaldi	PI
~	~	Kirsty Clarke	Co-I
~	~	Paul Flinders	Co-I
~	~	Ayyaz Munawar	Co-I
~	~	Shaikh Rana	Co-I
~	~	Mark Churn	
~	~	Kate Field	
~	~	Monica Gauntlett	
~	~	Linda Higgins	
~	~	Hayley Hodson	
~	~	M Habib Khan	
~	~	Emma Marshall	
~	~	Hugh Morrow	
~	~	Sarah Moss	
~	~	Zeeshaan Parvez	
~	~	Patricia Rimell	

INVESTIGATORS AND COLLABORATORS: SITE STAFF
Staff on site delegation logs

City	Care_Site	Person_Name	Site_PI
~	~	Alison Rosoman	
~	~	Veronica Rowlands	
~	~	Sally Stringer (pr. Davis)	
~	~	Helen Tranter	
~	~	Jayne Tyler	
~	~	Ann White	
~	~	Julie Wollaston	
Kilmarnock, UK	Crosshouse Hospital	Margaret McKernan	
Lancaster, UK	Royal Lancaster Infirmary	Sophie Raby	
Larbert, UK	Forth Valley Royal Hospital	Norma Sidek	PI
~	~	Saurabh Borgaonkar	
~	~	Stephanie Brogan (nee Roddie)	
~	~	Maureen Hamill	
~	~	Eilidh Henderson	
~	~	Carolynn Lamb	
~	~	Amy Martin	
~	~	Stephen McKay	
~	~	Nadja Melquiot	
~	~	Adam Peters	
~	~	Lynn Prentice	
~	~	Joanne Robinson	
~	~	John Martin Russell	
~	~	Lesley Symon	
~	~	Seamus Teahan	
~	~	Anne Todd	
~	~	Patricia Turner	
~	~	Sally Young	
~	~	Alison Yule	
Leeds, UK	Cookridge Hospital	Richard Kaplan	
~	~	Anne Kiltie	
~	~	Carmel Loughrey	
Leeds, UK	Leeds General Infirmary	Caroline Bedford	
~	~	Adrian Joyce	

INVESTIGATORS AND COLLABORATORS: SITE STAFF
Staff on site delegation logs

City	Care_Site	Person_Name	Site_PI
Leeds, UK	St James University Hospital (Leeds)	William Cross	PI
~	~	Peter Whelan	Ex-PI
~	~	Naveen Vasudev	Co-I
~	~	Dolapo Ajayi	
~	~	Polapo Ajayi	
~	~	Javeria Akhtar	
~	~	Gemma Austin (nee Glover)	
~	~	Caroline Bedford	
~	~	Ian Boon	
~	~	David Bottomley	
~	~	Janet Brown	
~	~	James Cavanagh	
~	~	Judith Chapman	
~	~	John Chester	
~	~	Jude Clarke	
~	~	Anne Crossley	
~	~	Claire Daisey	
~	~	Luis Daverede	
~	~	Emily Davies	
~	~	Svetoslava Doshmanonska	
~	~	Judith Evans	
~	~	Kevin Franks	
~	~	Catherine Gray	
~	~	Maria Hall	
~	~	Ann Henry	
~	~	Jodene Hill	
~	~	Liz Hudson	
~	~	Satinder Jagdev	
~	~	Sunjay Jain	
~	~	Joseph Joji	
~	~	Adrian Joyce	
~	~	Mercy Kaiga	
~	~	Richard Kaplan	

INVESTIGATORS AND COLLABORATORS: SITE STAFF
Staff on site delegation logs

City	Care_Site	Person_Name	Site_PI
~	~	Richard Khafagy	
~	~	Anne Kiltie	
~	~	Sanjeev Kotwal	
~	~	Sam Lotfi	
~	~	Carmel Loughrey	
~	~	Emma Lundy	
~	~	Jade McCann	
~	~	Angela Morgan	
~	~	Hima Bindu Musunuru	
~	~	Catherine Parbutt	
~	~	Alan Paul	
~	~	Helen Payne	
~	~	Charlotte Pool	
~	~	Stephen Prescott	
~	~	Christy Ralph	
~	~	Hannah Roberts	
~	~	Sue Rodwell	
~	~	Krishna Shastry	
~	~	Sue Sibson	
~	~	Rafal Turo	
~	~	Hannah Wigginton	
~	~	Christopher Williams	
~	~	Lorraine Wiseman	
~	~	Ruiyang Yan	
Lincoln, UK	Lincoln County Hospital	Thiagarajan Sreenivasan	PI
~	~	Prantik Das	Co-I
~	~	Ana Fernandez-Ots	Co-I
~	~	Sindhu Ramarwothy	Co-I
~	~	Alfredo Addeo	
~	~	Simon Archer	
~	~	Suzanne Archer	
~	~	Christian Arias	
~	~	David Ballesteros-Quintail	

INVESTIGATORS AND COLLABORATORS: SITE STAFF

Staff on site delegation logs

City	Care_Site	Person_Name	Site_PI
~	~	Giuseppe Banna	
~	~	Karin Baria	
~	~	Sarah Bell	
~	~	Jayne Borley	
~	~	Susie Butler	
~	~	Diane Carey	
~	~	Helen Carolan	
~	~	Sarah Coombs	
~	~	Olesya Francis	
~	~	Annette Hilldrith	
~	~	Kathryn Hoare	
~	~	Kerri Johnson	
~	~	Andrew Judd	
~	~	Carol Lockwood	
~	~	Ray McDermott	
~	~	Yogesh Nishchal	
~	~	Maryanne Okubanjo	
~	~	Miguel Panades	
~	~	Kathryn Pearson	
~	~	Rhiannan Pegg	
~	~	Gunjan Phalod	
~	~	Jenny Salmon	
~	~	Andrew Sloan	
~	~	Rebecca Spencer	
~	~	Caroline Taylor	
~	~	Janet Tomlinson	
~	~	Elena Umbrarescu	
~	~	Laura Walsh	
~	~	Alyson Wilson	
Liverpool, UK	Royal Liverpool University Hospital	Zafar Malik	PI
~	~	Chinnamani Eswar	Co-I
~	~	Nicola Bermingham	
~	~	Lizzie Dale	

INVESTIGATORS AND COLLABORATORS: SITE STAFF
Staff on site delegation logs

City	Care_Site	Person_Name	Site_PI
~	~	Lynsey Dean	
~	~	Lisa Dobson (nee Child)	
~	~	Sharon Dunn (nee Johnson)	
~	~	Sue Green	
~	~	Julie Griffiths	
~	~	Paul Griffiths	
~	~	Jasima Latif	
~	~	Kevin McDonald	
~	~	Pauline Pilkington	
~	~	Dawn Porter	
~	~	Philip Reynolds	
~	~	Sandra Robinson	
~	~	Peter Robson	
~	~	Nidhi Sibal	
~	~	Katy Treherne	
~	~	Emma Whitby	
~	~	Pembe Yesildag	
Liverpool, UK	University Hospital Aintree	Peter Robson	PI
~	~	Ian Allen	
~	~	Wesley Artist	
~	~	Lucy Berresford	
~	~	Lisa Dobson (nee Child)	
~	~	Rachael Fergusson	
~	~	Julie Griffiths	
~	~	Paul Hill	
~	~	Lorraine Lancaster	
~	~	Haley McCulloch	
~	~	Leigh Pauls	
~	~	Sandra Robinson	
London, UK	Charing Cross Hospital	Alison Falconer	PI
~	~	Stephen Mangar	Co-I
~	~	Najma Ahmed	
~	~	Kwame Ansu	

INVESTIGATORS AND COLLABORATORS: SITE STAFF
Staff on site delegation logs

City	Care_Site	Person_Name	Site_PI
~	~	Gareth Barker	
~	~	Bindu Chikkamuniyappa	
~	~	Ross Dalton-Short	
~	~	Andrea Davis-Cook	
~	~	Steve Edwards	
~	~	Daisy Floyd	
~	~	Jill Gallagher	
~	~	Paul Kabuubi	
~	~	Zohanon Sabine Loko	
~	~	Ethna Mannion	
~	~	Akeema Paul	
~	~	Ibiyemi Sadare (Olaleye)	
~	~	Naveed Sarwar	
~	~	Stephanie Steadman	
~	~	Samantha Weller	
London, UK	Guy's Hospital (London)	Sarah Rudman	PI
~	~	Sarah Howiett	Co-I
~	~	Vishal Manik	Co-I
~	~	Chara Stavraka	Co-I
~	~	Awo Abdi	
~	~	Delali Adjogatse	
~	~	Ajay Aggarwal	
~	~	Fahim Ahmed	
~	~	Rayhan Ahmed	
~	~	Ramin Ajami	
~	~	Susanne Allan	
~	~	Stephanie Argue	
~	~	Caterina Aversa	
~	~	Eva Batovska	
~	~	Ronald Beaney	
~	~	Thomas Bird	
~	~	Trevor Bott	
~	~	Sabeeh Butt	

INVESTIGATORS AND COLLABORATORS: SITE STAFF

Staff on site delegation logs

City	Care_Site	Person_Name	Site_PI
~	~	Declan Cahill	
~	~	Jozer Calara	
~	~	Donna Cassidy	
~	~	Emilia Caverly	
~	~	Charleen Chan Wah Hak	
~	~	Belinda Chitando	
~	~	Simon Chowdhury	
~	~	Chi Yee Chung	
~	~	Sharon Clovis	
~	~	Danielle Crawley	
~	~	Francesca Curran	
~	~	Kafui Dossa	
~	~	Michelle Dutton	
~	~	Deborah Enting	
~	~	Louisa Fleure	
~	~	Angel Garcia-Imhof	
~	~	Tahereh Ghadimi	
~	~	Sharmistha Ghosh	
~	~	Clare Gilson	
~	~	Claire Glendon	
~	~	Charalampos Gousis	
~	~	Teresa Guerrero-Urbano	
~	~	Sarah Hargreaves	
~	~	Peter Harper	
~	~	Simon Hughes	
~	~	Sheeba Irshad	
~	~	Ruth Johnson	
~	~	Eleni Josephides	
~	~	Debra Josephs	
~	~	Lucy Juggins	
~	~	Srivani Kandasamy	
~	~	Matthaius Kapiris	
~	~	Anna Karpathakis	

INVESTIGATORS AND COLLABORATORS: SITE STAFF
Staff on site delegation logs

City	Care_Site	Person_Name	Site_PI
~	~	Muhammad Khan	
~	~	Rosalind Kieran	
~	~	Sarah King	
~	~	Ursula Kirwan	
~	~	Lawrence Krieger	
~	~	Hartmut Kristeleit	
~	~	Cheryl Lawrence	
~	~	Archie Macnair	
~	~	Thubeena Manickavasagar	
~	~	Louisa McDonald	
~	~	Sharon McPherson	
~	~	Vasiliki Michalarea	
~	~	Stephen Morris	
~	~	Vinod Mullassery	
~	~	Ngozi Muoneke	
~	~	Janette Nichol	
~	~	Emma O'Connor	
~	~	Temi Olusi	
~	~	Anna Parker	
~	~	Elias Pintus	
~	~	Rick Popert	
~	~	Vivien Quan	
~	~	Antonio Querol-Rubiera	
~	~	Lucy Reed	
~	~	Philip Reynolds	
~	~	Catherine Rogers	
~	~	Hannah Rush	
~	~	Linda Shephard	
~	~	Sumeet Sisodia	
~	~	Susie Slater	
~	~	Helen Snow	
~	~	Anita Soma	
~	~	Thomas Spencer	

INVESTIGATORS AND COLLABORATORS: SITE STAFF
Staff on site delegation logs

City	Care_Site	Person_Name	Site_PI
~	~	Rushan Sylva	
~	~	Kiruthikah Thillai	
~	~	Rebecca Todd	
~	~	Daniel Tong	
~	~	Gerry Trillana	
~	~	Eirini Tsotra	
~	~	Nikolaos Tsoukalas	
~	~	Jennifer Turner	
~	~	Suzanne Vizor	
~	~	Mark Voskoboynik	
~	~	Sally Walker	
~	~	Rebecca Way	
~	~	Kate Williams	
~	~	Yin Wu	
~	~	Kamarul Zaki	
London, UK	Hammersmith Hospital	Alison Falconer	PI
~	~	Stephen Mangar	Co-I
~	~	Ilyas Ali	
~	~	Steve Edwards	
~	~	Nikki Kettley	
~	~	Emily Pickford	
~	~	Regina Storch	
London, UK	King George Hospital	Neil Fisher	
~	~	Ramachandran Subramaniam	
London, UK	Queen Elizabeth Hospital (Woolwich)	Sindu Vivekanandan	PI
~	~	Vinod Mullessey	Ex-PI
~	~	Vinod Mullassery	Co-I
~	~	Elias Pintus	Co-I
~	~	Rayhan Ahmed	
~	~	Shahreen Ahmed	
~	~	Jagdev Bains	
~	~	Laura Beschizza	
~	~	Belinda Chitando	

INVESTIGATORS AND COLLABORATORS: SITE STAFF
Staff on site delegation logs

City	Care_Site	Person_Name	Site_PI
~	~	Sharai Chitando	
~	~	Suzanne Chukundah	
~	~	Miriam Cottle	
~	~	Nadia El-Sayed	
~	~	Martha Handousa	
~	~	Rachel Harper	
~	~	Hazel Harrop	
~	~	Nigel Holmes	
~	~	Simon Hughes	
~	~	Abhijit Jadhav	
~	~	Abel Jalloh	
~	~	Bridget Kabagambe	
~	~	Arunansu Kar	
~	~	Sagira Khatun	
~	~	Hartmut Kristeleit	
~	~	Maria Liskova	
~	~	Luke Maidment	
~	~	Nick Maisey	
~	~	Joyce Maravi	
~	~	Jennifer Martin	
~	~	Theodorah Nago	
~	~	Melody Ncube	
~	~	Eti Omoregie	
~	~	Samia Pilgrim	
~	~	Lee Porin	
~	~	Philip Reynolds	
~	~	Thomas Sarkodie	
~	~	Aarti Shah	
~	~	Anne-Marie Vindidu	
~	~	Shanna Wilson	
London, UK	Royal Free Hospital	Sarah Needleman	PI
~	~	Maria Vilarino-Varela	Ex-PI
~	~	Magdalena Kubiak	Co-I

INVESTIGATORS AND COLLABORATORS: SITE STAFF
Staff on site delegation logs

City	Care_Site	Person_Name	Site_PI
~	~	Nicola Rosenfelder	Co-I
~	~	Emily Scott	Co-I
~	~	Kate Smith	Co-I
~	~	Grant Stewart	Co-I
~	~	Naomi Anderson	
~	~	Juniebel Cooke	
~	~	Emma Douch	
~	~	Sara Fawcitt	
~	~	Jessica Hunt	
~	~	Claire Jarvis	
~	~	Marisa Lanzman	
~	~	Ruochen Li	
~	~	Su Fung Lo	
~	~	Kharishma Makani	
~	~	Angela McCadden	
~	~	Sabina Melander	
~	~	Aarti Nandani	
~	~	Lorna O'Shea	
~	~	Anna Osadcow	
~	~	Katherine Pigott	
~	~	Hannah Powell	
~	~	Kaliyanee Ramtohul	
~	~	Daniel Smith	
~	~	Tesha Suddason	
~	~	Elizabeth Woodford	
London, UK	Royal Marsden Hospital (London)	Vincent Khoo	PI
~	~	Ewan Chapman	Co-I
~	~	Laillah-Crystal Banda	
~	~	Trevor Bott	
~	~	Karen Brooks	
~	~	Karen Chan	
~	~	Rosalind Eeles	
~	~	Nicola Harman	

INVESTIGATORS AND COLLABORATORS: SITE STAFF
Staff on site delegation logs

City	Care_Site	Person_Name	Site_PI
~	~	Holly Hogan	
~	~	James Lowe	
~	~	Nicola Lucas	
~	~	Chloe McCormack	
~	~	Jennifer Morrison	
~	~	Vedang Murthy	
~	~	Annette Musallam	
~	~	Marisa Pinto Peixoto	
~	~	Suraya Quadir	
~	~	Alison Reid	
~	~	Debbie Rolfe	
~	~	Bernard Sill	
~	~	Bernard Siu	
~	~	Ruth Stafferton	
~	~	Helen Stidwell	
~	~	Sarah Storrs	
~	~	Debra Townsend-Thorn	
~	~	Nicholas Van As	
~	~	Vijitha Vijayakumar	
~	~	Li Wancheung	
London, UK	St Bartholomews Hospital (London)	Karen Tipples	PI
~	~	Paula Wells	Co-I
~	~	Marina Baccarini	
~	~	P Cathcart	
~	~	Samantha Chetiyawardana	
~	~	Fatjon Dekaj	
~	~	Shahanara Ferdous	
~	~	Stephanie Gibbs	
~	~	Denise Humfress	
~	~	Resmi Jayachandran	
~	~	Janet Kiff	
~	~	Cheryl Lawrence	
~	~	Wing-Kin Liu	

INVESTIGATORS AND COLLABORATORS: SITE STAFF
Staff on site delegation logs

City	Care_Site	Person_Name	Site_PI
~	~	Sebastien Martin	
~	~	Alastair Nicholson	
~	~	Jude Nixon	
~	~	Janet Oladimeji	
~	~	Hannah Payne	
~	~	Oscar Riches	
~	~	Jonathon Shamash	
~	~	Cavitha Vivekananthan	
London, UK	St Georges Hospital (London)	Mehran Afshar	PI
~	~	Laura Camburn	Co-I
~	~	Jason Chow	Co-I
~	~	Nia Alsamarrai	
~	~	Michael Brown	
~	~	Sue Cromarty	
~	~	Alice Dainty	
~	~	Deirdre Daly	
~	~	Serena Dover	
~	~	Gelareh Eslamian	
~	~	Claire Gilmartin	
~	~	Sophie Golden	
~	~	Jane Gregg	
~	~	Hakim Guessous	
~	~	Anne Haldeos	
~	~	Sam Hollingworth	
~	~	Geoffrey Howell	
~	~	Mohammed Mahgoub	
~	~	Roxane Mather	
~	~	Sophie McGrath	
~	~	Asha Mistry	
~	~	Uforma Ogrigri	
~	~	Chandni Patel	
~	~	Lisa Pickering	
~	~	Mark Quarrell	

INVESTIGATORS AND COLLABORATORS: SITE STAFF
Staff on site delegation logs

City	Care_Site	Person_Name	Site_PI
~	~	Debbie Rolfe	
~	~	Helen Tighe	
~	~	Juel Tuazon	
~	~	Robert Varro	
London, UK	St Marys Hospital (London)	Alison Falconer	PI
~	~	Melloney Allnutt	
~	~	Gareth Barker	
~	~	Angela Chamberlain	
~	~	Bindu Chikkamuniyappa	
~	~	Laura Custins	
~	~	Andrea Davis-Cook	
~	~	Steve Edwards	
~	~	Daisy Floyd	
~	~	Gillian Hornzee	
~	~	Joy Liao	
~	~	Joy Liau	
~	~	Zohanon Sabine Loko	
~	~	Stephen Mangar	
~	~	Akeema Paul	
~	~	Severine Rey	
~	~	Simon Stewart	
London, UK	University College Hospital	Ursula McGovern	PI
~	~	Richard Kaplan	Co-I
~	~	Mark Linch	Co-I
~	~	Heather Payne	Co-I
~	~	Adrienne Abioye	
~	~	Didem Agdiran	
~	~	Javeria Akhtar	
~	~	Hannah Ansell	
~	~	Uzma Asghar	
~	~	Natasha Aslam	
~	~	Aileen Austria	
~	~	Holly Baker (nee. Wing)	

INVESTIGATORS AND COLLABORATORS: SITE STAFF
Staff on site delegation logs

City	Care_Site	Person_Name	Site_PI
~	~	Ignacio Blanch	
~	~	Judith Cave	
~	~	Noan-Minh Chau	
~	~	Patricia Danaswamy	
~	~	Reena Davda	
~	~	Danny Garrett	
~	~	Annelies Gillesen	
~	~	Roshni Goel	
~	~	Stephen Harland	
~	~	Yemi Ilumoka	
~	~	Bihani Kularatne	
~	~	Jane Leach	
~	~	Suzy Lowi	
~	~	John Masters	
~	~	Anita Mitra	
~	~	Dieo Ottaviani	
~	~	Kristian Warnes	
~	~	Agnieska Zielonka	
~	~	Helene Zilkha	
London, UK	University College London	Holly Baker (nee. Wing)	
London, UK	University Hospital Lewisham	Aarti Shah	
London, UK	Whittington Hospital (London)	Simon Wan	
Maidstone, UK	Maidstone Hospital	Patryk Brulinski	PI
~	~	Delali Adjogatse	
~	~	Claire Baldry	
~	~	Sharon Beesley	
~	~	Jess Brady	
~	~	Vivienne Breen	
~	~	Jane Brown	
~	~	Su Burrage	
~	~	Clare Calvert	
~	~	Amanda Clarke	
~	~	Laura Clayton	

INVESTIGATORS AND COLLABORATORS: SITE STAFF
Staff on site delegation logs

City	Care_Site	Person_Name	Site_PI
~	~	Emma Craske	
~	~	Alison Davison	
~	~	Anna English	
~	~	Clary Evans	
~	~	Matthew Fittall	
~	~	Gavin Fossey	
~	~	Louise Hooper-Gilham	
~	~	Carmel Jope	
~	~	Emma Kipps	
~	~	Kathryn Lees	
~	~	Sarah Martins	
~	~	Romaana Mir	
~	~	Jane Murray	
~	~	Ian Pamphlett	
~	~	Joanne Patterson	
~	~	Ann Phillips	
~	~	Alison Richards	
~	~	Verity Roberts	
~	~	Alicia Synowiec	
~	~	Henry Taylor	
~	~	Katy Taylor	
~	~	Amie Thomas	
~	~	Lisa Tribe	
~	~	Joanne Williams	
~	~	Claudia Woodger	
Manchester, UK	Christie Hospital	Noel Clarke	PI
~	~	Ruth Conroy	Co-I
~	~	Christoph Oing	Co-I
~	~	Ali Al-Hashimi	
~	~	Susan Arrand	
~	~	Sreeja Aruketty	
~	~	Ian Bottomley	
~	~	Anna Bowron	

INVESTIGATORS AND COLLABORATORS: SITE STAFF
Staff on site delegation logs

City	Care_Site	Person_Name	Site_PI
~	~	Michael Braun	
~	~	Anna Bruzzan	
~	~	Megan Bunce	
~	~	Emma Burke	
~	~	Sharon Capper	
~	~	Clara Chan	
~	~	Stephen Chin	
~	~	Ananya Choudhury	
~	~	Richard Cowan	
~	~	Catherine Coyle	
~	~	Sue Davison	
~	~	Sarah-Ellen Ellen (née McCarthy)	
~	~	Tony Elliott	
~	~	Thiraviyam Elumalai	
~	~	Kim Fair	
~	~	Stefanie Fisder	
~	~	Laura Flanagan	
~	~	Silke Gillessen	
~	~	Lynne Gilmore	
~	~	Sarah Green	
~	~	Amber Hart	
~	~	Charlotte Heywood	
~	~	Andrew Hudson	
~	~	Cathryn James	
~	~	A Jegannathen	
~	~	Cathryn Jones	
~	~	Ather Kazmi	
~	~	Jacqueline Livsey	
~	~	John Logue	
~	~	Emma Lowther	
~	~	Jeanette Lyons	
~	~	Damian McCall	
~	~	Damian McCaul	

INVESTIGATORS AND COLLABORATORS: SITE STAFF
Staff on site delegation logs

City	Care_Site	Person_Name	Site_PI
~	~	Samah Mughal	
~	~	Roonak Nazari	
~	~	Kate O'Connor	
~	~	Jackie O'Dwyer	
~	~	Joanne Oliver	
~	~	Ekugbe Onogbe	
~	~	Ekugbe Onoge	
~	~	Alkesh Patel	
~	~	Kamlesh Patel	
~	~	Maria Petsa	
~	~	Catherine Pettersen	
~	~	Vijay Ramani	
~	~	Catherine Redshaw	
~	~	Vijay Sangar	
~	~	Sue Seifi	
~	~	Sarah-Ellen Smith	
~	~	Yee Pei Song	
~	~	Willemijn Spoor	
~	~	Martin Swinton	
~	~	Viv Thomas	
~	~	David Thompson	
~	~	Shaun Tolan	
~	~	Anna Tran	
~	~	Trishna Uttamlal	
~	~	Marie Woolley	
~	~	Lucy Worsley	
~	~	James Wylie	
~	~	You Yone	
~	~	salina tsui	
Manchester, UK	Withington Hospital	Vijay Sangar	PI
~	~	Vijay Ramani	Co-I
~	~	Humera Ahmed	
~	~	Linda Bailey	

INVESTIGATORS AND COLLABORATORS: SITE STAFF

Staff on site delegation logs

City	Care_Site	Person_Name	Site_PI
~	~	Vivienne Benson	
~	~	Julie Bramley	
~	~	Rebecca Corless	
~	~	Tania Cutts	
~	~	Annie Duffy	
~	~	Beatriz Duran Jimenez	
~	~	A. Emara	
~	~	Kathryn Fellows	
~	~	Anna Gipson	
~	~	Stephanie Hargreaves	
~	~	Helen Haydock	
~	~	Tarnya Hulme	
~	~	Damian McCall	
~	~	Thobekile Mthethwa	
~	~	Fiona Murtagh	
~	~	Lillian Partington	
~	~	Lindsay Piper	
~	~	Tracey Platt	
~	~	Catherine Redshaw	
~	~	Karen Robb	
~	~	Janet Smith	
~	~	Lorraine Turner	
~	~	James Wylie	
Manchester, UK	Wythenshawe Hospital	Vijay Sangar	PI
~	~	Linda Bailey	
~	~	Vivienne Benson	
~	~	Angela Chrisopoulou	
~	~	Annie Duffy	
~	~	Beatriz Duran Jimenez	
~	~	A. Emara	
~	~	Julie Fielding	
~	~	Angela Gowrie	
~	~	Wendy Guest	

INVESTIGATORS AND COLLABORATORS: SITE STAFF
Staff on site delegation logs

City	Care_Site	Person_Name	Site_PI
~	~	Sarah Liptrott	
~	~	Claire McGuire	
~	~	Kirsty Melia	
~	~	Thobekile Mthethwa	
~	~	Lindsay Piper	
~	~	Tracey Platt	
~	~	Kathryn Slevin	
Margate, UK	Queen Elizabeth The Queen Mother Hospital	Carys Thomas	PI
~	~	Albert Edwards	Co-I
~	~	Jessica Little	Co-I
~	~	Natasha Mithal	Co-I
~	~	Rakesh Raman	Co-I
~	~	Jennifer Turner	Co-I
~	~	Ifigenia Vasiliadou	Co-I
~	~	Louise Allen	
~	~	Bonny Appleby	
~	~	Sharon Beesley	
~	~	Hayley Blackgrove	
~	~	Tracy Boakes	
~	~	Patryk Brulinski	
~	~	Julie Buckley	
~	~	Miguel Capo-Mir	
~	~	Natalie Catt	
~	~	Mathilda Cominos	
~	~	Denise Crawford	
~	~	Nikki Crisp	
~	~	Steve Dann	
~	~	Julie-Ann Davies	
~	~	Susan Drakeley	
~	~	Clary Evans	
~	~	Sam Gibson	
~	~	Andrew Gillian	
~	~	Louise Gladwell	

INVESTIGATORS AND COLLABORATORS: SITE STAFF
Staff on site delegation logs

City	Care_Site	Person_Name	Site_PI
~	~	Coral Greenstreet	
~	~	Sandra Holness	
~	~	Laura Kehoe	
~	~	Sue Kelly	
~	~	Rachel Larkins	
~	~	Kathryn Lees	
~	~	Sarah Lightfoot	
~	~	Sarah Lines	
~	~	Margaret Lipsham	
~	~	Sydnie Loveland	
~	~	Rohit Malde	
~	~	Kim Mears	
~	~	Sharon Middleton	
~	~	Arafat Mirza	
~	~	Kannon Nathan	
~	~	Udaiveer Panwar	
~	~	Claire Pelham	
~	~	Karen Robinson	
~	~	Susan Rogers	
~	~	Lesley Rose	
~	~	Cindy Slater	
~	~	Mathini Sridharan	
~	~	Stephane Tankoua	
~	~	Katy Taylor	
~	~	Kim Travis	
~	~	Alba Tubau	
~	~	Kathleen (Kathy) Walsh	
~	~	Paula Whichelo	
~	~	Claire White	
~	~	Jo Williams	
~	~	Joanne Williams	
~	~	Elizabeth Williamson	
~	~	Victoria Williamson	

INVESTIGATORS AND COLLABORATORS: SITE STAFF
Staff on site delegation logs

City	Care_Site	Person_Name	Site_PI
~	~	Marian Wood	
~	~	Linda Wray	
~	~	Hilary Zurakovsky	
Middlesbrough, UK	James Cook University Hospital	Clive Peedell	PI
~	~	Alison Barnes	
~	~	Helen Carver	
~	~	David Chadwick	
~	~	Alison Chilvers	
~	~	Helen Dunn	
~	~	Claire Elliott	
~	~	Vicky Hanlon	
~	~	John Hardman	
~	~	Anne Hardwick	
~	~	Keith Harland	
~	~	Charlotte Jacobs(née Kitching)	
~	~	Paul Jones-King	
~	~	Mohammed Kagzi	
~	~	Sarah Kiddell	
~	~	Carol Long	
~	~	Emanuela Mahmoud	
~	~	Sarah McAuliffe	
~	~	Julia McBride	
~	~	Lynne Naylor	
~	~	Lisa Peacock (nee Wayman)	
~	~	Julie Potts	
~	~	Steven Pratt	
~	~	Fiona Rowling	
~	~	Luca Settimo	
~	~	Devadasan Shakespeare	
~	~	Agnieszka Skotnicka	
~	~	Emma Thompson	
~	~	Jane Thompson	
~	~	Katherine Tyler	

INVESTIGATORS AND COLLABORATORS: SITE STAFF
Staff on site delegation logs

City	Care_Site	Person_Name	Site_PI
~	~	Hans Van der Voet	
~	~	Andrea Watson	
~	~	David Wilson	
~	~	Jason Wong	
~	~	Maha Zarroug	
Newcastle upon Tyne, UK	Newcastle General Hospital	Judith Moore	
Newcastle-upon-Tyne, UK	Freeman Hospital	Ashraf Azzabi	PI
~	~	John Frew	Co-I
~	~	Shahid Iqbal	Co-I
~	~	Rhona McMenemin	Co-I
~	~	Ian Pedley	Co-I
~	~	Craig Alderson	
~	~	Katie Bain	
~	~	Lucy Blackwell	
~	~	Lauren Boal	
~	~	Penny Bradley	
~	~	Elle Cameron	
~	~	Ian Campbell	
~	~	Roger Carr	
~	~	Kay Carson	
~	~	Robert Chandler	
~	~	Caroline Dobeson	
~	~	Hannah Downs	
~	~	Sue Farrell	
~	~	Hazel Forsyth	
~	~	Elaine Greaves	
~	~	Noor Harris	
~	~	Amanda Henderson	
~	~	Andrew Herridge	
~	~	Ben Hood	
~	~	Ann Hudson	
~	~	Laura Jameson	
~	~	Thomas Jarvis	

INVESTIGATORS AND COLLABORATORS: SITE STAFF
Staff on site delegation logs

City	Care_Site	Person_Name	Site_PI
~	~	Xue Jiang	
~	~	Irene Jobson	
~	~	Mark Johnson	
~	~	Sunita Kholi	
~	~	Emma King	
~	~	Sunita Kollu	
~	~	Lavanya Mariappan	
~	~	Hazel Masson	
~	~	Peter Murphy	
~	~	Lesley Naik	
~	~	Gemma O'Neill	
~	~	Sarah Osborne	
~	~	Edgar Paez	
~	~	Elizabeth Reay	
~	~	Georgia Ross	
~	~	Sarah Rowling	
~	~	Jenny Smith	
~	~	Marianne Smith	
~	~	Naeem Soomro	
~	~	Carole Stobbart	
~	~	Julie Thohig	
~	~	Dianne Turner	
~	~	Dianne Wake	
~	~	Nichola Waugh	
Newport, UK	St Mary's Hospital (Newport)	Alison Brown	
~	~	Elizabeth Harrison	
~	~	Kudingila Madhava	
~	~	Tracey Tidbury	
~	~	Cindy Whitbread	
North Shields, UK	North Tyneside General Hospital	Mark Johnson	
Northampton, UK	Northampton General Hospital	Rachel Gabitass	
Northwood, UK	Mount Vernon Hospital	Peter Hoskin	PI
~	~	Viwod Mullassery	Ex-PI

INVESTIGATORS AND COLLABORATORS: SITE STAFF
Staff on site delegation logs

City	Care_Site	Person_Name	Site_PI
~	~	Hamoun Rozati	Co-I
~	~	Sara Abbassi	
~	~	Mohammed Abdul-Latif	
~	~	Farhan Ahmed	
~	~	Roberto Alonzi	
~	~	Nicola Anyamene	
~	~	Freya Ball	
~	~	Dolan Basak	
~	~	Rose Bell	
~	~	Neel Bhuva	
~	~	Sam Bosompem	
~	~	Jennifer Chard	
~	~	Lai Cheng Yew	
~	~	Helen Cladd	
~	~	Lucy Collins	
~	~	Janaka Cooray	
~	~	Nicola Cutmore	
~	~	Nazma Damani	
~	~	Paolo De Jesu	
~	~	Jeanette Dickson	
~	~	Kari Evans	
~	~	Jessica Finch	
~	~	Shiv Gayadeen	
~	~	Shaista Harpeer	
~	~	Olivia Hatcher	
~	~	Robert Hughes	
~	~	Rakhi Jain	
~	~	Suzanne Jenkins	
~	~	Bhanthi Kanagaratnam	
~	~	Sapna Kaur	
~	~	Rachael Khong	
~	~	Joanne Kosmin	
~	~	Paulina Kowalewska	

INVESTIGATORS AND COLLABORATORS: SITE STAFF
Staff on site delegation logs

City	Care_Site	Person_Name	Site_PI
~	~	Shakeda Lakha	
~	~	Sonia Li	
~	~	Elaine Lousley	
~	~	Henry Mandeville	
~	~	Jessica Milner	
~	~	Russell Moule	
~	~	Peter Ostler	
~	~	Kasia Owczarczyk	
~	~	Hannah Phillips	
~	~	Alice Ramsden	
~	~	Aamna Rashid	
~	~	Tahmina Shakil	
~	~	Mausam Singhera	
~	~	Linda Swaney	
~	~	David Tan	
~	~	Hannah Tharmalingam	
~	~	Harsha Vara	
~	~	Charlotte Westbury	
~	~	M Williams	
~	~	Katie Wood	
~	~	David Woolf	
~	~	Huiqi Yang	
~	~	Lai-Cheng Yew	
~	~	Kent Yip	
~	~	Claire Zane	
Nottingham, UK	Nottingham University Hospitals, City Campus	Santhanam Sundar	PI
~	~	Sadia Abdullah	Co-I
~	~	Eliot Chadwick	Co-I
~	~	Junhao Lim	Co-I
~	~	Rohan Tharaka	Co-I
~	~	Georgina Walker	Co-I
~	~	Leanne Alder	
~	~	Alex Blades	

INVESTIGATORS AND COLLABORATORS: SITE STAFF
Staff on site delegation logs

City	Care_Site	Person_Name	Site_PI
~	~	Matthew Brazkiewicz	
~	~	Louise Brookes	
~	~	Katie Carter	
~	~	Rena Chauhan	
~	~	Rachael Chivers	
~	~	Chin Chong	
~	~	Owen Cole	
~	~	Jade Eggleton	
~	~	Susan Elliott	
~	~	Charlotte Ellis	
~	~	Carol Gooch	
~	~	Stacey Green	
~	~	Lucy Howard	
~	~	Camille Hutchinson	
~	~	Daniel Kumar	
~	~	Adele Malson	
~	~	Jamie Mills	
~	~	Kayleigh Mills	
~	~	Kathryn Moore	
~	~	Asmaa Sa Omer	
~	~	Maeve Pomeroy	
~	~	Tin Sang-Tsang	
~	~	Daniel Saunders	
~	~	Ian Sayers	
~	~	Ewan Shawcroft	
~	~	Tania Slater	
~	~	Anita Stevenson	
~	~	Phillipa Sum	
~	~	Jacob Szolin-Jones	
~	~	Sarah Taylor	
~	~	Rohan Tharakan	
~	~	Hannah Thurlow	
~	~	Caitlin Todd	

INVESTIGATORS AND COLLABORATORS: SITE STAFF
Staff on site delegation logs

City	Care_Site	Person_Name	Site_PI
~	~	Sarah Widdowson	
Nuneaton, UK	George Eliot Hospital	Yakhub Khan	PI
~	~	Inderjit Atwal	
~	~	Jacob Bourne	
~	~	Andrew Chan	
~	~	Rachel Fergusson	
~	~	Sarah Fergusson	
~	~	Kerry Flahive	
~	~	Jessica Gunn	
~	~	Michaela Hill	
~	~	Pritpal Klear	
~	~	Jeanette Knapp	
~	~	Judith Lake	
~	~	Holly Lawrence	
~	~	Alison McCallum	
~	~	Andrea Mills	
~	~	Albert Mislang	
~	~	Sabiya Nasima	
~	~	Rachael Oates	
~	~	Winni Singh	
~	~	Melanie Taylor	
~	~	Andrew White	
~	~	Jenna Williams	
Oldham, UK	Royal Oldham Hospital	Ruth Conroy	PI
~	~	Ananya Choudhury	Co-I
~	~	Parth Desai	Co-I
~	~	Ehab Ibrahim	Co-I
~	~	Shaveta Mehta	Co-I
~	~	Anna Tran	Co-I
~	~	Mohammad Abutarb	
~	~	Joanne Allsop	
~	~	Hadia Ashraf	
~	~	Suzanne Bland	

INVESTIGATORS AND COLLABORATORS: SITE STAFF
Staff on site delegation logs

City	Care_Site	Person_Name	Site_PI
~	~	Wendy Cook	
~	~	Anthea Cree	
~	~	Kanal Gupta	
~	~	Ruth Halford	
~	~	Terence Hinton	
~	~	Shabaz Hussain	
~	~	Joanne Johnson	
~	~	Dawn Johnstone	
~	~	Richard Jones	
~	~	Helen Joyce	
~	~	Stephen Kennedy	
~	~	Victoria Lavin	
~	~	Mark Livingstone	
~	~	Jacqueline Livsey	
~	~	Peter Mbanu	
~	~	Jemma McLaughlin	
~	~	Leena Mistry	
~	~	Udeme Ohia	
~	~	Anna Pracz	
~	~	Kamala Ramatar	
~	~	Joanne Reed	
~	~	Agata Rembielak	
~	~	Dellesa Robinson	
~	~	Lyndsay Scarratt	
~	~	Shazril Imran Shaukat	
~	~	Amy Slack	
~	~	Kirstie Smith	
~	~	Hwoeifen Soohoo	
~	~	Richard Walshaw	
Oxford, UK	Churchill Hospital	Andrew Protheroe	PI
~	~	Daniel Ajzensztejn	Co-I
~	~	Gerard Andrade	Co-I
~	~	Philip Camilleri	Co-I

INVESTIGATORS AND COLLABORATORS: SITE STAFF
Staff on site delegation logs

City	Care_Site	Person_Name	Site_PI
~	~	Meenali Chitnis	Co-I
~	~	David J Cole	Co-I
~	~	Benjamin Fairfax	Co-I
~	~	Avinash Gupta	Co-I
~	~	Katherine Hyde	Co-I
~	~	Ami Sabharwal	Co-I
~	~	Robert Stuart	Co-I
~	~	Gemma Austin (nee Glover)	
~	~	Magdalena Benysek	
~	~	Lauren Booker	
~	~	Jane Boutflower	
~	~	Rosita Broderick	
~	~	Leigh Burns	
~	~	Anju Chalin	
~	~	Henry Chesson	
~	~	Richard Cousins	
~	~	Charlotte Davies	
~	~	Hugo De La Pena	
~	~	Ana De Veciana	
~	~	Jane Gibbard	
~	~	Will Goodman	
~	~	Trish Green	
~	~	Tessa Greenhalgh	
~	~	Elizabeth Hadley	
~	~	Silke Hahnewald	
~	~	Rachel Hart	
~	~	Katherine Jacob	
~	~	Patrycja Jastrzebska	
~	~	Evanthia Komninidou	
~	~	Sarah Lawrey	
~	~	Sarah Markus	
~	~	Kerrie Marston	
~	~	Paul Colin Miller	

INVESTIGATORS AND COLLABORATORS: SITE STAFF

Staff on site delegation logs

City	Care_Site	Person_Name	Site_PI
~	~	Matthew Mooney	
~	~	Sandra Mukkath	
~	~	Ann Murphy	
~	~	Julie Pinder	
~	~	Mark Prentice	
~	~	Thinn Pwint	
~	~	Laura Robledo	
~	~	Naveen Sankighatta	
~	~	Elaine Sugden	
~	~	Swapna Thummala	
~	~	Mark Tuthill	
~	~	Usharani Devi Wahengbam	
~	~	James Wakelin	
~	~	Robert Watson	
~	~	Sandie Wellman	
~	~	Kelly Wigglesworth	
~	~	Jo Wilson	
~	~	Martha Woodward	
~	~	Simon Wyatt	
~	~	Hazel Wynn	
Paisley, UK	Royal Alexandra Hospital	Tiago Rodrigues	
Poole, UK	Poole Hospital	Sue Brock	PI
~	~	Perric Crellin	Co-I
~	~	Joseph Davies	Co-I
~	~	Yogesh Nishchal	Co-I
~	~	Neal Beamish	
~	~	Hilary Blaney	
~	~	Deryck Burton	
~	~	Felicity Clapp	
~	~	Elizabeth Clarke	
~	~	Teresa Coffin	
~	~	Joe Davies	
~	~	Nichola Downs	

INVESTIGATORS AND COLLABORATORS: SITE STAFF
Staff on site delegation logs

City	Care_Site	Person_Name	Site_PI
~	~	Savina Elitova	
~	~	Maxine Flubacher	
~	~	Sally Gillespie	
~	~	Louise Heckford	
~	~	Amanda Iskender	
~	~	Lyn Jackson	
~	~	Stephanie Jones	
~	~	May Lwin	
~	~	Fiona Mellor	
~	~	Sally Munden	
~	~	Kate Mutendera	
~	~	Sara Orford	
~	~	Sarah Patch	
~	~	Sharon Power	
~	~	Sandy Pressdee	
~	~	Sophie Rix	
~	~	Susan Saxby	
~	~	Lee Tbaily	
~	~	Becky Troke	
~	~	Kate Urquhart	
~	~	Craig Vincent	
~	~	Emma Wesley	
~	~	Roger Wheelwright	
~	~	Delia Whiteman	
~	~	Emma Williams	
~	~	Elizabeth Woodward	
~	~	Seonaid Wright	
Portadown, UK	Craigavon Area Hospital	Judith Carser	PI
~	~	Fionnuala Houghton	Co-I
~	~	Leanne McCourt	
Portsmouth, UK	Queen Alexandra Hospital	Joanna Gale	PI
~	~	Shyamkia Acharige	Co-I
~	~	Oluwatobi Adeagbo	Co-I

INVESTIGATORS AND COLLABORATORS: SITE STAFF
Staff on site delegation logs

City	Care_Site	Person_Name	Site_PI
~	~	Giuseppe Banna	Co-I
~	~	Joanna Hack	Co-I
~	~	Harliana Mohd Yusof	Co-I
~	~	Syed Shah	Co-I
~	~	Jillian Andrews	
~	~	Kathy Blight	
~	~	Daniel Bloomfield	
~	~	Jack Broadfoot	
~	~	Tracy Callen	
~	~	Caroline Chau	
~	~	Jeng Heng Ching	
~	~	Heather Cuell	
~	~	Alisha Damani	
~	~	Charlotte Davies	
~	~	Tracey Dobson	
~	~	Sarah Ellis	
~	~	Wendy Golding	
~	~	Mya Gyi	
~	~	Jennifer Hale	
~	~	Dominic Hodgson	
~	~	Chloe Holden	
~	~	Joni Howells	
~	~	Eleanor Jones	
~	~	Robert Keating	
~	~	Kudingila Madhava	
~	~	Nataliya Martynyuk	
~	~	Lorna Meadows	
~	~	Badrriyya Mohamedali	
~	~	Yoodhvir Nagar	
~	~	Mark Noble	
~	~	Mila Roca	
~	~	Megan Rowley	
~	~	Wendy Stacey	

INVESTIGATORS AND COLLABORATORS: SITE STAFF

Staff on site delegation logs

City	Care_Site	Person_Name	Site_PI
~	~	Anna Stephenson	
~	~	Azarel Virgo	
~	~	Mary Wands	
~	~	Catrin Watkinson	
~	~	Alice White	
~	~	Robert Williams	
Preston, UK	Royal Preston Hospital	Alison Birtle	PI
~	~	Natalie Charnley	Co-I
~	~	Nicola Flaum	Co-I
~	~	Christina Hague	Co-I
~	~	Duleer Majeed	Co-I
~	~	Omi Parikh	Co-I
~	~	Sophie Raby	Co-I
~	~	Jose Rico	Co-I
~	~	Yee Pei Song	Co-I
~	~	Marcus Wise	Co-I
~	~	Amanda Alty	
~	~	Nafisa Arden	
~	~	Mandy Armstrong	
~	~	Andrea Ashton	
~	~	Katherine Ashton	
~	~	Hazel Aston	
~	~	David Barber	
~	~	Margaret Brunton	
~	~	Shelia Calvert	
~	~	Claire Corless	
~	~	Stephanie Cornthwaite	
~	~	William Croxford	
~	~	Sharon Curran	
~	~	Falalu Danwata	
~	~	Rose Ellard	
~	~	Davide Garau	
~	~	Cassandra Gleeson	

INVESTIGATORS AND COLLABORATORS: SITE STAFF
Staff on site delegation logs

City	Care_Site	Person_Name	Site_PI
~	~	Shahzad Gul	
~	~	Caroline Hatch	
~	~	Billy Hefferon	
~	~	Claire Hennigan	
~	~	Louise Hough	
~	~	Haiyan Huang	
~	~	Deepsi Khatiwada	
~	~	Patricia Knight	
~	~	Anna Macpherson	
~	~	Andrew Martyniak	
~	~	Dominic Mounsey	
~	~	Tanmay Mukhopadhyay	
~	~	Hemant Patel	
~	~	Hazel Preston	
~	~	Sarah Preston	
~	~	Christina Robinson	
~	~	Roy Shentall	
~	~	Norma Sidek	
~	~	Win Soe	
~	~	Martin Swinton	
~	~	Catherine Thompson	
~	~	Nina Vekaria	
~	~	Catherine Walmsley	
~	~	Rebecca Wilby (nee Hall)	
~	~	Deborah Williamson	
Reading, UK	Royal Berkshire Hospital	Paul Rogers	PI
~	~	Osamah Al-Asadi	Co-I
~	~	Rowena Cazalet	Co-I
~	~	Rebecca Johnson	Co-I
~	~	Ali Abbas	
~	~	Abdolnasser Aminiraouf	
~	~	Jane Atkinson	
~	~	Gabrielle Ball	

INVESTIGATORS AND COLLABORATORS: SITE STAFF
Staff on site delegation logs

City	Care_Site	Person_Name	Site_PI
~	~	Gagan Bhatnagar	
~	~	Richard B Brown	
~	~	Debbie Cartwright	
~	~	James Church	
~	~	Claire Connolly	
~	~	Kristy Coomber	
~	~	Nicola Dallas	
~	~	Catherine Deytrikh-Smith	
~	~	Juliette Dye	
~	~	Shawn Ellis	
~	~	Fiona Everson	
~	~	Suzanne Foxwell	
~	~	Maxine Gauntlett	
~	~	Anna Gillham	
~	~	Sanita Gurm	
~	~	Royda Hadi	
~	~	Silke Hahnewald	
~	~	Jo Hand	
~	~	Elizabeth Haydon	
~	~	Kirsty Horwood	
~	~	Allison Hunt	
~	~	Sian James	
~	~	Phillipa Johnstone	
~	~	Robert Jones	
~	~	Thomas Kindley	
~	~	Wioletta Kowalczyk-Williams	
~	~	Christina Lewis	
~	~	Geraldine Mason	
~	~	Sean O'Cathail	
~	~	Helen O'Donnell	
~	~	Omotola Ogunnigbo	
~	~	Tolu Okeke	
~	~	Pooja Pabari	

INVESTIGATORS AND COLLABORATORS: SITE STAFF
Staff on site delegation logs

City	Care_Site	Person_Name	Site_PI
~	~	Stephen Parr	
~	~	Kate Preston	
~	~	Helen Purdon	
~	~	Norma Shields	
~	~	Georges Sinclair	
~	~	Emma Vowell	
~	~	Phillip Webb	
~	~	Simon Wyatt	
~	~	Andreia da Cruz	
Redditch, UK	Alexandra Hospital	Lisa Capaldi	PI
~	~	Mujtaba Syed-Khaja	Co-I
~	~	Maggie Brown	
~	~	Stephanie Cook	
~	~	Jonathan Davies	
~	~	Joanna Hamilton	
~	~	Alison Harrison	
~	~	Hayley Hodson	
~	~	Jeanette Knapp	
~	~	Bartlomeij Kurec	
~	~	Asha Sivapalasuntharam	
~	~	Helen Tranter	
~	~	Jennifer Young	
Redhill, UK	East Surrey Hospital	Eva Letalova	
Romford, UK	Oldchurch Hospital	Neil Fisher	
Romford, UK	Queen's Hospital (Romford)	Kathryn Tarver	PI
~	~	Stephanie Gibbs	Ex-PI
~	~	Amani Chowdhury	
~	~	Dalisay Domingo	
~	~	Parveen Dugh	
~	~	Revanth Jannapureddy	
~	~	Mohammed Rashid Khan	
~	~	Helen Mackenzie	
~	~	Tina Mills-Baldock	

INVESTIGATORS AND COLLABORATORS: SITE STAFF

Staff on site delegation logs

City	Care_Site	Person_Name	Site_PI
~	~	Simerjyot Mudhar	
~	~	Samuel Mugari	
~	~	Neale O'Brien	
~	~	Ana-Marie Pena-Remorin	
~	~	Yousaf Razzak	
~	~	Jonathon Shamash	
~	~	Ramachandran Subramaniam	
Runcorn, UK	Halton Hospital	Ian Allen	
~	~	Duncan Knowles	
~	~	Carrie Lowthian	
~	~	Rebecca Madew (nee Tinker)	
~	~	Nemonie Marriott	
~	~	Andrea Young	
Salford, UK	Salford Royal Hospital	Noel Clarke	PI
~	~	Tony Elliott	Co-I
~	~	Euan Green	Co-I
~	~	Maurice Lau	Co-I
~	~	Anna Tran	Co-I
~	~	Rachael Allen	
~	~	Angela Ashton	
~	~	Chris Betts	
~	~	Nicholas Boxall	
~	~	Richard Cowan	
~	~	Soney Dharmaprasad	
~	~	Claire Dickson	
~	~	Claire Duncan (nee Keatley)	
~	~	Christine Farnworth	
~	~	Helen Farrell	
~	~	Kathryn Fry	
~	~	Siny George	
~	~	Kay Goulden	
~	~	Samia Hanif	
~	~	Ashley Harris	

INVESTIGATORS AND COLLABORATORS: SITE STAFF
Staff on site delegation logs

City	Care_Site	Person_Name	Site_PI
~	~	Leah Harter	
~	~	Joanne Henry	
~	~	Jason Howard	
~	~	Jean Jellicoe	
~	~	Richard Jones	
~	~	Elina Jose	
~	~	Claire Keatley	
~	~	Sarah Kirk	
~	~	Kieran O'Flynn	
~	~	Anne-Marie Peers	
~	~	Danielle Platt	
~	~	Catherine Redshaw	
~	~	David Shackley	
~	~	Mark Stapleton	
~	~	Melanie Taylor	
~	~	Vicky Thomas	
~	~	Cellins Vinod	
~	~	Oliver Wadsworth	
~	~	Jill Youd	
Scarborough, UK	Scarborough General Hospital	Mohammad Muneeb Khan	PI
~	~	Mohan Hingorani	Ex-PI
~	~	Simon Hawkyard	Co-I
~	~	Khaliq Rehman	Co-I
~	~	Alison Ames	
~	~	Donna Anderson	
~	~	Lisa Armitage	
~	~	Fizzah Asif	
~	~	Laura Barman	
~	~	Chloe Box	
~	~	Kevin Brame	
~	~	Pippa Carlton-Rylance	
~	~	Courtney Cole	
~	~	Poppy Cottrell-Howe	

INVESTIGATORS AND COLLABORATORS: SITE STAFF
Staff on site delegation logs

City	Care_Site	Person_Name	Site_PI
~	~	Cheryl Donne	
~	~	Nabil El-Mahdawi	
~	~	Arran Fletcher	
~	~	Joanne Fletcher	
~	~	Vic Gacek	
~	~	Tracey Hawkes	
~	~	Sacha Honour	
~	~	Diana Ionita	
~	~	Adnan Kabir	
~	~	Sarah Kent	
~	~	Richard Khafagy	
~	~	Janine Mallinson	
~	~	Russell Morgan	
~	~	Tania Neale	
~	~	Polly Needs	
~	~	Anne Nunn	
~	~	Carol Popplestone	
~	~	Ian Renwick	
~	~	Andrew Robertson	
~	~	Alicia Rodgers	
~	~	Abigail Rowbotham	
~	~	Jacqui Smith	
~	~	Rachel Spooner	
~	~	Amie Stewart	
~	~	Jane Taylor	
~	~	Alison Turnbull	
~	~	Paul Wood	
Sheffield, UK	Weston Park Hospital	Carmel Pezaro	PI
~	~	Omar Din	Co-I
~	~	Shabbir Rawther	Co-I
~	~	Virgil Sivoglo	Co-I
~	~	Jess Aldred	
~	~	Cyper Allan	

INVESTIGATORS AND COLLABORATORS: SITE STAFF

Staff on site delegation logs

City	Care_Site	Person_Name	Site_PI
~	~	Mymoona Alzouebi	
~	~	Ryan Asher	
~	~	Lynne Ashmore	
~	~	Lucy Birch	
~	~	Joanne Bird	
~	~	Susan Bishop	
~	~	Katie Bowen	
~	~	Janet Brown	
~	~	Richard Brown	
~	~	Sarah Brown	
~	~	Roger Burkinshaw	
~	~	Chloe Clegg	
~	~	Gemma Dale	
~	~	Tathagata Das	
~	~	Julia Disney	
~	~	Linda Evans	
~	~	Catherine Ferguson	
~	~	Leigh Fiorentino	
~	~	Alexandra Firth	
~	~	Steffy George	
~	~	Kate Gibbins	
~	~	Elizabeth Hodgkinson	
~	~	Mark Holliday	
~	~	Marion Hutchinson	
~	~	Peter Kirkbride	
~	~	James Lester	
~	~	Rebecca Lomax-Allen	
~	~	Eileen Marsh	
~	~	John Martindale	
~	~	Jessica Medcalf	
~	~	Louise Murray	
~	~	Prashanth Sanganalmath	
~	~	Ruta Segamogaite	

INVESTIGATORS AND COLLABORATORS: SITE STAFF
Staff on site delegation logs

City	Care_Site	Person_Name	Site_PI
~	~	Roseleen Sheehan	
~	~	Janine Smedley (nee McCabe)	
~	~	Lucy Smith	
~	~	Anne Smythe	
~	~	Catherine Spalton	
~	~	Rachel Toes	
~	~	Lucy Walkington	
~	~	Katherine Williams	
~	~	Kim Wood	
Shrewsbury, UK	Royal Shrewsbury Hospital	Narayanan Srihari	PI
~	~	Ravi Prashant	Co-I
~	~	Riquella Abbott	
~	~	Huzeifa Abdel	
~	~	Marion Adams	
~	~	Beshar Allos	
~	~	Shazad Aslam	
~	~	Mandy Bates	
~	~	Erica Beaumont	
~	~	Mandy Beekes	
~	~	James Best	
~	~	Rajanee Bhana	
~	~	Lisa Capaldi	
~	~	Danielle Childs	
~	~	Lisa Evans	
~	~	Gill Ferguson	
~	~	Huzeifa Gadir	
~	~	Qamar Ghafoor	
~	~	Nicola Henderson	
~	~	Hayley Hughes	
~	~	Nicola Jones	
~	~	Sanal Jose	
~	~	Siobhan Kilbane	
~	~	Verity King	

INVESTIGATORS AND COLLABORATORS: SITE STAFF
Staff on site delegation logs

City	Care_Site	Person_Name	Site_PI
~	~	Sunita Kurian-Downer	
~	~	Jenny Lakin	
~	~	Anna Law	
~	~	Gemma Lee	
~	~	Michael Leigh	
~	~	Rachel McGregor	
~	~	Elena Michael	
~	~	Helen Moore	
~	~	Emma Neeves	
~	~	Karen Nicholas	
~	~	Catherine Orrell	
~	~	Lucy Pennant	
~	~	Craig Pickering	
~	~	Suzanne Pope	
~	~	Sally Potts	
~	~	Renee Poulsom	
~	~	Aitzaz Qaisar	
~	~	Catherine Santiago	
~	~	Gemma Searle	
~	~	Jenny Simm	
~	~	Harpreet Singh	
~	~	Sandra Smith	
~	~	Andy Taylor	
~	~	Alison Tilley	
~	~	Mathai Varghese	
~	~	Natasha Wallbank	
~	~	Emma Weaver	
~	~	Rebecca Wilcox	
~	~	Sundus Yahya	
~	~	Angela Yeomans	
~	~	Abel Zachariah	
South Shields, UK	South Tyneside District Hospital	Ashraf Azzabi	PI
~	~	Amy Burns	

INVESTIGATORS AND COLLABORATORS: SITE STAFF
Staff on site delegation logs

City	Care_Site	Person_Name	Site_PI
~	~	Maxine Goldsbrough	
~	~	Sally Hall	
~	~	Judith Moore	
~	~	Sue Morrison	
~	~	Ruth Tindle	
Southampton, UK	Southampton General Hospital	Simon Crabb	PI
~	~	Emma Brown	Co-I
~	~	Tessa Greenhalgh	Co-I
~	~	Chloe Holden	Co-I
~	~	Harish Reddy	Co-I
~	~	Caroline Andrews	
~	~	Liane Armstrong	
~	~	Holly Burton	
~	~	Nikki Carney	
~	~	Chris Coyle	
~	~	Kirsty Cumming	
~	~	Lucy Elswood	
~	~	Archana Gadve	
~	~	Julie Gwilt	
~	~	Annelise Haskell	
~	~	Catherine Heath	
~	~	Julie Kennedy	
~	~	Donna Kimber	
~	~	Yanli Li	
~	~	Maureen McAuley	
~	~	Victoria McFarlane	
~	~	Graham Mead	
~	~	Carolyn Mitchell	
~	~	Fabiola Morales-Azofra	
~	~	Susan Morton	
~	~	Carina Mundy	
~	~	Oyeleye Oyebola	
~	~	Nikki Prewitt	

INVESTIGATORS AND COLLABORATORS: SITE STAFF
Staff on site delegation logs

City	Care_Site	Person_Name	Site_PI
~	~	Leanne Reader	
~	~	Rebecca Rice	
~	~	Adele Ruiz	
~	~	Lorraine Street	
~	~	Sau-Mon Tsang	
~	~	Shauna Wakefield	
~	~	Matthew Wheater	
~	~	Aneta Zahorska	
Southport, UK	Southport and Formby District General Hospital	Manal Alameddine	PI
~	~	Neeraj Bhalla	Ex-PI
~	~	Dawn Barker	
~	~	Margaret Brunton	
~	~	Lisa Dobson (nee Child)	
~	~	Chinnamani Eswar	
~	~	Ken Gardner	
~	~	Julie Griffiths	
~	~	Laurie Lomax	
~	~	Marie McBride	
~	~	Teresa Monahan	
~	~	Heidi Moran	
~	~	Anna Morris	
~	~	Sandra Robinson	
~	~	Linda Schinkel	
~	~	Angela Scullion	
~	~	Asha Sivapalasuntharam	
~	~	Ann Wearing	
St Leonards-on-Sea, UK	Conquest Hospital	Caroline Manetta	PI
~	~	Atikah Ayaz	
~	~	Theresa Baumber	
~	~	Sharon Beesley	
~	~	Sarah Draper	
~	~	Steve Garnett	
~	~	Duncan Gilbert	

INVESTIGATORS AND COLLABORATORS: SITE STAFF
Staff on site delegation logs

City	Care_Site	Person_Name	Site_PI
~	~	Sarah Goodwin	
~	~	Joanna Howard	
~	~	Kay Jones-Skipper	
~	~	Kathryn Lees	
~	~	Lauren McCrisken	
~	~	Fiona McKinna	
~	~	Roger Plail	
~	~	Gail Pottinger	
~	~	Aspasia Soultati	
~	~	Jo-Anne Taylor	
~	~	Mark Whitfield	
Stevenage, UK	Lister Hospital	Robert Hughes	PI
~	~	Stephen Almond	
~	~	Anna Anosova	
~	~	Alkhaldi Ashraf	
~	~	Mawuelikem Assoku	
~	~	Corinne Bradshaw	
~	~	Clare Collins	
~	~	Sura Dabbagh	
~	~	Martin Ebon	
~	~	Jemma Gilmore	
~	~	Sunita Gohil	
~	~	Vicky Hills	
~	~	Rachel Low	
~	~	Leena Mukherjee	
~	~	Sayyida Nembhard	
~	~	Nikhil Oommen	
~	~	Katie Poole	
~	~	Natalie Rahim	
~	~	Anita Rana	
~	~	Roisin Schimmel	
~	~	Jonathan Towler	
~	~	Alice Valle	

INVESTIGATORS AND COLLABORATORS: SITE STAFF
Staff on site delegation logs

City	Care_Site	Person_Name	Site_PI
~	~	David Ward	
~	~	Steven Watkins	
~	~	Elen Witness	
~	~	David Woolf	
Stockport, UK	Stepping Hill Hospital	John Logue	PI
~	~	Adebanji Adeyoju	
~	~	Wasim Akhtar	
~	~	Carmel Anandadas	
~	~	Eleanor Anscombe	
~	~	Miriam Avery	
~	~	Paul Berry	
~	~	Aelens Brauckman	
~	~	Stephen Bromage	
~	~	Richard Brough	
~	~	Louise Brown	
~	~	Stephen CW Brown	
~	~	Jean Cheetham	
~	~	Pat Clitheroe	
~	~	Tracie Cocks	
~	~	Gerald Collins	
~	~	Sarah Connolly nee McKenna	
~	~	Sam Corcoran	
~	~	Catherine Coyle	
~	~	Catherine Fox	
~	~	Christina Gilmour	
~	~	Emma Goodwin	
~	~	Susan Graham	
~	~	Umi Hatimy	
~	~	Helen Haydock	
~	~	Nicola Hermitage	
~	~	Emma Hewitt	
~	~	Sheila Hodgkinson	
~	~	Susan Hopkins	

INVESTIGATORS AND COLLABORATORS: SITE STAFF
Staff on site delegation logs

City	Care_Site	Person_Name	Site_PI
~	~	Apurna Jegannathen	
~	~	Zoe Jordan	
~	~	Anna Kellingray	
~	~	Alissa Kent	
~	~	John Kilmartin	
~	~	Magda Kujawa	
~	~	Abigail Mackley	
~	~	Patrick O'Reilly	
~	~	Oluwademilade Odewumi	
~	~	Lucy Orrell	
~	~	Abigail Pemberton	
~	~	Benjamin Ralphs	
~	~	Mkyla Reilly	
~	~	David Ross	
~	~	Andrew Sinclair	
~	~	Emma Taylor	
~	~	Jill Taylor	
~	~	Satish Venkateshan	
~	~	Katrina Wade	
~	~	Jonathan Wong	
~	~	Donald van Welsenes	
Stockton-on-Tees, UK	North Tees General Hospital	Devadasan Shakespeare	
Stockton-on-Tees, UK	University Hospital of North Tees	Darren Leaning	PI
~	~	Alison Chilvers	
~	~	Helen Dunn (nee Carey)	
~	~	Emma Jameson	
~	~	Hyder Latif	
~	~	Abdul Mian	
~	~	Victor Palit	
~	~	Moira Percival	
~	~	Sarah Pitcairn	
~	~	Leigh Pollard	
~	~	Lynda Poole	

INVESTIGATORS AND COLLABORATORS: SITE STAFF

Staff on site delegation logs

City	Care_Site	Person_Name	Site_PI
~	~	Pam Race	
~	~	Devadasan Shakespeare	
~	~	Andrew Sigsworth	
~	~	Helen Wardle (nee Wilson)	
~	~	Bill Wetherill	
Stoke-on-Trent, UK	Royal Stoke University Hospital	Salil Vengalil	PI
~	~	Fawzi Adab	
~	~	Eden Ball	
~	~	Rajanee Bhana	
~	~	Isabel Breeze	
~	~	Marion Evans	
~	~	Grace Gough	
~	~	Robert Green	
~	~	Emma Jackson	
~	~	Christopher Luscombe	
~	~	Alison Myatt	
~	~	Katrina Parkinson	
~	~	Angela Peake	
~	~	Sharon Rollison	
~	~	Elizabeth Sellars	
~	~	Rowena Smith	
~	~	Julie Storer	
~	~	Alison Tute	
~	~	Liberty Verueco	
~	~	Angela Ward	
~	~	Elizabeth Williamson	
Sunderland, UK	Sunderland Royal Hospital	Ashraf Azzabi	PI
~	~	Rachel Pearson	Co-I
~	~	Ian Pedley	Co-I
~	~	Kathryn Wright	Co-I
~	~	Rod Beard	
~	~	Stephen Butler	
~	~	Jane Cole	

INVESTIGATORS AND COLLABORATORS: SITE STAFF
Staff on site delegation logs

City	Care_Site	Person_Name	Site_PI
~	~	Michelle Edwards	
~	~	Terri Haldane	
~	~	Christine Harle	
~	~	Amanda Howey	
~	~	Vivienne Hullock	
~	~	Shahid Iqbal	
~	~	Stephen Laybourne	
~	~	Paula Newton	
~	~	Julia Scott	
~	~	Karen Shield	
~	~	Fiona Wakinshaw	
Sutton Coldfield, UK	Good Hope Hospital	Daniel Ford	PI
~	~	Mark O'Beirn	Co-I
~	~	Kamaldeep Ajimal	
~	~	Shobit Baijal	
~	~	Chen Bartlett	
~	~	Ellen Drew	
~	~	Steve Hay	
~	~	Lubna Khan	
~	~	Alison Maidment	
~	~	Beena Mistry	
~	~	Katy Moore	
~	~	Rachael O'Beney	
~	~	Janet Prentice	
~	~	Sarah Rogers	
~	~	Sundip Sohanpal	
~	~	Lorna Swaddle	
~	~	Helen Taylor	
~	~	Helen Thomas	
~	~	James Whitehouse	
Sutton, UK	Royal Marsden Hospital (Sutton)	Chris Parker	PI
~	~	Douglas Brand	Co-I
~	~	Angela Pathmanathan	Co-I

INVESTIGATORS AND COLLABORATORS: SITE STAFF
Staff on site delegation logs

City	Care_Site	Person_Name	Site_PI
~	~	Nora Sundahl	Co-I
~	~	Fatima Ahmed	
~	~	Rookmeen Alighan	
~	~	Eva Batovska	
~	~	Martha Bullimore	
~	~	Sue Cromarty	
~	~	Claire Crowley	
~	~	Kirsty Cuthbertson	
~	~	David Dearnaley	
~	~	Rosalind Eeles	
~	~	Lucy Featherstone	
~	~	Janine Flohr	
~	~	Amir El Ghazal	
~	~	Zaynah Gurreebun	
~	~	Laura Hennelly	
~	~	Adham Hijab	
~	~	Alan Horwich	
~	~	Robert Huddart	
~	~	Nick Hunnings	
~	~	Tiaan Jacobs	
~	~	Bernadette Johnson	
~	~	Kelly Jones	
~	~	Vincent Khoo	
~	~	Susan Lalondrelle	
~	~	Alexander Macnab	
~	~	Chloe McCormack	
~	~	Gerard McVey	
~	~	Sally Moore	
~	~	Annette Musallam	
~	~	Jenni Parmar	
~	~	Ray Shepherd	
~	~	Victoria Sjolin	
~	~	Helen Stidwell	

INVESTIGATORS AND COLLABORATORS: SITE STAFF
Staff on site delegation logs

City	Care_Site	Person_Name	Site_PI
~	~	Alex Tan	
~	~	Alison Tree	
~	~	Ruth Woode-Amissah	
Sutton-in-Ashfield, UK	King's Mill Hospital	Georgina Walker	PI
~	~	Daniel Saunders	Ex-PI
~	~	Louise Brookes	Co-I
~	~	Benjamin Masters	Co-I
~	~	Sadia Abdullah	
~	~	Samantha Boam	
~	~	Andrew Brocklehurst	
~	~	Jamie-Rae Burgoyne	
~	~	Eliot Chadwick	
~	~	Muhammad Gill	
~	~	Robert Goldspring	
~	~	Steve Haigh	
~	~	Shila Hamzepur	
~	~	Rebecca Holmes	
~	~	Lauren Jones	
~	~	Jun Lim	
~	~	Wayne Lovegrove	
~	~	Samantha March	
~	~	Victoria Moore	
~	~	Dominic Nash	
~	~	Michael Ocathail	
~	~	Linda Otter	
~	~	Andrea Palfreman	
~	~	James Price	
~	~	Lisa Rahn	
~	~	Wai Hou Sam	
~	~	Terri-Ann Sewell	
~	~	Sarah Shelton	
~	~	Katie Slack	
~	~	Fiona Smith	

INVESTIGATORS AND COLLABORATORS: SITE STAFF

Staff on site delegation logs

City	Care_Site	Person_Name	Site_PI
~	~	Susan Smith	
~	~	Sarah Taylor	
~	~	Elena Umbrarescu	
~	~	Lynne Wade	
~	~	Margaret Wheatley	
~	~	Inez Wynter	
Swansea, UK	Singleton Hospital	Ahmed Shaheen	PI
~	~	Rhian Davies	Co-I
~	~	Helen Fitzgerald	Co-I
~	~	Nia Jackson	Co-I
~	~	Sheena Lam	Co-I
~	~	Aijaz Lone	Co-I
~	~	Wael Mohamed	Co-I
~	~	Mau-Don Phan	Co-I
~	~	Fiona Williams	Co-I
~	~	Carl Ackland	
~	~	Russell Banner	
~	~	Gianfilippo Bertelli	
~	~	Lynne Breeze-Jones	
~	~	David Brown	
~	~	Jayne Caparros	
~	~	Helen Cheley	
~	~	Karen Chesters	
~	~	Amanda Cook	
~	~	Emma Dangerfield	
~	~	Nicola Davies	
~	~	Lisa Ellis	
~	~	Elizabeth Evans	
~	~	Stuart Evans	
~	~	Tracey Ford	
~	~	Alex Franklin	
~	~	Ricky Fraser	
~	~	Lorraine Gammon	

INVESTIGATORS AND COLLABORATORS: SITE STAFF
Staff on site delegation logs

City	Care_Site	Person_Name	Site_PI
~	~	Sharath Gangadhara	
~	~	Judith Gooding	
~	~	Sarah Gwynne	
~	~	Emily Harris (n. Marchant)	
~	~	Amanda Jackson	
~	~	Chelsea Jenkins	
~	~	Maria Johnstone	
~	~	Gillian Jones	
~	~	Lewis Jones	
~	~	Ashok Kumar	
~	~	Satish Kumar	
~	~	Donna Lear	
~	~	Nicola Lemon	
~	~	Jason Lester	
~	~	James Morgan	
~	~	Gillian Palmer	
~	~	Angharad Phillips	
~	~	Brian Phillips	
~	~	Karen Phillips	
~	~	Susie Pitcher	
~	~	Gail Povey	
~	~	Euan Pratt	
~	~	Delia Pudney	
~	~	Leanne Quinn	
~	~	Amy Quinton	
~	~	Alex Richards	
~	~	Mair Roberts	
~	~	Mark Rogers	
~	~	Michelle Romano	
~	~	N Sindgi	
~	~	Alison Stretch	
~	~	Ellen Tait	
~	~	Katie Tanner	

INVESTIGATORS AND COLLABORATORS: SITE STAFF
Staff on site delegation logs

City	Care_Site	Person_Name	Site_PI
~	~	Anne Thomas	
~	~	Nia Viney	
~	~	John Wagstaff	
~	~	Gillian Willetts	
~	~	Dawn Withers	
~	~	Naomi Woods	
~	~	Charlotte Young	
Swindon, UK	Great Western Hospital	Omar Khan	PI
~	~	Gerard Andrade	
~	~	Aiste Baltramaityte	
~	~	Rebecca Belcher	
~	~	Graham Brown	
~	~	Christopher Clarke	
~	~	David J Cole	
~	~	Amanda Colston	
~	~	Sarah Cotton	
~	~	Nicola Cowling	
~	~	Shiroma De Silva-Minor	
~	~	Jan Dodge	
~	~	Fahad Fazal	
~	~	Victoria Gibson	
~	~	Sarah Grayland	
~	~	Lesley Haxton	
~	~	Ellie Hewitt	
~	~	Esme Hill	
~	~	Raj Jampana	
~	~	Ania Jones	
~	~	Jean Kordula	
~	~	Lynsey Kyeremeh	
~	~	Donna Lake	
~	~	Jonathan Lewis	
~	~	Mike Lewis	
~	~	Catherine Lewis Clarke	

INVESTIGATORS AND COLLABORATORS: SITE STAFF
Staff on site delegation logs

City	Care_Site	Person_Name	Site_PI
~	~	Sarah Long	
~	~	Dorothe Maramak	
~	~	Dorota Marciniak	
~	~	Laura McCafferty	
~	~	Sue Meakin	
~	~	Aruna Medisetti	
~	~	Rachel Messenger	
~	~	Chanelle Meyer	
~	~	David Newell	
~	~	Tim Owen	
~	~	Debbie Palmer	
~	~	Cerila Parajes	
~	~	Sally-Ann Parkin (nee) Lee	
~	~	Ronak Patel	
~	~	Suzannah Pegler	
~	~	Caroline Pensotti	
~	~	Tracey Sargent	
~	~	Deborah Scott	
~	~	Karen Smith	
~	~	Ellen Starling	
~	~	Joseph Stevens	
~	~	Emma Wakefield	
~	~	Helen Winter	
~	~	Vivian Zinyemba	
Taunton, UK	Musgrove Park Hospital	Emma Gray	PI
~	~	John Graham	Ex-PI
~	~	Nicola Cox	Co-I
~	~	Mohini Varughese	Co-I
~	~	John Allinson-Smith	
~	~	Jan Ashcroft	
~	~	Nita Beacham	
~	~	Hannah Berry	
~	~	Ian Bodger	

INVESTIGATORS AND COLLABORATORS: SITE STAFF

Staff on site delegation logs

City	Care_Site	Person_Name	Site_PI
~	~	Joanne Botten	
~	~	Lisa Bowern	
~	~	Darren Brady	
~	~	Christina Branfield	
~	~	Rebecca Brown	
~	~	Clair Brunner	
~	~	Richard Burgess	
~	~	Alison Chedham	
~	~	Rachel Coe	
~	~	Hayley Cornall	
~	~	Susan Crouch	
~	~	Nicola Cutmore	
~	~	Rebecca Denslow	
~	~	Jarrod Dunn	
~	~	Michelle Farrar	
~	~	Abby Farzaneh	
~	~	Simon Goldsworthy	
~	~	Fiona Goodchild	
~	~	Amanda Groves	
~	~	Clair Hinton	
~	~	Lucy Howell-Drewett	
~	~	Joseph Jelski	
~	~	Odunayo Kalejaiye	
~	~	Joan Kemp	
~	~	Manjusha Keni	
~	~	Catherine Lane	
~	~	Lynn Leat	
~	~	Fen Lewen	
~	~	Angela Locke	
~	~	Ruaraidh MacDonagh	
~	~	Sue Mahoney	
~	~	Anna Masamba	
~	~	Judith Mathie	

INVESTIGATORS AND COLLABORATORS: SITE STAFF

Staff on site delegation logs

City	Care_Site	Person_Name	Site_PI
~	~	Sara Myers	
~	~	Sayyida Nembhard	
~	~	Samantha Northover	
~	~	Corinne Pawley	
~	~	George Plataniotis	
~	~	Ceri Poyntz-wright	
~	~	Rebecca Purnell	
~	~	Gihan Ratnayake	
~	~	Guillermo Reina-Ruiz	
~	~	Joanne Rogers	
~	~	Joy Rowe	
~	~	Tamlyn Russell	
~	~	Amy Sawyer	
~	~	Alison Snell	
~	~	Claire Sowerby	
~	~	Luke Stephens	
~	~	Moira Tait	
~	~	Karen Tanner	
~	~	Joanne Taylor	
~	~	Mary Tighe	
~	~	Rebecca Tucker	
~	~	Rebecca Twemlow	
~	~	Elena Umbrarescu	
~	~	Rebecca Wallbutton	
~	~	Joshua Woollven	
~	~	Jasmine Youens	
~	~	Robert Zorica	
Taunton, UK	Taunton and Somerset Hospital	Jan Ashcroft	
~	~	Jarrod Dunn	
~	~	Ruaraidh MacDonagh	
~	~	Judith Mathie	
~	~	Rebecca Tucker	
Torquay, UK	Torbay District General Hospital	Anna Lydon	PI

INVESTIGATORS AND COLLABORATORS: SITE STAFF
Staff on site delegation logs

City	Care_Site	Person_Name	Site_PI
~	~	Fiona Roberts	Co-I
~	~	Michele Allison	
~	~	Kenneth Almedilla	
~	~	Emmie Arbury	
~	~	Victoria Bell	
~	~	Martyn Blundell	
~	~	Lauren Blunt	
~	~	Jo Blurton	
~	~	Mark Brennan	
~	~	Catherine Brookman	
~	~	Shelley Chamberlain	
~	~	Melody Cross	
~	~	Donna Cuffe	
~	~	Stacey Davies	
~	~	Sue Forbes	
~	~	Angela Foulds	
~	~	Helen Greedus	
~	~	Andrew Harford-Brown	
~	~	Helen Kimber	
~	~	Magdi Kirollos	
~	~	Ingrid Koehler	
~	~	Sally Maddison	
~	~	Catherine Marshall	
~	~	Robert Mason	
~	~	Seamus McDermott	
~	~	Jorg Michels	
~	~	Lyn Micklewright	
~	~	Amy Millington	
~	~	Sophie Norman	
~	~	Louise Paatz	
~	~	Janet Palmer	
~	~	Kirsty Pearce	
~	~	Christine Rawlings	

INVESTIGATORS AND COLLABORATORS: SITE STAFF
Staff on site delegation logs

City	Care_Site	Person_Name	Site_PI
~	~	Sarah Rees	
~	~	Rajaguru Srinivasan	
~	~	Lorraine Thornton	
~	~	Elaine Vandecandalaere	
~	~	Amanda Vian	
~	~	Beverley Watkins	
~	~	Erica Watts	
~	~	Sally Wells	
~	~	Linda Welsh	
~	~	Sarah Wright	
Warrington, UK	Warrington Hospital	Isabel Syndikus	PI
~	~	Shaun Tolan	Co-I
~	~	Lucy Berresford	
~	~	Lisa Dobson (nee Child)	
~	~	Jade Keenan	
~	~	Duncan Knowles	
~	~	Lisa Lee	
~	~	Carrie Lowthian	
~	~	Rebecca Madew (nee Tinker)	
~	~	Nemonie Marriott	
~	~	Philip Reynolds	
~	~	Sandra Robinson	
~	~	Andrea Young	
Warwick, UK	Warwick Hospital	Andrew Chan	PI
~	~	Maggie Brown	
~	~	Judith Chettle	
~	~	Jacqui Harris	
~	~	Lyn Hartwell	
~	~	Julia Jones	
~	~	Linda Maher	
~	~	Helen Millage	
~	~	Emily Noonan	
~	~	Eilish O'Neill	

INVESTIGATORS AND COLLABORATORS: SITE STAFF
Staff on site delegation logs

City	Care_Site	Person_Name	Site_PI
~	~	Jackie Sears	
~	~	Lucy Shafiq	
~	~	Andrew Stockdale	
~	~	Donna Walsh	
~	~	Frances Walsh	
~	~	Jo Williams	
Westcliff on Sea, UK	Southend University Hospital	Imtiaz Ahmed	PI
~	~	Abby Cyriac	Co-I
~	~	David Tsang	Co-I
~	~	Sue Bowman	
~	~	Kelly Buckhorn	
~	~	Thomas Carr	
~	~	Olivia Chan	
~	~	Stuart Chandler	
~	~	Lesley Cranfield	
~	~	Tracey Davies	
~	~	Terry Dowling	
~	~	Lesley Googe	
~	~	Kathryn Hawkesford	
~	~	Andrew Ho	
~	~	Ken Kennedy	
~	~	Joana Kyte	
~	~	Richard Lodge	
~	~	Tanatswa Mabhoyi	
~	~	Katrina Maitland	
~	~	Lesley Nichols	
~	~	Shanas Noor	
~	~	Ololade Omodunbi	
~	~	Sreekanth Palvai	
~	~	Meera Patel	
~	~	Jan Prejbisz	
~	~	Amdadur Rahman	
~	~	Usha Ravichandran	

INVESTIGATORS AND COLLABORATORS: SITE STAFF
Staff on site delegation logs

City	Care_Site	Person_Name	Site_PI
~	~	Sheila Reece	
~	~	Rachel Sadan	
~	~	Naveed Sarwar	
~	~	Ryan Wong	
~	~	Nuhu Yaroson	
Weston Super Mare, UK	Weston General Hospital	Serena Hilman	PI
~	~	Thomas Bird	Co-I
~	~	Tom Wells	Co-I
~	~	Kathy Beard	
~	~	Sandra Beech	
~	~	Debbie Coles	
~	~	Donna Cotterill	
~	~	Harvey Dymond	
~	~	Symeon Eleftheriadis	
~	~	Rajesh Gamare	
~	~	Denise Leighton-Price	
~	~	Hugh Lloyd-Jones	
~	~	Jennifer Maby	
~	~	Andrew McKendrick	
~	~	Kristina Owens	
~	~	Dave Pack	
~	~	Glenn Saunders	
~	~	Dawn Simmons	
~	~	Marjorie Tomlinson	
~	~	Rachel Warinton	
~	~	Susan Wilkinson	
Whitehaven, UK	West Cumberland Hospital	Fiona Douglas	PI
~	~	Anil Kumar	PI
~	~	Angela Birt	
~	~	Christopher Brewer	
~	~	Alan Denholm	
~	~	Charlotte Eyles	
~	~	Grace Fryer	

INVESTIGATORS AND COLLABORATORS: SITE STAFF
Staff on site delegation logs

City	Care_Site	Person_Name	Site_PI
~	~	Tim Marshalsea	
~	~	Patricia Nicholls	
~	~	Jonathan Nicoll	
~	~	Muhammad Rahman	
~	~	Norma Sidek	
~	~	Fiona Spence	
~	~	Jenna Wildey	
~	~	Beverley Wilkinson	
~	~	Joanne Wilkinson	
~	~	Fergus Young	
Wigan, UK	Royal Albert Edward Infirmary	Anna Tran	PI
~	~	Euan Green	Co-I
~	~	Steve Adejumo	
~	~	Julie Barnes	
~	~	David J Bell	
~	~	Jenny Bradshaw	
~	~	Jennifer Cannon	
~	~	Richard Cowan	
~	~	Louise Devereaux	
~	~	Alison Doran	
~	~	Sonia Evans	
~	~	Diane Forrest	
~	~	Elian Green	
~	~	Paul Higham	
~	~	Claire Hill	
~	~	Andrew Hudson	
~	~	Su Kim	
~	~	Sarah Kirk	
~	~	Andrew McPartlin	
~	~	Karen Moss	
~	~	Muthuswarmy Nagarajan	
~	~	Michael Parks	
~	~	Angela Power	

INVESTIGATORS AND COLLABORATORS: SITE STAFF
Staff on site delegation logs

City	Care_Site	Person_Name	Site_PI
~	~	Catherine Redshaw	
~	~	Tonia Louise Selby	
~	~	Dianna Thompson	
~	~	Zoe Trumper	
~	~	Marissa Walters	
Winchester, UK	Royal Hampshire County Hospital	Sangeeta Paisey	PI
~	~	Rao Vuyyuru	Co-I
~	~	Andrew Adamson	
~	~	Louise Beattie	
~	~	Julie Conti	
~	~	Victoria Corner	
~	~	Angela Firth	
~	~	Liz Happle	
~	~	Ina Hoad	
~	~	Lesley Hollister	
~	~	Abigail Hughes	
~	~	Lauriane Kerwood	
~	~	Carley Merritt	
~	~	Christina Narh	
~	~	Fasar Sarwar	
~	~	Jackie Smith	
~	~	Anna Song	
Wolverhampton, UK	New Cross Hospital	Ian Sayers	PI
~	~	Syed Abdullah Bukhari	Co-I
~	~	Amrita Solanki	Co-I
~	~	Amarpal Bains	
~	~	Ann Bentley	
~	~	Emily Carter	
~	~	Vanda Carter	
~	~	Mark Churn	
~	~	Peter Cooke	
~	~	Georgi Georgiev	
~	~	Anna Grant	

INVESTIGATORS AND COLLABORATORS: SITE STAFF
Staff on site delegation logs

City	Care_Site	Person_Name	Site_PI
~	~	Kay Hadlington	
~	~	Uttara Karnik	
~	~	Kelly Kauldhar	
~	~	Pek Keng-Koh	
~	~	Christine Kirk	
~	~	Claire Lomas	
~	~	Nataliya Martynyuk	
~	~	Joanne Mundy	
~	~	Renita Pawaroo	
~	~	Bajinder Rai	
~	~	Jason Rogers	
~	~	Sharon Rudge	
~	~	Gurminder Sahota	
~	~	Emma Sharman	
~	~	Debbie Spruce	
~	~	Arvind Tripathy	
~	~	Davina Warrender	
Worcester, UK	Worcestershire Royal Hospital	Lisa Capaldi	PI
~	~	Menna Fouda	Co-I
~	~	Kamalnayan Gupta	Co-I
~	~	Ayyaz Munawar	Co-I
~	~	Susan Anderson	
~	~	Khin Aye	
~	~	Dagmara Bak	
~	~	Jo Bowen	
~	~	Kristy Cleary	
~	~	Sue Davies	
~	~	Paul Flinders	
~	~	Janet Forkes	
~	~	Monica Gauntlett	
~	~	Alison Harrison	
~	~	Jennifer Healey-Mariano	
~	~	Hayley Hodson	

INVESTIGATORS AND COLLABORATORS: SITE STAFF
Staff on site delegation logs

City	Care_Site	Person_Name	Site_PI
~	~	Amanda Holdsworth	
~	~	Bartlomeij Kurec	
~	~	Zeeshaan Parvez	
~	~	Jayadevkumar Pawadshetti	
~	~	Heather Perry	
~	~	Patricia Rimell	
~	~	Alison Rosoman	
~	~	Asha Sivapalasuntharam	
~	~	Sally Stringer (pr. Davis)	
~	~	Jacob Taylor	
~	~	Helen Tranter	
~	~	Jayne Tyler	
~	~	Ann White	
~	~	Nicola Williams	
Worthing, UK	Worthing Hospital	Ashok Nikapota	PI
~	~	David Bloomfield	Ex-PI
~	~	Irvin Balagosa	
~	~	Stephanie Brown	
~	~	Fiona Castell	
~	~	Dawn Crowe (nee Hughes)	
~	~	Marian Flynn-Batham	
~	~	Linda Folkes	
~	~	Sarah Funnell	
~	~	Jeanette Gilbert	
~	~	Raquel Gomez-Marcos	
~	~	Celia Gonzalez	
~	~	Sarah House	
~	~	Helen Jones	
~	~	Sarah Kimber	
~	~	Jordi Margalef	
~	~	Leanne Mills	
~	~	Sally Moore	
~	~	George Plataniotis	

INVESTIGATORS AND COLLABORATORS: SITE STAFF

Staff on site delegation logs

City	Care_Site	Person_Name	Site_PI
~	~	Susan Rockall	
~	~	Matthew Smith	
~	~	Yvette Thirlwall	
~	~	Tan Tsawayo	
~	~	Nikki Turner	
~	~	Wendy Wood	
Yeovil, UK	Yeovil District Hospital	Tim Porter	PI
~	~	Sabri Ahmed	Co-I
~	~	Erica Beaumont	Co-I
~	~	Joanna Allison	
~	~	Zenaida Armstrong	
~	~	Claire Barron	
~	~	Nigel Beer	
~	~	Kate Beesley	
~	~	Debbie Cole	
~	~	Sunil Daryanani	
~	~	Sarah De Bruijn	
~	~	David Donaldson	
~	~	Tracey Duckett	
~	~	Shirley Fox	
~	~	Emma Gray	
~	~	Hassan Hameed	
~	~	Michelle Kotze	
~	~	David Laws	
~	~	Jess Perry	
~	~	Lucy Pippard	
~	~	Charlotte Reeves	
~	~	Kerry Rennie	
~	~	Geoffrey Sparrow	
~	~	Amanda Sweet	
~	~	Pamela White	
York, UK	University of York	Mark Schulpher	
York, UK	York District Hospital	Paul Brittain	

INVESTIGATORS AND COLLABORATORS: SITE STAFF
Staff on site delegation logs

City	Care_Site	Person_Name	Site_PI
~	~	Claire Brookes	
~	~	Flor Davies	
~	~	Cheryl Donne	
~	~	Mark Fearnley	
~	~	Sally Gilroy	
York, UK	York Teaching Hospital	Joji Joseph	PI
~	~	Ben Blake-James	Co-I
~	~	David Bottomley	Co-I
~	~	Russ Wilson	Co-I
~	~	Mark Aldous	
~	~	Ornella Belvedere	
~	~	Paul Brittain	
~	~	Claire Brookes	
~	~	Poppy Cottrell-Howe	
~	~	Tracey Dorey	
~	~	Mark Elliott	
~	~	Richard Evans	
~	~	Fereshteh Fallah	
~	~	Jayne Hammond	
~	~	Tom Hearfield	
~	~	Jo Ingham	
~	~	Laura Jeffery	
~	~	Kay Kell	
~	~	Prithivi Maheswaran	
~	~	Lisa Mole	
~	~	Daniel Petty	
~	~	Kate Ritchie	
~	~	Abigail Rowbotham	
~	~	Paula Strider	
~	~	Debora Twydell	
~	~	John Wightman	
~	~	Paul Wood	
~	~	Emily Worrall	

INVESTIGATORS AND COLLABORATORS: SITE STAFF
Staff on site delegation logs

City	Care_Site	Person_Name	Site_PI
Basel, CH	Universitätsspital Basel	Cyrill Rentsch	PI
~	~	Frank Stenner-Liewen	Co-I
~	~	Alexander Bachmann	
~	~	Nicole Ebinger	
~	~	Mana Farsad	
~	~	Eloise Kremer	
~	~	Simone Marini	
~	~	Kristina Muller	
~	~	Nicole Neumann	
~	~	N Ott	
~	~	Heike Puschel	
~	~	Christoph Rochlitz	
~	~	Bettina Seifest	
~	~	M Timmermann	
~	~	Stephen Wyler	
Bellinzona, CH	Istituto Oncologico della Svizzera Italiana	Ricardo Pereira Mestre	PI
~	~	Enrico Roggero	PI
~	~	Ngwa Che Azinwi	
~	~	Carolina De Almeida	
~	~	Maria Delgrande	
~	~	Vittoria Espeli	
~	~	Eloise Kremer	
~	~	Anna Llado	
~	~	Barbara Marongiu	
~	~	Michele Moro	
~	~	Gianfranco Pesce	
~	~	Sabine Van Den Bosch	
Berne, CH	Inselspital (University Hospital Berne)	Jörg Beyer	PI
~	~	Daniel Aebersold	
~	~	Anna-Katharina Herrmann	
~	~	Eloise Kremer	
~	~	Anselm Lafita	
~	~	Susan Meierhans	

INVESTIGATORS AND COLLABORATORS: SITE STAFF
Staff on site delegation logs

City	Care_Site	Person_Name	Site_PI
~	~	Timo Nannen	
~	~	Kathi Ochsner	
~	~	Simone Rimoldi	
~	~	Beat Roth	
~	~	George Thalmann	
~	~	Barbara Uhlmann	
~	~	Antje Ulrich	
~	~	Martin Waeber	
Biel, CH	Spitalzentrum Biel	Markus Borner	PI
~	~	Silvia Hanselmann	
~	~	Eloise Kremer	
~	~	Annette Winkler Vatter	
~	~	Béatrice Zimmerli Schwab	
Chur, CH	Kantonsspital Graubünden	Raeto Strebel	PI
~	~	Richard Cathomas	
~	~	Dirk Kienle	
~	~	Eloise Kremer	
~	~	Gabriela Manetsch	
~	~	M Mark	
~	~	Radmila Moudry	
~	~	Michael Schwitter	
~	~	Roger von Moos	
Lausanne, CH	Centre Hospitalier Universitaire Vaudois (CHUV)	Dominik Berthold	PI
~	~	May-Lucie Meyer	Co-I
~	~	Alice Abdallah	
~	~	Tewfik Abedlaziz	
~	~	Veronica Aedo	
~	~	Catherine Bender	
~	~	Galaad Bernard	
~	~	Yohan Boillat	
~	~	Floriane Bouilly	
~	~	Anna-Sophia Briod	
~	~	Carmen Castagna	

INVESTIGATORS AND COLLABORATORS: SITE STAFF
Staff on site delegation logs

City	Care_Site	Person_Name	Site_PI
~	~	Anabela Costa	
~	~	Antonella Diciolla	
~	~	Nathalie Divorne	
~	~	Akram Farhat	
~	~	Sabine Galland	
~	~	Sylvie Haudidier	
~	~	Fernanda Herrera	
~	~	Agnes Hiou Feige	
~	~	Nicole James Faresse	
~	~	Patrice Jichlinski	
~	~	Eloise Kremer	
~	~	Fabrice Lalubin	
~	~	Sofiya Latifyan	
~	~	Cynthia Leclerc	
~	~	Margaret McLauchlan	
~	~	Benangene Midez	
~	~	Sophia Murel	
~	~	Kaniana Ntanga Muambayi	
~	~	Rebecca Oppenheim	
~	~	Angela Orcurto	
~	~	Louis Parisod	
~	~	Claire Perrinjaquet	
~	~	Alexandra Rideau	
~	~	Hans-peter Roth	
~	~	Marc Schnety	
~	~	Cosette Schuler	
~	~	Norlene Silva	
~	~	Sandra Toffanin	
~	~	Geert Van Driessche	
~	~	Sophie Voegtlin	
~	~	Aline Voidey	
~	~	Celine Yerly	
~	~	Jean-Philippe Zurcher	

INVESTIGATORS AND COLLABORATORS: SITE STAFF
Staff on site delegation logs

City	Care_Site	Person_Name	Site_PI
Liestal, CH	Kantonsspital Liestal	Vanessa Fuhrer	
~	~	Eloise Kremer	
~	~	Andreas Lohri	
~	~	Simone Marini	
St Gallen, CH	Kantonsspital St Gallen	Daniel Engeler	PI
~	~	Aurelius Omlin	Co-I
~	~	Christian Rothermundt	Co-I
~	~	Christoph Schwab	Co-I
~	~	Dominik Abt	
~	~	Silke Gillessen	
~	~	Claudia Hormann	
~	~	Mannel Jungi	
~	~	Eloise Kremer	
~	~	Sigrid Patel	
~	~	Stefan Prensser	
~	~	Sibylle Schapper	
~	~	Karin Zuern	
~	~	Karin Zurn	
St. Gallen, CH	Klinik fur Urologie	Claudia Hormann	
~	~	Sibylle Schapper	
Winterthur, CH	Kantonsspital Winterthur	Hubert John	Ex-PI
~	~	Beatrice Brinkers	
~	~	Natalie Fisher	
~	~	Nicole Kradolfer	
~	~	Eloise Kremer	
~	~	Claudia Langer	
~	~	Muller	
~	~	Veronika Nagy	
~	~	Martina Pfitzner	
~	~	Miklos Pless	
~	~	Sabina Schacher	
~	~	SusyAnn Shaw	
~	~	Cindy Wanger	

INVESTIGATORS AND COLLABORATORS: SITE STAFF
Staff on site delegation logs

City	Care_Site	Person_Name	Site_PI
Zurich, CH	Hirslanden Medical Centre	Razvan Popescu	PI
~	~	Katja Kilcher	
~	~	Eloise Kremer	
~	~	Helen Leemann	
~	~	Eva Lehmann Fueter	
~	~	Sylvie Nuc	
~	~	Klaus Schalk	
~	~	Belinda Schegg	
~	~	Louise Seiler	
~	~	Melanie Stahel	
~	~	Michelle Suppiger	
Zurich, CH	Triemlispital	Donat Durr	PI
~	~	Maximillian Asanger	
~	~	Camillo Cetuzzi	
~	~	Irene Hones	
~	~	Eloise Kremer	
~	~	Alexandra Pfister	
~	~	Karin Scheuch	
~	~	Daniele Siciliano	
~	~	Stefan Suter	
Zurich, CH	University Hospital Zurich	Daniel Fetz	
~	~	Eloise Kremer	
~	~	Michael Muntener	
~	~	Cedric Poyet	
~	~	Frank Stenner-Liewen	

INDUSTRY COLLABORATORS

Clovis Oncology
Support for the STAMPEDE trial has been provided by Clovis Oncology.

Janssen
Laurent Antoni
Joaquin Casariego garcia luben
Ilde Herrygars
Florence Lefresne
Rod Murphy
Mohamed Samir
Support for the STAMPEDE trial has been provided by Janssen.

Novartis
Support for the STAMPEDE trial has been provided by Novartis Pharmaceuticals UK Limited.

Sanofi-Aventis
Paul Cadle
Christine Geffriaud-Ricouard
Support for the STAMPEDE study has been provided by Sanofi-Aventis.

PARTICIPANTS

More than 12,000 people have chosen to participate in STAMPEDE. In addition to their clinical teams, they have been supported by family, friends and other key people. Every person who has participated in the trial is appreciated by the trial team and should be appreciated by the wider public. The findings from clinical trials can change practice for the future, but clinical trials only happen because people find the time and make the effort to support them. Thank you.
